# Evolving better solvate electrolytes for lithium secondary batteries†

**DOI:** 10.1039/d4sc01492h

**Published:** 2024-04-11

**Authors:** Frederik Philippi, Maleen Middendorf, Keisuke Shigenobu, Yuna Matsuyama, Oriele Palumbo, David Pugh, Taku Sudoh, Kaoru Dokko, Masayoshi Watanabe, Monika Schönhoff, Wataru Shinoda, Kazuhide Ueno

**Affiliations:** a Department of Chemistry and Life Science, Yokohama National University 79-5 Tokiwadai, Hodogaya-ku Yokohama 240-8501 Japan; b University of Münster Corrensstraße 28/30 48149 Münster Germany; c Research Institute for Interdisciplinary Science, Okayama University Okayama 700-8530 Japan; d Consiglio Nazionale delle Ricerche Istituto dei Sistemi Complessi, P.le Aldo Moro 5 00185 Rome Italy; e Department of Chemistry, Britannia House, Kings College London 7 Trinity Street London SE1 1DB UK; f Advanced Chemical Energy Research Centre, Advanced Institute of Sciences, Yokohama National University 79-5 Tokiwadai, Hodogaya-ku Yokohama 240-8501 Japan

## Abstract

The overall performance of lithium batteries remains unmatched to this date. Decades of optimisation have resulted in long-lasting batteries with high energy density suitable for mobile applications. However, the electrolytes used at present suffer from low lithium transference numbers, which induces concentration polarisation and reduces efficiency of charging and discharging. Here we show how targeted modifications can be used to systematically evolve anion structural motifs which can yield electrolytes with high transference numbers. Using a multidisciplinary combination of theoretical and experimental approaches, we screened a large number of anions. Thus, we identified anions which reach lithium transference numbers around 0.9, surpassing conventional electrolytes. Specifically, we find that nitrile groups have a coordination tendency similar to SO_2_ and are capable of inducing the formation of Li^+^ rich clusters. In the bigger picture, we identified a balanced anion/solvent coordination tendency as one of the key design parameters.

## Abstract 2

Electrolytes are a key component of lithium batteries with significant potential for improvement. Here we present strategies to navigate the large chemical space of electrolytes by combining the strengths of theoretical and experimental approaches.

## Graphical abstract

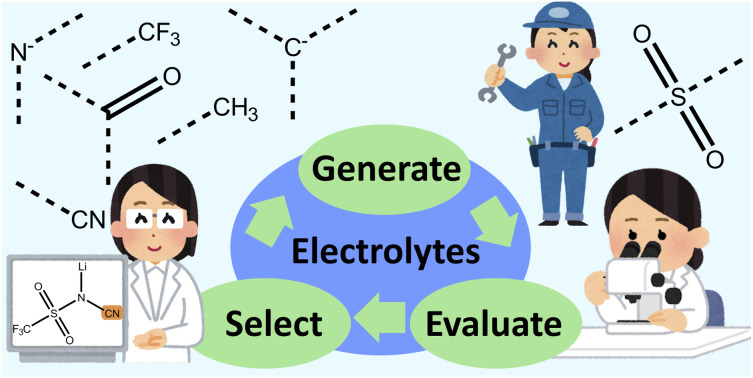

## Introduction

Batteries have become an indispensable part our daily life. Especially lithium-ion batteries transformed society like few other technologies have in the past. The vast majority of mobile applications such as smartphones, tablets, and electric cars rely on lithium-ion batteries. However, the development of lithium ion batteries is far from over, and many problems remain to be solved.^1^

Battery optimisation is an incredibly complex problem for two reasons. First, the battery as a final product needs to fulfil a wide variety of requirements such as safety, energy density, power density, efficiency, cost, lifetime, *etc.* Second, from a design perspective, the battery itself consists of many components, each of which can be realised in several ways, with complex interactions and interfaces between them.

One of the key components in a battery is the electrolyte.^2^ Like other parts of the battery, there are many simultaneous requirements for an ideal electrolyte, and compromises are often inevitable. Conventional electrolytes such as Li[PF_6_] diluted in organic solvents have a number of disadvantages, first and foremost limited stability, which in turn presents a safety hazard in certain cases. Several cases of thermal runaways of lithium-ion batteries have gained worldwide public attention.^3^

Another important requirement of an ideal electrolyte is a high lithium transference number. The lithium transference number (*t*^+^_Li_) is the proportion of the total (ionic) current which is due to the movement of lithium cations. Ideally, this number would be close to unity. In reality, the lithium transference number of conventional electrolytes is rather low, for example ≈0.07 for 1 M Li[PF_6_] in EC/DMC.^4^ As a result, a large proportion of the current is lost to the transport of ionic species other than lithium. Thus, the efficiency of the battery is reduced significantly, since energy is wasted building up and maintaining an undesired concentration gradient in the electrolyte during charging and discharging.

**Fig. 1:**
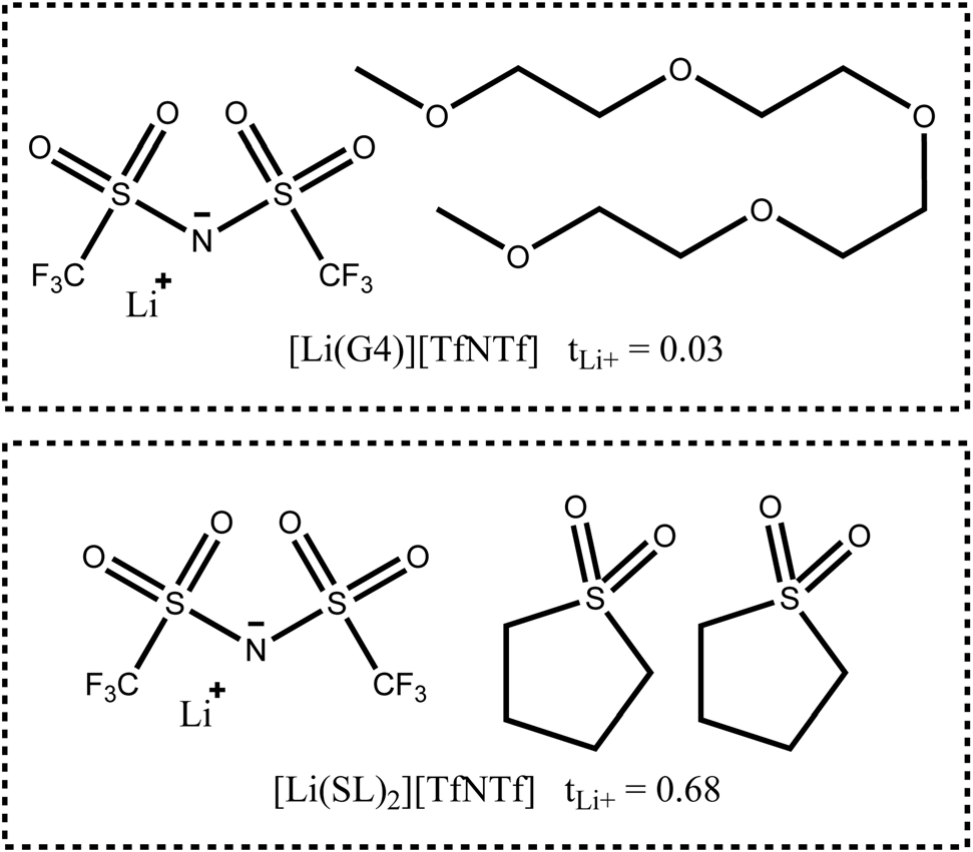
Two typical HCEs with very different lithium transference numbers.^11^

In this work, we will focus on highly concentrated electrolytes (HCEs). Compared to conventional electrolytes with a salt concentration around 1 M, HCEs have much higher salt concentration usually exceeding 3 M. HCEs have recently attracted attention as they offer beneficial properties such as electrochemical and thermal stability, low volatility, reduced flammability, *etc.*^5–9^ The use of HCEs, however, does not guarantee high transference numbers, see the two examples in Fig. 1. The tetraglyme (G4) based HCE shown here exhibits a transference number as low as 0.03, inferior to conventional electrolytes.^10,11^ Replacing tetraglyme as a solvent with sulfolane (SL) increases the transference number significantly, with the ionic current now being dominated by the lithium cations.^11^

The high transference number of many sulfolane based electrolytes has been qualitatively rationalised with the formation of lithium network structures bridged by sulfolane and anion molecules, resulting in an efficient hopping conduction mechanism of the lithium cations.^11–15^ Neat sulfolane itself is long known to crystallise into a plastic phase near room temperature,^16^ and the rotational freedom of sulfolane in sulfolane based HCEs is likely related to the high lithium mobility.^14^ This prevalence of hopping conduction in sulfolane as solvent compared to the vehicular mechanism often observed in carbonates or glymes is what led us to choose sulfolane as the main solvent for this study.^9,17,18^ Even the very small monoglyme (G1), in which vehicular transport is less pronounced compared to G4, leads to transference numbers much lower than those of sulfolane in HCEs (*e.g. t*_Li^+^_ = 0.35 for [Li(G1)_2_][TfNTf]).^8^

In HCEs, it is necessary to consider correlations between ions to develop a quantitative understanding of lithium transport. Simple models such as the Nernst–Einstein relation, neglecting ion correlations, fail when the concentration of ions becomes high. Thus, in systems such as ionic liquids and HCEs, cross correlations must be considered, see the section ‘ion correlations’ for more details.^19,20^

In order to quantify the drift velocity of single constituents of an electrolyte and their respective dynamic correlations, electrophoretic NMR (eNMR) has entered the field of concentrated electrolytes.^21,22^ Here, the electrophoretic mobility of each species can be determined directly. To this end, the sample is subjected to an external electric field pulse and the migration of cation, anion, and solvent is observed. The method is not new,^23,24^ but application to concentrated electrolytes requires a dedicated experimental setup and special procedures.^21,25^ A key finding was the direct detection of vehicular Li transport in net negatively charged clusters in salt-in-ionic liquid systems, as the observed lithium drift direction occurs against the electric field.^26–28^ Meanwhile, in the field of electrolyte development, eNMR has been recently used to great success, and has proven to be a highly relevant and powerful method.^29–34^ In particular, it gives valuable information to understand ion–ion dynamic correlations and to obtain Onsager coefficients, as demonstrated for solvate electrolytes.^32,35^

The impact of battery materials on health and environment has recently gained attention as additional design constraint. In particular, it is desirable to avoid the use of fluorinated electrolytes which have the potential to become persistent pollutants.^36–38^ Fluorination is a widely employed strategy to improve electrolyte properties, and the battery chemistry is often designed for fluorinated electrolytes.^39^ Hence, such a critical change to the electrolyte composition will also require a redesign of other battery components and will require several iterations of optimisation.

The design of a battery or even just one component cannot be achieved in just one step. Instead, it is often necessary to follow an iterative, evolutionary approach similar to the genetic algorithm which has been successfully applied to optimisation problems in other fields. Thus, in each iteration, a given population of candidates is modified and their performance evaluated. The iterative optimisation of complex systems is key in physical chemistry. The reason for this is not just the complexity of exploring chemical space, but also that usually new knowledge is gained in each iteration, for example new structure–property relationships. This new knowledge helps to heuristically select a better population in the next iteration. It is furthermore important to keep successful building blocks/design elements while also introducing entirely new ones to efficiently explore the optimisation space. In a sense, the recent trend towards environmentally more benign electrolytes is akin to a change in the performance evaluation (fitness function).

In this work, we follow the philosophy of an iterative optimisation by applying targeted modifications to an established HCE. A targeted modification is a small, deliberate change in the molecular structure of a chemical system.^40^ This change is deliberate in the sense that confounding variables are minimised. Our focus is put on changes to the anion. For example, we investigate nitrile functionalised anions such as [TfNCN]^−^ and partly fluorinated anions such as [TfNMs]^−^ or [TfNAc]^−^. However, it will follow naturally from our observations that changes to the solvent are also necessary. Hence, we also present a small number of novel HCE systems with unusual salt–solvent combinations to help guide future iterations of the evolution of electrolytes.

## Results and discussions

### Outline and screening of suitable anions

**Fig. 2:**
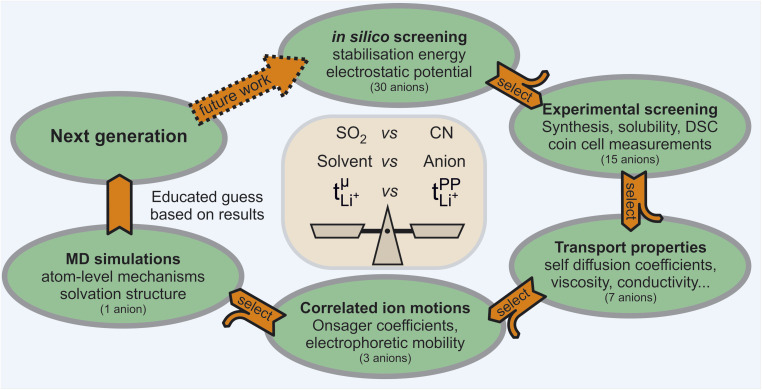
Schematic depiction of the tiered approach used in this manuscript. In each tier, the most interesting and promising systems are studied with a more sophisticated method in the next tier, reducing the number of anions by half. The combined results allow detailed insight into complex phenomena such as ion association and are used to suggest electrolyte systems for the next generation.

In this work, we chose a tiered approach which enabled us to assess many potential HCEs, Fig. 2. First, we performed an *in silico* screening of anions by means of *ab initio* simulations. Interesting candidates were then synthesised and investigated experimentally using small scale tests such as solubility and performance in a symmetric Li‖Li coin cell. Finally, a selection of HCEs emerging from the preliminary screening were studied in detail using more sophisticated techniques such as eNMR and MD simulation.

**Fig. 3:**
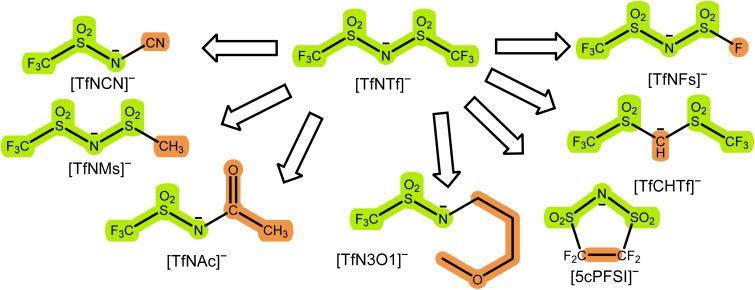
Examples of targeted modifications of the [TfNTf]^−^ anion used in this work. The parts which stem from the parent compound [TfNTf]^−^ are highlighted in green, the modified parts leading to new structures are highlighted in orange.

The initial selection of ions was chosen using targeted modifications of the bis(trifluoromethylsulfonyl)imide [TfNTf]^−^ anion, Fig. 3. This weakly coordinating anion is also known as bistriflimide, [NTf_2_]^−^, TFSI, or TFSA and is commonly used in concentrated electrolytes.^41^ Here we chose abbreviations which highlight the modifications, see also the nomenclature overview in the ESI, Section 1.† Previous studies on Onsager coefficients suggest that very high transference numbers exceeding 0.7 should be possible if cation–anion correlation were to be increased starting from [TfNTf]^−^ based electrolytes and sulfolane as solvent.^11,42^ This design requirement was the rationale for choosing anions like [TfN3O1]^−^ or [TfNCN]^−^ with coordination sites that can interact with lithium, but also anions of general higher basicity and coordination ability such as [TfNMs]^−^ or [TfNAc]^−^ due to their lower degree of fluorination.

Most of the anions shown in Fig. 3 have been studied previously from the perspective of conformational flexibility.^43^ We define conformational flexibility as the capability of a molecule to easily change its shape. In other words, a flexible system has many thermally accessible minimum energy structures that are significantly different in terms of their shape and separated by small energy barriers. For [TfNTf]^−^, the relevant mode is rotation around the two N–S bonds, *i.e.* the reorientation of the side chains. It is important to consider conformational flexibility since this is one of the key confounding variables which might impact transport properties. [TfNFs]^−^ and [TfNTf]^−^ are of similar flexibility, differing only in their symmetry.‡‡When compared to [TfNTf]^−^, a preferable anion is [PfNFs]^−^ rather than [TfNFs]^−^, since [TfNTf]^−^ and [PfNFs]^−^ have the same molecular weight and volume and are thus more suitable from a perspective of targeted modifications. We have included this anion in the ESI, however as shown previously for ionic liquids, the impact of mass is negligible. The half-fluorinated [TfNAc]^−^ is, like [TfNTf]^−^, a very flexible anion. [TfNMs]^−^ is intermediate as rotation is only energetically feasible for one of the two N–S bonds. In contrast, [TfCHTf]^−^ and [5cPFSI]^−^ are rigid, non-flexible anions. The direct comparison of [TfNCN]^−^ and [TfN3O1]^−^ in terms of flexibility is not possible due to their different backbone structure. However, the corresponding rotational energy barrier is relatively low for [TfNCN]^−^, see ESI Section 16.†

**Fig. 4:**
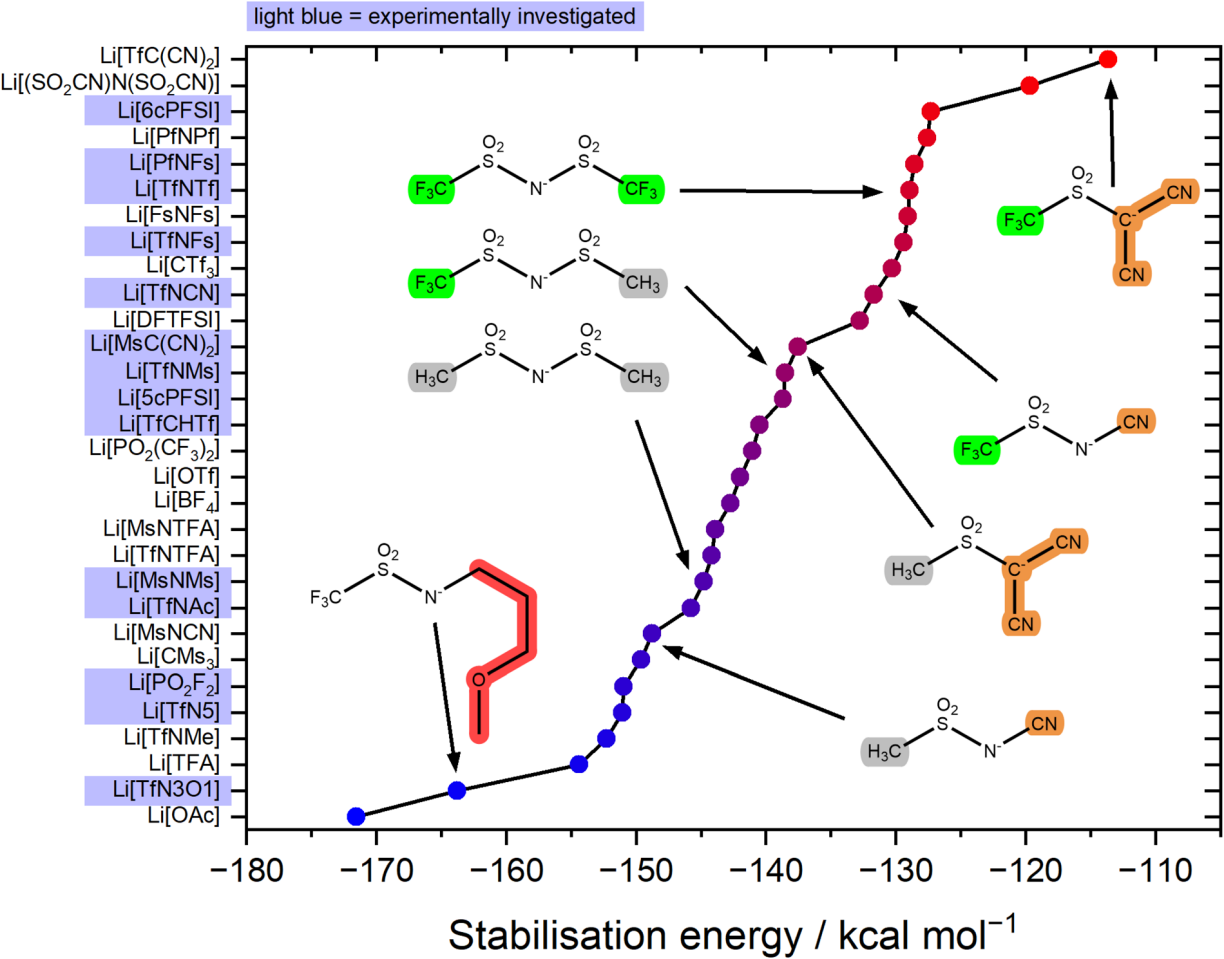
Stabilisation energy of different Li^+^-anion pairs in the gas phase. Anions which are not considered targeted modifications of [TfNTf]^−^ are also shown for context within the spectrum of currently used anions for electrolytes.

The stabilisation energy Δ*E*_form_ gives a first approximation of how favourable the complex formation is between a given anion and Li^+^. The stabilisation energies for the Li^+^-anion pairs in this work range from approximately −170 kcal mol^−1^ to −110 kcal mol^−1^, Fig. 4 and ESI Section 16.† Comparing [TfNTf]^−^ with [TfNMs]^−^ and [MsNMs]^−^, anion fluorination tentatively yields complexes which dissociate easily. This generally desired effect is also evident comparing the pairs of fluorinated/non-fluorinated anions containing nitrile groups shown in Fig. 4. However, the stabilisation energy fails to capture the differences between anions such as [TfNTf]^−^, [TfNCN]^−^, and [TfN3O1]^−^, as will be discussed below.

**Fig. 5:**
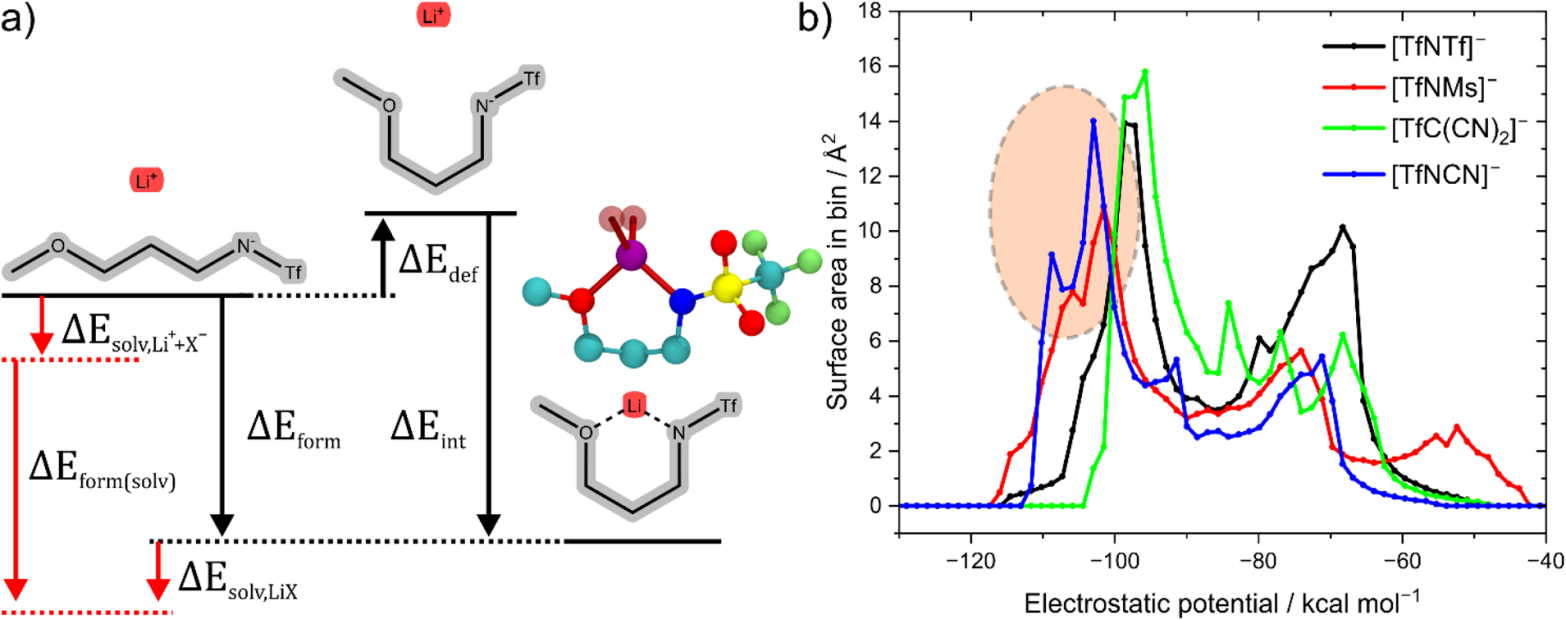
(a) Energy definitions in this work. For some ions such as Li[TfN3O1]^−^, the deformation energy includes a change in conformation, see also the corresponding geometry from the experimental crystal structure shown as CPK model. H atoms were omitted in the geometry from the crystal structure. The red part includes the energies associated with solvating the ions and the ion pair from the gas phase, respectively. (b) Surface area histogram of the electrostatic potential for the isolated anions.

The stabilisation energies in Fig. 4 were calculated in the gas phase. In principle, solvation can be included as shown in Fig. 5a. This aspect is explored in the ESI Section 16.3.† Solvation was found to not significantly affect the qualitative conclusions drawn based on the stabilisation energy in this work. However, some of the changes in the presence of a solvent model are worth noting. For example, the most dissociative anion becomes [(SO_2_CN)N(SO_2_CN)]^−^ rather than [TfC(CN)_2_]^−^, and [BF_4_]^−^ is found to be similar to the weakly coordinating [TfNFs]^−^ (rather than [OTf]^−^ without solvent model).

For the experimental study, we selected the highlighted anions in Fig. 4 to cover a range of stabilisation energies. In the electrolyte, a more negative stabilisation energy of Li-anion pairs is expected to lead to increased cation–anion correlations. For electrolyte systems such as [Li(SL)_2_][TfNTf], this in turn is expected to lead to increased lithium transference numbers.^11,42^ Some anions were ruled out due to issues with stability, or accessibility. For example, partially hydrogenated anions have shown interesting properties, but are at present prohibitively expensive and difficult to access.^44,45^ Similarly, [(SO_2_CN)N(SO_2_CN)]^−^ is promising as a non-fluorinated weakly coordinating anion, but remains inaccessible synthetically to the best of our knowledge.^46,47^

One of the key limiting factors we encountered when studying the sulfolane based HCEs is solubility. Both at our desired molar ratio of salt:solvent of 1 : 2 and the less concentrated 1 : 3, the salts Li[6cPFSI], Li[5cPFSI], Li[TfN3O1], Li[TfN5], Li[MsNMs], and Li[MsC(CN)_2_] turned out to be insoluble or at least not fully miscible at 30 °C. A straightforward solution would be a change of solvent. For example, tetraglyme G4 can saturate the coordination environment of Li^+^ with weakly coordinating anions like [TfNTf]^−^, leading to solvate (or chelate) ionic liquids.^48–52^ Such a solvent might be more suitable for some of the salts/anions.

The solubility problems especially of Li[TfN3O1] can be rationalised with the coordination geometry of lithium, which is incorporated into a favourable 6 membered ring, Fig. 5a. Indeed, we were able to grow crystals both from sulfolane and tetraglyme as solvents, without the solvent participating in the coordination of Li^+^ in the single crystal X-ray structure. In these structures every lithium atom is coordinated by three anions and *vice versa*, two of the anions coordinating *via* SO_2_ oxygens and one anion chelating. The strong interaction specifically for Li[TfN3O1] is consistent with the high melting point of this salt reported in the literature (256 °C), especially compared to the analogous potassium salt K[TfN3O1] which was reported to melt at 51 °C.^53,54^ A possible strategy to use such salts would be a change of solvent, including polymers such as PEO.^54,55^

A drawback of the stabilisation energy is that it is based only on a single geometry, steric and entropic effects are largely neglected. Similarly, the stabilisation energy as presented here does not take into account the denticity and steric demand of the ligands. The [TfN3O1]^−^ anion has a very high stabilisation energy, however only two [TfN3O1]^−^ anions are sufficient to fully saturate a lithium cation. The low steric demand of some anions leaves room for additional anions or solvent molecules to coordinate to one central lithium cation, thus further lowering the energy.

The [TfNCN]^−^ anion is particularly interesting in this regard. It has been reported that coordination of lithium cations by the nitrile group are competitive to coordination by SO_2_ groups.^56–58^ Nürnberg *et al.* studied mixtures of aprotic ionic liquids and Li[TfNCN], and obtained properties consistent with structural diffusion/lithium ion hopping at high salt loading.^56^ At first sight this is in contrast to our *ab initio* simulations, which revealed that the lowest energy structure in Li[TfNTf] and also Li[TfNCN] involves bidentate coordination of lithium by SO_2_ groups. However, the monodentate –CN⋯Li coordination energy is not much lower, see ESI Section 16.†

Why does [TfNCN]^−^ coordinate stronger to lithium cations than [TfNTf]^−^, despite the similar stabilisation energy? First, there is a higher density of coordinating sites relative to the surface to the anion, since the –CF_3_ groups are essentially inert. Second, the interaction between these coordinating sites and Li^+^ is mostly electrostatic, as expected intuitively and confirmed *via* SAPT2+ and NBO calculations, *cf*. ESI Section 16.† Lastly, the critical aspect of the Li⋯anion interaction in these two anions is accessibility. To this end, Fig. 5b shows a histogram of the surface area of four anions with a certain value of the electrostatic potential (“sigma profile”, *cf*. ESI Section 16†). Areas of negative electrostatic potential are relevant for the interaction with cations such as Li^+^. While the surface of the [TfNTf]^−^ anion has parts with very negative electrostatic potential around −110 kcal mol^−1^, the actual area of these surfaces is small. In contrast, a significant proportion of the surface of [TfNCN]^−^ has an electrostatic potential in this range. Thus, using the electrostatic potential energy surface rather than the stabilisation energy, the [TfNCN]^−^ anion is more similar to the half-fluorinated [TfNMs]^−^ anion, and the [TfNTf]^−^ anion is more similar to the methanide anion [TfC(CN)_2_]^−^.

### Physicochemical characterisation

Syntheses and comprehensive physicochemical measurements of new HCEs are extremely time consuming, especially if the required lithium salts or precursors are not available commercially. To accommodate for these issues, we chose initial experimental screening methods which use small amounts of sample, specifically differential scanning calorimetry (DSC, as little as 10 μL electrolyte) and coin cell measurements (<0.3 mL electrolyte for three repeats). The required Li–Li symmetric coin cells and DSC pans can be assembled in a glovebox with relatively little effort. The potentiostatic polarisation experiment on a Li‖Li coin cell yields the lithium transference numbers which we aimed to optimise. Other indicators of general battery performance such as stability towards lithium metal and bulk resistance can also be inferred from impedance measurements on the same cell.

**Fig. 6:**
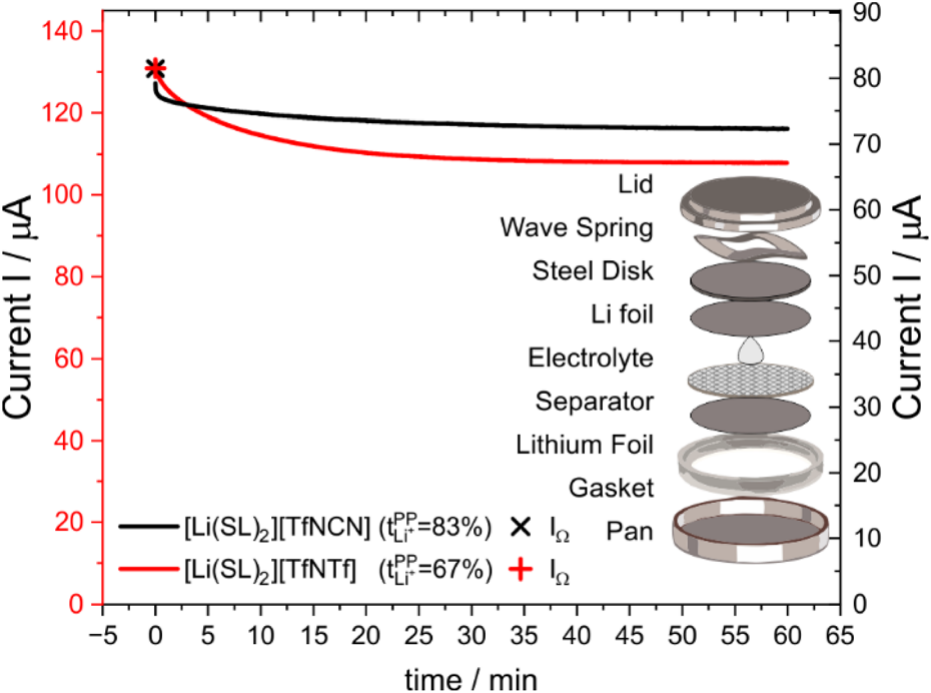
Current as a function of time during a typical potentiostatic polarisation experiment. The initial currents are different due to the different conductivity, curves have been shifted to better illustrate the differences in asymptotic behaviour. The insert shows an exploded view drawing of the symmetric Li‖Li coin cell used for the potentiostatic polarisation experiment.

Fig. 6 shows the current–time curve from a potentiostatic polarisation experiment on two HCEs, [Li(SL)_2_][TfNCN] and [Li(SL)_2_][TfNTf]. During the experiment, a constant potential (=‘potentiostatic’) is applied, and a small current passes through the cell. The electrodes are non-blocking towards lithium. The anions generally do not react (=‘anion blocking’) but still contribute to the (initial) current, resulting in a salt concentration gradient (=‘polarisation’). In Fig. 6, after ≈60 min steady state is reached. Thus, the anion migration is balanced by salt diffusion, and the current is exclusively due to Li^+^ transport including diffusion. The ratio of steady state and initial (bulk) current gives the lithium transference number, *cf*. ESI Section 7.† Hence, a flat curve with little change from the initial current is preferable. While this method has some limitations, it is an invaluable tool to quantify lithium transference.^4,59–65^ The full list of transference numbers measured as part of this work can be found in the ESI† (Section 7) together with their definitions.

**Fig. 7:**
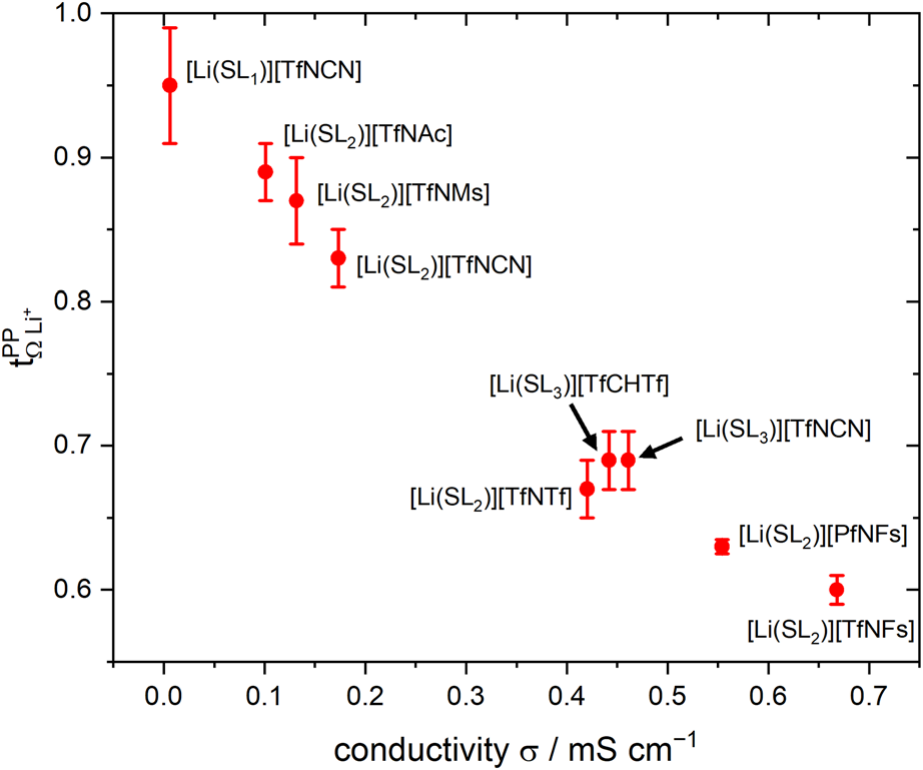
Trade-off between lithium transference number 
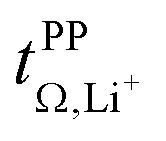
 and electrical conductivity *σ*. The error bars of the conductivity obtained from multiple repeats for [Li(SL)_2_][TfNCN], [Li(SL)_2_][TfNAc], and [Li(SL)_2_][TfNMs] are smaller than the size of the symbols.

Key physicochemical properties are summarised in Table 1, see ESI† for details. For the sake of simplicity, we only discuss lithium transference in terms of 
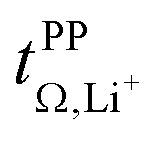
 which showed good agreement with 
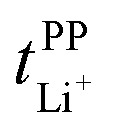
 and 
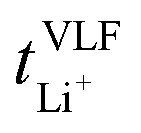
. For these samples, we also determined the electrical conductivity *σ* and the viscosity *η*. In general, high conductivity is desirable for good battery performance (together with a high transference number). Low viscosities facilitate handling of the electrolytes. In addition, viscosity is anticorrelated with dynamics, *i.e.* systems with low viscosity usually show fast ion diffusion and high conductivity. It is often observed empirically that transference and conductivity are anticorrelated.^63^ This rule of thumb also applies to the samples in this work, Fig. 7.

**Table 1:** Overview of key physicochemical properties for a selection of electrolytes used in this work. Unless otherwise mentioned, ‘lithium transference’ refers to 
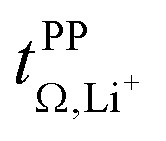

	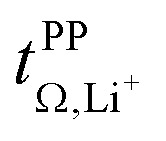	*T*_G_/°C	*c* ^30°C^/mol L^−1^	*σ*/mS cm^−1^	*η*/mPa s
[Li(SL)_2_][TfNTf]	0.67(2)	−74	2.97	0.42 (ref. 13)	616
[Li(SL)_3_][TfCHTf]	0.69(2)	−74	2.30	0.442	352
[Li(SL)_2_][TfNFs]	0.60(1)	−78	3.24	0.668	355
[Li(SL)_2_][PfNFs]	0.630(5)	−76	2.99	0.554	485
[Li(SL)_1_][TfNCN]	0.95(4)	−43		≈0.006	
[Li(SL)_2_][TfNCN]	0.83(2)	−72	3.43	0.173	1215
[Li(SL)_2.2_][TfNCN]	0.82(1)	−75	3.22		790
[Li(SL)_2.4_][TfNCN]	0.79(2)	−79	3.03		530
[Li(SL)_3_][TfNCN]	0.69(2)	−85	2.59	0.461	235
[Li(SL)_2_][TfNMs]	0.87(3)	−63	3.17	0.132	1962
[Li(SL)_2_][TfNAc]	0.89(2)	−68	3.24	0.101	600

Within the different fully fluorinated anions, the observed transference number showed little variation. The electric conductivity shows a small, expected dependence on size and substitution symmetry. Comparing [Li(SL)_2_][TfNTf] with the ‘asymmetric’ [Li(SL)_2_][PfNFs], which have the same molecular weight, [Li(SL)_2_][PfNFs] has a 32% higher conductivity. Similarly, comparing the two ‘asymmetric’ HCEs [Li(SL)_2_][PfNFs] and [Li(SL)_2_][TfNFs], the conductivity of the smaller [Li(SL)_2_][TfNFs] is higher by 21%. While the difference is small and comes at a cost of a reduced transference number, the fluorosulfonyl motif (–SO_2_F, abbreviated as ‘Fs’) has the advantages of in general better solid electrolyte interphase (SEI) formation and potentially a shorter lifetime if leaked into the environment.^66^

The influence of conformational flexibility is best discussed using the example of [Li(SL)_3_][TfCHTf]. The key difference between the otherwise extremely similar anions [TfCHTf]^−^ and [TfNTf]^−^ is conformational flexibility, with [TfCHTf]^−^ being rigid and [TfNTf]^−^ being flexible. Li[TfCHTf] did not dissolve in sulfolane in the salt : solvent ratio 1 : 2 at ambient conditions. It does, however, form a homogeneous solution at slightly elevated temperature which remains supercooled for days to weeks at 30 °C. Although we could not obtain a crystal structure of sufficient quality for publication using the crystals formed from the supercooled phase, a preliminary analysis suggested a solvent ratio in the crystal of 1 : 1. A rather high transference number of 0.78 was measured for supercooled [Li(SL)_2_][TfCHTf], however partial crystallisation during ageing cannot be ruled out. Interestingly, [Li(SL)_3_][TfCHTf] and [Li(SL)_2_][TfNTf] have virtually identical 
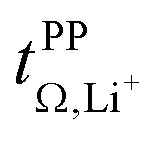
, *T*_G_ and *σ* despite the higher salt concentration and 1.8 times higher viscosity of [Li(SL)_2_][TfNTf].

The HCE [Li(SL)_2_][TfNCN] showed a high transference number of 0.83, a significant improvement compared to [Li(SL)_2_][TfNTf], however with a higher viscosity and thus lower conductivity. The viscosity (and, thus, conductivity) can be tuned with the solvent content. At a salt : solvent ratio of 1 : 3, [Li(SL)_3_][TfNCN] has the same transference number as [Li(SL)_3_][TfCHTf] and [Li(SL)_2_][TfNTf], but a significantly lower TG and viscosity. An extraordinarily high transference number of 0.95 could be obtained when increasing the salt : solvent ratio to 1 : 1. In contrast to [Li(SL)_2_][TfCHTf], we did not observe any crystallisation over the course of months for samples of [Li(SL)_1_][TfNCN]. While this demonstrates the excellent plasticising properties of this asymmetrically substituted nitrile functionalised anion, it comes at the expense of fluidity. [Li(SL)_1_][TfNCN] is so viscous that handling at ambient temperature is severely limited, and the measured conductivity was on the order of μS cm^−1^.

High transference numbers approaching 0.90 were found for the two HCEs based on half fluorinated anions, [Li(SL)_2_][TfNMs] and [Li(SL)_2_][TfNAc]. However, the conductivity of these two HCEs was even lower than that of [Li(SL)_2_][TfNCN]. For [Li(SL)_2_][TfNMs], this is not surprising since this was the sample with the highest observed viscosity in Table 1. In contrast, [Li(SL)_2_][TfNAc] has a viscosity just below that of [Li(SL)_2_][TfNTf], hence the conductivity is lower than what would be expected from a simple hydrodynamic model.

**Fig. 8:**
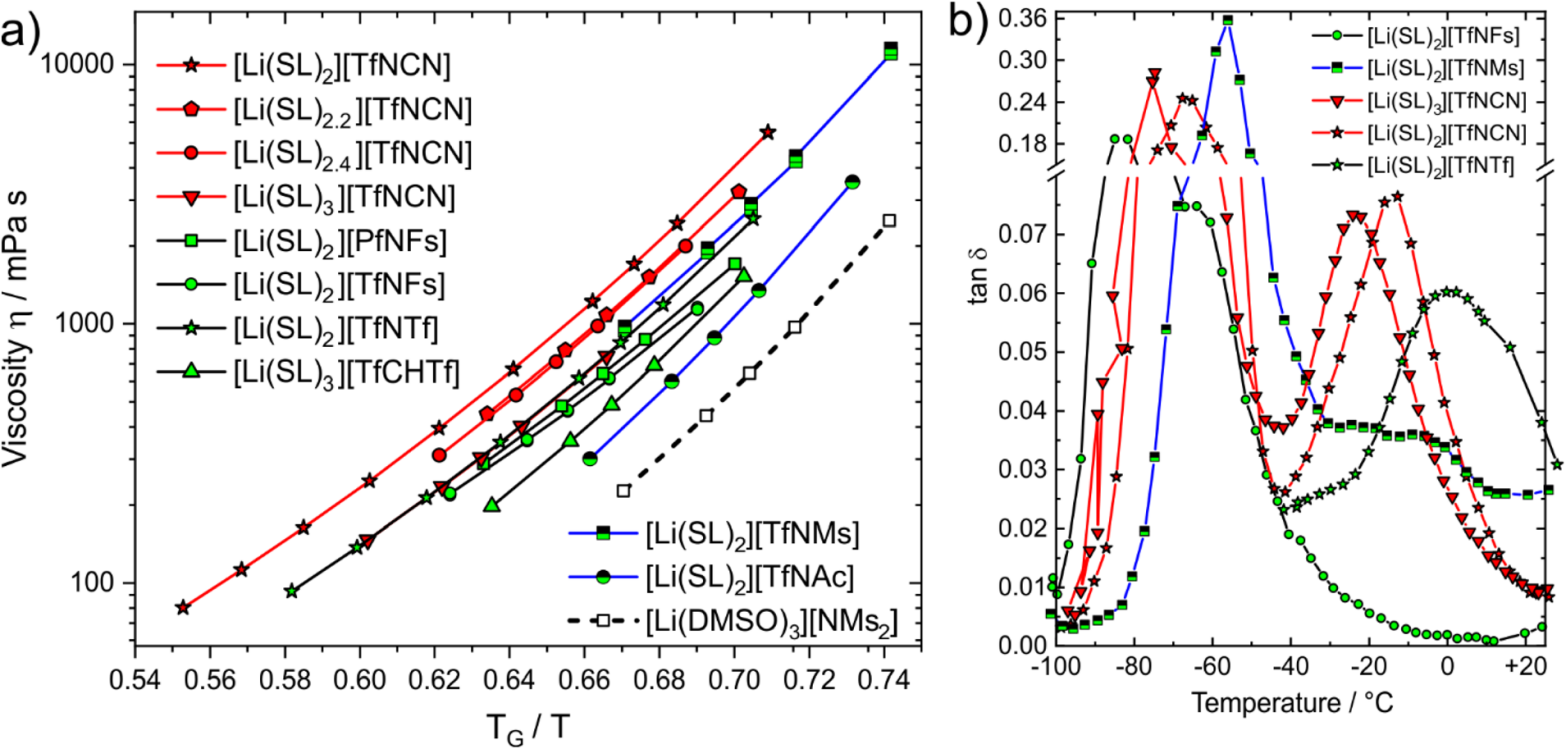
(a) Angell plot of a selection of electrolytes in this work. (b) DMTA results (loss tangent) for the cooling curve at a frequency of 5 Hz.

Dynamics slow down exponentially approaching a glass transition *T*_G_, hence the proximity to *T*_G_ affects transport properties. The Angell plot, Fig. 8a, compensates for this effect, allowing for a ‘fair’ comparison of systems with different glass transition temperatures. In some cases, the qualitative order changes. For example, we included the completely non-fluorinated electrolyte [Li(DMSO)_3_][MsNMs] in Fig. 8a. In the Angell plot, [Li(DMSO)_3_][MsNMs] has a lower viscosity than for example [Li(SL)_3_][TfCHTf], even though this is not the case when comparing at the same temperature (the viscosity of [Li(DMSO)_3_][MsNMs] is 25% higher at 30 °C). Hence, factors which affect the glass temperature are critical when designing better non-fluorinated electrolytes which can compete with existing fluorinated systems.

The Angell plot is also valuable when comparing the fluorinated HCEs to each other. For example, the conductivity of [Li(SL)_3_][TfNCN] (0.461 mS cm^−1^) is 1.7 times higher than that of [Li(SL)_2_][TfNCN] (0.172 mS cm^−1^) at 30 °C. To account for the indirect effect of dilution on the glass transition, we also measured the electrical conductivity of [Li(SL)_3_][TfNCN] at 11.3 °C, and obtained a value of 0.199 mS cm^−1^. At this temperature, the ratio *T*_G_/*T* is the same as for [Li(SL)_2_][TfNCN] at 30 °C, and the difference in conductivity becomes much smaller.

More broadly, when comparing the different samples shown in the Angell plot, it is clear that with this ‘correction’ the curves for [Li(SL)_2_][TfNTf], [Li(SL)_2_][TfNMs], and [Li(SL)_3_][TfNCN] essentially coincide. For these samples, we recorded DMTA spectra (dynamic mechanical thermal analysis, see ESI† Section 4 for details) to gain more insight into the structural relaxation related to viscous flow. In addition, we performed DMTA experiments also on [Li(SL)_2_][TfNFs] as an ‘asymmetric’ fully fluorinated anion, on the HCE [Li(SL)_2_][TfNCN], and on neat sulfolane.

The DMTA results are summarised in Fig. 8b, details can be found in the ESI,† Section 4. At low temperature, all samples display a narrow peak (between −90 and −40 °C) due to the glass transition. Besides this peak, a distinct thermally activated peak was observed only for [Li(SL)_2_][TfNCN], [Li(SL)_3_][TfNCN], and [Li(SL)_2_][TfNTf]. For [Li(SL)_2_][TfNFs] a relaxation also occurs but too close to the glass transition to be clearly detected, while for [Li(SL)_2_][TfNMs] a broad, low intensity relaxation is discernible against the background. [Li(SL)_2_][TfNTf] showed a broad relaxation, which could be fitted with a two-site hopping model (without energy difference between the two sites) and using a Vogel–Fulcher–Tammann dependence for the relaxation time. The obtained activation energy and Vogel–Fulcher–Tammann parameters are similar to those reported from diffusion data in the literature.^67^ The relaxation time parameter *τ*_0_ is in the order of 10 ns, which indicates large relaxing units or diffusive motion. These observations are consistent with strong coupling between cation, anion, and sulfolane. Critically, despite the effect on macroscopic properties in general, the influence of conformational flexibility of the anion could not be detected with this analysis. The reason for this is likely the simultaneous presence of conformational flexibility with large scale relaxing units, in line with the observed broad thermally activated peak.

The two HCEs with the [TfNCN]^−^ anion, [Li(SL)_2_][TfNCN] and [Li(SL)_3_][TfNCN], showed similar behaviour to [Li(SL)_2_][TfNTf], but with more well defined thermally activated peaks. However, for [Li(SL)_3_][TfNCN], the thermally activated peak is shifted towards lower temperature, in agreement with the conclusions drawn from the Angell plot. For both samples, the relaxation peak was analysed with the same model used for [Li(SL)_2_][TfNTf], and the obtained parameters further confirmed that the mechanism giving rise to the peak involves large relaxing units or is the signature of diffusive motion.

Neat sulfolane shows no thermally activated peak due to the high melting point, which we confirmed experimentally. Similarly, for [Li(SL)_2_][TfNMs] and especially [Li(SL)_2_][TfNFs], no thermally activated structural relaxation process was clearly resolved in the spectrum. For [Li(SL)_2_][TfNFs], only a shoulder is observed near the glass transition. This behaviour is consistent with a lower activation energy in this system and, thus, the lower symmetry of the structure and negligible activation volume for conformational reorganisation of the fluorosulfonyl group. In the case of [Li(SL)_2_][TfNMs], the behaviour is more complex, with several apparent overlapping relaxation processes.

Conductivity and viscosity are collective macroscopic properties. In order to gain insight into the relative (diffusive) mobility of specific ions, we also measured diffusion coefficients *via* pulsed field gradient stimulated echo (PFGSTE) NMR diffusometry, Table 2. Here, we do not observe significant correlation between the NMR derived quantities 
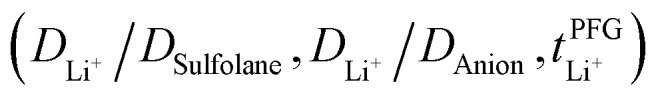
 and 
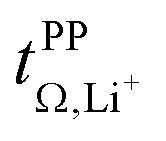
. Especially 
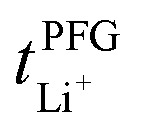
 is often used due to its simplicity, but it is known to be a poor descriptor of lithium transference due to the presence of correlated ion motion.^4,11,26,29,32,64,68,69^

**Table 2:** Self-diffusion coefficients and derived quantities from the PFGSTE measurements. The values where an experimental error is given were obtained from quadruple measurements from independent samples in two separate research groups

Sample name	Self-diffusion *D*/10^−12^ m^2^ s^−1^			Derived quantities		
	Sulfolane	Lithium	Anion	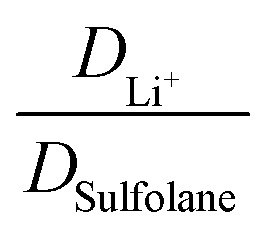	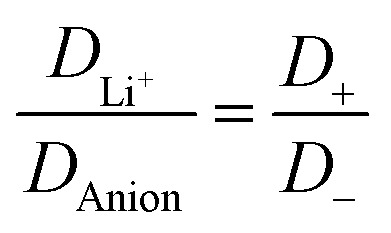	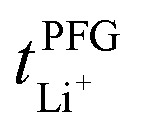
[Li(SL)_2_][TfNCN]	1.64(2)	1.71(8)	1.03(3)	1.04(5)	1.65(9)	0.62(1)
[Li(SL)_3_][TfNCN]	8.51	5.79	4.23	0.68	1.37	0.58
[Li(SL)_2_][TfNMs]	1.15(1)	1.30(3)	0.71(1)	1.14(3)	1.84(5)	0.65(1)
[Li(SL)_2_][TfNFs]	4.98	6.84	4.46	1.37	1.53	0.61
[Li(SL)_2_][TfNTf]^13^	2.9	3.5	2.2	1.21	1.59	0.61
[Li(SL)_2_][PfNFs]	3.91	4.71	3.64	1.20	1.29	0.56
[Li(SL)_3_][TfCHTf]	6.61	4.75	3.46	0.72	1.37	0.58
[Li(SL)_2_][TfNAc]	4.23(14)	1.80(19)	1.59(2)	0.42(5)	1.13(12)	0.53(3)

The electrolyte [Li(SL)_2_][TfNAc] stands out as having the lowest lithium diffusivity relative to both solvent and anion, and consequently also the lowest 
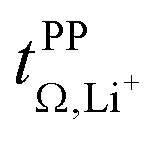
. The diffusion coefficients of cation and anion in [Li(SL)_2_][TfNAc] are virtually identical within experimental error. For comparison, for the [Li(SL)_*X*_][TfNTf] system, *D*_Li^+^_ = *D*_Anion_ is reached only for a salt : solvent ratio of 1 : 8.^13^ [Li(SL)_2_][TfNAc] also exhibited one of the lowest conductivities with 0.101 mS cm^−1^. In contrast, the viscosity was relatively low as well, comparable to [Li(SL)_2_][TfNTf], *cf*. Table 1 and Fig. 8a. The low conductivity and lithium mobility despite the low viscosity suggests that ion aggregation – and thus a lower degree of dissociation – plays a role in this case.

Table 2 reveals an important difference between [Li(SL)_2_][TfNCN] and [Li(SL)_3_][TfNCN]. On one hand, in [Li(SL)_3_][TfNCN], the lithium cation is significantly less diffusive than sulfolane, in fact the sulfolane diffusivity in this sample is the highest of those shown in Table 2. This indicates a significant amount of free sulfolane in the case of [Li(SL)_3_][TfNCN] and a decoupling of ion and solvent motion. Interestingly, on the other hand, the *D*_Li^+^_/*D*_Anion_ ratio is much lower than in the more concentrated [Li(SL)_2_][TfNCN]. Hence, there appears to be a unique transport mechanism for lithium cations which becomes relevant when transitioning from the medium concentrated electrolyte [Li(SL)_3_][TfNCN] to the HCE [Li(SL)_2_][TfNCN] (and [Li(SL)_1_][TfNCN]), explaining the unusually high transference number in the HCEs. We investigated this unique transport mechanism by means of Molecular Dynamics simulation.

An interesting aspect of HCEs is the high electrode potential of lithium metal in the presence of such electrolytes, which has been rationalised with the decreasing activity of the solvent since almost all solvent molecules are involved in lithium ion coordination.^70,71^ Here, we observed that the electrode potential (at comparable salt concentration) increased in the order [Li(SL)_2_][TfNAc] < [Li(SL)_2_][TfNMs] < [Li(SL)_2_][TfNCN]. This trend is in excellent agreement with the HCE character of [Li(SL)_2_][TfNCN] observed in the other physicochemical measurements. Similarly, the relatively low electrode potential of lithium metal in [Li(SL)_2_][TfNAc] further supports a significant degree of association as postulated above.

### Ion correlations

**Fig. 9:**
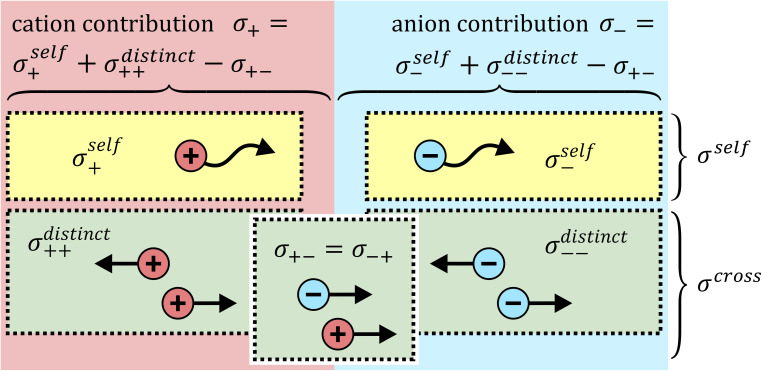
Schematic depiction of different contributions to the ionic conductivity.

The total ionic conductivity *σ* can be calculated from the displacement vectors 
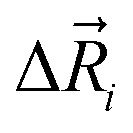
 of the charge carriers in three dimensions, which in turn can be used to define a charge diffusion coefficient *D*_*σ*_, eqn (1).^19,72^1
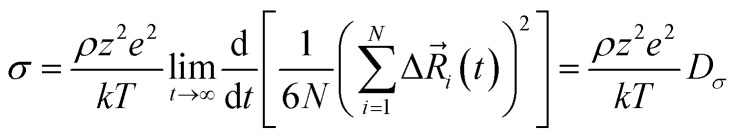
Here, *ρ* is the number density of ions, *z* the charge, *e* the elementary charge, *k* the Boltzmann constant, *T* the thermodynamic temperature, *N* the number of ions. The total ionic conductivity *σ* can then be separated into cation contributions *σ*_+_ and anion contributions *σ*_−_, which in turn can be split into self terms (*σ*^self^_+_ and *σ*^self^_−_) and cross terms (*σ*^distinct^_++_, *σ*^distinct^_−−_, and *σ*_+−_), Fig. 9. We follow the definitions used by Vargas-Barbosa and Roling for the latter five terms.^19^ The corresponding diffusion coefficients can be defined for each term *via* Einstein relations similar to the one in eqn (1) or *via* the corresponding Green–Kubo relations. A variety of equivalent approaches exist, and the reader is referred to the literature for more details.^19,65,72,73^

It is important to note that Fig. 9 is oversimplified. In reality, cross correlations can arise from attractive interactions, leading to correlated motion of constituents, and an often dominant contribution is given by the limitation of density fluctuations in an incompressible liquid. The latter gives rise to strong anticorrelations, as the volume flux of one species has to be compensated by an opposite flux of other species.^74^ Thus, for example, a positive *σ*_+−_ should not be interpreted as signature of ion pairing without further scrutiny.

The cross correlation terms are also often referred to as Onsager coefficients in the literature. They can be obtained experimentally as described in the literature and in the ESI,† Section 13 and have been used successfully to understand ion–ion dynamic correlations.^10,19^ However, the Onsager coefficients as presented here are only valid for a three constituent – two component system.§§The three constituents are the cation, the anion, and the solvent species. Cation and anion cannot separate macroscopically due to electroneutrality restrictions, thus there are only two components, the salt and the solvent. These two components are the only ones which can form a concentration gradient in the simple HCEs in this work. Other cases should be treated with caution, such as mixtures of solvents, mixtures of salts, or when other additives are present.^30,75^

Based on the physicochemical characterisation, we chose the three HCEs [Li(SL)_2_][TfNCN], [Li(SL)_2_][TfNAc], and [Li(SL)_2_][TfNMs] for a more thorough investigation of ion correlations by means of Onsager coefficients (described in detail in the ESI Section 13†). First, the ‘classic’ approach was employed to derive Onsager coefficients from electrochemical measurements, *i.e.*, *D*_salt_, d Δ*φ*/d ln *c*, and 
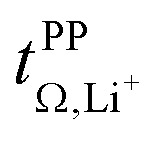
. Second, by measuring the electrophoretic mobility *via* eNMR, the coefficients were directly obtained. In both cases, diffusion coefficients are required as well.

**Fig. 10:**
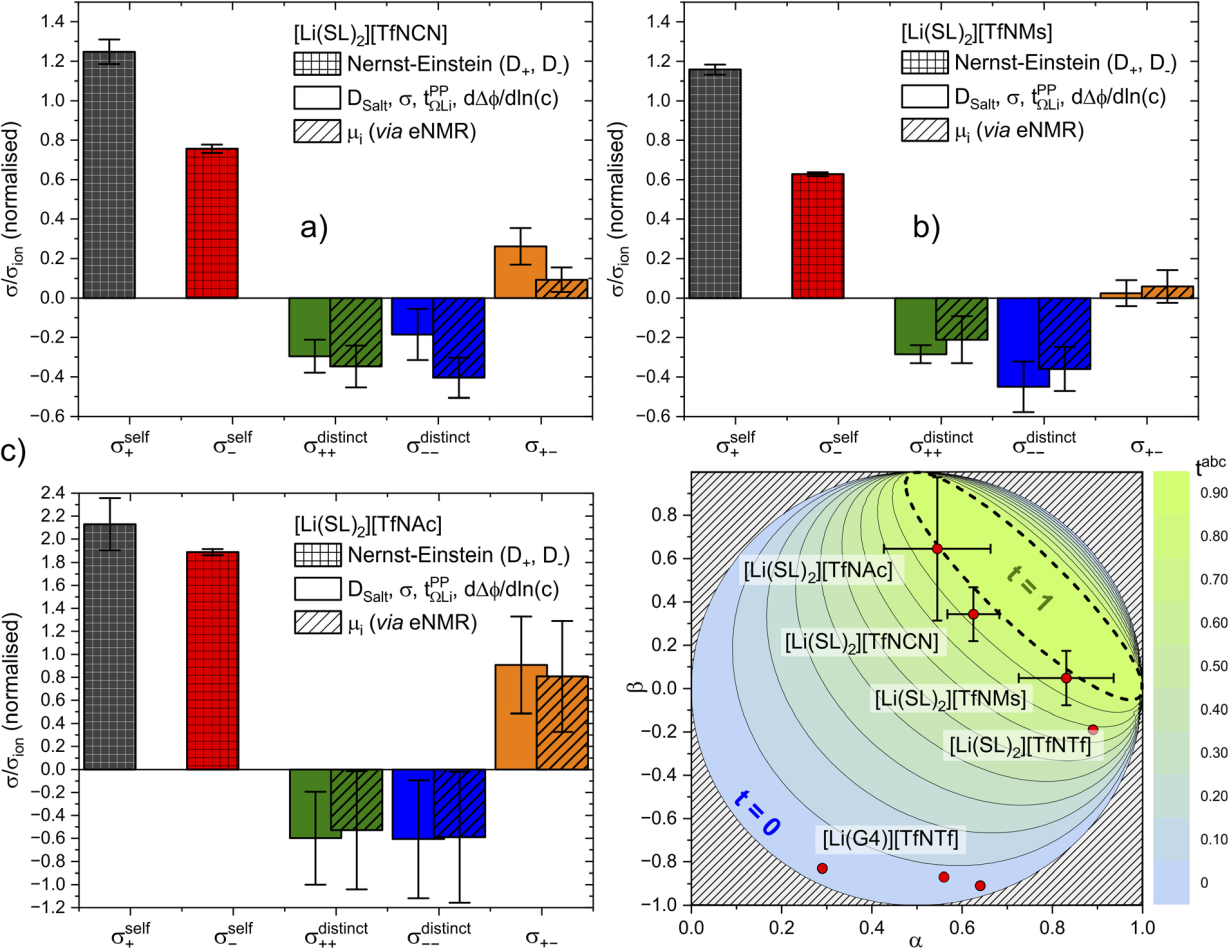
Onsager coefficients for (a) [Li(SL)_2_][TfNCN], (b) [Li(SL)_2_][TfNMs], and (c) [Li(SL)_2_][TfNAc]. (d) correlation between 
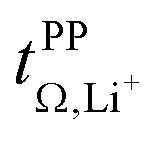
, *α*, and *β*. The values for [Li(SL)_2_][TfNTf] and [Li(G4)][TfNTf] (three points) were taken from the literature.^10,11,32^

The results are presented in Fig. 10. All contributions are normalised by the total ionic conductivity. In this work, we focused on obtaining reliable data including realistic error bars considering propagation of uncertainty from all relevant experimental sources. Importantly, the (distinct) Onsager coefficients we obtained with the two approaches are identical within experimental error. The large uncertainty in the values of [Li(SL)_2_][TfNAc] likely arises from inconsistencies across our independently synthesised samples, since issues with the measurements themselves would not yield such excellent agreement between the two methods. This observation shows the importance of repeated measurements involving samples synthesised independently from different batches of starting materials.

The self-contribution of the lithium cation *σ*^self^_+_ is larger than the total ionic conductivity, *i.e. σ*^self^_+_/*σ*_ion_ > 1, in all cases. This high mobility of Li^+^ is desirable for battery applications. The overall low ionic conductivity in spite of the high lithium mobility arises from the cross-contributions, with all of the distinct terms reducing the overall ionic conductivity. The balance between self and cross contributions is characteristic of HCEs and explains the general trend of high lithium transference number and low conductivity (*cf.*Fig. 7).

The large normalised Onsager coefficients for [Li(SL)_2_][TfNAc] (observe the different scale of Fig. 10c) have to be considered in view of the low ionic conductivity and need to be interpreted with the low *D*_Li^+^_/*D*_Sulfolane_ and *D*_Li^+^_/*D*_Anion_ in mind. However, the normalised Onsager coefficients we observed for [Li(SL)_2_][TfNAc] are still small compared to the highly associated [Li(G3)][TFA], for which *σ*_+−_/*σ*_ion_ ≈ 69 has been reported.^42^

It is important to not overinterpret the ‘negative’ impact of *σ*^cross^, since even *σ*^self^ = *σ*^self^_+_ + *σ*^self^_−_ is relatively small, between 0.234(5) mS cm^−1^ for [Li(SL)_2_][TfNMs] and 0.40(2) mS cm^−1^ for [Li(SL)_2_][TfNAc]. For comparison, the conductivity of 1 M Li[PF_6_] in ethylene carbonate/propylene carbonate as a conventional electrolyte is 13 mS cm^−1^.^8^ Hence, regarding the balance of ion correlations in HCEs, the beneficial impact on transference is much more significant than the detrimental impact on conductivity. In order to further improve HCEs in the future, it is thus reasonable to focus on ways of increasing the dynamics. For example, by engineering electrolytes with lower viscosity and/or glass transition temperatures.

The complex balance of ion correlations can be studied using the parameters *α* and *β* as defined in eqn (2) and (3), respectively.^4^ The lithium transference number depends on the values of these two parameters, which makes them a valuable tool for understanding highly correlated systems such as HCEs.^4,11,42,76^ The relation between lithium transference, *α*, and *β* is visualised with the contour plot in Fig. 10d.2
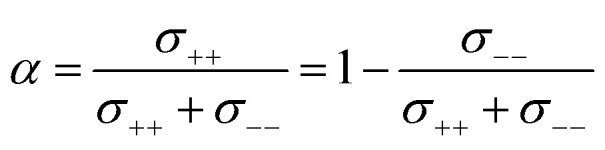
3
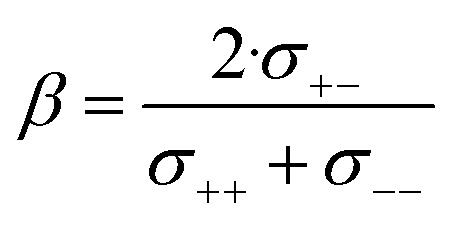


Each two-component electrolyte can be represented by a point in Fig. 10d. The green area enclosed with a dashed line in the top right corresponds to combinations of *α* and *β* which lead to the desired high values of lithium transference near unity. In contrast, systems with low lithium transference – which thus suffer from concentration polarisation – are found in the blue area. The error bars in this representation are large, which explains the considerable spread of the literature values of [Li(G4)][TfNTf]. Thus, it is critical for the discussion of such data to include an assessment of uncertainty. The two methods used here again agree well within experimental error, see the *α*, *β* pairs obtained *via* eNMR in the ESI Section 12† (not shown in Fig. 10d for clarity).

Two limiting cases are interesting when discussing Fig. 10d. Generally, in a binary ionic liquid in the barycentric reference frame, all cation–anion motion must be anticorrelated.^20^ The same holds for a volume-based reference frame, which is valid for incompressible electrolytes in a sample cell.^74^ On one hand, due to the low volume of the lithium cation compared to the anions in this work, the expected behaviour when neglecting Li volume and interaction with anions in the absence of solvent would be *α* ≈ 1 and *β* ≈ 0.^77,78^ On the other hand, electrolytes with strongly associated cations and anions should approach *α* ≈ 0.5 and *β* ≈ 1.^42^ The three HCEs studied in this work cover the range between those two limiting cases, with the *β* parameter increasing in the order [Li(SL)_2_][TfNMs] < [Li(SL)_2_][TfNCN] < [Li(SL)_2_][TfNAc]. In the same order, alpha decreases. Thus, with the systems shown in Fig. 10d, a wide spectrum of more or less associated HCEs covering the *α*, *β* space are now available.

The trends of *α* and *β* mentioned in the previous paragraph can be rationalised in terms of ion association. In general, anion fluorination is correlated with less associated salts. The interpretation of the *β* parameter as a measure of “ion association” would thus explain the positive *β* values in this work, since we used anions with reduced fluorine content. Similarly, the negative cation–anion correlations reported in the literature were observed for [TfNTf]^−^ based HCEs with a higher fluorine content and thus reduced ion association.^11^ Importantly, this direct comparison only works since the anions have an otherwise very similar structure, *e.g.* comparing [Li(SL)_2_][TfNTf] and [Li(SL)_2_][TfNMs] shows the expected influence of anion fluorination. If the solvent or anion structure is changed then this direct comparison is no longer meaningful. For example, very strongly associated ions have been reported for [Li(G3)][TFA], despite the fully fluorinated anion (*cf.*Fig. 4). Similarly, [Li(SL)_2_][TfNAc] is very similar to [Li(SL)_2_][TfNMs], with one sulfonyl group formally replaced by carbonyl, leading again to a slight increase in ion association despite having the same degree of fluorination.

Importantly, there have been studies pointing out potential issues of the potentiostatic polarisation method with strongly associated systems.^79^ However, these studies are based on the assumption that ‘association’ takes place in form of ion pairing, *i.e.* an equilibrium Li^+^ + X^−^ ⇋ LiX. The validity of this assumption is questionable in the case of an HCE, where virtually every ion is interacting closely with more than one counterion. This problem is resembling the discussion of ‘ion pairs’ in the ionic liquids community, which has been largely disproven and replaced by more general terms like ion association or clustering.^80–84^

The transference numbers from eNMR 
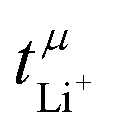
 and the potentiostatic polarisation experiment 
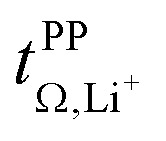
 lie in a similar range for [Li(SL)_2_][TfNCN] and [Li(SL)_2_][TfNMs], see Table 3. We note here that, due to their different definitions and measurement in different experimental conditions, these two numbers do not generally agree, in particular they depend on *σ*_+−_ in a different way, see ESI† section 13. For [Li(SL)_2_][TfNAc], the transference number from eNMR is lower, which is the result of a large positive value of *σ*_+−_. In contrast, for [Li(G4)][TfNTf], Pfeifer *et al.* observed much higher transference numbers from eNMR (
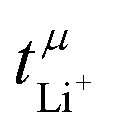
 = 0.58(11)) compared to those measured under anion blocking conditions 
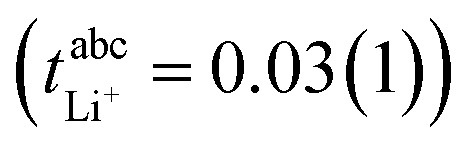
.^32^ In all four systems, *σ*_++_ and *σ*_−−_ are positive, and the apparent discrepancies can be resolved considering *σ*_+−_, eqn (4). In the case of [Li(G4)][TfNTf], *σ*_+−_ < 0, hence 
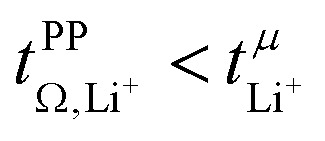
. For the more associated [Li(SL)_2_][TfNAc], *σ*_+−_ becomes positive with *σ*_+−_ < *σ*_−−_, hence 
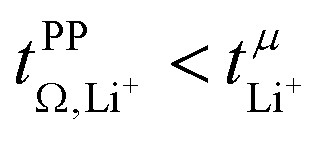
. This is also the case for [Li(SL)_2_][TfNCN] and [Li(SL)_2_][TfNMs], however *σ*_+−_ is considerably smaller and hence 
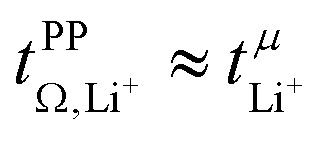
.4
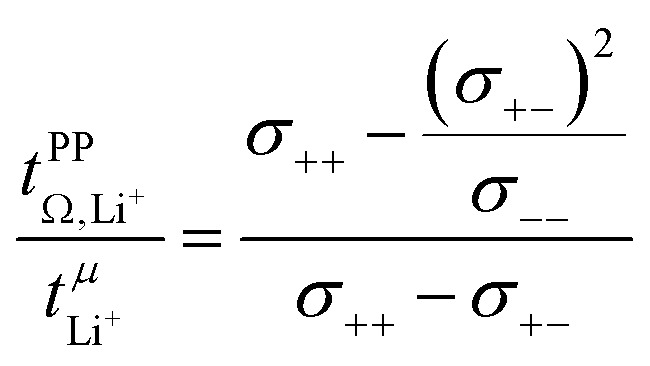


**Table 3:** Comparison of 
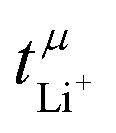
 and 
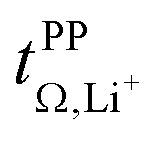

	[Li(SL)_2_][TfNCN]	[Li(SL)_2_][TfNMs]	[Li(SL)_2_][TfNAc]
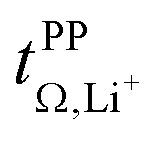	0.83(2)	0.87(3)	0.89(2)
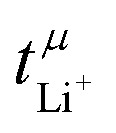 a	0.69(6)	0.85(5)	0.63(25)
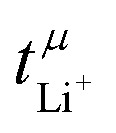 b	0.77(5)	0.82(5)	0.64(11)

a Predicted from 
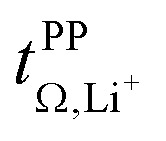
.

b Measured *via* eNMR.

Table 3 also shows the transference number 
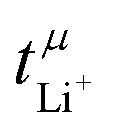
 calculated with the Onsager coefficients from 
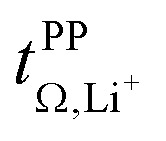
. Thus, if eqn (4) is accounted for, the transference numbers agree within experimental error. This excellent agreement across various methods is reassuring, especially since the Bruce–Vincent approach was originally developed for polymer systems with various strict assumptions such as the absence of convection.

Importantly, very high 
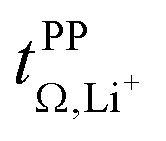
 are often observed for highly associated salts under anion blocking conditions. However, in these cases, the high cation transference might be misleading due to the significant contribution from the diffusion of neutral species. Hence, studies of highly associated salts such as [Li(G3)][TFA] strongly benefit from the use of complementary methods such as eNMR, as already shown in some studies of glyme systems.^32,35^

### Specific interactions in TfNCN based electrolytes

The results from our screening, the previously reported interesting properties, and the promising 
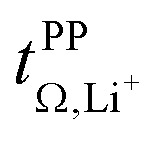
 warrant a detailed investigation of the interactions in [Li(SL)_2_][TfNCN].^56,57^ Thus, we have performed fully atomistic polarisable MD simulations, which generally yield reliable information about coordination environments.

**Fig. 11:**
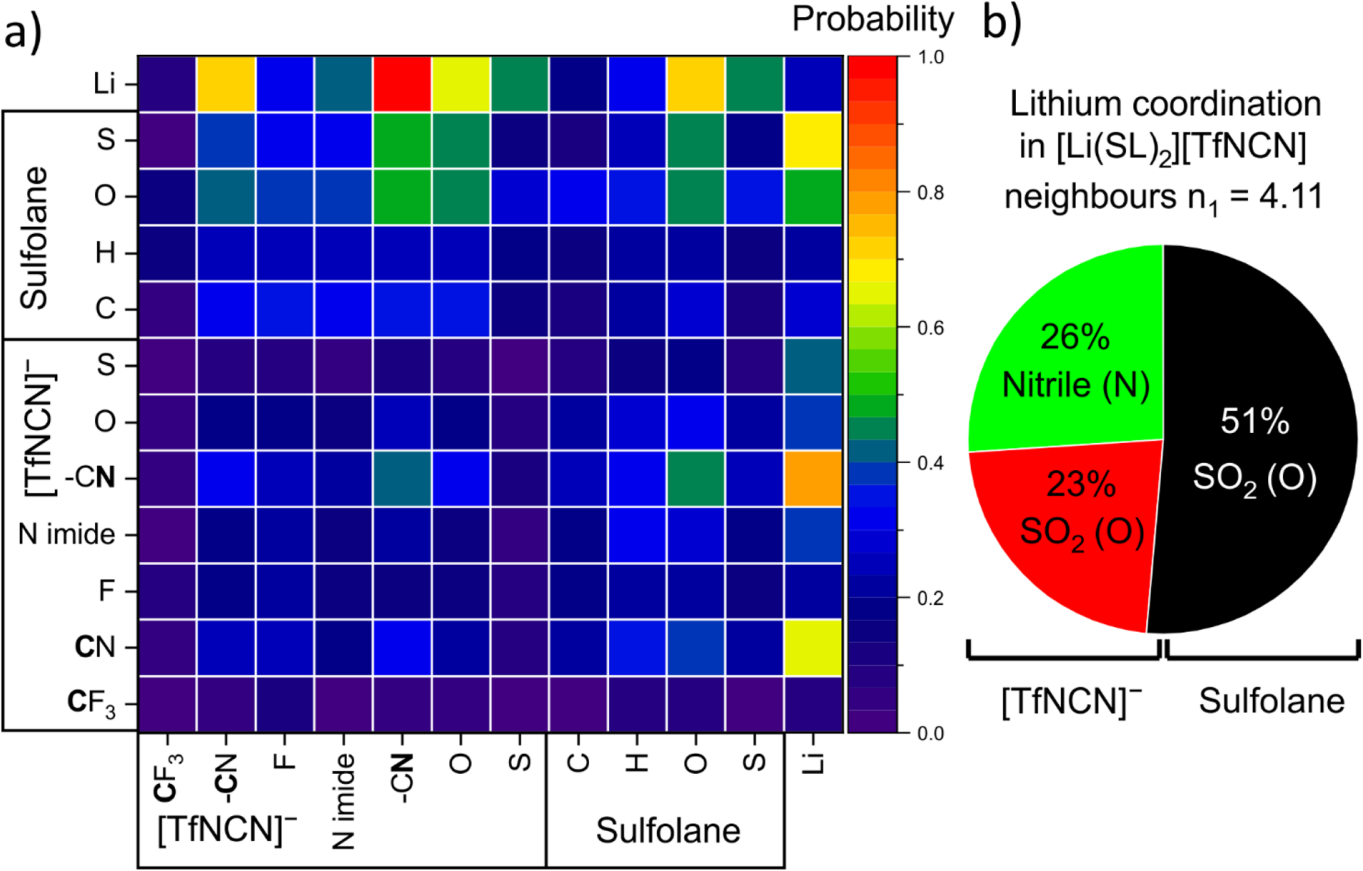
(a) Voronoi neighbourhood probability matrix. Rows are reference atoms; the columns are the neighbours. (b) Relative contributions to the first coordination shell of lithium atoms obtained from the number integral at the position of the first minimum in the atom–atom radial distribution functions.

There are 14 unique atomic sites in [Li(SL)_2_][TfNCN], hence the liquid structure of this HCE is defined by 104 pair distribution functions. The radical Voronoi tessellation implemented in the Travis software package offers a simple way of identifying those pair interactions which are relevant.^85^ The resulting intermolecular neighbourhood matrix is shown in Fig. 11a. The matrix is asymmetric, since for example a lithium atom can have four sulfolane oxygen atoms as neighbours, while it is improbable for a sulfolane oxygen atom to have four lithium atoms as neighbours.

Fig. 11a reveals a high probability for a given lithium atom to be coordinated by a nitrile group, followed by the oxygen atoms of the SO_2_ groups in both the solvent and the anion.¶¶The apparent quantitative discrepancy between the two approaches in Fig. 11 stems from the different normalisation. The probability of finding a nitrile nitrogen atom in a specific neighbour location out of four neighbours is 
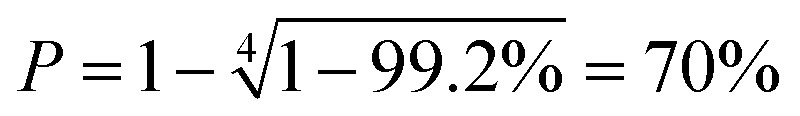
 for the Voronoi analysis and *P* = 1 − (1 – 26.0%)^4^ = 70% for the radial distribution function (more correctly, the number integral). Hence the methods agree very well. A more detailed analysis based on the corresponding radial distribution functions and number integrals revealed the contributions of these atoms to the lithium coordination environment shown in Fig. 11b. Hence, the average tendency to coordinate Li^+^ of one nitrile group is identical to that of one SO_2_ group. Thus, effectively sulfolane and [TfNCN]^−^ each contribute half to the solvation of lithium cations, despite the higher concentration of sulfolane.

**Fig. 12:**
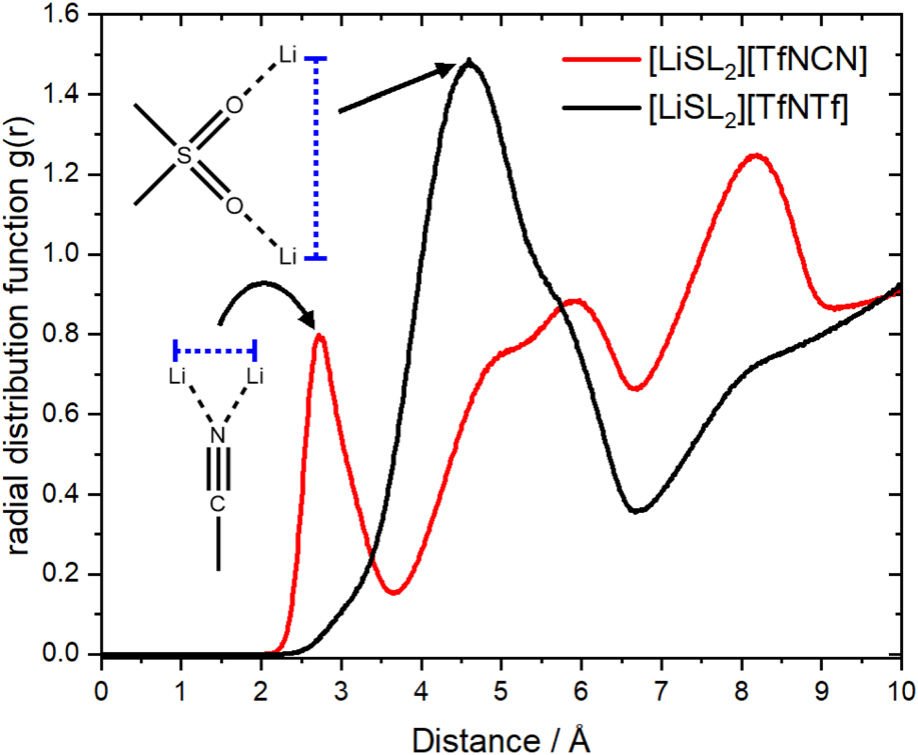
The radial distribution function of lithium – lithium atoms for [Li(SL)_2_][TfNCN] and [Li(SL)_2_][TfNTf].

It has been suggested that, for HCEs, every ion interacts with multiple counterions. A simple statistical analysis shows that ≈70% of lithium cations are indeed coordinated by two anions or more. Conversely, there are also anions which coordinate more than one lithium cation. The latter case is visible as close contacts in the Li^+^⋯Li^+^ radial distribution function, Fig. 12. The peak at 4.6 Å corresponds to a single sulfonyl group coordinating two lithium cations. The sulfonyl group can be part of the sulfolane or the anion, hence this peak is very pronounced for [Li(SL)_2_][TfNTf]. In contrast, the nitrile group in [Li(SL)_2_][TfNCN] is capable of bridging two lithium cations, leading to a peak at 2.7 Å.

**Fig. 13:**
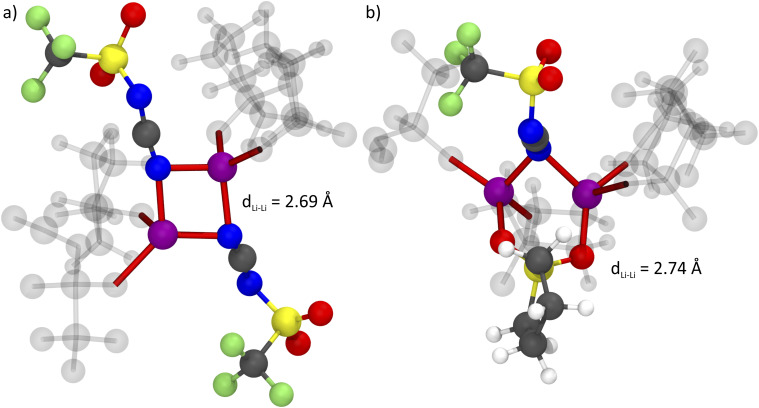
Bridging coordination patterns found in the MD simulation of [Li(SL)_2_][TfNCN], (a) involving two anions and (b) involving one anion and one sulfolane molecule. Lithium atoms are shown in purple. For each lithium atom, the four close contacts to the coordination partners are highlighted in red. Coordinating molecules not participating in bridging coordination are shown as ghosts. The colour code for the other atoms is blue – nitrogen, grey – carbon, yellow – sulphur, red – oxygen, green – fluorine.

The formation of anion-induced Li^+^⋯Li^+^ clusters in [Li(SL)_2_][TfNCN] is consistent with complementary analyses of the trajectory, *cf.* ESI Section 17.† The interesting solvation environment in these clusters can be seen in Fig. 13. The clusters were randomly selected from the middle of the trajectory using the maximum of the first peak in Fig. 12 as distance criterion. The presence of lithium sites in such close proximity suggests that lithium hopping might be enhanced in the vicinity of the nitrile group. We thus suggest that the formation of such clusters might contribute to the high lithium transference number in [Li(SL)_2_][TfNCN].

A similar study on [Li(G4)_*X*_][TfNTf] revealed three concentration regimes, with the HCE regime starting at a molality of ≈*m* > 4.5 mol kg^−1^.^86^ Beyond this threshold, the authors observed the formation of (negatively charged) anion rich clusters.^86^ For comparison, the molality of [Li(SL)_2_][TfNCN] is slightly lower than this threshold (*m* ≈ 4.2 mol kg^−1^). Nevertheless, we observed pronounced clustering and the characteristic behaviour of HCEs. Critically, the clustering in [Li(SL)_2_][TfNCN] appears to be cation-rich, which is preferable to facilitate lithium ion transport. Similar Li^+^ clusters have been reported in a very recent study on a Water-in-Salt electrolyte, in which water molecules were bridging the lithium cations rather than the anion.^87^ An interesting system to explore would thus be a Water-in-Salt electrolyte containing the [TfNCN]^−^ anion to further enhance Li^+^ cluster formation.

## What should the next generation look like?

**Fig. 14:**
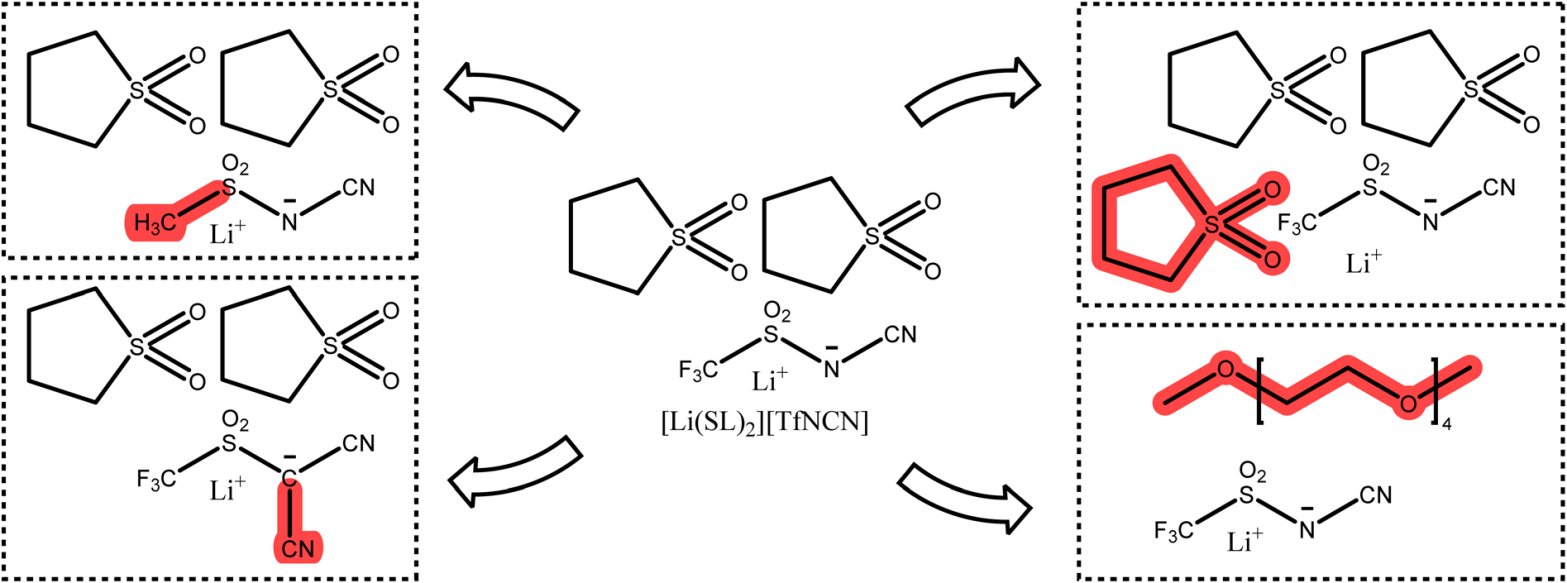
Potential targeted modifications of [Li(SL)_2_][TfNCN].

The optimisation of lithium battery electrolytes is far from being over, especially considering environmental aspects. Based on our results, we briefly discuss interesting targeted modifications to be considered for future studies. Starting from [Li(SL)_2_][TfNCN], systematic changes can be made to the anion or the solvent, Fig. 14.

One of the most interesting variations of [TfNCN]^−^ is the methanide anion [TfC(CN)_2_]^−^, which was the most dissociative anion based on the gas phase stabilisation energy, with a surface electrostatic potential distribution similar to the successful [TfNTf]^−^ anion. At the same time, the two nitrile groups in close proximity might be beneficial in promoting lithium clustering or lithium hopping along extended chain networks.

The [TfNCN]^−^ anion is likely persistent in the environment due to the presence of the CF_3_ group. An alternative at least on paper would be [FsNCN]^−^, at the cost of a more challenging synthesis and unknown stability. Such seemingly minor changes can have a major impact on other properties such as cycling stability and need to be considered in the bigger picture.^88^

A similar alternative to [TfNCN]^−^ would be completely non-fluorinated anions such as [MsNCN]^−^ or [MsC(CN)_2_]^−^. A wide variety of *e.g.* (RSO_2_)_*X*_(CN)_3−*X*_CH acids are known already from different context.^89^ Non-fluorinated imide-based anions gave promising results but also showed that the solvents likely need to be optimised too, as the commonly used solvents were naturally optimised for fully fluorinated anions.^90^ For example, the nitrile functionalised (fluorinated and non-fluorinated) imidazolate anions previously investigated by Scheers *et al.* could be revisited to study their suitability for use in HCEs.^91^

Considering the solvent, the most straightforward parameter is the salt : solvent ratio. Within the HCE regime, this ratio can be changed to optimise the trade-off between lithium transference and conductivity. The environmental aspects of course also apply to the solvent, with a recent trend towards fluorine free solvents.^36^ New approaches in this respect are for example the use of siloxane-based^37^ or Water-in-Salt^92^ electrolytes.

The most important factor to consider is the balance of solvent and anion in terms of their interaction with lithium cations. In this work, we explored this balance from the viewpoint of the anion (keeping the same solvent). Zhou *et al.* recently published similar findings from the viewpoint of the solvent (keeping the same anion) using a very different methodology. In line with this, previous studies have found that coordination environments can change drastically when only the anion, solvent polarity, or solvent content is changed in a given system without adjusting the other components.^52,86,93^ Thus, salts such as Li[MsNCN], Li[MsC(CN)_2_], or even Li[MsNMs] might be useable, but will require changes to the solvent as well, even if just for solubility reasons.

## Conclusions

We screened a large number of anions that are potentially suitable for lithium battery electrolytes with high lithium transference number. A variety of different theoretical and experimental approaches was employed to appropriately cover the complex interactions commonly found in HCEs. The anions were compared in terms of stabilisation energy, specific interactions with Li^+^, degree of fluorination, flexibility, viscous flow near the glass transition, structural relaxation, ion aggregation, HCE/LCE behaviour, dynamic ion correlations, statistical liquid structure and cluster formation. Among the many investigated electrolytes with high lithium transference number, we selected [Li(SL)_2_][TfNMs], [Li(SL)_2_][TfNCN], and [Li(SL)_2_][TfNAc] for a more detailed study. We discussed the ion association tendency and its implications on macroscopic and microscopic properties. The HCE [Li(SL)_2_][TfNCN] showed unique behaviour which we were able to trace back to a characteristic interaction between the nitrile group and the lithium cation. Specifically, the nitrile group is capable of inducing the formation of cation rich clusters. Sulfolane and [TfNCN]^−^ each contribute half to the solvation of lithium cations, despite the higher concentration of sulfolane. We postulate that, in order to maximise lithium mobility, the coordination tendency of solvent and anion should be matched. Lithium hopping might be promoted by such a balanced choice, since neither of the two coordination sites is preferred.

Onsager coefficients are usually derived from several error prone experiments. Thus, one of our central aims in this work was to provide realistic estimates of experimental uncertainty. The results show that knowledge of uncertainty in the Onsager coefficients (and *α*, *β*) is a point which cannot be ignored. For [Li(SL)_2_][TfNCN], the uncertainty in *D*_Salt_ accounted for roughly 85% of the total uncertainty, a large part of which is due to the assumed uncertainty of 0.01 mm in the separator thickness. The actual deviations from the nominal separator thickness might be even larger due to the pressure in the coin cell, hence methods with well-defined and verifiable electrode separation are preferable, but not always available.

In a nutshell, there is no perfect anion which works well with all solvents (and *vice versa*). The key to success appears to be the choice of the salt in relation to the solvent.

## Data availability

The data related to this article is available as ESI.†

## Author contributions

Frederik Philippi: conceptualization, methodology, software (prealpha software package), validation, formal analysis, investigation, data curation, writing – original draft, writing – review & editing, visualization, supervision, project administration, funding acquisition. Maleen Middendorf: validation, formal analysis (eNMR), investigation (eNMR), writing – original draft, writing – review & editing, visualization. Keisuke Shigenobu: investigation (running MD simulations), writing – original draft, writing – review & editing, visualization. Yuna Matsuyama: investigation (synthesis of HTfNAc/LiTfNAc, measurements of [LiG4][TfNAc], crystallography, TGA measurements), writing – review & editing. Oriele Palumbo: formal analysis (DMTA), investigation (DMTA), writing – original draft, writing – review & editing, visualization. David Pugh: formal analysis (crystallography), writing – review & editing. Taku Sudoh: writing – review & editing, supervision. Kaoru Dokko: resources, funding acquisition. Masayoshi Watanabe: conceptualization, resources, writing – review & editing, funding acquisition. Monika Schönhoff: conceptualization, resources, writing – review & editing, supervision, funding acquisition. Wataru Shinoda: conceptualization, software (MPDynPFF software package), resources, supervision, funding acquisition. Kazuhide Ueno: conceptualization, resources, writing – review & editing, supervision, project administration.

## Conflicts of interest

There are no conflicts to declare.

## Supplementary Material

SC-015-D4SC01492H-s001

SC-015-D4SC01492H-s002

SC-015-D4SC01492H-s003

## References

[cit1] Kim T., Song W., Son D.-Y., Ono L. K., Qi Y. (2019). J. Mater. Chem. A.

[cit2] Li M., Wang C., Davey K., Li J., Li G., Zhang S., Mao J., Guo Z. (2023). SmartMat.

[cit3] Mauger A., Julien C. M. (2017). Ionics.

[cit4] Wohde F., Balabajew M., Roling B. (2016). J. Electrochem. Soc..

[cit5] Yamada Y., Yamada A. (2015). J. Electrochem. Soc..

[cit6] McOwen D. W., Seo D. M., Borodin O., Vatamanu J., Boyle P. D., Henderson W. A. (2014). Energy Environ. Sci..

[cit7] Qian J., Henderson W. A., Xu W., Bhattacharya P., Engelhard M., Borodin O., Zhang J.-G. (2015). Nat. Commun..

[cit8] Ugata Y., Shigenobu K., Tatara R., Ueno K., Watanabe M., Dokko K. (2021). Phys. Chem. Chem. Phys..

[cit9] Giffin G. A. (2022). Nat. Commun..

[cit10] Dong D., Sälzer F., Roling B., Bedrov D. (2018). Phys. Chem. Chem. Phys..

[cit11] Shigenobu K., Dokko K., Watanabe M., Ueno K. (2020). Phys. Chem. Chem. Phys..

[cit12] Dokko K., Watanabe D., Ugata Y., Thomas M. L., Tsuzuki S., Shinoda W., Hashimoto K., Ueno K., Umebayashi Y., Watanabe M. (2018). J. Phys. Chem. B.

[cit13] Nakanishi A., Ueno K., Watanabe D., Ugata Y., Matsumae Y., Liu J., Thomas M. L., Dokko K., Watanabe M. (2019). J. Phys. Chem. C.

[cit14] Ugata Y., Sasagawa S., Tatara R., Ueno K., Watanabe M., Dokko K. (2021). J. Phys. Chem. B.

[cit15] Shimizu K., Watanabe M., Canongia Lopes J. N., de Freitas A. A. (2023). J. Mol. Liq..

[cit16] Della Monica M., Jannelli L., Lamanna U. (1968). J. Phys. Chem..

[cit17] Shigenobu K., Sudoh T., Murai J., Dokko K., Watanabe M., Ueno K. (2023). Chem. Rec..

[cit18] Valøen L. O., Reimers J. N. (2005). J. Electrochem. Soc..

[cit19] Vargas-Barbosa N. M., Roling B. (2020). ChemElectroChem.

[cit20] Kashyap H. K., Annapureddy H. V. R., Raineri F. O., Margulis C. J. (2011). J. Phys. Chem. B.

[cit21] Gouverneur M., Kopp J., van Wüllen L., Schönhoff M. (2015). Phys. Chem. Chem. Phys..

[cit22] Zhang Z., Madsen L. A. (2014). J. Chem. Phys..

[cit23] Holz M. (1994). Chem. Soc. Rev..

[cit24] Johnson C. S., He Q. (1989). Electrophoretic Nuclear Magnetic Resonance.

[cit25] Hallberg F., Furó I., Yushmanov P. V., Stilbs P. (2008). J. Magn. Reson..

[cit26] Gouverneur M., Schmidt F., Schönhoff M. (2018). Phys. Chem. Chem. Phys..

[cit27] Brinkkötter M., Giffin G. A., Moretti A., Jeong S., Passerini S., Schönhoff M. (2018). Chem. Commun..

[cit28] Ackermann F., Schönhoff M. (2021). J. Phys. Chem. C.

[cit29] Bergstrom H. K., Fong K. D., Halat D. M., Karouta C. A., Celik H. C., Reimer J. A., McCloskey B. D. (2023). Chem. Sci..

[cit30] Fang C., Halat D. M., Mistry A., Reimer J. A., Balsara N. P., Wang R. (2023). Chem. Sci..

[cit31] Rosenwinkel M. P., Schönhoff M. (2019). J. Electrochem. Soc..

[cit32] Pfeifer S., Ackermann F., Sälzer F., Schönhoff M., Roling B. (2021). Phys. Chem. Chem. Phys..

[cit33] Yu D., Troya D., Korovich A. G., Bostwick J. E., Colby R. H., Madsen L. A. (2023). ACS Energy Lett..

[cit34] Halat D. M., Mistry A., Hickson D., Srinivasan V., Balsara N. P., Reimer J. A. (2023). J. Electrochem. Soc..

[cit35] Schmidt F., Schönhoff M. (2020). J. Phys. Chem. B.

[cit36] Hernández G., Mogensen R., Younesi R., Mindemark J. (2022). Batteries Supercaps.

[cit37] Huang Y., Li R., Weng S., Zhang H., Zhu C., Lu D., Sun C., Huang X., Deng T., Fan L., Chen L., Wang X., Fan X. (2022). Energy Environ. Sci..

[cit38] Rensmo A., Savvidou E. K., Cousins I. T., Hu X., Schellenberger S., Benskin J. P. (2023). Environ. Sci.: Processes Impacts.

[cit39] Xu K. (2014). Chem. Rev..

[cit40] Philippi F., Welton T. (2021). Phys. Chem. Chem. Phys..

[cit41] Bonhôte P., Dias A.-P., Armand M., Papageorgiou N., Kalyanasundaram K., Grätzel M. (1996). Inorg. Chem..

[cit42] Shigenobu K., Shibata M., Dokko K., Watanabe M., Fujii K., Ueno K. (2021). Phys. Chem. Chem. Phys..

[cit43] Philippi F., Rauber D., Palumbo O., Goloviznina K., McDaniel J., Pugh D., Suarez S., Fraenza C. C., Padua A., Kay C. W. M., Welton T. (2022). Chem. Sci..

[cit44] Zhang H., Oteo U., Zhu H., Judez X., Martinez-Ibañez M., Aldalur I., Sanchez-Diez E., Li C., Carrasco J., Forsyth M., Armand M. (2019). Angew. Chem., Int. Ed..

[cit45] Santiago A., Castillo J., Garbayo I., Saenz de Buruaga A., Coca Clemente J. A., Qiao L., Cid Barreno R., Martinez-Ibañez M., Armand M., Zhang H., Li C. (2021). ACS Appl. Energy Mater..

[cit46] Osborne D. A., Breedon M., Rüther T., Spencer M. J. S. (2022). J. Mater. Chem. A.

[cit47] Scheers J., Jónsson E., Jacobsson P., Johansson P. (2012). Electrochemistry.

[cit48] Tsuzuki S., Shinoda W., Seki S., Umebayashi Y., Yoshida K., Dokko K., Watanabe M. (2013). ChemPhysChem.

[cit49] Tsuzuki S., Shinoda W., Matsugami M., Umebayashi Y., Ueno K., Mandai T., Seki S., Dokko K., Watanabe M. (2015). Phys. Chem. Chem. Phys..

[cit50] Johansson P., Tegenfeldt J., Lindgren J. (1999). Polymer.

[cit51] Saito S., Watanabe H., Hayashi Y., Matsugami M., Tsuzuki S., Seki S., Canongia Lopes J. N., Atkin R., Ueno K., Dokko K., Watanabe M., Kameda Y., Umebayashi Y. (2016). J. Phys. Chem. Lett..

[cit52] Murphy T., Callear S. K., Yepuri N., Shimizu K., Watanabe M., Canongia Lopes J. N., Darwish T., Warr G. G., Atkin R. (2016). Phys. Chem. Chem. Phys..

[cit53] Schkeryantz L., Nguyen P., McCulloch W. D., Moore C. E., Lau K. C., Wu Y. (2022). J. Phys. Chem. C.

[cit54] Lascaud S., Perrier M., Vallee A., Besner S., Prud'homme J., Armand M. (1994). Macromolecules.

[cit55] Lascaud S., Perrier M., Armand M., Prud'homme J., Kapfer B., Vallée A., Gauthier M. (1998). Electrochim. Acta.

[cit56] Nürnberg P., Lozinskaya E. I., Shaplov A. S., Schönhoff M. (2020). J. Phys. Chem. B.

[cit57] Penley D., Wang X., Lee Y.-Y., Garaga M. N., Ghahremani R., Greenbaum S., Maginn E. J., Gurkan B. (2022). J. Chem. Eng. Data.

[cit58] Brinkkötter M., Lozinskaya E. I., Ponkratov D. O., Vlasov P. S., Rosenwinkel M. P., Malyshkina I. A., Vygodskii Y., Shaplov A. S., Schönhoff M. (2017). Electrochim. Acta.

[cit59] Evans J., Vincent C. A., Bruce P. G. (1987). Polymer.

[cit60] Bruce P. G., Evans J., Vincent C. A. (1988). Solid State Ionics.

[cit61] Bruce P. G., Vincent C. A. (1987). J. Electroanal. Chem. Interfacial Electrochem..

[cit62] Watanabe M., Nagano S., Sanui K., Ogata N. (1988). Solid State Ionics.

[cit63] Galluzzo M. D., Maslyn J. A., Shah D. B., Balsara N. P. (2019). J. Chem. Phys..

[cit64] Ho J. S., Borodin O. A., Ding M. S., Ma L., Schroeder M. A., Pastel G. R., Xu K. (2023). Energy Environ. Mater..

[cit65] Xu K. , Electrolytes, Interfaces and Interphases, The Royal Society of Chemistry, 2023

[cit66] Shkrob I. A., Marin T. W., Zhu Y., Abraham D. P. (2014). J. Phys. Chem. C.

[cit67] Tsuzuki S., Ikeda S., Shinoda W., Shigenobu K., Ueno K., Dokko K., Watanabe M. (2023). J. Phys. Chem. B.

[cit68] Shah D. B., Nguyen H. Q., Grundy L. S., Olson K. R., Mecham S. J., DeSimone J. M., Balsara N. P. (2019). Phys. Chem. Chem. Phys..

[cit69] Sudoh T., Shigenobu K., Dokko K., Watanabe M., Ueno K. (2022). Phys. Chem. Chem. Phys..

[cit70] Ueno K., Tatara R., Tsuzuki S., Saito S., Doi H., Yoshida K., Mandai T., Matsugami M., Umebayashi Y., Dokko K., Watanabe M. (2015). Phys. Chem. Chem. Phys..

[cit71] Moon H., Tatara R., Mandai T., Ueno K., Yoshida K., Tachikawa N., Yasuda T., Dokko K., Watanabe M. (2014). J. Phys. Chem. C.

[cit72] Yao N., Chen X., Fu Z.-H., Zhang Q. (2022). Chem. Rev..

[cit73] Allen M. P. and Tildesley D. J., Computer Simulation of Liquids, Oxford University Press, 2nd edn, 2017, vol. 1

[cit74] Lorenz M., Kilchert F., Nürnberg P., Schammer M., Latz A., Horstmann B., Schönhoff M. (2022). J. Phys. Chem. Lett..

[cit75] Kjelstrup S., Gunnarshaug A. F., Gullbrekken Ø., Schnell S. K., Lervik A. (2023). J. Chem. Phys..

[cit76] Ikeda S., Tsuzuki S., Sudoh T., Shigenobu K., Ueno K., Dokko K., Watanabe M., Shinoda W. (2023). J. Phys. Chem. C.

[cit77] Koishi T., Kawase S., Tamaki S. (2002). J. Chem. Phys..

[cit78] Sinistri C. (1962). J. Phys. Chem..

[cit79] Bruce P. G., Hardgrave M. T., Vincent C. A. (1989). J. Electroanal. Chem. Interfacial Electrochem..

[cit80] Welton T. (2018). Biophys. Rev..

[cit81] Zhao W., Leroy F., Heggen B., Zahn S., Kirchner B., Balasubramanian S., Müller-Plathe F. (2009). J. Am. Chem. Soc..

[cit82] Kirchner B., Malberg F., Firaha D. S., Hollóczki O. (2015). J. Phys.: Condens.Matter.

[cit83] Lui M. Y., Crowhurst L., Hallett J. P., Hunt P. A., Niedermeyer H., Welton T. (2011). Chem. Sci..

[cit84] a Lee A., Vella D., Perkin S., Goriely A. (2014). J. Phys. Chem. Lett..

[cit85] Brehm M., Thomas M., Gehrke S., Kirchner B. (2020). J. Chem. Phys..

[cit86] Fang C., Halat D. M., Balsara N. P., Wang R. (2023). J. Phys. Chem. B.

[cit87] Goloviznina K., Serva A., Salanne M. (2024). J. Am. Chem. Soc..

[cit88] Liu J., Kaneko T., Ock J., Kondou S., Ueno K., Dokko K., Sodeyama K., Watanabe M. (2023). J. Phys. Chem. C.

[cit89] Dijkstra R., Backer H. J., des Trav R. (1954). Chim. Pays-Bas..

[cit90] Mandal B., Sooksimuang T., Griffin B., Padhi A., Filler R. (2004). Solid State Ionics.

[cit91] Scheers J., Johansson P., Szczeciński P., Wieczorek W., Armand M., Jacobsson P. (2010). J. Power Sources.

[cit92] Mendez-Morales T., Li Z., Salanne M. (2021). Batteries Supercaps.

[cit93] Ueno K., Murai J., Ikeda K., Tsuzuki S., Tsuchiya M., Tatara R., Mandai T., Umebayashi Y., Dokko K., Watanabe M. (2016). J. Phys. Chem. C.

