# Supplementary material for: Evolving better solvate electrolytes for lithium secondary batteries
Source: Chem Sci. 2024 Apr 11;15(19):7342–58. doi: 10.1039/d4sc01492h (PMC11095511; doi:10.1039/d4sc01492h)
Supplement: SC-015-D4SC01492H-s002 [file SC-015-D4SC01492H-s002.pdf]

Electronic Supplementary Material (ESI) for Chemical Science.
This journal is © The Royal Society of Chemistry 2024

Supporting Information to

Evolving Better Solvate Electrolytes for Lithium Secondary Batteries

Frederik Philippi,*[a] Maleen Middendorf,[b] Keisuke Shigenobu,[c] Yuna Matsuyama,[a] Oriele Palumbo,[d] David Pugh,[e] Taku Sudoh,[a] Kaoru Dokko,[a,f] Masayoshi Watanabe,[f] Monika Schönhoff,[b] Wataru Shinoda[c] and Kazuhide Ueno[a,f]

[a] Department of Chemistry and Life Science, Yokohama National University, 79-5 Tokiwadai, Hodogaya-ku, Yokohama 240-8501, Japan

[b] University of Münster, Corrensstraße 28/30, 48149 Münster, Germany

[c] Research Institute for Interdisciplinary Science, Okayama University, Okayama 700-8530, Japan

[d] Consiglio Nazionale delle Ricerche, Istituto dei Sistemi Complessi, P.le Aldo Moro 5, 00185 Rome, Italy

[e] Department of Chemistry, Britannia House, Kings College London, 7 Trinity Street, London SE1 1DB, UK

[f] Advanced Chemical Energy Research Centre, Advanced Institute of Sciences, Yokohama National University, 79-5 Tokiwadai, Hodogaya-ku, Yokohama 240-8501, Japan

Contents

1. Nomenclature 4
2. Syntheses 5
   - 2.1. Electrolyte precursors 6
     - N-pentyl-trifluoromethanesulfonamide 473 6
     - Lithium N-pentyl-trifluoromethanesulfonamide 474 6
     - N-((Trifluoromethyl)sulfonyl)acetamide 7
     - Lithium acetyl(trifluoromethylsulfonyl)imide 7
     - N-(3-methoxypropyl) trifluoromethanesulfonamide 436+438 8
     - Lithium (3-methoxypropyl)(trifluoromethanesulfonyl)amide 461 8
     - Bis(methanesulfonyl)amide 486 9
     - Lithium bis(methanesulfonyl)amide 506 9
     - 2-(methylsulfonyl)malononitrile 537 10
     - Lithium 2-(methylsulfonyl)malononitrile 539 10
   - 2.2. Infrared spectra 11
3. Thermal Transitions *via* DSC 18
4. DMTA measurements 29
5. Thermal stability *via* TGA 38
6. Density and Viscosity Measurements 40
7. Lithium transference from Symmetric Li-Li Coin Cell 43
8. Ionic Conductivity 47
   - 8.1. Conductivity cell (ex situ) 47
   - 8.2. Coin Cell (in situ) 48
9. Salt diffusion 49
10. Electromotive Force 53
11. PFGSTE NMR Diffusometry 55
12. Electrophoretic NMR 56
13. Onsager coefficients 60
   - 13.1. Method A 61
   - 13.2. Method B 63
14. Error estimates 63
   - 14.1. Method A 64
   - 14.2. Method B 65
15. Electrochemical window 65
16. *Ab initio* calculations 73
   - 16.1. Dihedral Potential Energy Scan 73
   - 16.2. Acidity and Lithium Affinity 74

16.3. Influence of Solvation 80
16.4. Surface charge density analysis 82
16.5. Symmetry adapted perturbation theory 83
17. MD Simulation Details 88
17.1. Force field parameters 88
17.2. Parameterisation of the $[TfNCN]^-$ anion 92
Angle bending 92
Dihedral torsions 92
Stabilisation energy of Li^+–$[TfNCN]^-$ 94
17.3. Dynamic properties 96
17.4. Static properties 98
17.5. Speciation Analysis 102
18. Crystallography 105
19. References 107

1. Nomenclature

Most of the ions in this work are more or less established in the literature, though not always in connection with lithium ion batteries, and are known under several synonyms, *cf.* [TfC(CN)$_2$]$^-$,[1] [MsC(CN)$_2$]$^-$,[2] [TfNCN]$^-$,[1,3–7] [MsNCN]$^-$, [TfN3O1]$^-$,[8–10] [TfN5]$^-$, [TfNMe]$^-$, [TfNTf]$^-$,[11] [MsNMs]$^-$, [12–15] [6cPFSI]$^-$,[14,16,17] [5cPFSI]$^-$,[16,18] [(SO$_2$CN)N(SO$_2$CN)]$^-$,[15,19] [TfNMs]$^-$,[14,20,21] [TfCHTf]$^-$,[14,22–25] [CTf$_3$]$^-$,[24,26,27] [CMs$_3$]$^-$,[28,29] [DFTFSI]$^-$,[21,30–33] [TfNFs]$^-$,[15,34–36] [FsNFs]$^-$,[15,37–39] [PfNFs]$^-$,[15] [PfNPf]$^-$,[6,15] [MsNTFA]$^-$,[40] [TfNAc]$^-$,[40] [TfNTFA]$^-$,[5–7,41] [PO$_2$(CF$_3$)$_2$] $^-$,[42] [PO$_2$F$_2$] $^-$[43]. To avoid ambiguity, the abbreviations used in this work are shown together with common abbreviations in Figure S1.

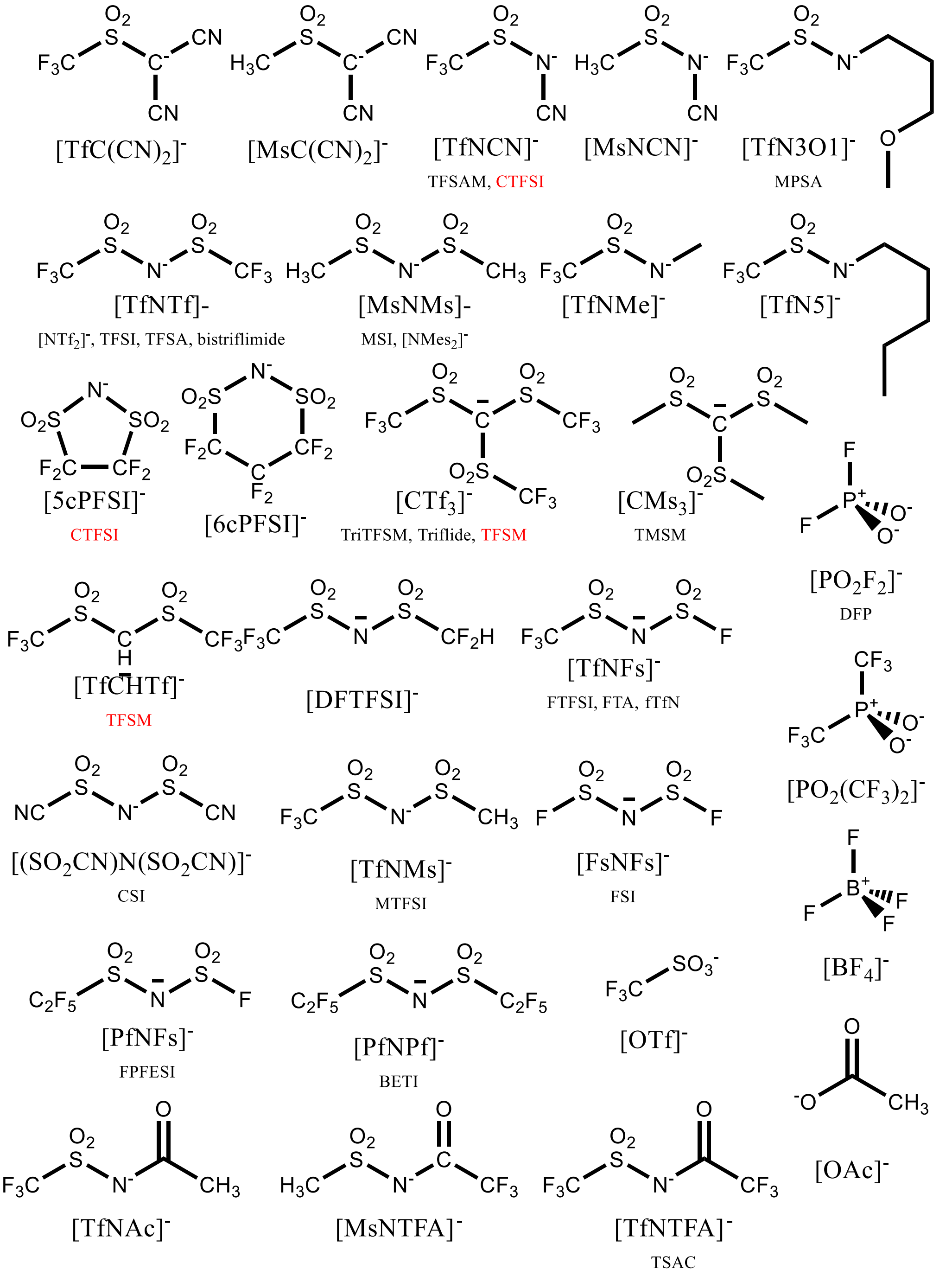

Figure S1: Scheme of anions and abbreviations used in this work. Literature abbreviations shown in smaller font.

2. Syntheses

Sample preparation and handling was performed under anaerobic conditions using standard Schlenk techniques[44] or in a dry chamber with dew point <−70°C (Daikin industries LTD, model HRG-60AR). Lithium salts were pre-dried (40-50°C, 24 h) under dynamic fine vacuum (ca 0.05 mbar, rotary vane pump, GCD-051X, ULVAC, with liquid nitrogen or dry ice cold trap to prevent back diffusion of oil) in a GTO-200 glass tube oven (Shibata scientific technology LTD) fitted with a drying bulb filled with fresh P_4O_{10} (repeatedly replaced with fresh P_4O_{10} if water uptake was visible). The pre dried samples were removed from the glass tube oven in a Glovebox with dew point ≈−100 °C (Miwa Manufacturing Co., Ltd., Model DBO-1KP-YUD03). Drying in high vacuum was achieved using a TURBOLAB Core90i high vacuum pumping station (8×10^{-8} mbar end vacuum, Leybold) equipped with liquid nitrogen cold trap (QF40 flange size, ANCORP). Dried salt samples were handled under argon in a glovebox with ≤0.5 ppm water (VAC vacuum atmospheres company, model OMNI-LAB).

Electrolyte samples were prepared by adding the desired solvent in the desired stoichiometry to the salt, followed by stirring at 40°C until dissolution was complete. Freshly cut lithium foil was then added, and the sample only used for measurements if no reaction (gas evolution, blackening of the foil) could be observed over the course of at least a day. A sample of $[Li(SL)_2]$[TfNCN] was prepared according to this general procedure (without adding lithium foil) and the water content measured *via* Karl Fischer titration to verify the synthesis protocol. The water content thus obtained was 42 ppm.

The reference electrolyte for electrochemical measurements was prepared by dissolving 10.406 g Li[TfNTf] in 30.818 g triethylene glycol dimethyl ether (triglyme) inside a glovebox.

Dry solvents (dichloromethane, toluene, ethyl acetate, acetonitrile, ethanol, methanol, hexane, diethyl ether, dimethyl sulfoxide, acetone, chloroform) were provided by FUJIFILM Wako Chemicals U.S.A. Corporation as super dehydrated grade (<10 ppm water). Commercial chemicals were purified as advised in the literature.[45,46] NMR spectra for compound identification and confirmation of purity were recorded at 25°C on an ECA 500 spectrometer (JEOL).

Commercial samples were Li[TfNCN] 99.2% <100 ppm water content / Li[PfNFs] 99.9% 27 ppm water content (PROVISCO CS, Czech Republic), OTf_2 >98.0% purity / Li[6cPFSI] >98.0% / Triethyl amine >99.0% / 3-methoxypropylamine >99.0% / (Tokyo Chemical Industry Co., Ltd., Tokyo, Japan), Li[TfNFs] 98.0-103.0% / Hydrochloric acid solution 6 M / sulfuric acid >95.0% (FUJIFILM Wako Chemicals U.S.A. Corporation), Lithium hydride ≥95% / Sodium hydroxide solution 4 M (Sigma Aldrich), other battery grade solvents such as sulfolane and salts such as Li[TfNTf] (Kishida Chemical Co., Ltd., Osaka, Japan).

The majority of electrolytes for this work were prepared using a salt:solvent molar ratio of 1:2, with the solvent being sulfolane. However, the salts Li[6cPFSI], Li[5cPFSI], Li[TfN3O1], Li[TfN5], Li[MsNMs], $Li[PO_2F_2]$, and $Li[MsC(CN)_2]$ were not sufficiently soluble both at 1:2 and even 1:3 molar ratios. Some salts such as Li[6cPFSI] dissolved in the heat (in this specific case on a hotplate around 55 °C), but solidified or crystallised out when cooling to ambient temperature. Even at 70°C, $[Li(SL)_3]$[TfN3O1] and $[Li(SL)_3]$[TfN5] still contained solid residue. The crystals obtained after cooling down $[Li(SL)_3]$[TfN3O1], $[Li(SL)_3]$[TfN5], and [Li(G4)][TfN3O1] were used for X-ray crystallography. We also tested additional solubilities in tetraglyme at a molar ratio of 1:1. $Li[PO_2F_2]$ and Li[6cPFSI] dissolved well in 1:1 LiX:G4, while Li[TfN3O1] and Li[MsNMs] were insoluble.

Mass spectra were recorded on a LaChrom Ultra (Hitachi High-Tech Corporation) and NanoFrontier LD (Hitachi Hich-Tech Corporation). Samples were prepared at a concentration of 10-100 ppm with equal amount of triethyl amine in acetonitrile and measured in ESI mode after direct injection. For calibration, TFA/NaTFA were used as negative ion marker.

There are several similar approaches in the literature for the desired kind of triflylation and mesylation reactions.[9,13,21,47–50] The malononitriles in this work are known to form adducts with HCl.[47,51,52] HTfNAc and Li[TfNMs] were prepared as described previously.[14] We observed a melting point of 94.4 °C for HTfNAc *via* differential scanning calorimetry (DSC) on a DSC7020 (Hitachi High-Tech Science). It is preferable to wash the lithium salts by either grinding them in a dry chamber or stirring them with suitable solvents under Schlenk conditions for several hours. Li[TfNMs] was purified by washing 3x with methylene chloride and 2x with pentane *via* filter cannula under Schlenk conditions, followed by drying as described above. The same purification method was used for commercial Li[6cPFSI].

2.1. Electrolyte precursors

N-pentyl-trifluoromethanesulfonamide

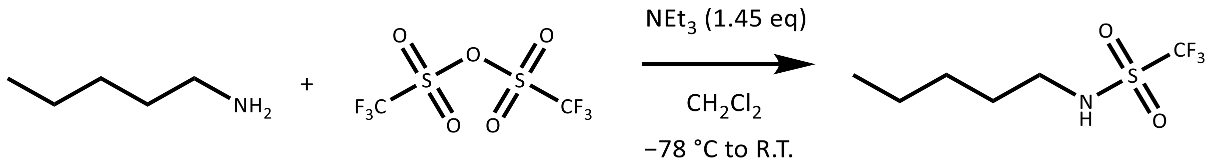

7.54 g Amylamine (86.5 mmol / 1.00 eq) and 17.5 mL triethyl amine (12.7 g / 125 mmol / 1.45 eq) were dissolved together in 180 mL dry methylene chloride and cooled to −78 °C in an external dry ice / methanol bath. 16 mL triflic anhydride (26.8 g / 95.1 mmol / 1.10 eq) in 80 mL dry methylene chloride were added to the reaction mixture over 2 h. After completion of the addition, the reaction mixture was allowed to warm to ambient temperature, and stirred at ambient temperature for 5 h. The majority of the solvent was removed under reduced pressure, 35 mL NaOH 4 M were added, and the mixture washed three times with 40 mL methylene chloride each. Then, 40 mL of HCl 6 M were added, and the aqueous phase extracted three times with 40 mL methylene chloride each. The combined organic phases were dried with $MgSO_4$, the solvent removed under reduced pressure, and the residue distilled under reduced pressure (≈60°C bath temperature, 0.3 mbar vacuum, Schlenk line), giving 10.2 g of the title compound (46.5 mmol / 54% isolated yield).

^{1}H NMR (MeCN-d_3, 500 MHz, δ in ppm): 6.51 (br s, 1H, N-***H***), 3.21 (t, $^3J_{H/H}$ = 7.1 Hz, 2H, N-C$\boldsymbol{H_2}$), 1.55 (p, $^3J_{H/H}$ = 7.1 Hz, 2H, N-CH_2-C$\boldsymbol{H_2}$), 1.38-1.26 (m, 4H, N-C_2H_4-$C_2\boldsymbol{H_4}$), 0.90 (t, $^3J_{H/H}$ = 6.9 Hz, 3H, C$\boldsymbol{H_3}$).

^{13}C{^{1}H} NMR (MeCN-d_3, 126 MHz, δ in ppm): 120.97 (q, $^1J_{C/F}$ = 320.7 Hz, $\boldsymbol{C}F_3$), 45.03 (s, N-$\boldsymbol{C}H_2$), 30.40 (s, N-CH_2-$\boldsymbol{C}H_2$), 29.09 (s, N-C_2H_4-$\boldsymbol{C_2}H_4$), 22.80 (s, N-C_2H_4-$\boldsymbol{C_2}H_4$), 14.17 (s, $\boldsymbol{C}H_3$).

^{19}F NMR (MeCN-d_3, 471 MHz, δ in ppm): −78.63 (t, $^5J_{F/H}$ = 0.8 Hz).

Lithium N-pentyl-trifluoromethanesulfonamide

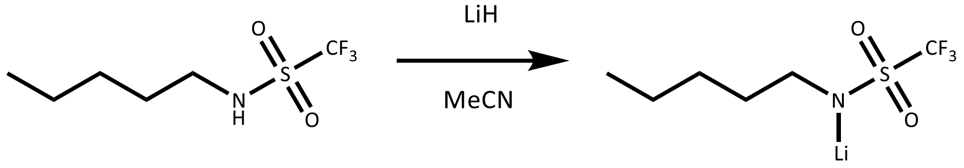

10.1 g N-(3-methoxypropyl) trifluoromethanesulfonamide (46.0 mmol / 1.00 eq) was slowly added with stirring to a suspension of 0.556 g lithium hydride (69.9 mmol / 1.52 eq) in 100 mL dry acetonitrile in a 200 mL Schlenk flask. The product precipitated during the addition. After the addition was complete, the mixture was heated to 85°C to redissolve completely, and then filtered hot through a filter cannula (glass fibre filter paper / Teflon tape / PTFE cannula) into a separate

Schlenk flask. The filtrate was kept at −18 °C overnight to allow the product to crystallise. The supernatant solvent was then removed *via* cannula, and the product washed three times with methylene chloride (20 mL – 50 mL – 5 mL), followed by 5 mL of pentane. The crystalline product was then dried at 40 °C overnight under vacuum, giving 8.06 g of the target compound (35.8 mmol / 78% isolated yield).

^{1}H NMR (DMSO-d$_6$, 500 MHz, δ in ppm): 2.82 (t, $^{3}J_{H/H}$ = 6.7 Hz, 2H, N-C***H***$_2$), 1.35-1.12 (m, 6H, N-CH$_2$-C$_3$***H***$_6$), 0.82 (t, $^{3}J_{H/H}$ = 6.6 Hz, 3H, C***H***$_3$).

^{13}C{^{1}H} NMR (DMSO-d$_6$, 126 MHz, δ in ppm): 123.15 (q, $^{1}J_{C/F}$ = 335.2 Hz, ***C***F$_3$), 45.78 (s, N-***C***H$_2$), 32.83 (s, N-CH$_2$-***C***H$_2$), 29.18 (s, N-C$_2$H$_4$-***C***$_2$H$_4$), 22.21 (s, N-C$_2$H$_4$-***C***$_2$H$_4$), 14.16 (s, ***C***H$_3$).

^{19}F NMR (DMSO-d$_6$, 471 MHz, δ in ppm): −75.52 (br s).

N-((Trifluoromethyl)sulfonyl)acetamide

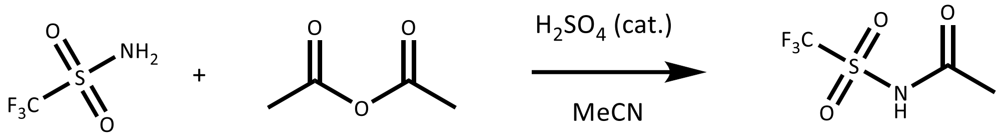

24.8 g Trifluoromethane sulfonamide (166 mmol / 1.00 eq), 50 mL acetic anhydride (529 mmol / 3.19 eq), 0.30 mL sulfuric acid and 250 mL dry acetonitrile were added together in a 500 mL round bottom flask under Schlenk conditions. The reaction mixture was stirred for 15 min at ambient temperature, followed by slow heating to 60 °C. After being stirred at 60 °C for 3 h, the mixture was cooled to ambient temperature and stirred overnight. Acetonitrile was removed under reduced pressure and the residue was stirred with 150 mL toluene. The toluene was then refrigerated in dry ice for 5 h to crystallise the product, and the supernatant decanted. This was followed by a second recrystallisation with 150 mL toluene, briefly stirring at 50 °C, after which the flask was kept in a fridge at 4 °C overnight. The resulting solid was washed with pentane twice. To this end, 65 mL pentane were added, the system was warmed up to room temperature and cooled to dry ice temperature for 3 h. After quickly decanting the solvent in the cold, the process was repeated using 100 mL pentane which was added and the system was warmed up to room temperature overnight, cooled to dry ice temperature for 2 h and finally the solvent was decanted. Then, the resulting product was dried by purging with Ar and transferred to a sublimation kit. Finally, two overnight sublimations yielded 14.5 g *N*-((trifluoromethyl)sulfonyl)acetamide (76 mmol / 46%). ^{1}H NMR (MeCN-d$_3$, 400 MHz, *δ* in ppm): 9.89 (br s, 1H, N–**H**), 2.13 (s, 3H, COC**H$_3$**); ^{13}C{^{1}H} NMR (MeCN-d$_3$, 126 MHz, *δ* in ppm): 167.77 (s, **C**OCH$_3$), 119.32 (q, $^{1}J_{C/F}$ = 320.7 Hz, **C**F$_3$), 23.49 (s, CO**C**H$_3$); ^{19}F NMR (MeCN-d$_3$, 377 MHz, *δ* in ppm): −77.67 (s, C**F**$_3$).

Lithium acetyl(trifluoromethylsulfonyl)imide

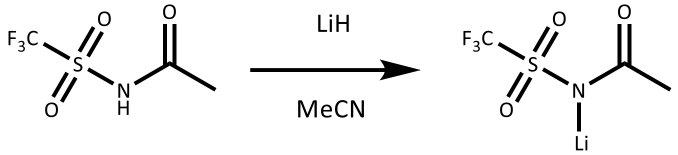

0.67 g lithium hydride (84 mmol / 1.12 eq) was suspended in 10 mL dry acetonitrile and added slowly into 14.40 g H[TfNAc] (75 mmol / 1.00 eq) in 15 mL dry acetonitrile. The reaction mixture was stirred for 3 h and excess LiH was removed using filter cannula. After that, solvent was removed by vacuum distillation. The residue was washed with 15 mL dry dichloromethane three times. Thus, 13.5 g lithium(*N*-((trifluoromethyl)sulfonyl)acetamide) (69 mmol / 91%) was obtained after vacuum drying

for 1 day. Crystals suitable for X-ray crystallography were grown using vapour diffusion with acetonitrile as solvent and chloroform as antisolvent.

^{1}H NMR (DMSO-d$_6$, 500 MHz, δ in ppm): 1.78 (s, COC**H**$_3$); ^{13}C{^{1}H} NMR (DMSO-d$_6$, 126 MHz, δ in ppm): 176.35 (s, **C**OCH$_3$), 120.92 (q, $^{1}J_{C/F}$ = 325.1 Hz, **C**F$_3$), 27.26 (s, CO**C**H$_3$); ^{19}F NMR (DMSO-d$_6$, 471 MHz, δ in ppm): –77.68 (s, C**F**$_3$).

N-(3-methoxypropyl) trifluoromethanesulfonamide

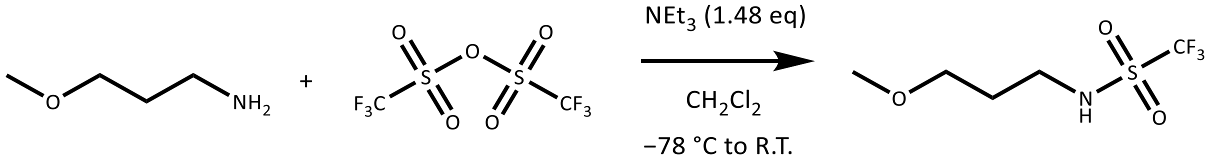

10 mL 3-methoxypropylamine (8.50 g / 95.4 mmol / 1.00 eq) and 20 mL triethyl amine (14.3 g / 141 mmol / 1.48 eq) were dissolved in 170 mL dry methylene chloride and cooled to −78 °C in an external dry ice / methanol bath. To this mixture, 17 mL triflic anhydride (29.1 g / 103 mmol / 1.08 eq) in 80 mL dry methylene chloride were added to the reaction mixture over 1.5 h. After completion of the addition, the reaction mixture was allowed to warm to ambient temperature, and stirred at ambient temperature for 5 h. The majority of the solvent was removed under reduced pressure, 40 mL NaOH 4 M were added, and the mixture washed three times with methylene chloride (50 mL – 50 mL – 30 mL). Then, 30 mL of HCl 6 M were added, and the aqueous phase extracted three times with methylene chloride (50 mL – 50 mL – 30 mL). The combined organic phases were dried with $MgSO_4$ and the solvent removed under reduced pressure. Thus, 14.6 g of the crude product containing some methylene chloride were obtained (12.4 g target compound / 56 mmol / 59% raw yield). The combined raw products of three batches synthesised as above (ca. 38 g product and 7 g methylene chloride) were further purified as follows. The methylene chloride was evaporated at reduced pressure (200 mbar / 30°C bath temperature). Then, 50 mL pentane were added, the mixture stirred, and kept at −18 °C overnight to allow the (lower) product phase to solidify. The supernatant solvent was decanted under argon, and the process repeated (addition of 50 mL pentane, storage at −18 °C overnight, decanting the solvent). Then, the remaining pentane was removed from the product phase under reduced pressure, and the residue distilled in vacuum (≈70°C bath temperature, 0.4 mbar vacuum, Schlenk line), giving 30 g of the title compound (≈80% yield of the purification step).

HRMS, ESI$^-$: m/z found 220.0256, calc. 220.0261 ($C_5H_9F_3NO_3S^-$).

^{1}H NMR (MeCN-d$_3$, 500 MHz, δ in ppm): 6.55 (br s, 1H, N-***H***), 3.41 (t, $^{3}J_{H/H}$ = 5.9 Hz, 2H, O-C***H***$_2$), 3.30 (tq, $^{3}J_{H/H}$ = 6.8 Hz, $^{5}J_{H/F}$ = 0.8 Hz, 2H, N-C***H***$_2$), 3.27 (s, 3H, C***H***$_3$), 1.78 (tt, $^{3}J_{H/H}$ = 6.8 Hz, $^{3}J_{H/H}$ = 5.9 Hz**,** 2H, N-CH$_2$-C***H***$_2$).

^{13}C{^{1}H} NMR (MeCN-d$_3$, 126 MHz, δ in ppm): 120.93 (q, $^{1}J_{C/F}$ = 320.8 Hz, ***C***F$_3$), 70.15 (s, O-***C***H$_2$), 58.83 (s, ***C***H$_3$), 42.72 (s, N-***C***H$_2$), 30.75 (s, N-CH$_2$-***C***H$_2$).

^{19}F NMR (MeCN-d$_3$, 471 MHz, δ in ppm): −78.60 (t, $^{5}J_{F/H}$ = 0.8 Hz).

Lithium (3-methoxypropyl)(trifluoromethanesulfonyl)amide

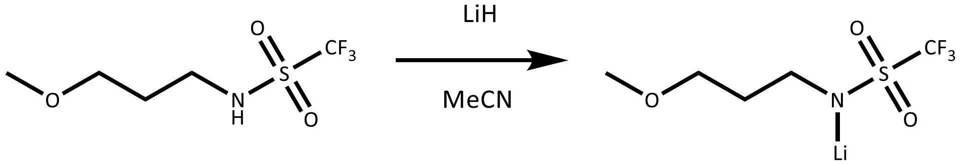

12 mL N-(3-methoxypropyl) trifluoromethanesulfonamide (16.1 g / 72.8 mmol / 1.00 eq) was slowly added with stirring to a suspension of 0.692 g lithium hydride (87.0 mmol / 1.20 eq) in 150 mL dry acetonitrile in a 200 mL Schlenk flask placed in a 400 mL water bath as heat sink. The product precipitates. After addition was complete, the mixture was heated to 80°C to redissolve completely, and then filtered through a filter cannula (glass fibre filter paper / Teflon tape / PTFE cannula) into a separate Schlenk flask. The filtrate was kept at −18 °C overnight to allow the product to crystallise. The supernatant solvent was then removed *via* cannula, and the product washed three times with 20 mL methylene chloride each, followed by a small amount of pentane. The crystalline product was then dried at 40 °C overnight under vacuum. The critical step is usually the filtration. Note: extended stirring at 80°C should be avoided.

^{1}H NMR (DMSO-d$_6$, 500 MHz, δ in ppm): 3.31 (t, $^{3}J_{H/H}$ = 6.7 Hz, 2H, O-C***H***$_2$), 3.18 (s, 3H, C***H***$_3$), 2.87 (tq, $^{3}J_{H/H}$ = 6.9 Hz, $^{5}J_{H/F}$ = 1.6 Hz, 2H, N-C***H***$_2$), 1.51 (p, $^{3}J_{H/H}$ = 6.8 Hz**,** 2H, N-CH$_2$-C***H***$_2$).

^{13}C{^{1}H} NMR (DMSO-d$_6$, 126 MHz, δ in ppm): 123.03 (q, $^{1}J_{C/F}$ = 335.3 Hz, ***C***F$_3$), 70.70 (s, O-***C***H$_2$), 57.77 (s, ***C***H$_3$), 42.70 (s, N-***C***H$_2$), 33.00 (s, N-CH$_2$-***C***H$_2$).

^{19}F NMR (DMSO-d$_6$, 471 MHz, δ in ppm): −75.56 (t, $^{5}J_{F/H}$ = 1.6 Hz).

Bis(methanesulfonyl)amide

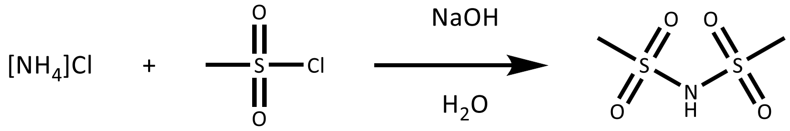

A solution of 27.2 g ammonium chloride (509 mmol / 1.00 eq) in 125 mL water was prepared and cooled to 0 °C in an external ice bath. To this solution, 118 g methanesulfonyl chloride (1.03 mol / 2.02 eq) were added together with a solution of 81.5 g sodium hydroxide (2.04 mol / 4.00 eq) in 200 mL water from two separate dropping funnels at the same time over the course of 1.5 h. After addition, the pH was adjusted to pH=7 with a small amount of NaOH 4 M. The reaction mixture was split into two portions due to its large volume. Each portion was transferred to a Kutscher-Steudel type perforator for heavier solvents, and washed continuously for 24 hours with methylene chloride. The methylene chloride was then replaced with fresh methylene chloride, 40 mL of 37% hydrochloric acid were added slowly to the aqueous phase in the extractor, and the acidified aqueous phase extracted for 24-48 h with methylene chloride. The organic phases from both portions containing the raw product dissolved in methylene chloride were combined and the solvent removed under reduced pressure. The product was then further purified by recrystallisation from 100 mL glacial acetic acid. The crystals were thoroughly washed twice with 50 mL pentane each, and dried in vacuum overnight giving 21.7 g of the target compound (125 mmol / 25% yield). Melting point observed at 152 °C.

^{1}H NMR (Acetone-d$_6$, 400 MHz, δ in ppm): 3.29 (s, C***H***$_3$).

^{13}C{^{1}H} NMR (Acetone -d$_6$, 100 MHz, δ in ppm): 43.42 (s, ***C***H$_3$).

Lithium bis(methanesulfonyl)amide

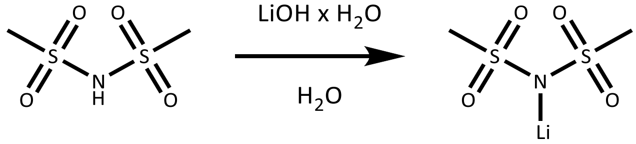

To 9.40 g bis(methanesulfonyl)amide (54.2 mmol / 1.00 eq) in 20 mL water was slowly added 2.28 g lithium hydroxide monohydrate (54.3 mmol / 1.00 eq). Then, activated carbon was added, stirred,

filtered through a syringe filter, and the water removed in vacuum. The resulting raw product was washed with methylene chloride, followed by pentane, and dried as described above. The target compound was obtained in quantitative yield.

^{1}H NMR (DMSO-d$_6$, 400 MHz, δ in ppm): 2.70 (s, C***H***$_3$).

^{13}C{^{1}H} NMR (DMSO -d$_6$, 100 MHz, δ in ppm): 42.75 (s, ***C***H$_3$).

2-(methylsulfonyl)malononitrile

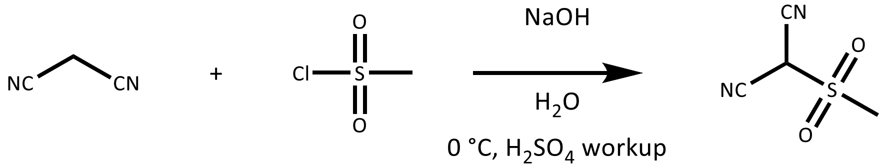

20.0 g malononitrile (freshly distilled from P_4O_{10}, 303 mmol / 1.00 eq) was dissolved in 100 mL water and cooled to 0 °C in an external ice bath. 23.5 mL Methanesulfonyl chloride (34.1 g / 298 mmol / 0.98 eq) and 24.3 g sodium hydroxide (608 mmol / 2.00 eq) dissolved in 120 mL water were added dropwise with stirring over the course of 1 h so that pH≈6, monitored *via* the use of a pH meter. After addition was complete, stirring was continued for 30 min. Then, 80 mL H_2SO_4 50% (v/v) was added. The reaction mixture was extracted four times with 60 mL ethyl acetate each. The combined organic extracts were dried with magnesium sulphate and the solvent removed under reduced pressure (bath temperature 25 °C). The residue was extracted four times with ≈100 mL portions of toluene, each time briefly stirring at 80-90 °C. The combined toluene extracts were kept at −18 °C overnight to allow the product to crystallise, and the toluene decanted in the cold. The obtained crystals were washed with a small amount of pentane and briefly dried in vacuum. Then, the crystals were dissolved in the minimum amount of ethyl acetate (approximately 30-40 mL), and the resulting solution stirred with charcoal. Then, hexane was slowly added with stirring to reprecipitate the product, after which the solution was kept at −18 °C overnight and the supernatant solvent decanted. It is important to allow enough time for crystallisation during and after addition of the hexanes. The dissolution in ethyl acetate and reprecipitation with hexane was repeated once to obtain after drying in vacuum 6.64 g of colourless crystalline material suitable for X-ray crystallography (46.0 mmol / 15% isolated yield). Melting point observed at 89 °C, exothermic event around 163 °C (polymerisation).

Elemental analysis (CHNS): calculated for $C_4H_4N_2O_2S$: C, 33.33; H, 2.80; N, 19.43; S, 22.24. Found C, 33.44; H, 2.82; N, 17.82; S, 22.51.

^{1}H NMR (MeCN-d$_3$, 500 MHz, δ in ppm): 5.95 (br s, C***H***(CN)$_2$), 3.60 (s, C***H***$_3$).

^{13}C{^{1}H} NMR (MeCN-d$_3$, 126 MHz, δ in ppm): 108.28 (s, ***C***N), 47.39 (s, ***C***H(CN)$_2$), 41.14 (s, ***C***H$_3$).

Lithium 2-(methylsulfonyl)malononitrile

6.64 g 2-(methylsulfonyl)malononitrile (46.0 mmol / 1.00 eq) was dissolved in 10 mL water and cooled to 0 °C in an external ice bath. 1.93 g Lithium hydroxide monohydrate (46.0 mmol / 1.00 eq) in 30 mL water was added slowly with stirring. After complete addition, activated carbon was added to the slightly acidic (pH≈6) solution and the mixture stirred for 1 h. After removal of the activated carbon *via* a syringe filter, the solution was evaporated to dryness at ambient temperature under vacuum, followed by the final drying protocol. This method (neutralisation with lithium hydroxide at low temperature, addition of activated carbon, and drying after filtering) was also used for the other lithium salts in this work, specifically $LiCHTf_2$. The purity can be further increased by dissolving the lithium salt in acetone, filtering through a syringe filter, and reprecipitating with diethyl ether. Similar to the parent acid compound, the lithium salt crystallises very slowly. If the ether is added too fast,

then a liquid phase separates. It is advisable to add diethyl ether to the acetone solution while shaking until the turbid precipitate just barely redissolves, and then allowing the homogeneous clear solution to stand overnight, during which crystals form (suitable for X-ray crystallography).

Elemental analysis (CHNS): calculated for $LiC_4H_3N_2O_2S$: C, 32.01; H, 2.01; N, 18.67; S, 21.36. Found C, 31.66; H, 1.97; N, 17.38; S, 19.49.

^{1}H NMR (DMSO-d_6, 500 MHz, δ in ppm): 2.67 (s, C***H***$_3$).

^{13}C{^{1}H} NMR (DMSO-d_6, 126 MHz, δ in ppm): 119.64 (s, ***C***N), 45.60 (s, ***C***H(CN)$_2$), 41.45 (s, ***C***H$_3$).

2.2. Infrared spectra

FTIR spectra were recorded from 7800 cm^{-1} to 350 cm^{-1} with a resolution of 4 cm^{-1} on a FT/IR6000 spectrometer (JASCO, Hachioji, Tokyo, Japan) equipped with ATR PRO450-S single reflection ATR accessory (no ATR correction performed) and DLATGS detector. For each measurement, 32 scans were accumulated. The samples were prepared in a glovebox and kept under argon atmosphere until immediately before the measurement to avoid uptake of atmospheric moisture, since the primary purpose of the IR measurements here was to confirm purity including the absence of water (≈3500 cm^{-1}).

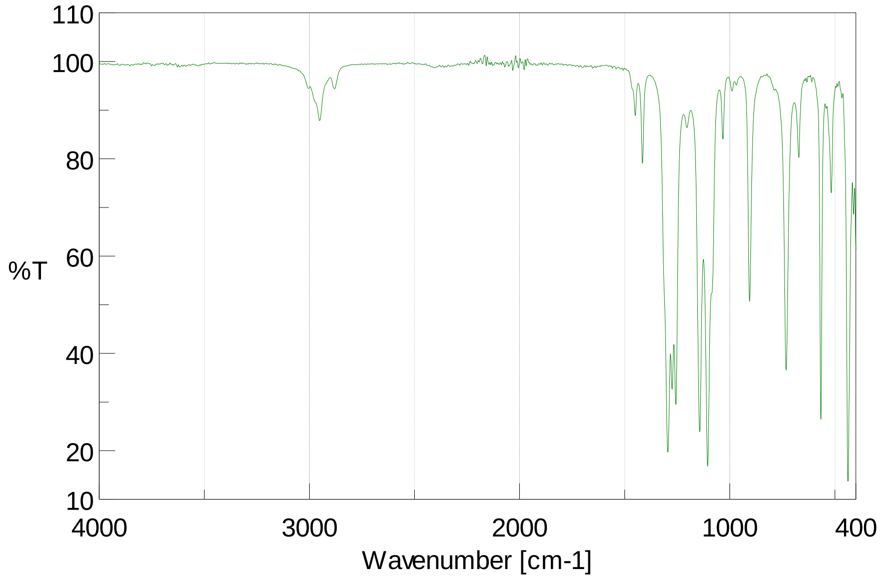

Figure S2: FTIR spectrum of neat sulfolane.

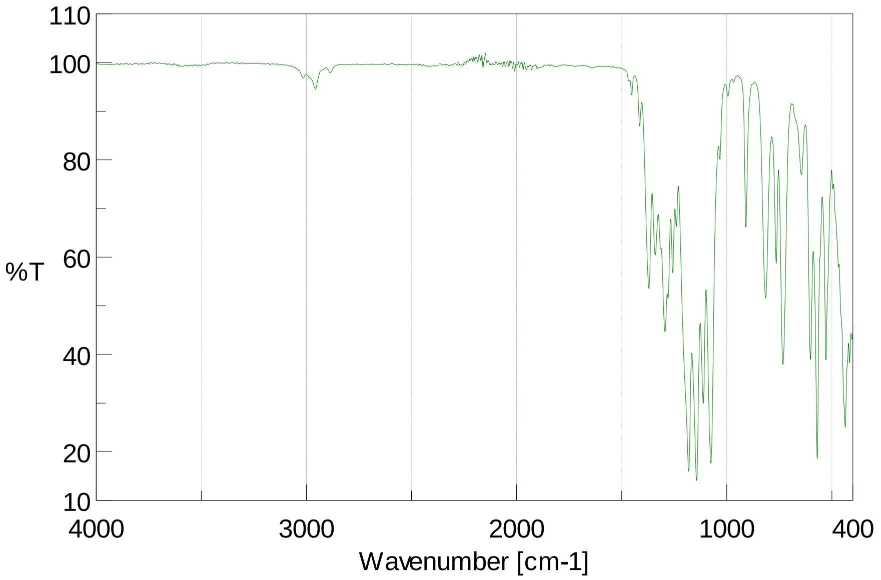

Figure S3: FTIR spectrum of [Li(SL)$_2$][TfNFs].

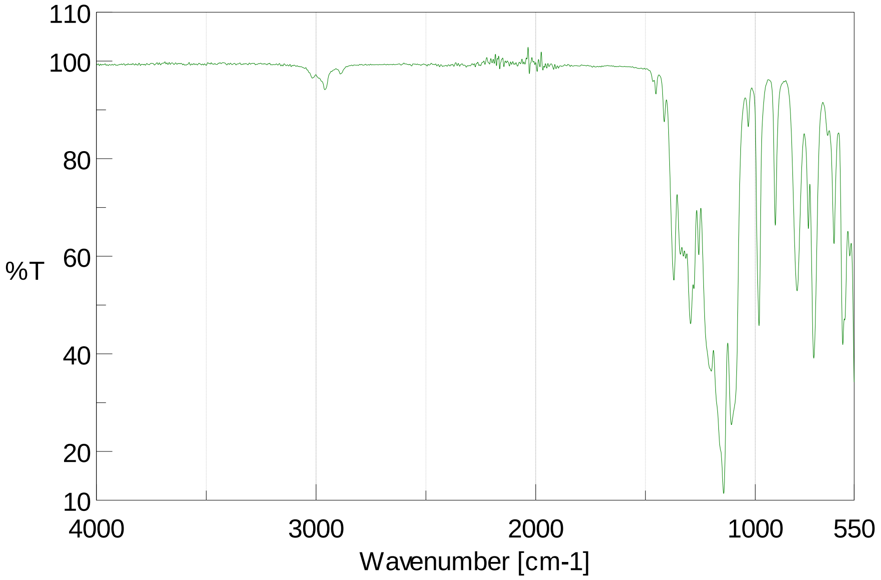

Figure S4: FTIR spectrum of [Li(SL)$_2$][PfNFs].

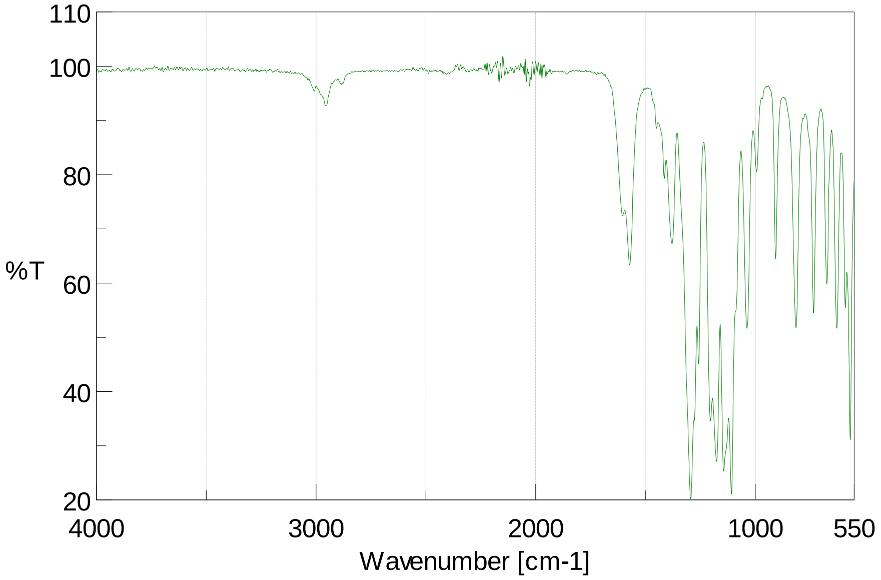

Figure S5: FTIR spectrum of $[Li(SL)_2][TfNAc]$.

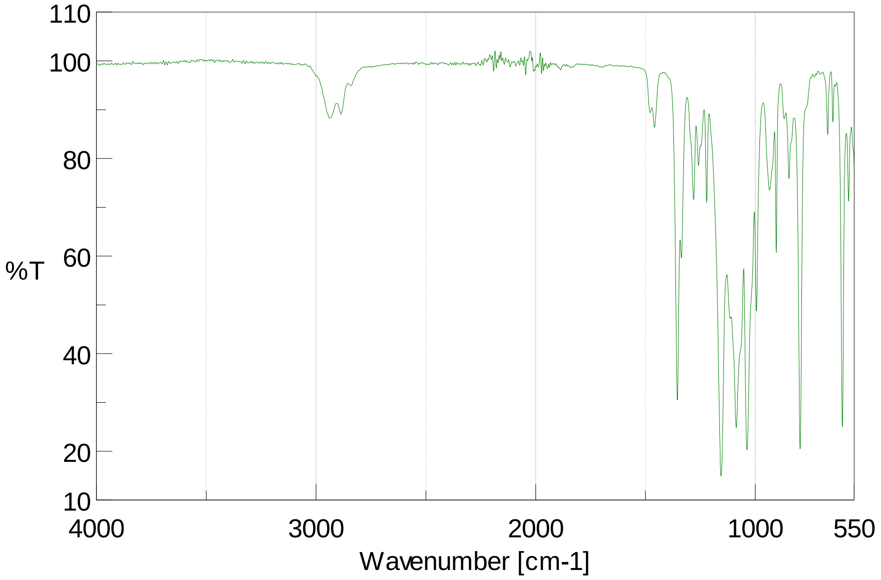

Figure S6: FTIR spectrum of [Li(G4)][6cPFSI].

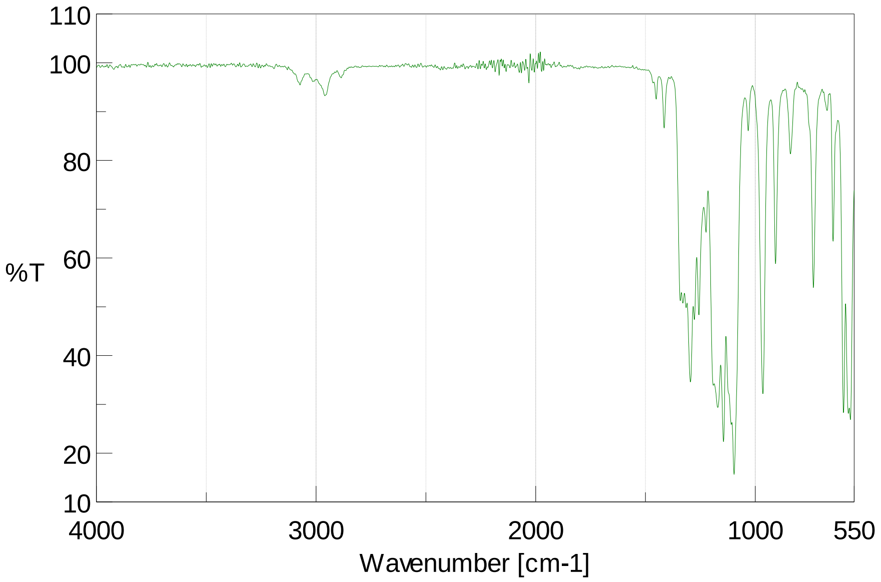

Figure S7: FTIR spectrum of [Li(SL)$_3$][CHTf2].

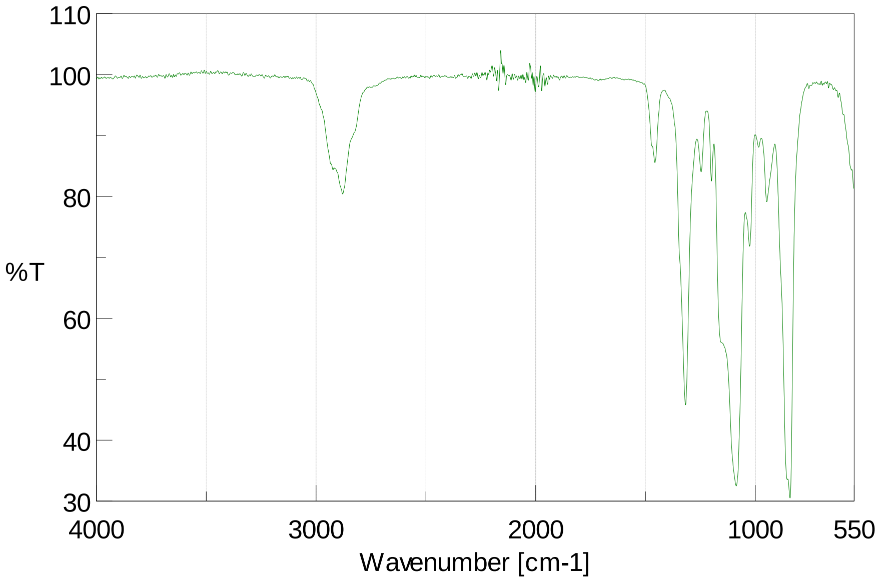

Figure S8: FTIR spectrum of [Li(G4)][PO_2F_2].

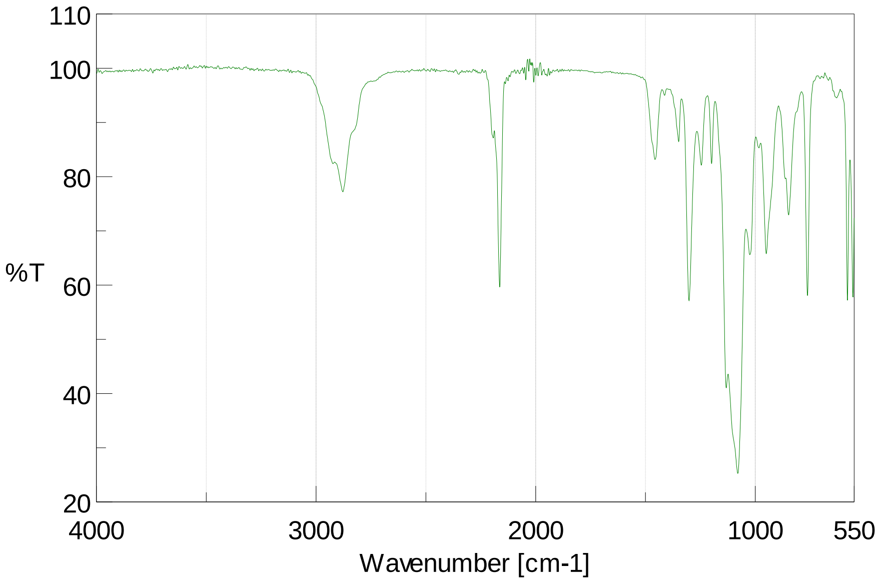

Figure S9: FTIR spectrum of $[Li(G4)_2][MsC(CN)_2]$.

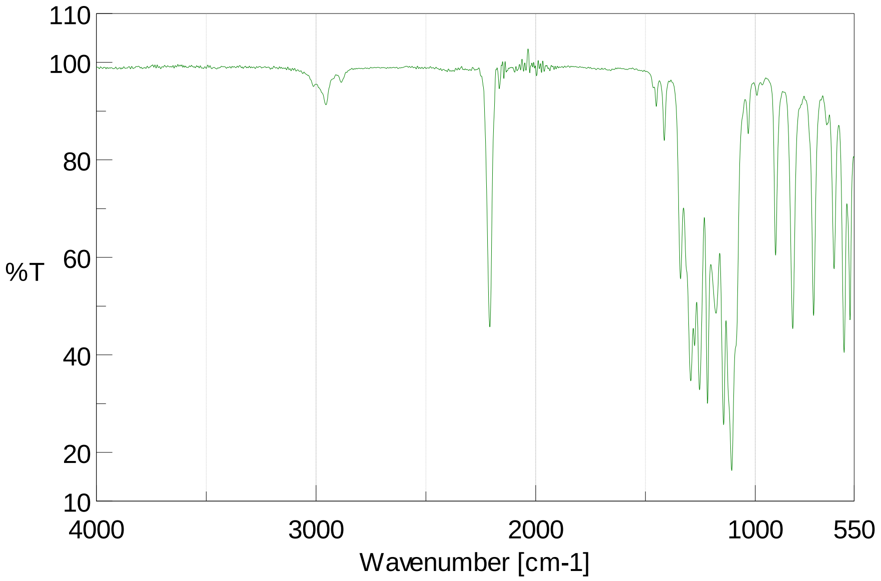

Figure S10: FTIR spectrum of $[Li(SL)_3][TfNCN]$.

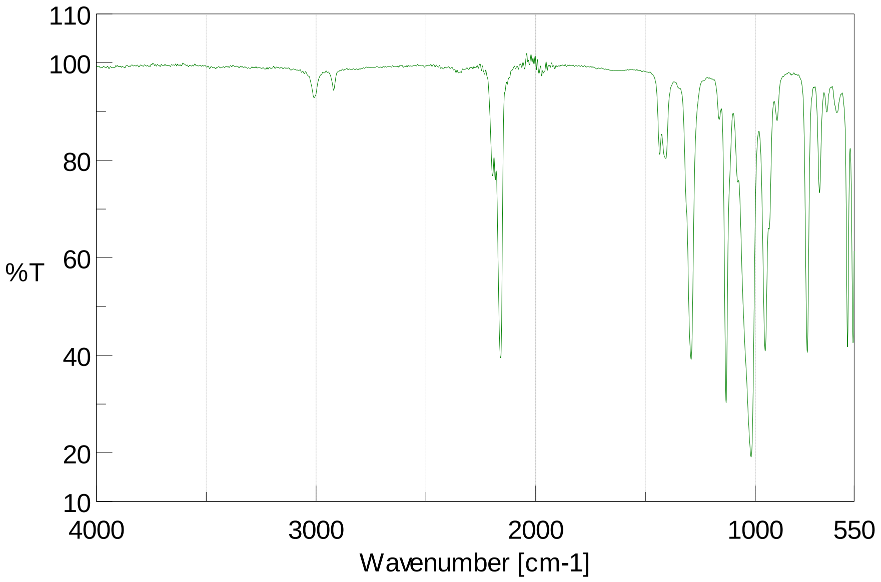

Figure S11: FTIR spectrum of $[Li(DMSO)_3][MsC(CN)_2]$.

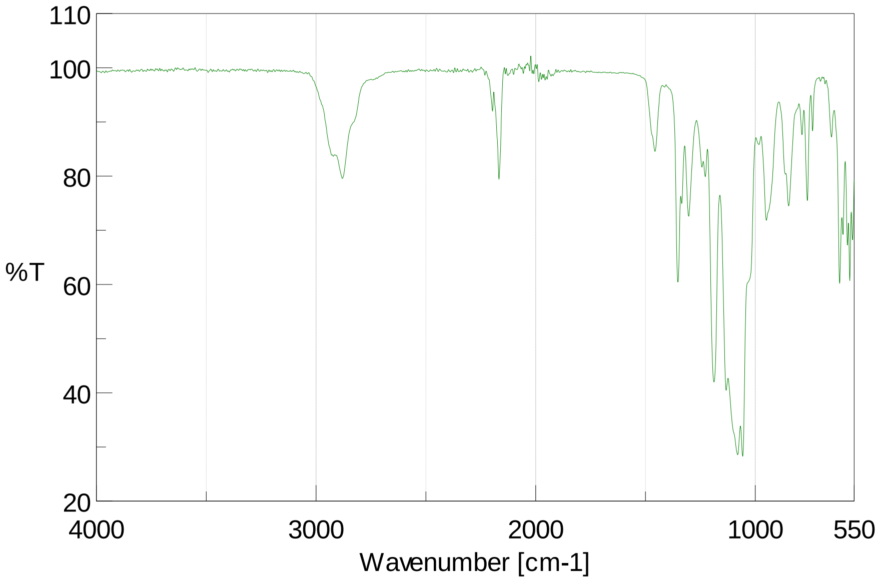

Figure S12: FTIR spectrum of $[Li_2(G4)_2][MsC(CN)_2][TfNTf]$.

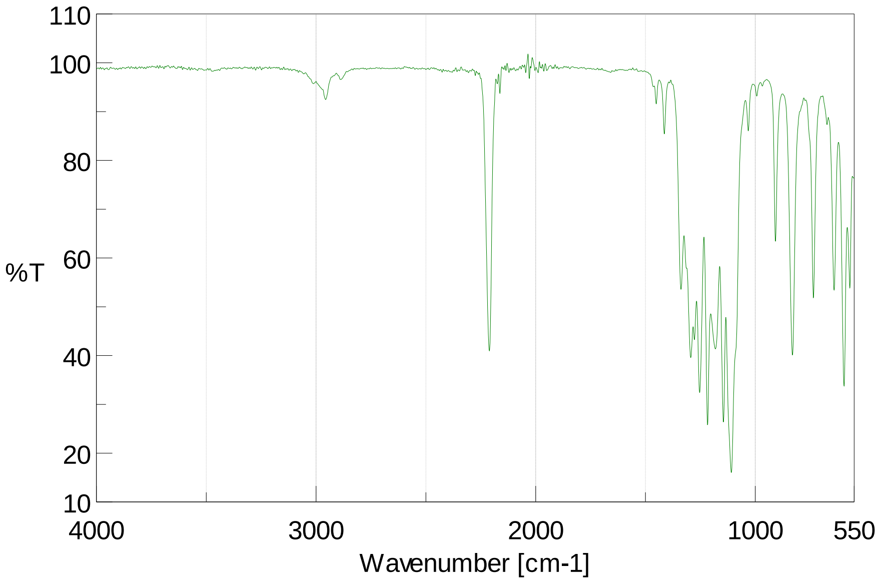

Figure S13: FTIR spectrum of $[Li(SL)_2][TfNCN]$.

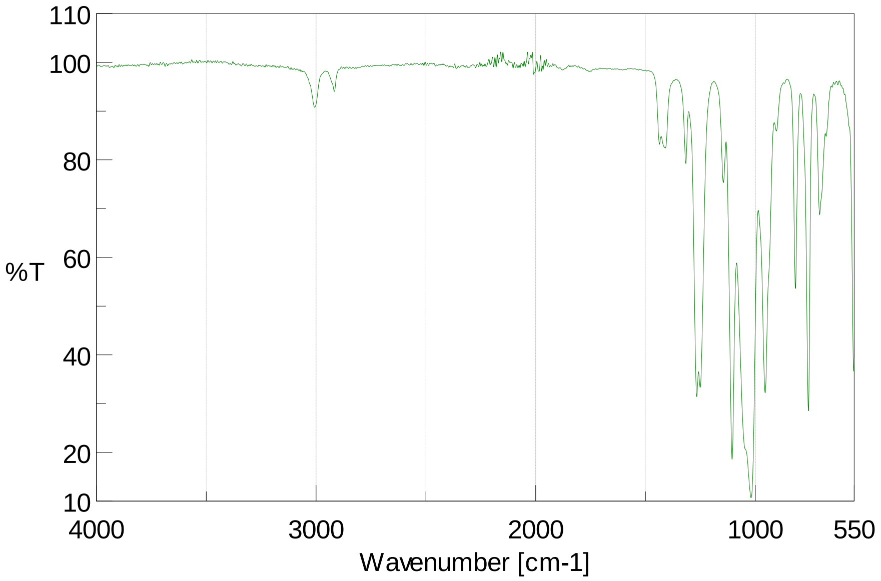

Figure S14: FTIR spectrum of $[Li(DMSO)_3][MsNMs]$

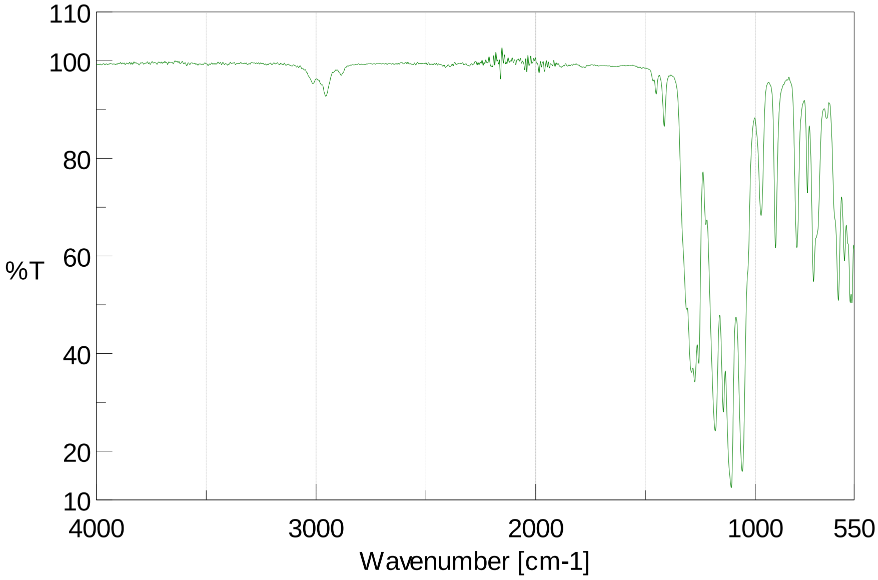

Figure S15: FTIR spectrum of [Li(SL)$_2$][TfNMs]

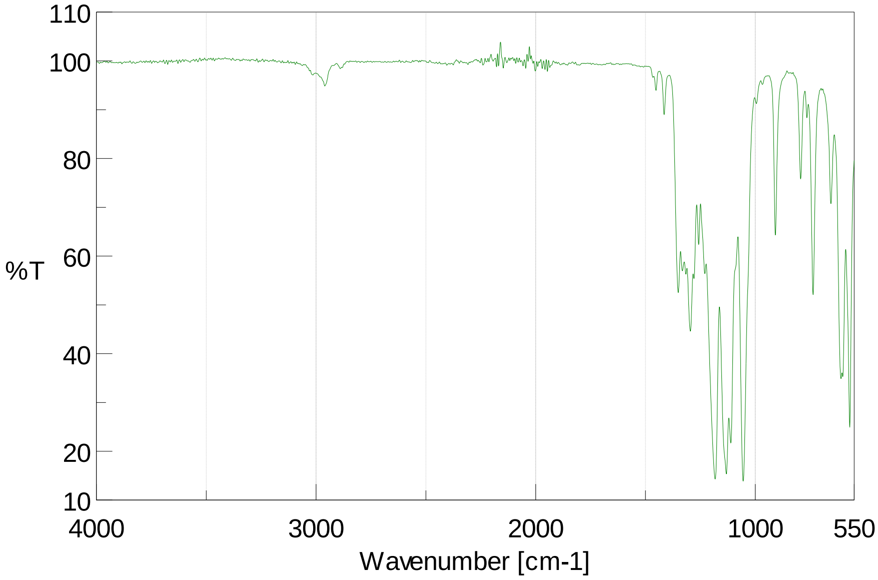

Figure S16: FTIR spectrum of [Li(SL)$_2$][TfNTf].

3. Thermal Transitions *via* DSC

Differential scanning calorimetry was performed on a DSC7020 (Hitachi) equipped with liquid nitrogen cooling. The sample chamber was flushed with nitrogen (100 mL/min) during the measurement.

DSC pans (Aluminium, 3MPa, 6 mm O.D., 4 mm height, 15 µL volume, Hitachi High-tech science corporation) were filled and hermetically sealed using an automatic press (Seiko Instruments Inc.) inside an argon filled glovebox with ≤0.5 ppm water (VAC vacuum atmospheres company, model OMNI-LAB). Pans were handled using clean PVDF coated tweezers (Ideal-Tek).

During the DSC measurement, the samples were first heated from 25°C to 100°C with a rate of 20°C/min and held at 100°C for 10 min to erase thermal history. A cooling step to −150°C with a rate

of −10°C/min and a hold time of 30 min followed. Finally, the samples were heated from −150°C to +150°C at a heating rate of 5°C/min; the reported thermal transitions were obtained from this final heating step, see Table S1 and Figure S18 to Figure S38. Lithium salts were measured using a different temperature profile; first cooling to −10°C, then heating to 180°C. No thermal events were observed in this heating step for Li[TfNCN], Li[TfNMs], Li[MsNMs], Li[MsC(CN)$_2$], Li[TfCHTf], Li[TfNAc], Li[TfN3O1]. For neat sulfolane, a solid-solid transition at 16.0 °C and a melting transition at 26.8 °C were observed, Figure S17, in good agreement with the literature.[53]

Table S1: Thermal transitions (glass transition temperatures T_G) for the solvate electrolytes in this work. Multiple entries correspond to repeat measurements from different samples.

Sample	T_G / °C	Sample	T_G / °C
[Li(SL)$_2$][TfNFs]	−78.4<br>−77.7<br>−77.7	[Li(SL)$_2$][TfNCN]	−72.4<br>−70.6<br>−73.5
[Li(SL)$_2$][TfNTf]	−73.5	[Li(AN)$_{1.5}$][TfNCN]	−61.3
[Li(SL)$_1$][TfNCN]	−43.1	[Li(GN)$_{1.5}$][TfNCN]	−60.8
[Li(SL)$_2$][PfNFs]	−76.1<br>−74.9	[Li(SL)$_{2.2}$][TfNCN]	−74.6
[Li(SL)$_2$][TfNMs]	−63.1	[Li(SL)$_{2.4}$][TfNCN]	−78.6
[Li(SL)$_2$][TfNAc]	−66.0<br>−69.2	[Li(SL)$_3$][TfNCN]	−84.6
[Li(SL)$_2$][TfCHTf]	−60.9<br>−71.2 [a]	[Li(G4)$_2$][MsC(CN)$_2$]	−87.1
[Li(DMSO)$_3$][MsNMs]	−63.2	[Li$_2$(G4)$_2$][MsC(CN)$_2$][TfNTf]	−90.6
[Li(DMSO)$_3$][MsC(CN)$_2$]	−75.4	[Li(G4)][TfCHTf]	−53.7
[Li(SL)$_3$][TfCHTf]	−74.2	[Li(G4)][PO$_2$F$_2$]	−93.7

[a] additional inhomogeneous, broad endothermal event between 20 °C and 80 °C, peak at ≈ 54 °C. This sample was measured without the initial heating step to erase thermal history, and the sample was aged at 4 °C for several weeks.

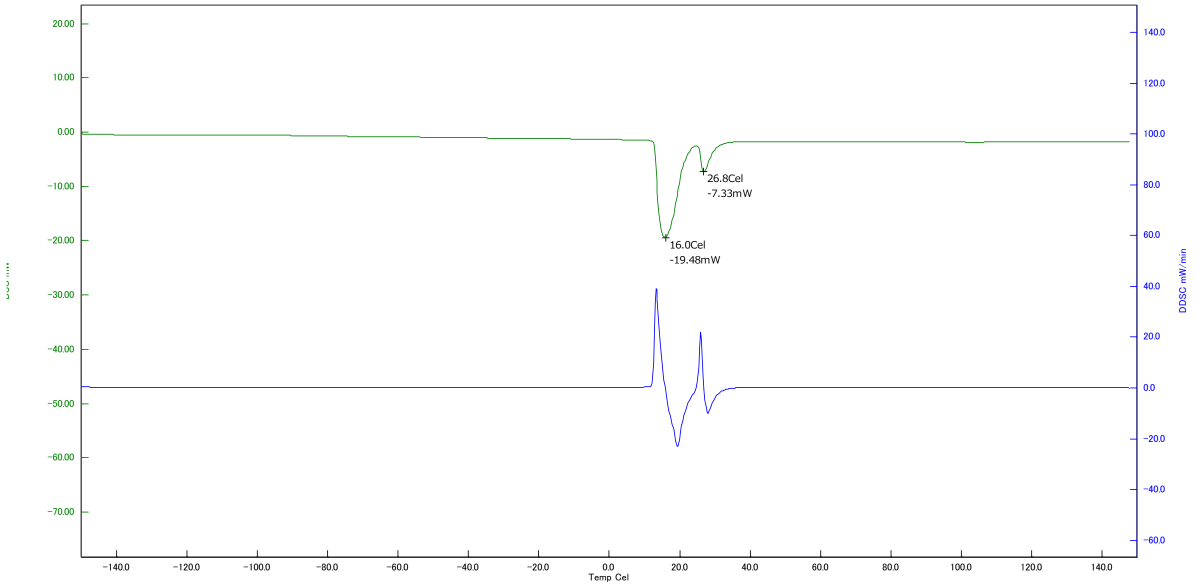

Figure S17: DSC trace (heating) of neat sulfolane.

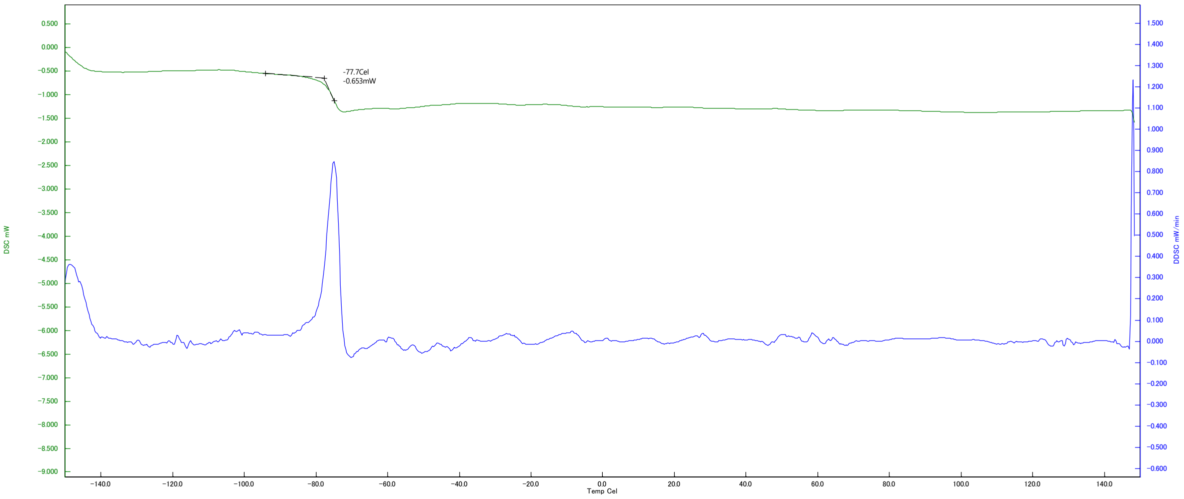

Figure S18: DSC trace (heating) of [Li(SL)$_2$][TfNFs].

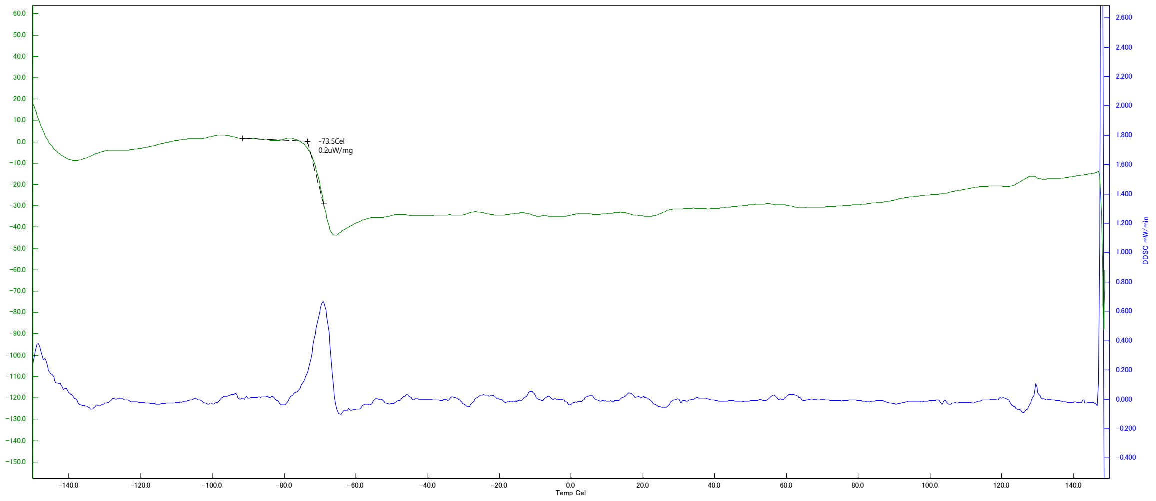

Figure S19: DSC trace (heating) of [Li(SL)$_2$][TfNTf].

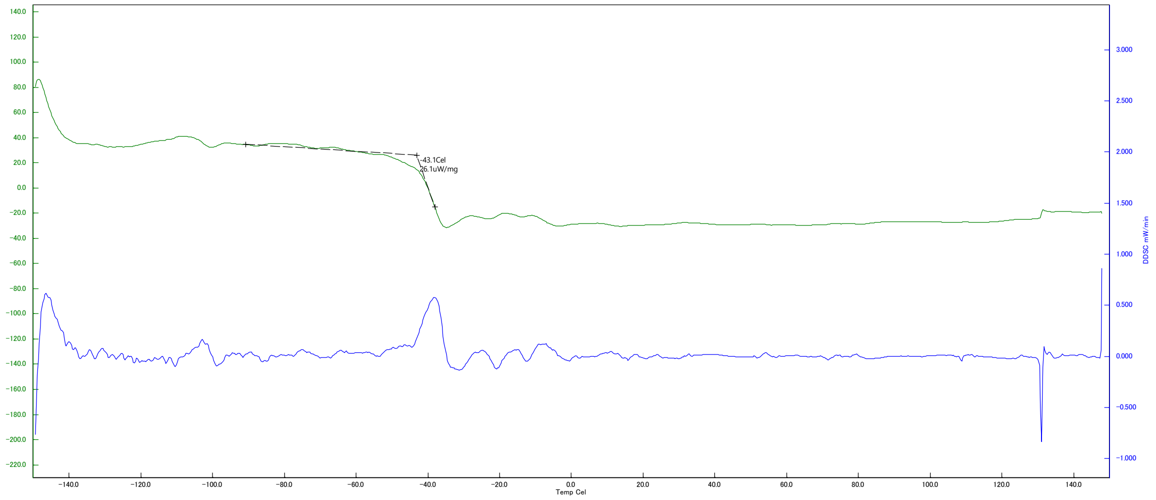

Figure S20: DSC trace (heating) of [Li(SL)$_1$][TfNCN].

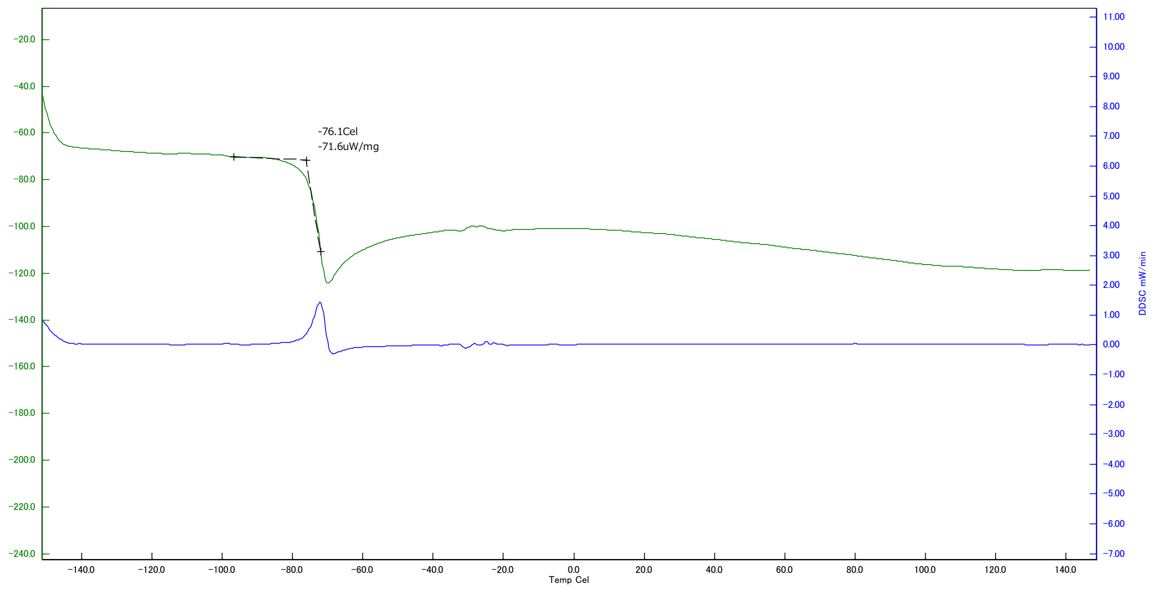

Figure S21: DSC trace (heating) of [Li(SL)$_2$][PfNFs].

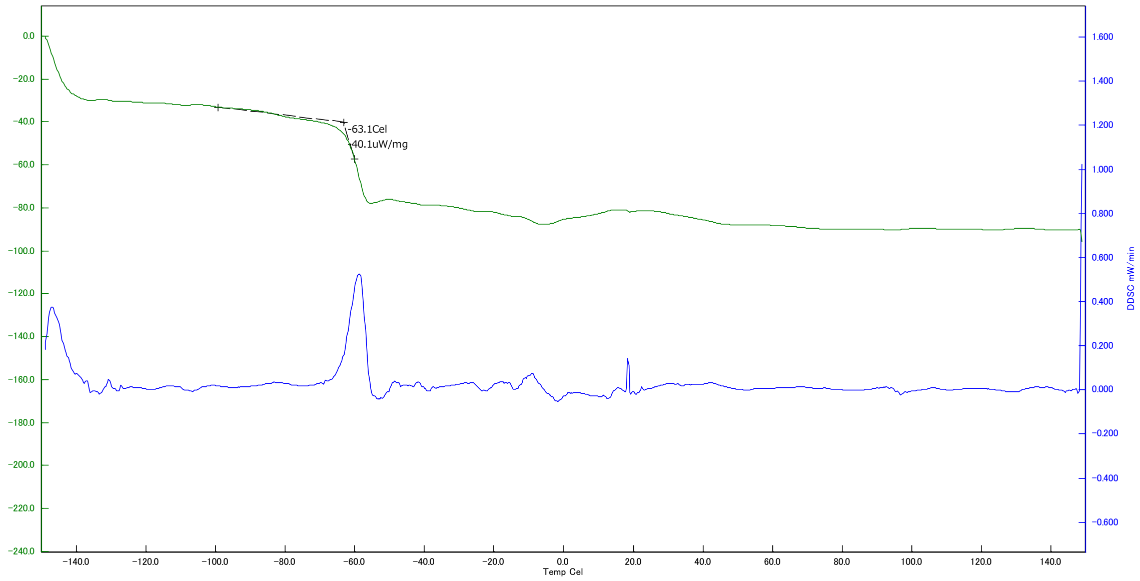

Figure S22: DSC trace (heating) of [Li(SL)$_2$][TfNMs].

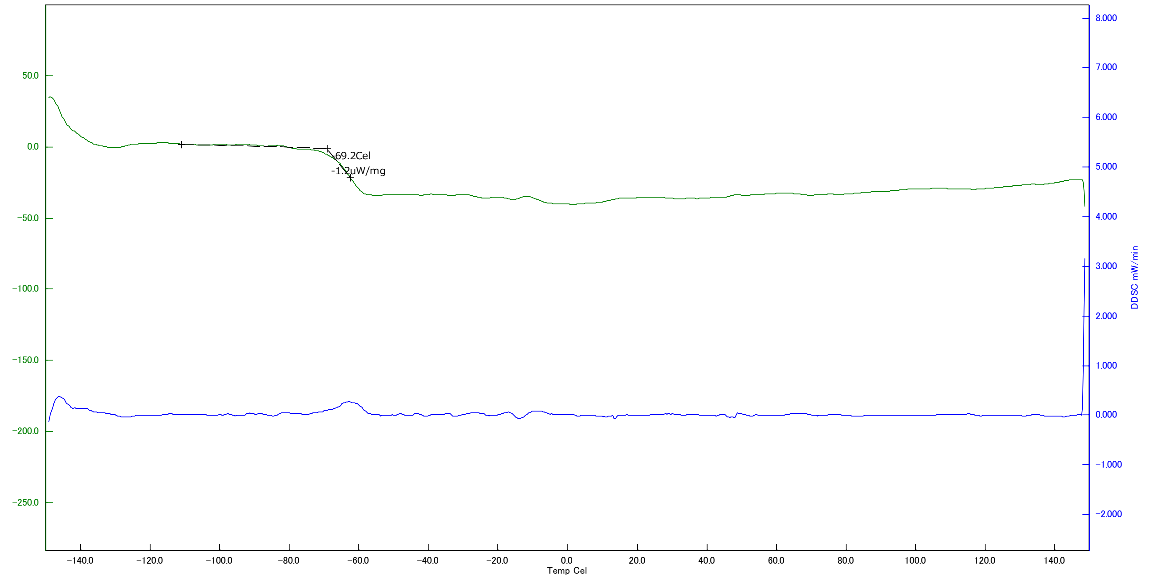

Figure S23: DSC trace (heating) of [Li(SL)$_2$][TfNAc].

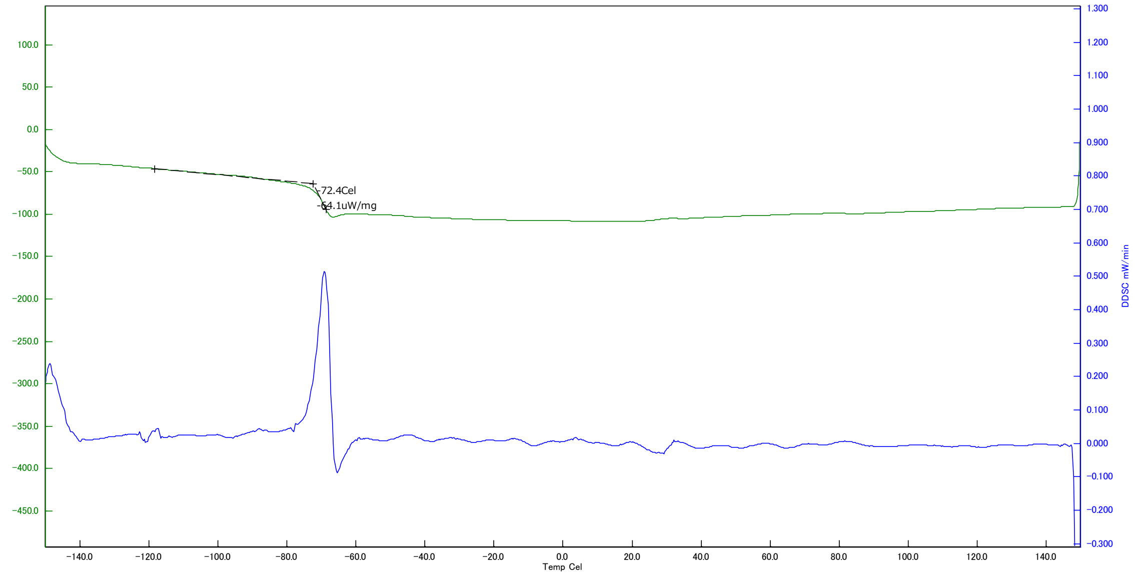

Figure S24: DSC trace (heating) of [Li(SL)$_2$][TfNCN].

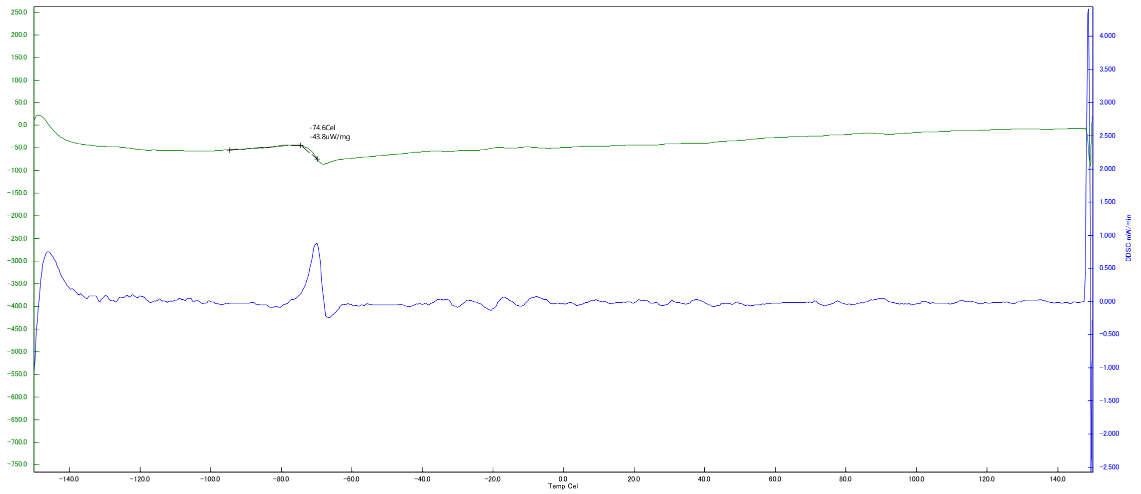

Figure S25: DSC trace (heating) of [Li(SL)$_{2.2}$][TfNCN].

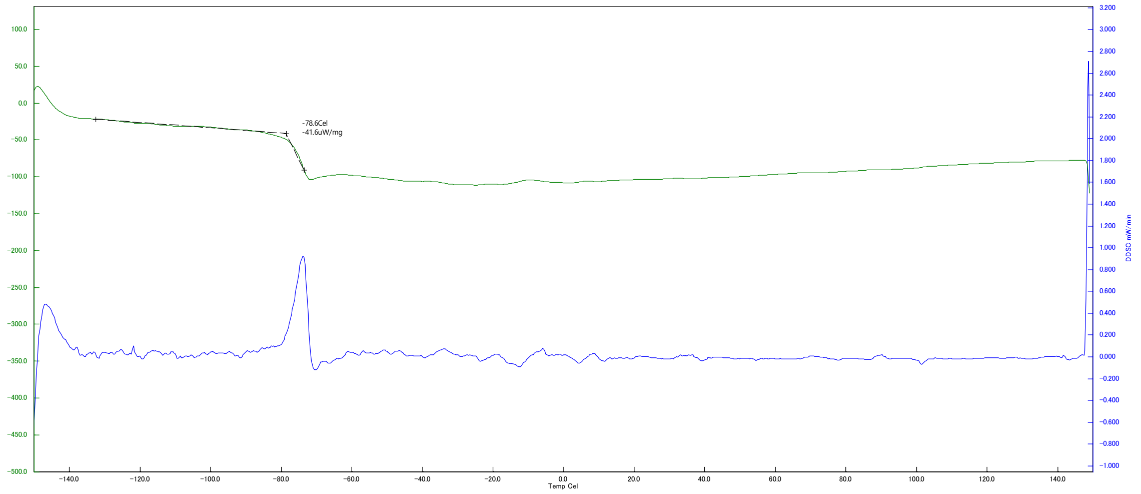

Figure S26: DSC trace (heating) of [Li(SL)$_{2.4}$][TfNCN].

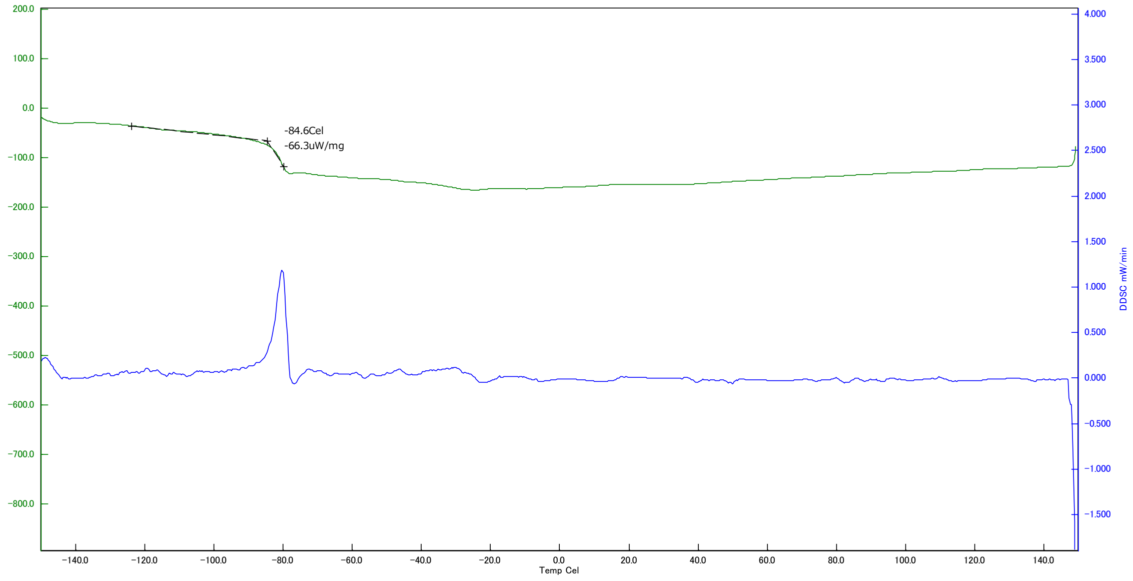

Figure S27: DSC trace (heating) of [Li(SL)$_3$][TfNCN].

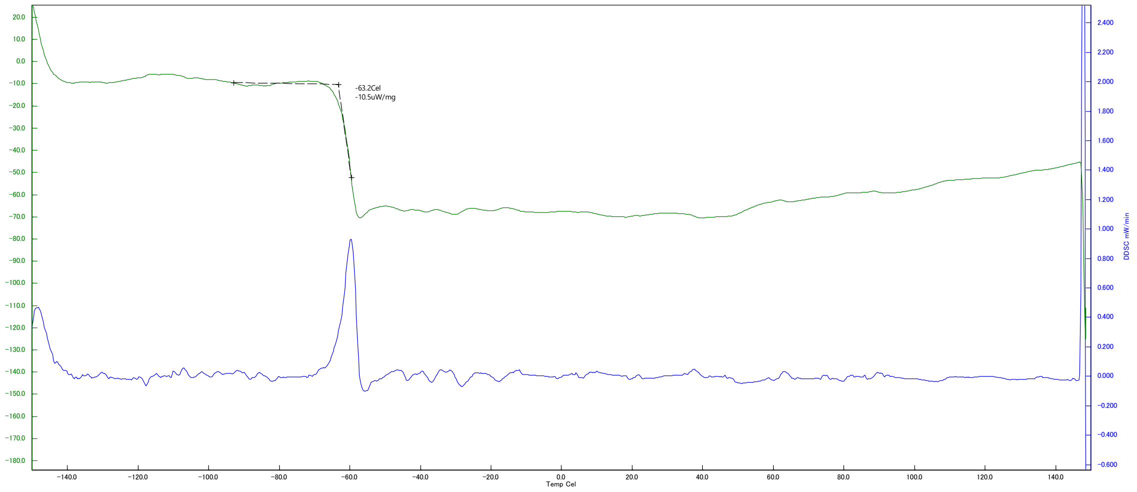

Figure S28: DSC trace (heating) of [Li(DMSO)$_3$][MsNMs].

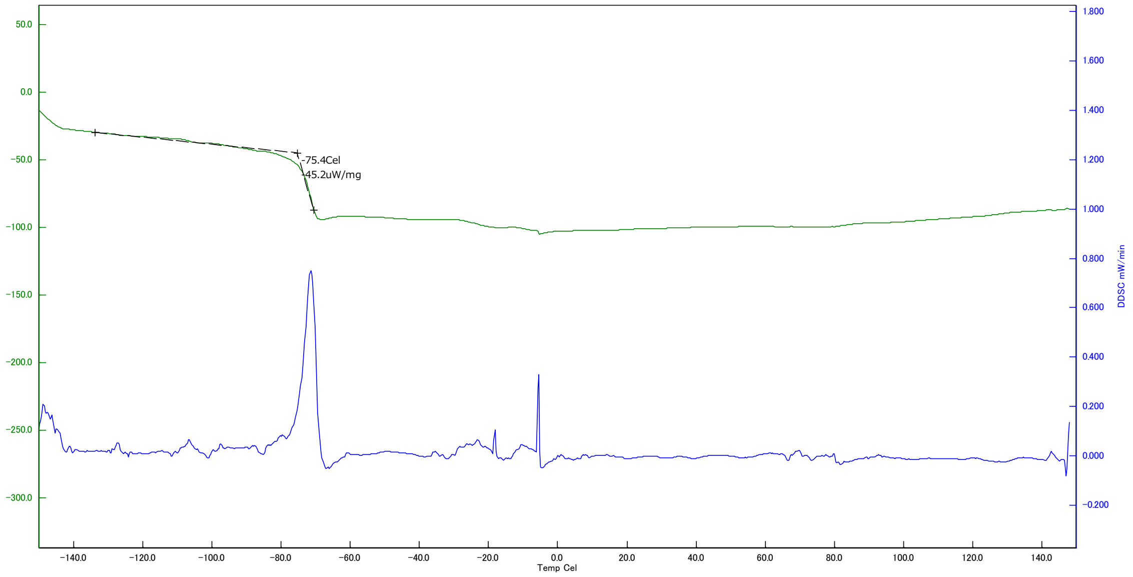

Figure S29: DSC trace (heating) of [Li(DMSO)$_3$][MsC(CN)$_2$].

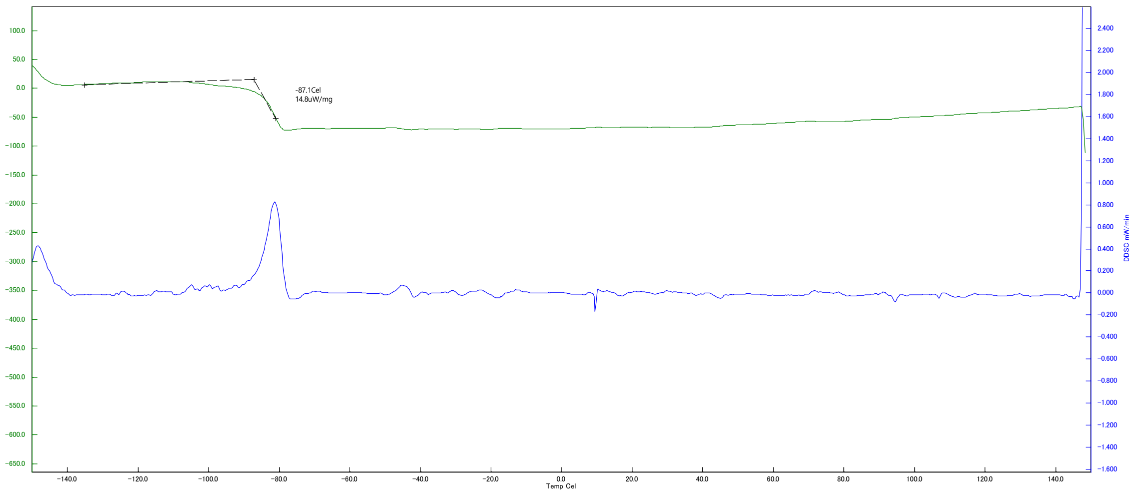

Figure S30: DSC trace (heating) of $[Li(G4)_2][MsC(CN)_2]$.

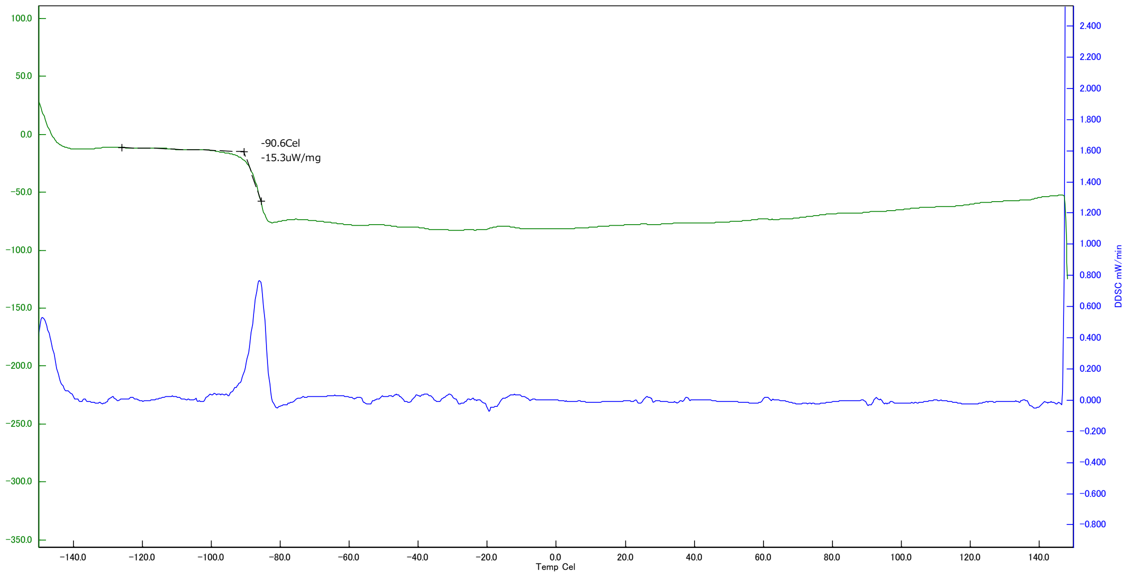

Figure S31: DSC trace (heating) of $[Li_2(G4)_2][MsC(CN)_2][TfNTf]$.

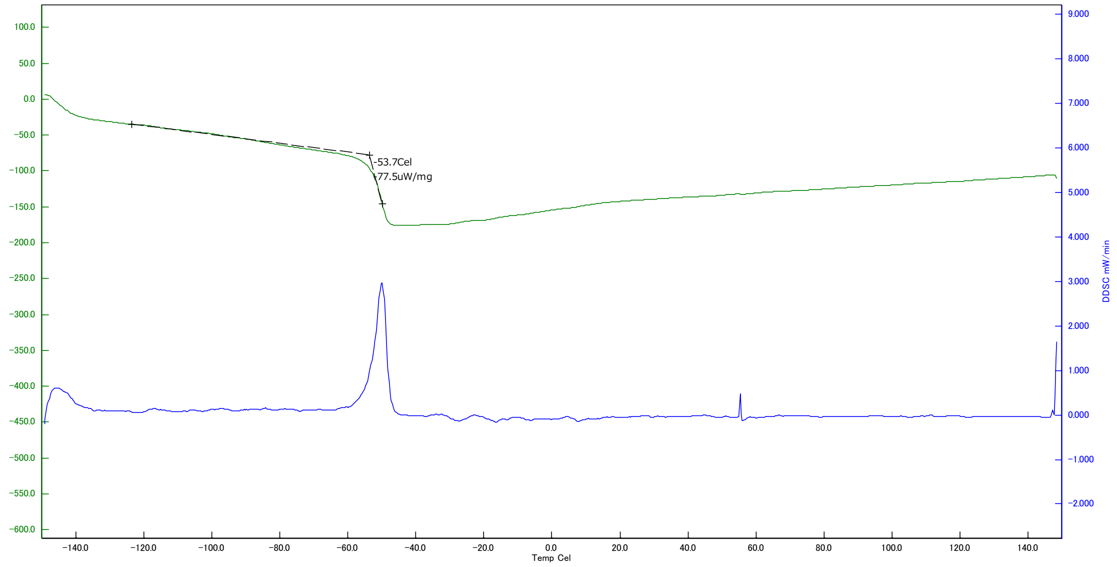

Figure S32: DSC trace (heating) of [Li(G4)][TfCHTf].

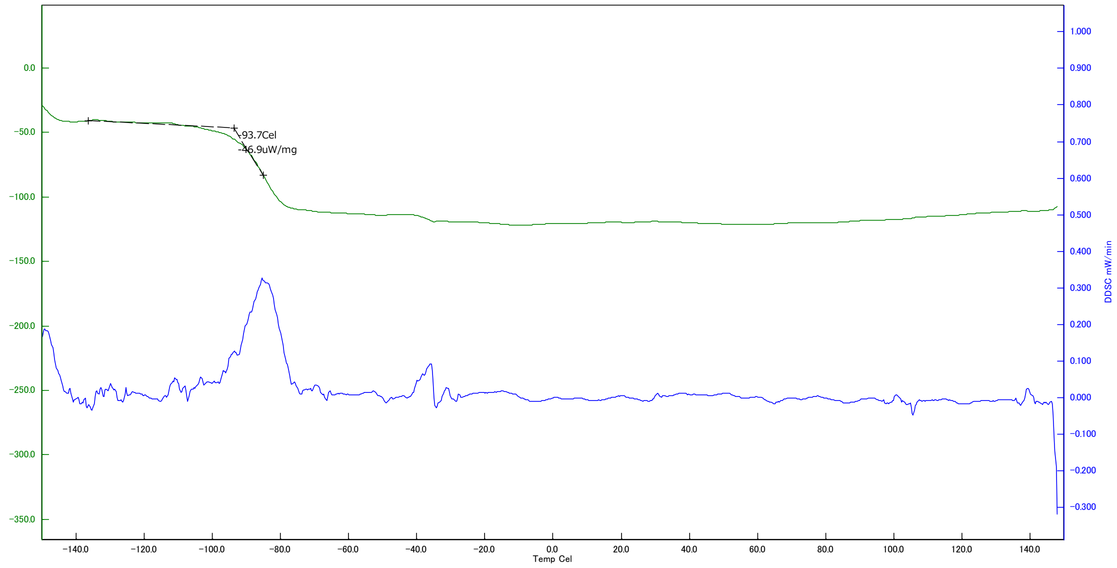

Figure S33: DSC trace (heating) of [Li(G4)][PO_2F_2].

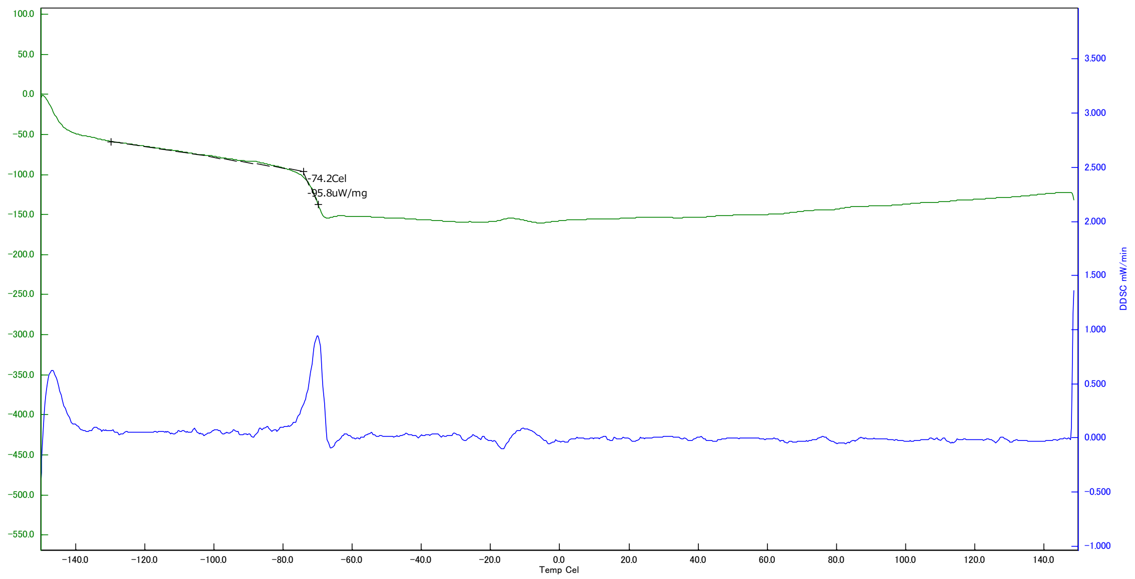

Figure S34: DSC trace (heating) of [Li(SL)$_3$][TfCHTf].

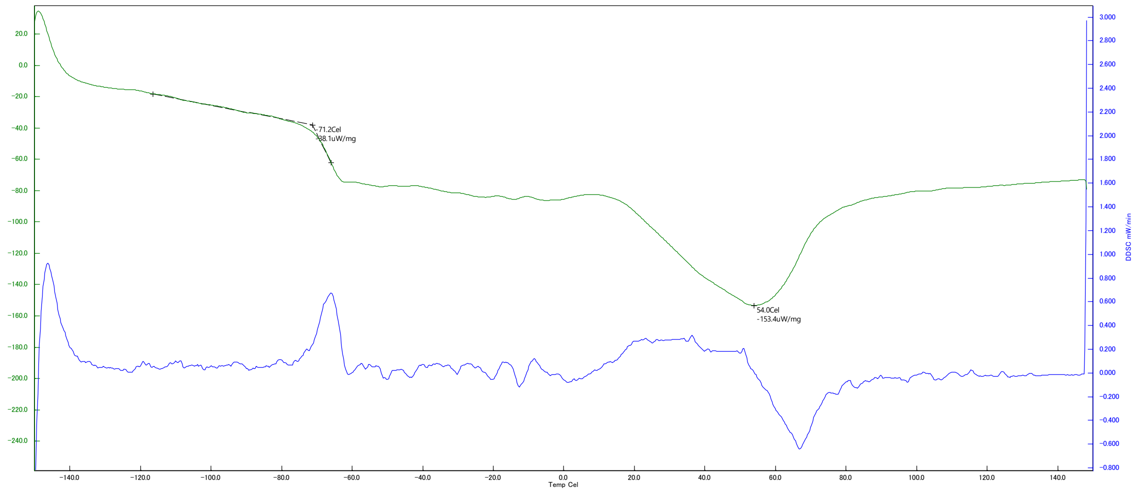

Figure S35: DSC trace (heating) of aged [Li(SL)$_2$][TfCHTf] without prior heating step.

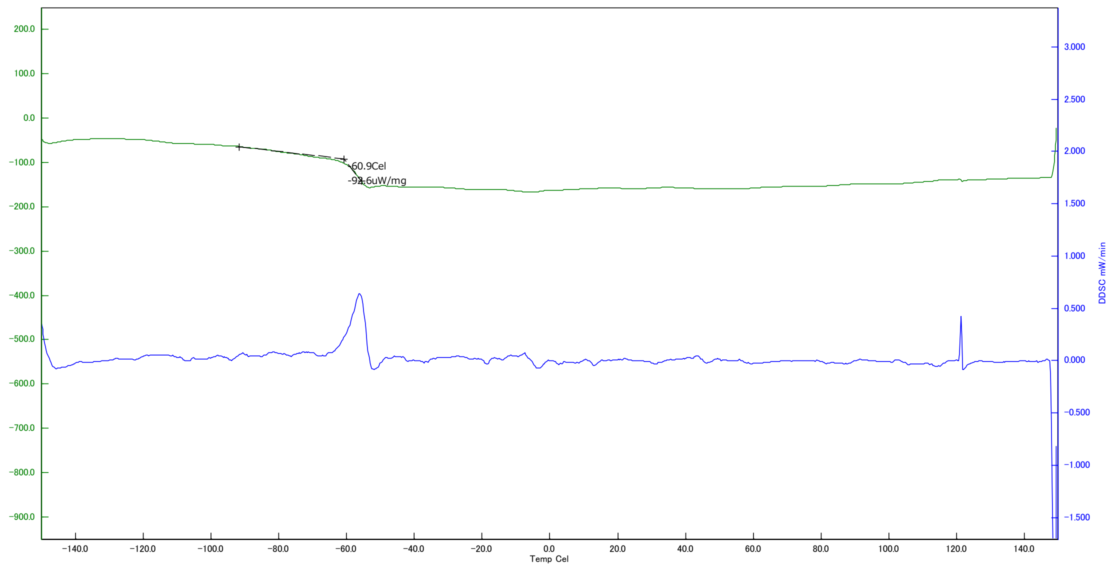

Figure S36: DSC trace (heating) of [Li(SL)$_2$][TfCHTf] with heating step and without ageing.

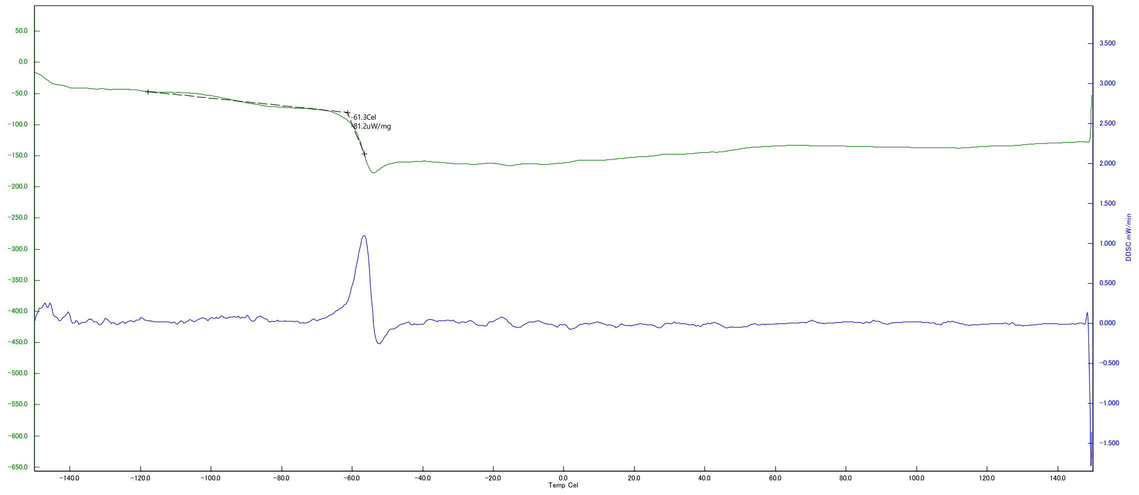

Figure S37: DSC trace (heating) of [Li(AN)$_{1.5}$][TfNCN].

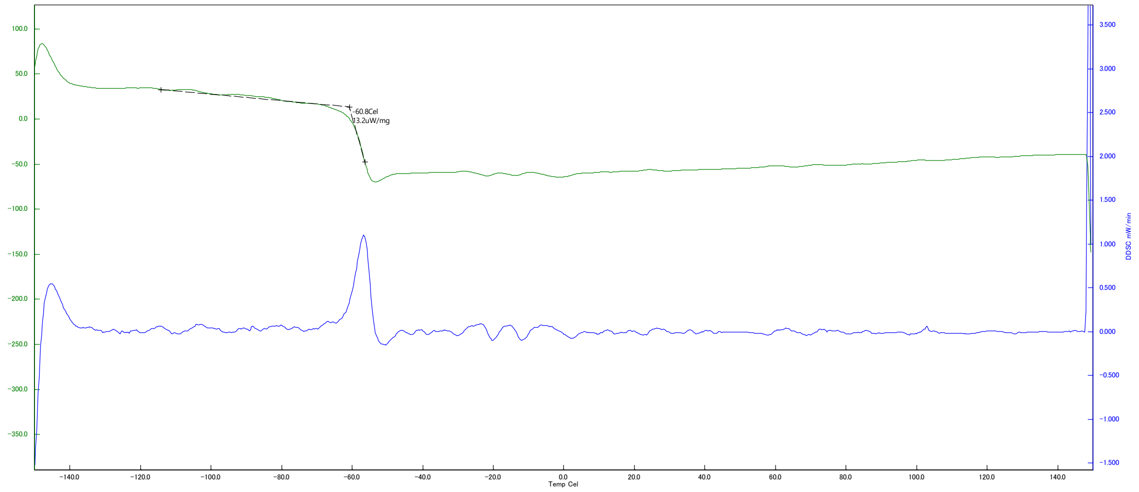

Figure S38: DSC trace (heating) of [Li(GN)$_{1.5}$][TfNCN].

4. DMTA measurements

Dynamic mechanical thermal analysis (DMTA) was carried out using a PerkinElmer (Waltham, MA, USA) DMA 8000 instrument, by means of a method already established in previous works.[14,54,55] The liquid samples were laid out into a Material Pocket supplied by PerkinElmer (30.0 mm by 14.0 mm by 0.5 mm), which is then folded in half and closed. Flexural vibration measurements were performed in the three-point bending configuration on this pocket.

The storage modulus, E', and the elastic energy dissipation, $\tan\delta$, were measured in an inert nitrogen atmosphere at variable frequencies (1, 5, 10 and 30 Hz) and a heating/cooling rate of 4 °C/min in a range between 180 and 350 K. With this setup, the stress applied on the sample is not a pure shear stress, but, due to the spatial isotropy of liquids, the measured modulus contains shear and bulk components. In addition, the stainless steel pocket contributes a baseline component to the measured modulus.[54,55]

The $\tan\delta$ and the modulus experimental data are shown in Figure S39 to Figure S44. Due to background component of the pocket, the moduli are plotted as relative variation with respect to the value at 300 K.

For neat sulfolane (Figure S39) an abrupt increase in the modulus curve and a step in $\tan\delta$, is observed for all the measured frequencies at the same temperature of about 300 K. These features indicate the occurrence of the solidification phase transition, in agreement with the literature.[53]

Samples $[Li(SL)_2][TfNTf]$, $[Li(SL)_2][TfNCN]$ and $[Li(SL)_3][TfNCN]$ displayed similar features, namely an intense $\tan\delta$ peak accompanied by a noticeable modulus variation around 200 K, likely due to the occurrence of the glass transition,[14,54,55] and at higher temperatures a broad and less intense peak accompanied by a small variation in the modulus. This peak is thermally activated since its maximum shifts at higher temperatures with increasing frequencies and it is likely due to the occurrence of a relaxation process.

The samples $[Li(SL)_2][TfNFs]$ and $[Li(SL)_2][TfNMs]$ showed a peak in the $\tan\delta$ curve accompanied by a modulus variation in correspondence of the glass transition. At higher temperatures, $[Li(SL)_2][TfNMs]$ displays some broad features, while $[Li(SL)_2][TfNFs]$ displays a shoulder on the high temperature side of the glass transition peak, which could be due to the occurrence of a relaxation process. However, this peak is not fully resolved due to the overlapping with the glass transition peak.

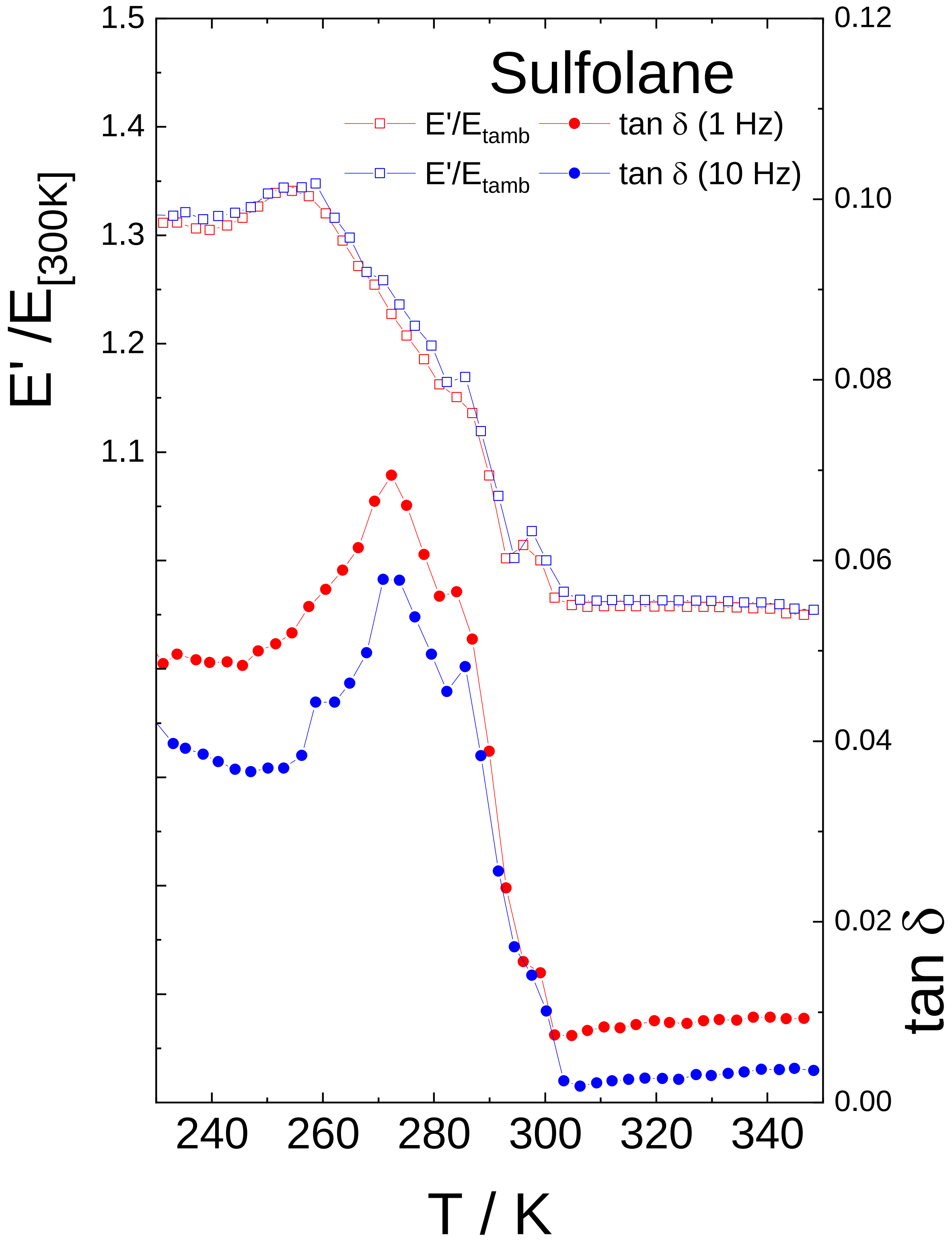

Figure S39: Experimental DMTA spectra of neat sulfolane. Empty squares are relative E’, filled circles are tan δ. Lines are a guide for the eye.

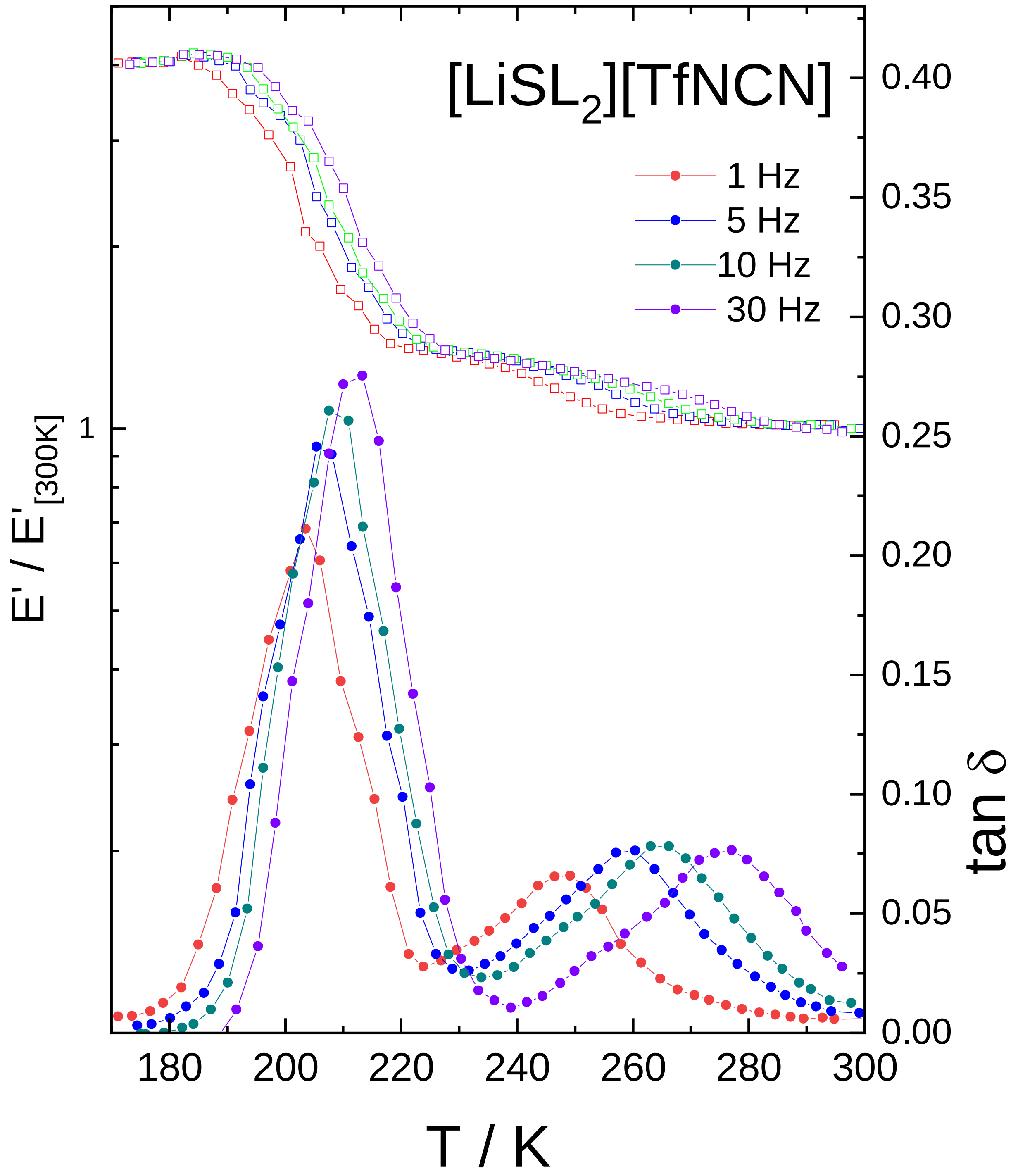

Figure S40: Experimental DMTA spectra of [Li(SL)$_2$][TfNCN]. Empty squares are relative E’, filled circles are tan δ. Lines are a guide for the eye.

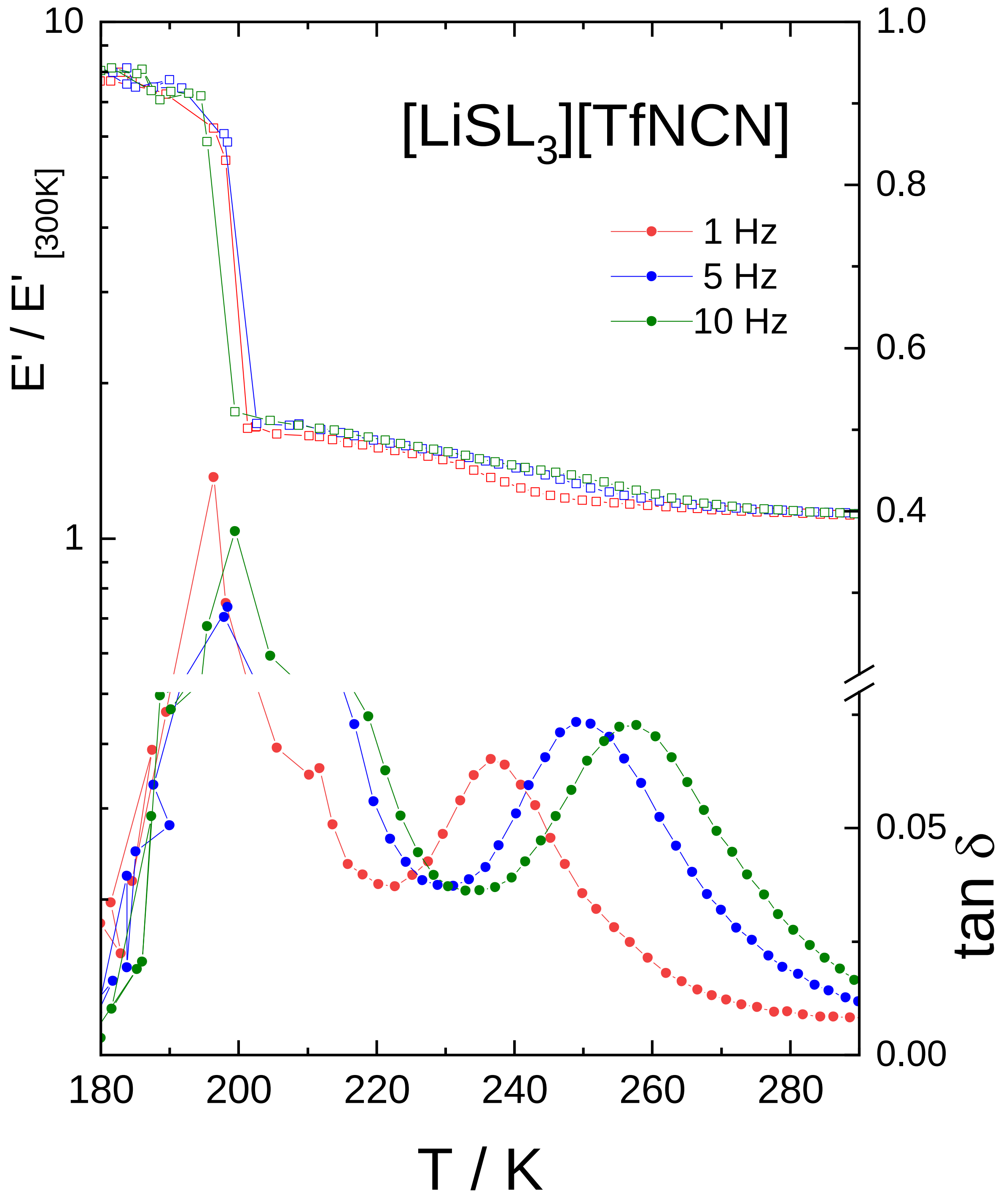

Figure S41: Experimental DMTA spectra of $[Li(SL)_3][TfNCN]$. Empty squares are relative E', filled circles are tan δ. Lines are a guide for the eye.

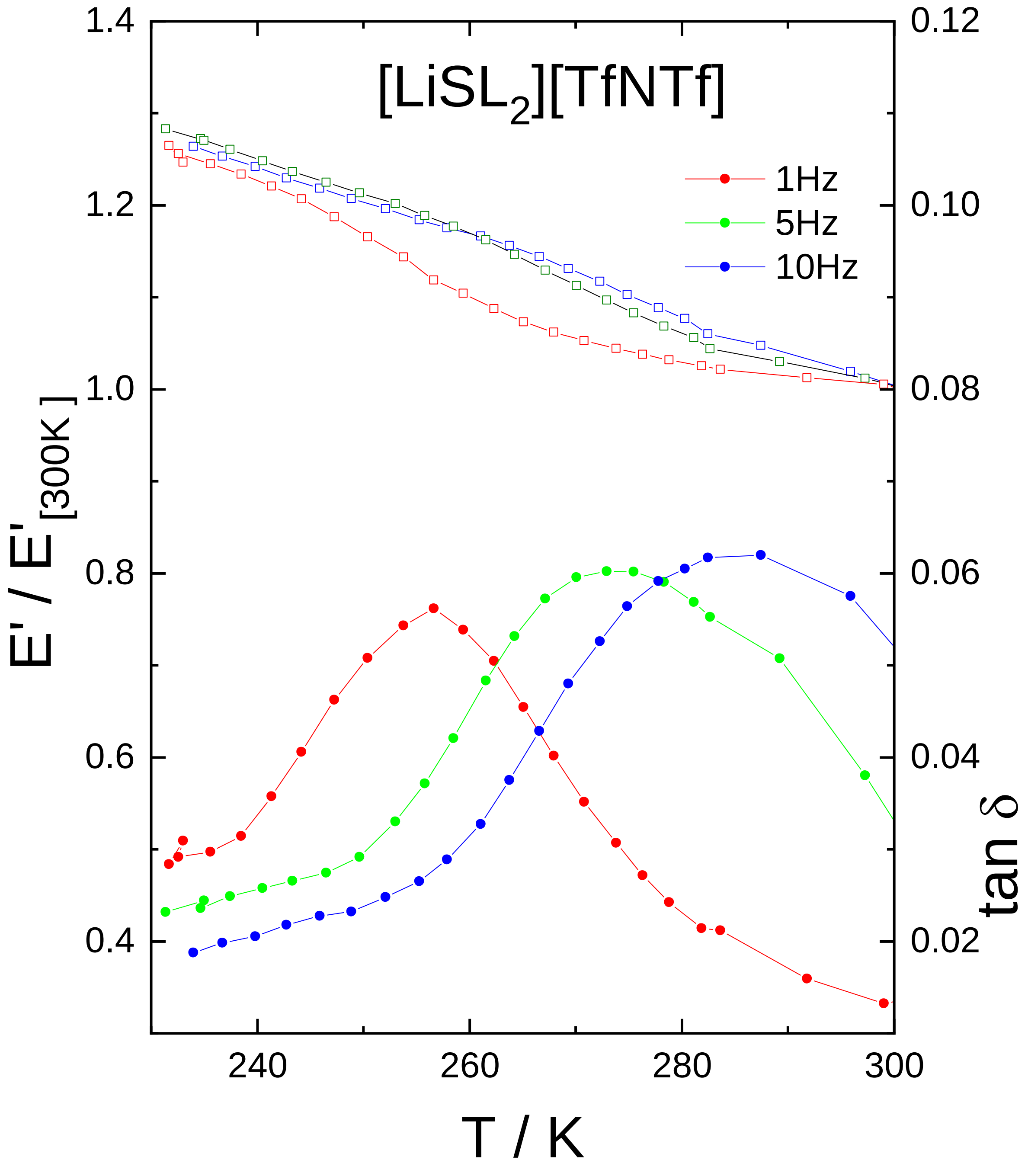

Figure S42: Experimental DMTA spectra of $[Li(SL)_2][TfNTf]$. Empty squares are relative E’, filled circles are tan δ. Lines are a guide for the eye.

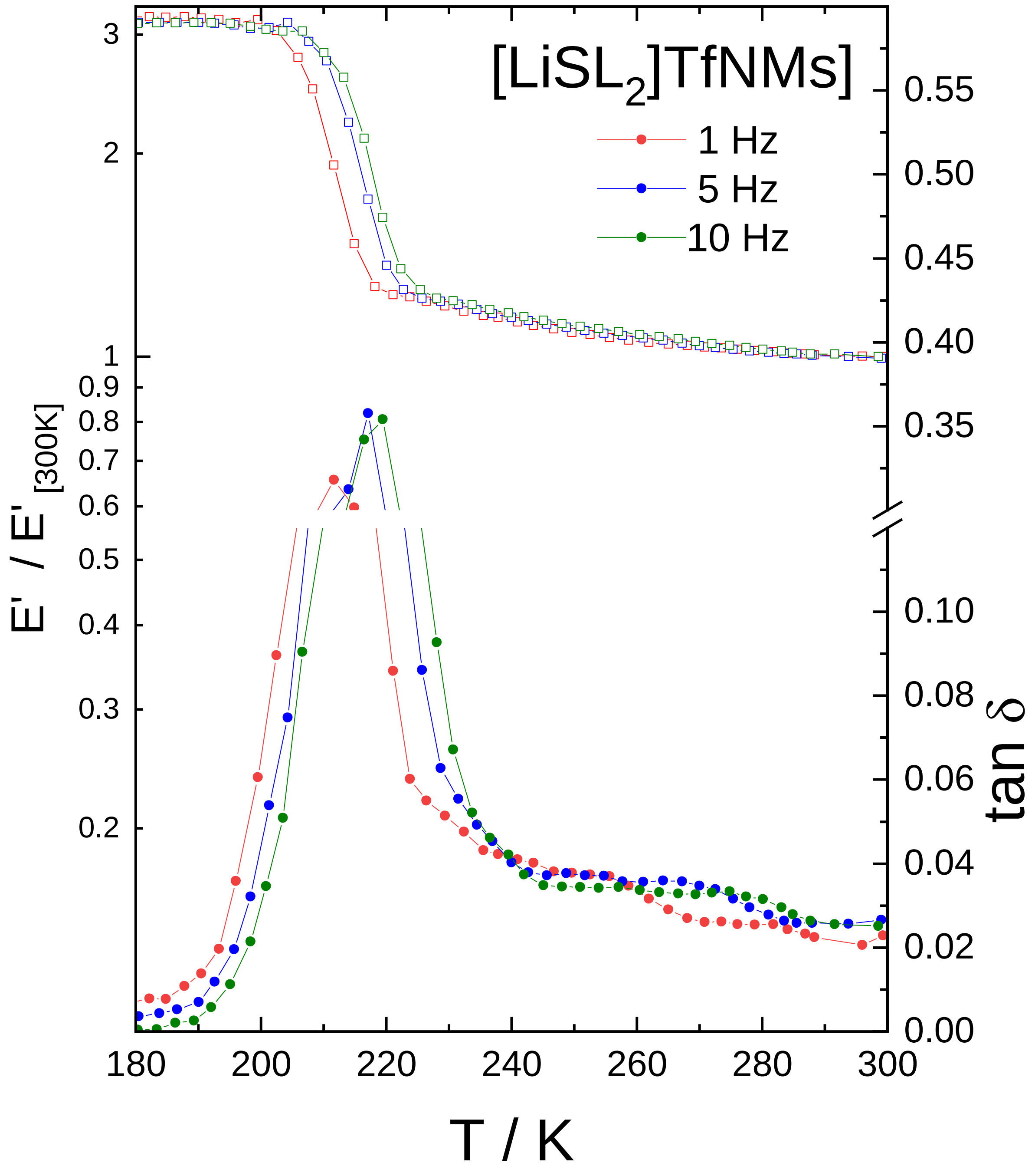

Figure S43: Experimental DMTA spectra of [Li(SL)$_2$][TfNMs]. Empty squares are relative E’, filled circles are tan δ. Lines are a guide for the eye.

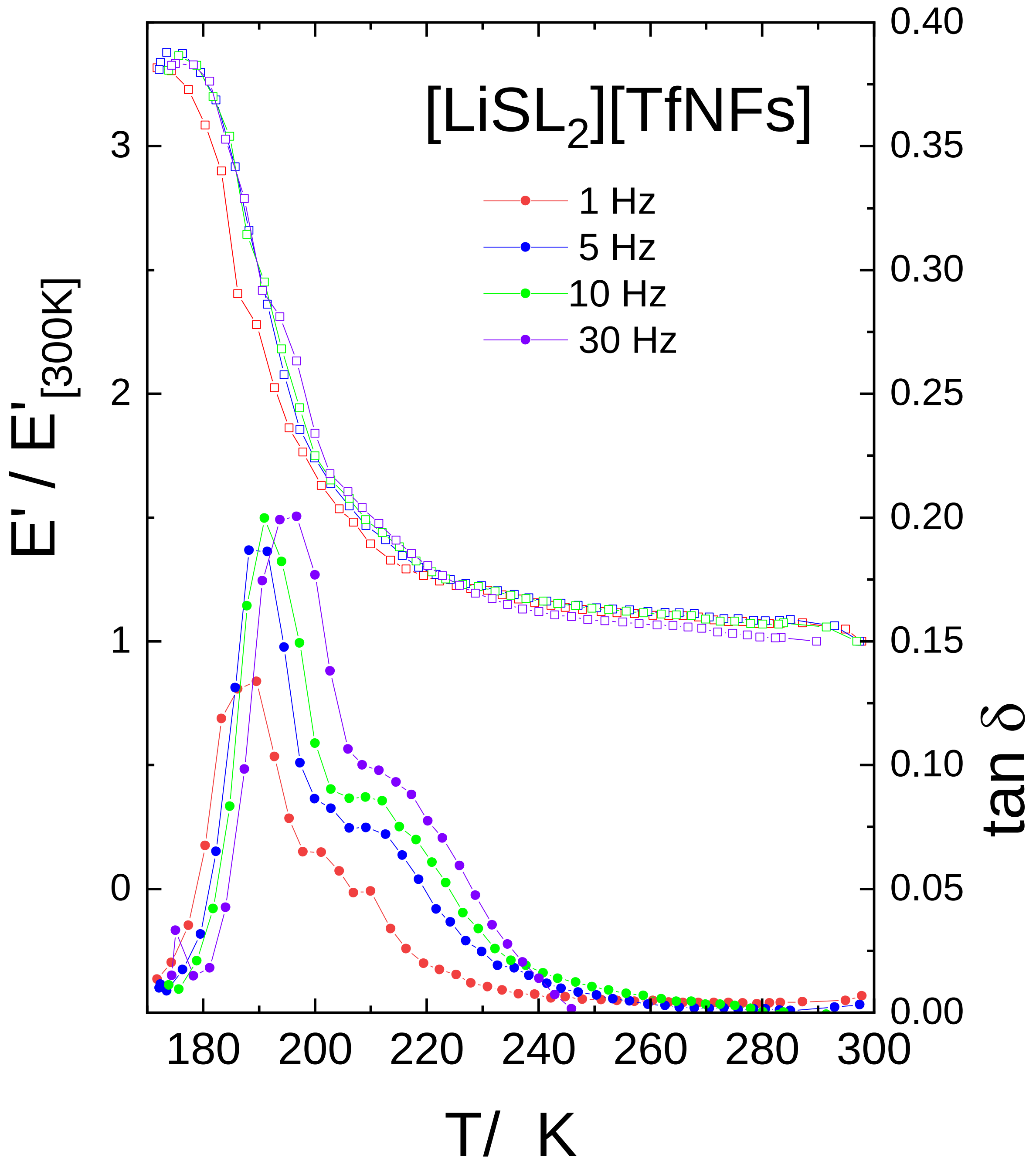

Figure S44: Experimental DMTA spectra of [Li(SL)2][TfNFs]. Empty squares are relative E', filled circles are tan δ. Lines are a guide for the eye.

The observed relaxation can be described using a simplified two state model, where the rate-limiting 'elementary reaction' is the thermally activated transition between two states. Such a state may be a well-defined minimum energy configuration or a collection of minimum energy configurations separated by small energy barriers. Within this model, the relaxing units can move between two configurations with a relaxation rate τ^{-1} by means of thermal activation. In a standard anelastic solid, the elastic energy dissipation presents a maximum when the Debye relaxation condition, $\omega\tau = 1$, is satisfied. For a single relaxation time τ, $\tan\delta$ is given by Equation (S1).

$$\tan\delta = \frac{\Delta(T)}{(\omega\tau)^{\alpha} + (\omega\tau)^{-\alpha}} \tag{S1}$$

Here, ω is the angular vibration frequency, $\Delta(T)$ is the relaxation intensity and is proportional to the concentration of the relaxing species, to the elastic modulus and to the change in the local distortion, α is the Fuoss–Kirkwood width parameter and is equal to 1 for a single time Debye relaxation (α < 1 produces broadened peaks with respect to Debye ones). The rate of this transition is characterised by a temperature dependent relaxation time τ, which is described by the VFT equation, Equation (S2).

$$\tau = \tau_0 \cdot e^{\frac{B}{T-T_0}} = \tau_0 \cdot e^{\frac{W}{R(T-T_0)}} \tag{S2}$$

where τ_0, B and T_0 are parameters. Parameter B represents the equivalent temperature of an apparent activation energy W.[54,55]

The fits of the $\tan\delta$ data corresponding to the relaxation processes for each of the three samples are reported in Figure S45. The values of the best fit parameters are reported in Table S2. For the [Li(SL)$_2$][TfNTf] sample the obtained activation energy and Vogel-Fulcher-Tammann parameters are similar to those obtained from recently reported diffusion data.[56]

Table S2: Best fit parameters obtained for the relaxation processes in the samples. In all cases the value obtained for the energy difference in the two site model was zero (*via* $\Delta(T)$, not shown here for the sake of simplicity, *cf.* literature.

	τ_0 / s	T_0 / K	α	B / K	W / meV
[Li(SL)$_2$][TfNTf]	(2.8±0.8) 10^{-8}	95±4	0.81	2503±85	216±7
[Li(SL)$_2$][TfNCN]	(9.0±3.1) 10^{-8}	161±1	0.80	1255±55	108±5
[Li(SL)$_3$][TfNCN]	(8.9±2.9) 10^{-8}	163±1	0.85	1120±58	97±5

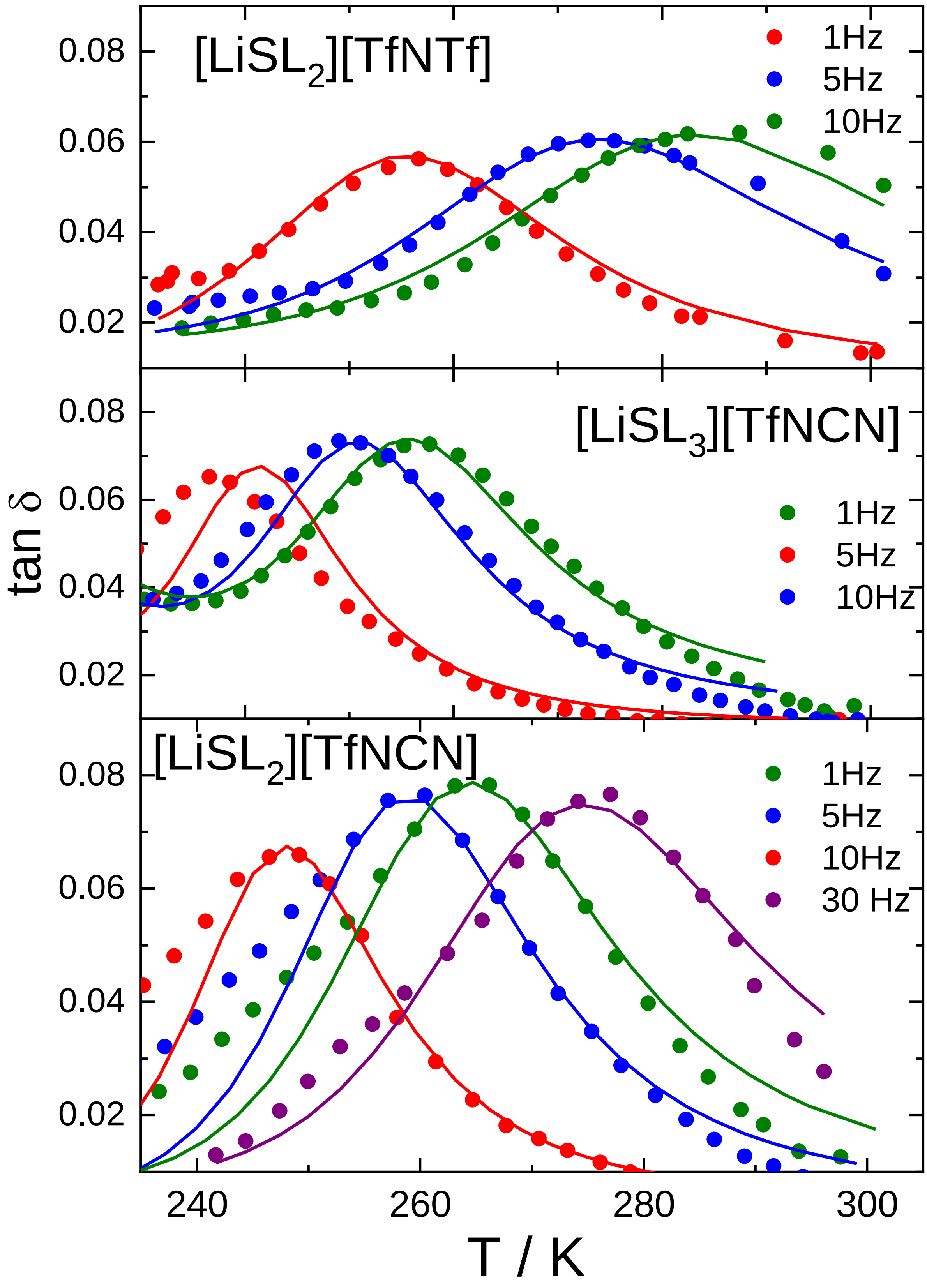

Figure S45: Summary of experimental DMTA spectra (only tan δ) and the fit using the model described in the text.

5. Thermal stability *via* TGA

Thermogravimetric Analysis was performed on an STA7200 Thermogravimetry/Differential Thermal Analyzer (Hitachi High-Tech Science Corporation). The sample chamber was flushed with nitrogen (100 mL/min) during the measurement. TGA open pans (Aluminium, 5.2 mm O.D., 2.5 mm height, 45 µL volume, 600 °C max. temperature, Hitachi High-Tech Science Corporation) were handled with clean PVDF coated tweezers (Ideal-Tek). Samples were filled into the pans in a dry chamber with dew point <−70 °C (Daikin industries LTD, model HRG-60AR). During the TGA measurement, the samples were heated from 30 °C to 550 °C with a rate of 5 °C/min. The results are summarised in Table S1, TGA traces are shown in Figure S46 to Figure S48.

Table S3: Temperatures at which 1% (T_{99}) or 5% (T_{95}) mass loss occurred.

System	T_{99} / °C	T_{95} / °C	System	T_{99} / °C	T_{95} / °C
Li[TfNCN]	308	331	[Li(SL)$_2$][TfNCN]	133	170
Li[TfNMs]	333	359	[Li(SL)$_2$][TfNMs]	116	151
Li[TfNAc]	264	284	[Li(SL)$_2$][TfNAc]	110	142

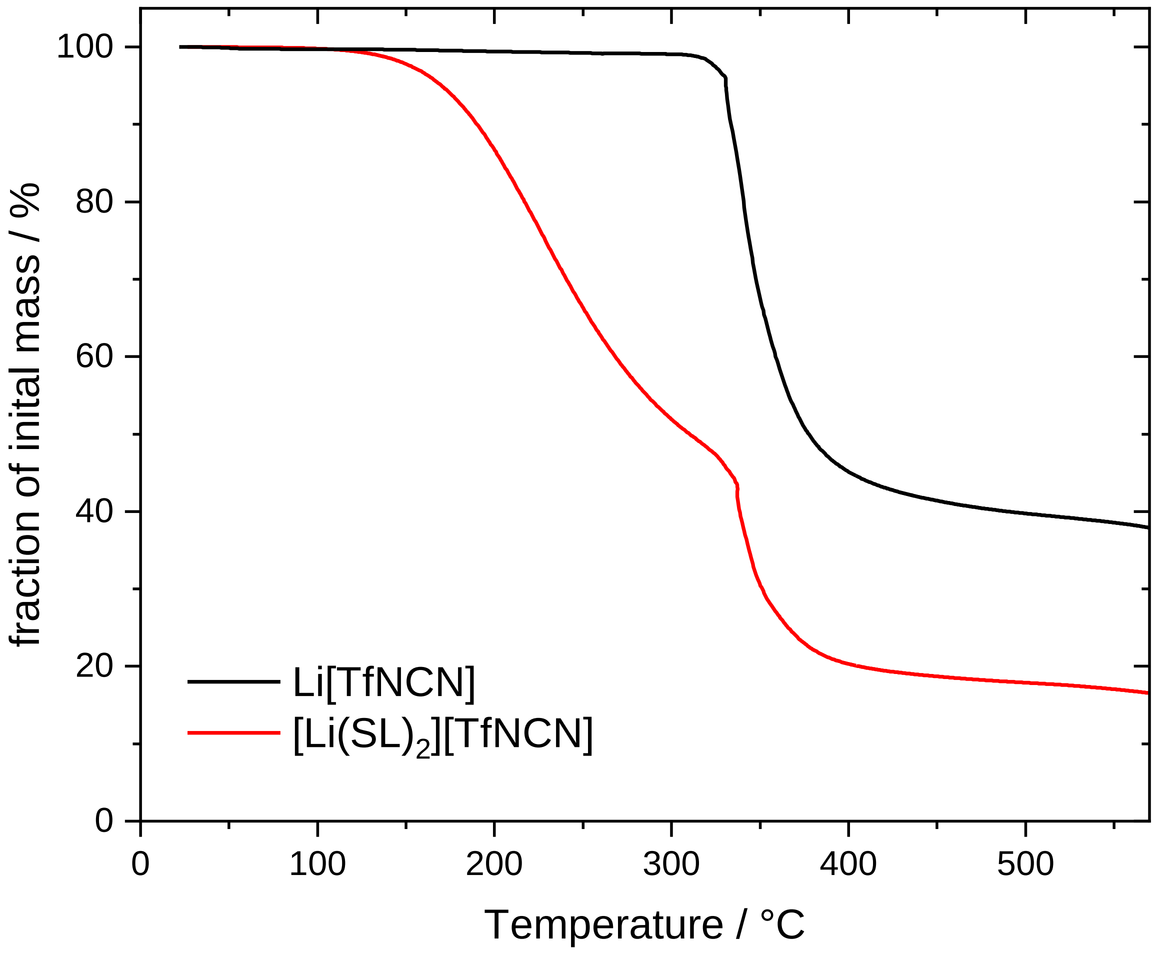

Figure S46: Thermograms of Li[TfNCN] and [Li(SL)$_2$][TfNCN].

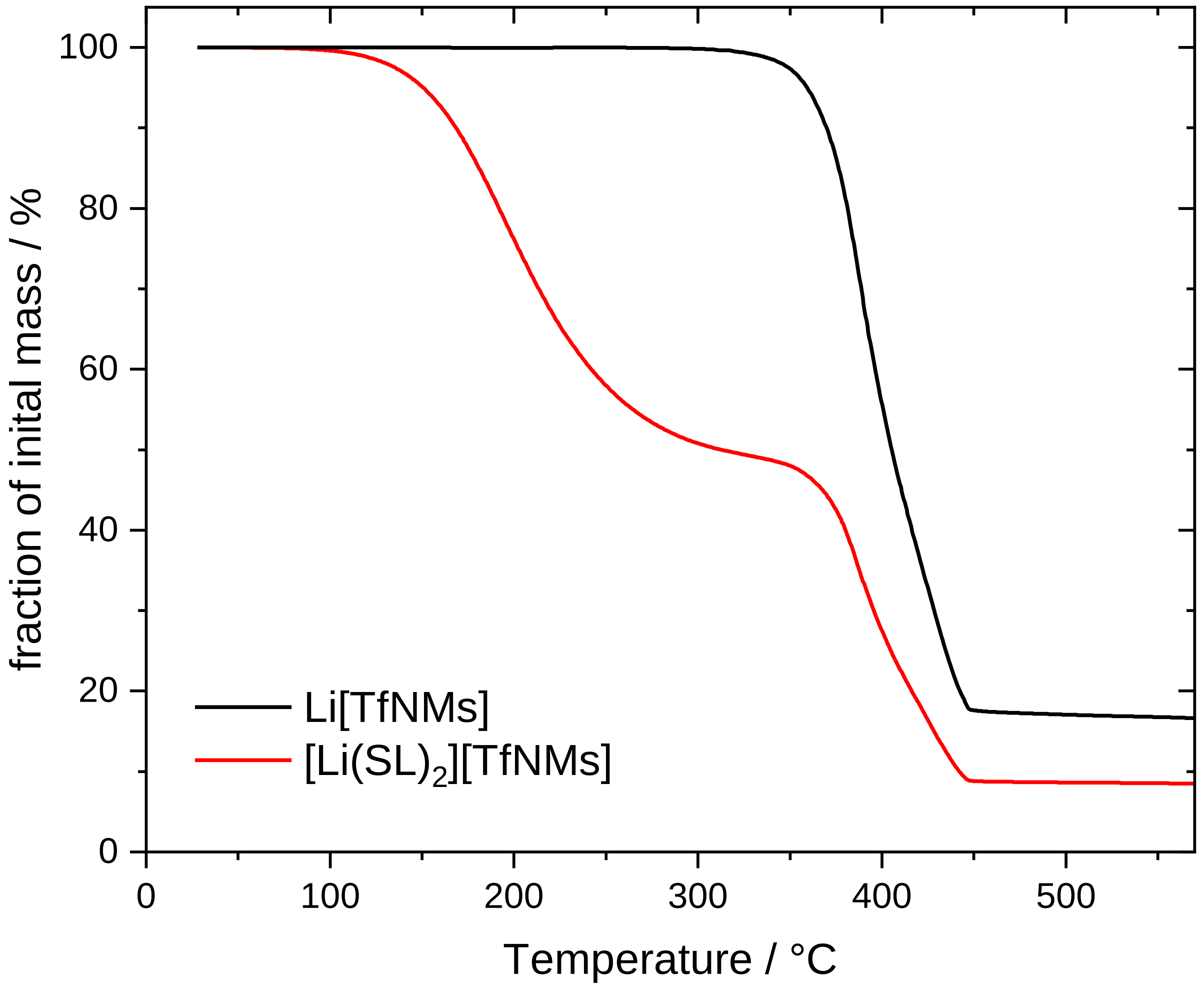

Figure S47: Thermograms of Li[TfNMs] and $[Li(SL)_2][TfNMs]$.

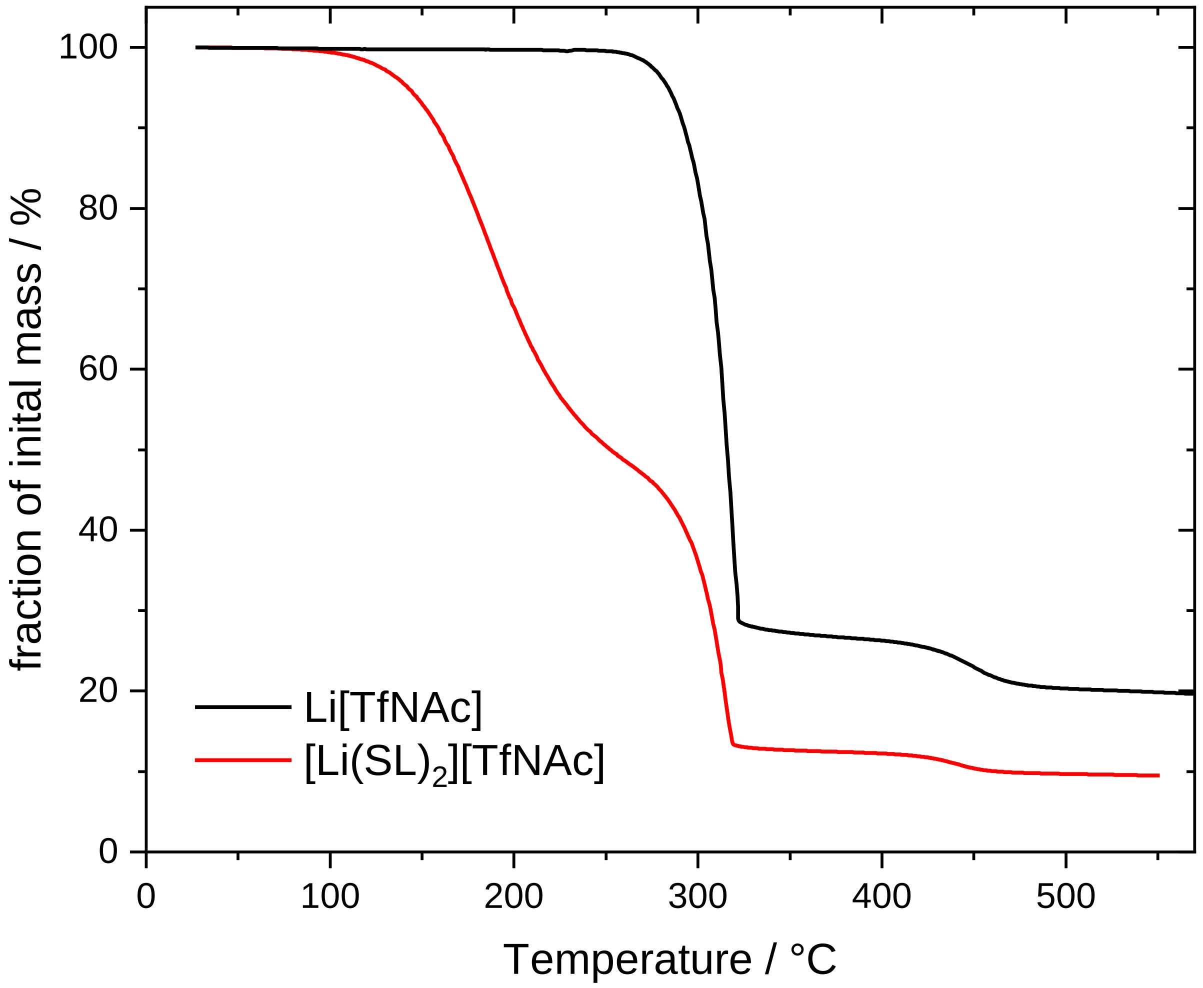

Figure S48: Thermograms of Li[TfNAc] and $[Li(SL)_2][TfNAc]$.

6. Density and Viscosity Measurements

Density ρ and viscosity η measurements were performed on a SVM 3000 Stabinger Viscometer (Anton Paar) with combined oscillating U-tube densitometer, Table S4 to Table S6. A density and viscosity reference standard (Anton Paar, Austria) was used to check the accuracy of the experimental setup. Thus, the density was reproduced to within the last measured digit (0.0001 g/cm^{-3}), while the viscosity showed an average deviation of 0.9%. Some key derived quantities are presented in Table S7 together with the masses recorded during sample preparation.

Table S4: Experimental densities in g/cm^{-3} at different temperatures. The observed average deviation across repeats from independently synthesised samples was approximately 0.0008 g/cm^{-3}.

System	Temperature				
	10 °C	20 °C	25 °C	30 °C	40 °C
[Li(SL)$_2$][TfNCN]	1.4574	1.4487	1.4444	1.4401	1.4315
[Li(SL)$_{2.2}$][TfNCN]	1.4481	1.4394	1.4350	1.4307	1.4219
[Li(SL)$_{2.4}$][TfNCN]	1.4381	1.4293	1.4249	1.4205	1.4118
[Li(SL)$_3$][TfNCN]	1.4160	1.4070	1.4026	1.3983	1.3899
[Li(SL)$_2$][TfNFs] a	1.5689	1.5588	1.5537	1.5487	1.5391
[Li(SL)$_2$][TfNFs] b	1.5676	1.5571	1.5523	1.5475	1.5377
[Li(SL)$_2$][PfNFs] a	1.6001	1.5896	1.5844	1.5791	1.5688
[Li(SL)$_2$][PfNFs] b	1.5997	1.5887	1.5836	1.5786	1.5683
[Li(SL)$_2$][TfNAc]	1.4375	1.4281	1.4233	1.4185	1.4089
[Li(SL)$_2$][TfNMs]	1.5190	1.5098	1.5053	1.5008	1.4918
[Li(SL)$_3$][TfCHTf]	1.5073	1.4972	1.4920	1.4870	1.4772
[Li(DMSO)$_3$][MsNMs]	1.3264	1.3176	1.3131	1.3086	1.2999
[Li(G4)][PO_2F_2]	1.2265	1.2170	1.2123	1.2077	1.1986
[Li(SL)$_{2.2}$][TfNMs]	1.5061	1.4970	1.4925	1.4880	1.4788
[Li(SL)$_{2.2}$][TfNAc]	1.4288	1.4193	1.4144	1.4095	1.4002

Table S5: Experimental viscosities in mPa s at different temperatures. The observed average deviation across repeats from independently synthesised samples was approximately 1% of the viscosity value.

System	Temperature				
	10 °C	20 °C	25 °C	30 °C	40 °C
[Li(SL)$_2$][TfNCN]	5461	2434	1699	1215	666
[Li(SL)$_{2.2}$][TfNCN]	3222	1514	1081	790	449
[Li(SL)$_{2.4}$][TfNCN]	1992	977	712	530	311
[Li(SL)$_3$][TfNCN]	749	403	305	235	147
[Li(SL)$_2$][TfNFs] a	1155	611	460	352	217
[Li(SL)$_2$][TfNFs] b	1131	615	464	357	221
[Li(SL)$_2$][PfNFs] a	1706	868	641	482	288
[Li(SL)$_2$][PfNFs] b	1665	873	647	488	293
[Li(SL)$_2$][TfNAc]	3529	1347	883	600	302
[Li(SL)$_2$][TfNMs]	11510	4431	2903	1962	974
[Li(SL)$_3$][TfCHTf]	1522	690	486	352	197
[Li(DMSO)$_3$][MsNMs]	2500	968	643	441	226
[Li(G4)][PO_2F_2]	700	369	277	213	131
[Li(SL)$_{2.2}$][TfNMs]	6615	2717	1830	1270	657
[Li(SL)$_{2.2}$][TfNAc]	2017	831	563	397	209

Table S6: Experimental viscosities and densities for a wider temperature range for $[Li(SL)_2][TfNCN]$ and $[Li(SL)_2][TfNTf]$.

	$[Li(SL)_2][TfNCN]$		$[Li(SL)_2][TfNTf]$	
Temperature	η / mPa s	ρ / g cm^{-3}	η / mPa s	ρ / g cm^{-3}
10 °C	5487	1.4576	2543	1.5883
20 °C	2444	1.4489	1185	1.5780
25 °C	1707	1.4446	844	1.5729
30 °C	1222	1.4403	616	1.5676
40 °C	670	1.4317	350	1.5573
50 °C	395	1.4231	213	1.5473
60 °C	248	1.4148	137	1.5376
70 °C	163	1.4066	93	1.5280
80 °C	112	1.3987		
90 °C	80	1.3909		

Table S7: Mass m, molality b, molar concentration c, and molar ratio of solvent and salt.

System	m_{salt} / g	$m_{solvent}$ / g	b / mol kg^{-1}	$c^{30\ °C}$ / mol L^{-1}	$\frac{n_{solvent}}{n_{salt}}$
$[Li(SL)_2][TfNCN]$	1.953	2.607	4.16	3.43	2.000
$[Li(SL)_{2.2}][TfNCN]$	3.428	5.030	3.79	3.22	2.198
$[Li(SL)_{2.4}][TfNCN]$	3.699	5.943	3.46	3.03	2.407
$[Li(SL)_3][TfNCN]$	3.917	7.844	2.77	2.59	3.000
$[Li(SL)_2][TfNFs]$ a	2.633	2.666	4.17	3.25	1.998
$[Li(SL)_2][TfNFs]$ b	3.162	3.206	4.16	3.24	2.000
$[Li(SL)_2][PfNFs]$ a	4.866	4.071	4.16	2.99	1.999
$[Li(SL)_2][PfNFs]$ b	3.543	2.966	4.16	2.99	2.000
$[Li(SL)_2][TfNTf]$	4.794	4.020	4.15	2.97	2.003
$[Li(SL)_2][TfNAc]$	2.616	3.193	4.16	3.24	2.002
$[Li(SL)_2][TfNMs]$	5.129	5.288	4.16	3.17	2.000
$[Li(SL)_3][TfCHTf]$	3.671	4.635	2.77	2.30	3.006
$[Li(DMSO)_3][MsNMs]$	5.110	6.690	4.26	3.16	3.002
$[Li(G4)][PO_2F_2]$	3.994	8.226	4.50	3.66	1.000
$[Li(SL)_{2.2}][TfNMs]$	2.591	2.939	3.78	2.99	2.200
$[Li(SL)_{2.2}][TfNAc]$	2.844	3.831	3.77	3.05	2.209

7. Lithium transference from Symmetric Li-Li Coin Cell

Coin cells were assembled as shown in Figure S49 and hermetically sealed under argon in a glovebox with ≤0.5 ppm water content (VAC vacuum atmospheres company, model OMNI-LAB). Circular pieces of lithium with 16 mm diameter were cut from lithium foil (Honjo Metal Co., Ltd., Japan) using a lithium punch tool (PTFE punch/die, Hohsen). Circular pieces of GA-55 (ADVANTEC) glass fibre filter paper with 17 mm diameter were cut outside the glovebox using a steel punch tool and transferred to the glovebox after drying overnight in a glass tube oven at 40°C. The symmetric coin cells were assembled using a R2032-type coin cell kit (case, cap, gasket, spacer, wave washer, SUS316L stainless steel parts, provided by Hohsen) and the GA-55 separator soaked with 80 µL of electrolyte sandwiched between two pieces of lithium foil. The cells were sealed using an automatic coin cell crimper (Keihinrika Industry Co. LTD, Model HSACC-11) and aged at 30°C for 24 h before measurement. A thermostatic chamber (LS-5N BioChamber, Nippon Blower Co. LTD) was used to achieve constant temperature during ageing and measurement.

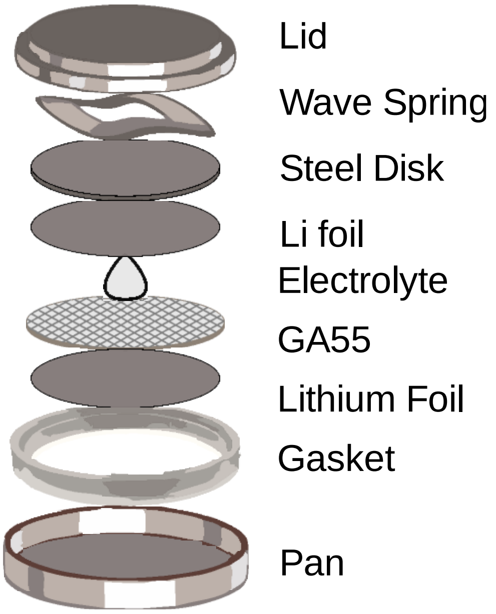

Figure S49: Schematic view showing the coin cell components and the order in which they were assembled for the measurement under anion-blocking conditions using the GA55 type separator.

The measurements were performed using a ModuLab XM potentiostat (Solartron Analytical) as follows.

1) Open circuit measurement for 30 min.
2) Potentiostatic impedance measurement (at 0 V DC vs. OCV) from 1 MHz to 100 mHz in logarithmic steps, 10 points per decade, 10 mV RMS amplitude.
3) Open circuit measurement for 10 min.
4) Potentiostatic impedance as in 2)
5) Open circuit measurement for 10 min.
6) Potentiostatic impedance as in 2), but from 1 MHz to 0.1 mHz.
7) Open circuit measurement for 30 min.
8) Potentiostatic impedance as in 2). This measurement was used to determine $R_0 = R1_0 + R2_0$ and R_{bulk}.
9) Potentiostatic polarisation experiment (V_{DC} = 10 mV) for 2 h to determine I_0, I_{SS}
10) Potentiostatic impedance as in 2) at the DC level from the previous step 9). This measurement was used to determine $R_{SS} = R1_{SS} + R2_{SS}$ and to check for the absence of changes in R_{bulk}.

An example for the polarisation curve for two electrolytes with different transference number is shown in Figure S50.

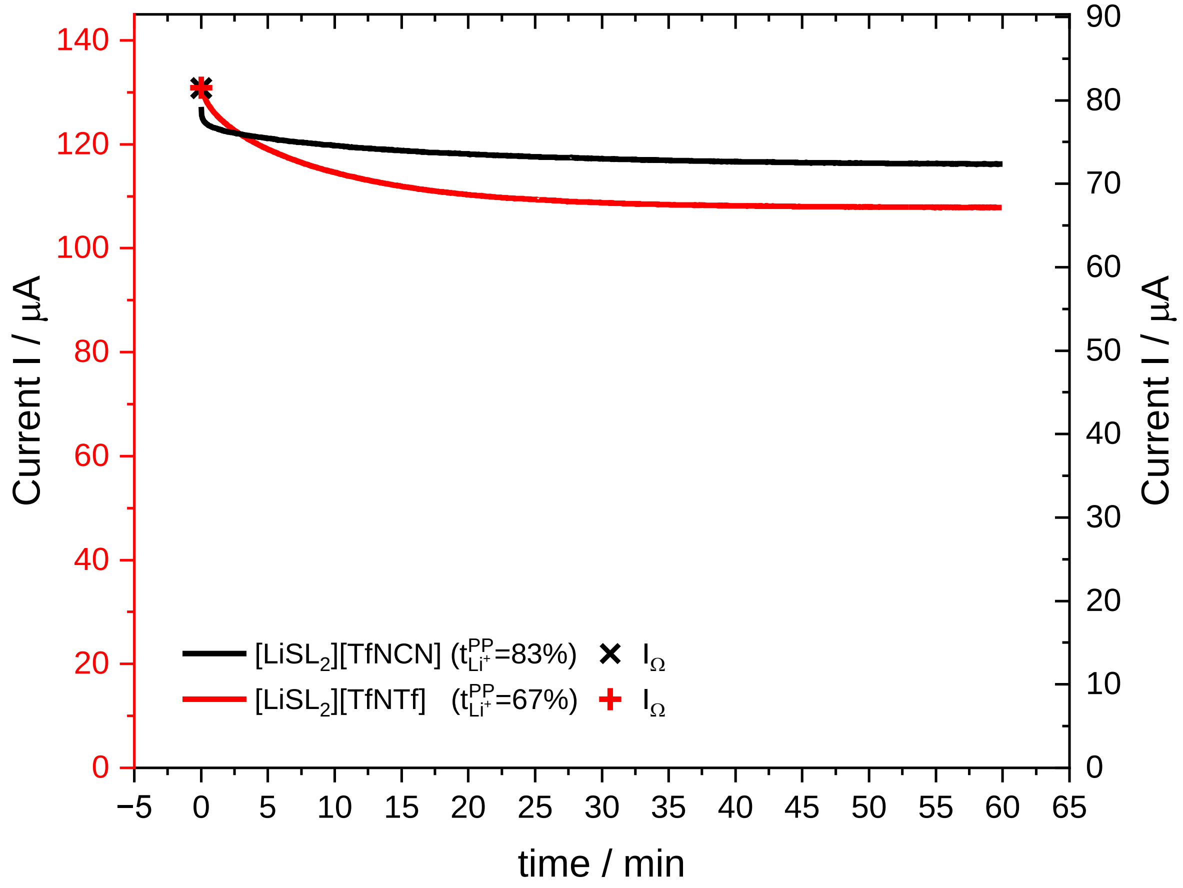

Figure S50: Representative polarisation curves for two different electrolytes. The scales are chosen so that the initial currents I_Ω overlap.

Impedance data were fitted with the equivalent circuit in Figure S51 using the ZView software package, version 4.0g (Scribner Associates, Inc.) in combination with the XM-studio ECS software package, version 3.4 (Solartron Analytical).

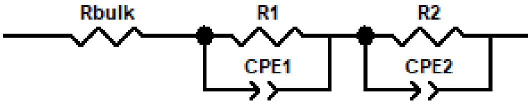

Figure S51: Equivalent circuit used to fit Impedance spectra.

Thus, the transference number can be obtained using Equation (S3).

$$t_{Li^+}^{PP} = \frac{I_{SS}(V_{DC} - I_0 R_0)}{I_0(V_{DC} - I_{SS} R_{SS})} \tag{S3}$$

However, the transference number as determined using Equation (S3) suffers from the limited accuracy of the measurement of I_0. Hence, it is preferable to calculate the initial current I_Ω via Ohm's law, Equation (S4).

$$I_\Omega = \frac{V_{DC}}{R_0 + R_{bulk}} \tag{S4}$$

This leads to the transference number t_{Ω,Li^+}^{PP} in Equation (S5), which is the one referred to in the main manuscript unless specified otherwise.

$$t_{\Omega,Li^+}^{PP} = \frac{I_{SS}(V_{DC} - I_0 R_0)}{I_\Omega(V_{DC} - I_{SS} R_{SS})} \tag{S5}$$

In addition, the very low frequency transference number $t_{Li^+}^{VLF}$ could in some cases be obtained from the impedance measurement in step 6) described above as shown in Equation (S6).[57]

$$t_{Li^+}^{VLF} = \frac{R_{bulk}}{R_{Ws} + R_{bulk}} \tag{S6}$$

Here, R_{Ws} is the resistance from a Warburg Short element added in series to the equivalent circuit shown in Figure S51 to fit the low frequency impedance data.

The results are summarised in Table S8. In general, the differences across the three transference numbers are small, and the literature value $t_{\Omega,Li^+}^{PP} = 68\%$ could be reproduced.[58]

Table S8: Results from the symmetric Li-Li coin cell experiments. The resistances are given for one randomly selected cell to demonstrate that the changes during the polarisation experiment were small as is required to obtain reliable results. In addition, the bulk resistance serves as an approximate indicator for bulk conductivity. Unless mentioned otherwise, three repeats were performed. The standard deviations are given in brackets.

	Resistances / Ω					Lithium transference numbers		
	$R1_0$	$R2_0$	R_{bulk}	$R1_{SS}$	$R2_{SS}$	t^{PP}_{Ω,Li^+}	$t^{VLF}_{Li^+}$	$t^{PP}_{Li^+}$
[Li(SL)$_2$][TfNTf]	32	8	37	32	7	67(2)%	68.5(5)%	68(2)%
[Li(SL)$_2$][TfNFs]	17	8	18	17	6	60(1)%		66(7)%
[Li(SL)$_2$][PfNFs]	20	7	25	20	6	63.0(5)%		68(4)%
[Li(SL)$_1$][TfNCN]	106		2573	107		95(4)%	94(6)%	95(2)%
[Li(SL)$_2$][TfNCN] [a]	36	10	76	35	9	83(2)%	83(9)%	83(3)%
[Li(SL)$_2$][TfNCN] [b]	4	2	23	4	2	79(3)%	78(2)%	94(11)%
[Li(SL)$_{2.2}$][TfNCN]	37	10	73	37	8	82(1)%		81(2)%
[Li(SL)$_{2.4}$][TfNCN][c]	38	10	48	37	9	79(2)%		80(4)%
[Li(SL)$_3$][TfNCN] [c]	36	10	28	34	7	69(2)%		74(1)%
[Li(SL)$_2$][TfNMs] [a]	40	18	104	40	15	87(3)%		86(2)%
[Li(SL)$_2$][TfNAc]	57	22	134	57	19	89(2)%	88(2)%	87.4(3)%
[Li(SL)$_2$][TfCHTf] [d]	56	14	86	55	11	78%		83%
[Li(SL)$_3$][TfCHTf] [c]	43	9	30	42	9	69(2)%		67(8)%
[Li(G4)][TfNCN]	55	11	33	55	10	22(1)%	21.8(1)%	21(2)%
[Li(G4)][TfNAc]	48	13	66	47	12	60(9)%	66(1)%	60(10)%
[Li(G4)][6cPFSI]	84		37	84		3.2(1)%	3.33(5)%	9(6)%
[Li(G4)][5cPFSI]	79	20	18	81	19	3.0(1)%	2.9(1)%	1(2)%
[Li(G4)][PO_2F_2]	208	13	371	208	9	89.2(5)%		90(1)%
[Li(G4)][TfNMs]	53	17	56	54	15	34(1)%	34(1)%	33(9)%
[Li(G4)][TfN5]	54	19	209	54	17	85(2)%	84(1)%	82.7(2)%
[Li(G4)][TfCHTf]	92	13	30	92	12	8.8(2)%	9.1(3)%	14(5)%
[LiGN$_{1.5}$][TfNCN]	37	10	162	37	9	84(2)%		84(1)%
[LiAN$_{1.5}$][TfNCN]	50	17	205	50	15	83(2)%		83(5)%
[LiMsC(CN)$_2$] 1 M in Sulfolane [c]	50	7	15	48	6	60(4)%		66(2)%
[Li(G4)$_2$][MsC(CN)$_2$] [a,c]	62	7	19	61	6	55(4)%	65(1)%	60(5)%
[Li$_2$(G4)$_2$][MsC(CN)$_2$][TfNTf] [c]	56	8	10	55	7	36(2)%	43.9(4)%	40(6)%
[Li(DMSO)$_3$][MsC(CN)$_2$]	48	10	23	48	9	27(1)%	28(1)%	31(1)%
[Li(DMSO)$_3$][MsNMs]	56	11	66	55	9	51(1)%	53(2)%	53(6)%

[a] 6 cells measured in total

[b] measurement at 60 degrees

[c] reduction current discernible in some cells

[d] only one cell, assembled with supercooled sample.

8. Ionic Conductivity

8.1. Conductivity cell (ex situ)

Conductivity cells equipped with two platinised platinum electrodes (CG-511B, TOA Electronics) were calibrated using 0.01 M KCl solution (Kanto Chemical Co. Inc, cica reagent) at 25°C. After thorough cleaning and drying, the conductivity cells were filled and sealed under argon in a glovebox with ≤0.5 ppm water content (VAC vacuum atmospheres company, model OMNI-LAB). SUS316 cylindrical stainless steel conductivity cells with 8.4 mm nominal internal diameter and 5 mm nominal internal height (MICLAB inc., Yamato, Kanagawa, Japan) were used in similar fashion to conduct repeat measurement with limited sample volume. In this case, both steel electrodes were polished for 9 min each using deagglomerated alumina suspensions (SANKEI Co., Ltd.) with decreasing particle sizes of 1 µm, 0.3 µm, 0.05 µm (three minutes for each particle size and electrode surface).

Conductivity was measured at 30°C. The temperature for calibration and measurement was kept constant using a thermostatic chamber (Model SU-242, ESPEC).

Impedance spectra were recorded using a VSP-300 potentiostat (BioLogic SAS). After a 25 min waiting period, Impedance spectra were recorded (at 0.0 V DC) from 500 kHz to 1 Hz in logarithmic steps, 10 points per decade, with 100 mV amplitude (≈70.7 mV RMS), averaging 10 measurements per frequency. Three impedance spectra were recorded in this manner after each other, and checked for the absence of drift. The bulk resistance R_{bulk} was then taken as the touchdown in the Nyquist plot as is common practise. The specific conductivity was obtained using Equation (S7).

$$\sigma = \frac{d}{R_{bulk}A} = \frac{K}{R_{bulk}} \qquad \text{(S7)}$$

Here, d is the distance between electrodes and A their area. For the *ex-situ* measurements, the calibration constant K was determined by measuring 0.01 M KCl rather than relying on the cell geometry. The results of the measurement including the concentration c and the molar conductivity $\Lambda = \sigma/c$ are shown in Table S9 and Table S10.

Table S9: Conductivity data, measured with Pt electrode conductivity cell.

	σ / mS cm^{-1}	c / mol L^{-1}	Λ / mS mol^{-1} cm^{-1}
$[Li(SL)_2][PfNFs]$	0.554	2.99	0.185
$[Li(SL)_2][TfNFs]$	0.668	3.24	0.206
$[Li(SL)_2][TfNMs]$	0.133	3.17	0.042
$[Li(SL)_2][TfNAc]$	0.106	3.24	0.033
$[Li(SL)_2][TfNCN]$	0.183	3.43	0.053
[Li(G4)][TfNAc]	0.207		
$[Li(SL)_3][TfNCN]$ 11.3 degrees	0.199	2.59	0.077
$[Li(SL)_3][TfNCN]$	0.461	2.62	0.176
$[Li(G4)][PO_2F_2]$	0.035	3.66	0.010
$[Li(SL)_3][TfCHTf]$	0.442	2.30	0.192
$[Li(DMSO)_3][MsNMs]$	0.274	3.16	0.087
Li[TfNCN] 1 M in EC:DMC 1:1	4.124	1.00	4.124

Table S10: Conductivity data, measured with SUS conductivity cell.

	σ / mS cm^{-1}	c / mol L^{-1}	Λ / mS mol^{-1} cm^{-1}
[Li(SL)$_2$][TfNCN] a	0.171	3.43	0.050
[Li(SL)$_2$][TfNCN] b	0.166	3.43	0.049
[Li(SL)$_2$][TfNCN] c	0.172	3.43	0.050
[Li(SL)$_2$][TfNMs]	0.131	3.17	0.041
[Li(SL)$_2$][TfNAc] a	0.100	3.24	0.031
[Li(SL)$_2$][TfNAc] b	0.097	3.24	0.030
[Li(SL)$_1$][TfNCN]	0.006		

8.2. Coin Cell (in situ)

The conductivity within the GA55 separator was measured in a coin cell under blocking conditions using symmetric stainless-steel electrodes assembled in a glovebox with ≤0.5 ppm water content (VAC vacuum atmospheres company, model OMNI-LAB), Figure S52. Impedance spectra were recorded using a ModuLab XM potentiostat (Solartron Analytical). After a 15 min waiting period, Impedance spectra were recorded (at 0.0 V DC *vs.* OCV) from 1 MHz to 0.1 Hz in logarithmic steps, 10 points per decade, with 5 mV. At least two impedance spectra were recorded in this manner after each other including the initial waiting period, and checked for the absence of drift. The bulk resistance R_{bulk} was then taken as the touchdown in the Nyquist plot as is common practise, *cf.* Equation (S7). The measurement was performed in a thermostatic chamber (LS-5N BioChamber, Nippon Blower Co. LTD) at 30°C.

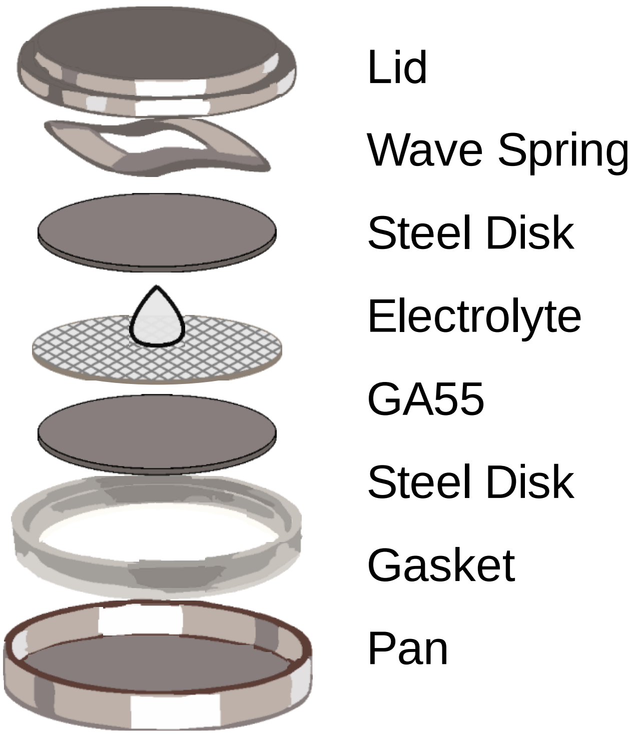

Figure S52: Schematic view showing the coin cell components and the order in which they were assembled for the measurement under blocking conditions.

The purpose of this measurement is to calculate the tortuosity τ. First, the cell constant is calculated from the cell geometry *via* Equation (S8), also *cf.* Equation (S7), with the separator thickness of 0.21(1) mm and the steel disk diameter of 16 mm.

$$K = \frac{d}{A} = \frac{0.21\ \mathrm{mm}}{201\ \mathrm{mm}^2} = 0.0104(5)\ \mathrm{cm}^{-1} \tag{S8}$$

Across several coin cells, the bulk resistances and conductivities of the soaked separator (without corrections for separator tortuosity and porosity) shown in Table S11 were obtained.

Table S11: Correction constant N_m from *in situ* and *ex situ* measured conductivities.

	R_{bulk} / Ω	$\sigma^{*}_{in\ situ}$ / mS cm^{-1}	$\sigma_{ex\ situ}$ / mS cm^{-1}	N_m	$\sigma_{in\ situ}$ / mS cm^{-1}
$[Li(SL)_2][TfNCN]$	74.9(9)	0.139(7)	0.173(4)	1.24(7)	0.174(1)
$[Li(SL)_2][TfNMs]$	102(1)	0.103(5)	0.132(2)	1.28(7)	0.128(1)
$[Li(SL)_2][TfNAc]$	129(1)	0.081(4)	0.101(3)	1.24(7)	0.101(1)

From the actual *ex situ* measured conductivities, as shown in Section 8.2 and reproduced in Table S11, the correction constant for the presence of a separator between the electrodes is defined as in Equation (S9).

$$N_m = \frac{\sigma}{\sigma^*} \tag{S9}$$

As a sanity check, we also measured six coin cells with 0.01 M KCl (assembled outside the glovebox), which resulted in $N_m = 1.25(4)$. Furthermore, the calibrated coin cell conductivities $\sigma_{in\ situ}$ shown in Table S11 were obtained, in excellent agreement with the *ex situ* measured conductivities $\sigma_{ex\ situ}$ for $[Li(SL)_2][TfNCN]$ and $[Li(SL)_2][TfNAc]$. The deviation was slightly larger for $[Li(SL)_2][TfNMs]$, hence we use a final $N_m = 1.24(5)$ based on the results for $[Li(SL)_2][TfNCN]$ and $[Li(SL)_2][TfNAc]$.

9. Salt diffusion

Salt diffusion coefficients were determined using a symmetric Li-Li coin cell assembled as described in Section 7.[59] The measurements were performed using a ModuLab XM potentiostat (Solartron Analytical) and thermostatic chamber (LS-5N BioChamber, Nippon Blower Co. LTD) as described previously, including ageing overnight at 30°C. After recording the open circuit voltage for 30 min, the cells were polarised under galvanostatic conditions for 1 h applying 0.5 mA, thus passing 1.8 C through the cell (potential between 57 mV and 103 mV across all cells and samples). After polarisation, the open circuit voltage was recorded for 12 h and fitted using Equation (S10) for approximately $t > (0.05 \cdot L^2)/D_{salt}$ (*cf.* literature).[59]

$$U(t) = U_{t=0} + a\ \mathrm{e}^{-bt} = U_{t=0} + a\ \mathrm{e}^{-\frac{t}{\tau}} \tag{S10}$$

The resulting fits are presented in Figure S53 to Figure S55.

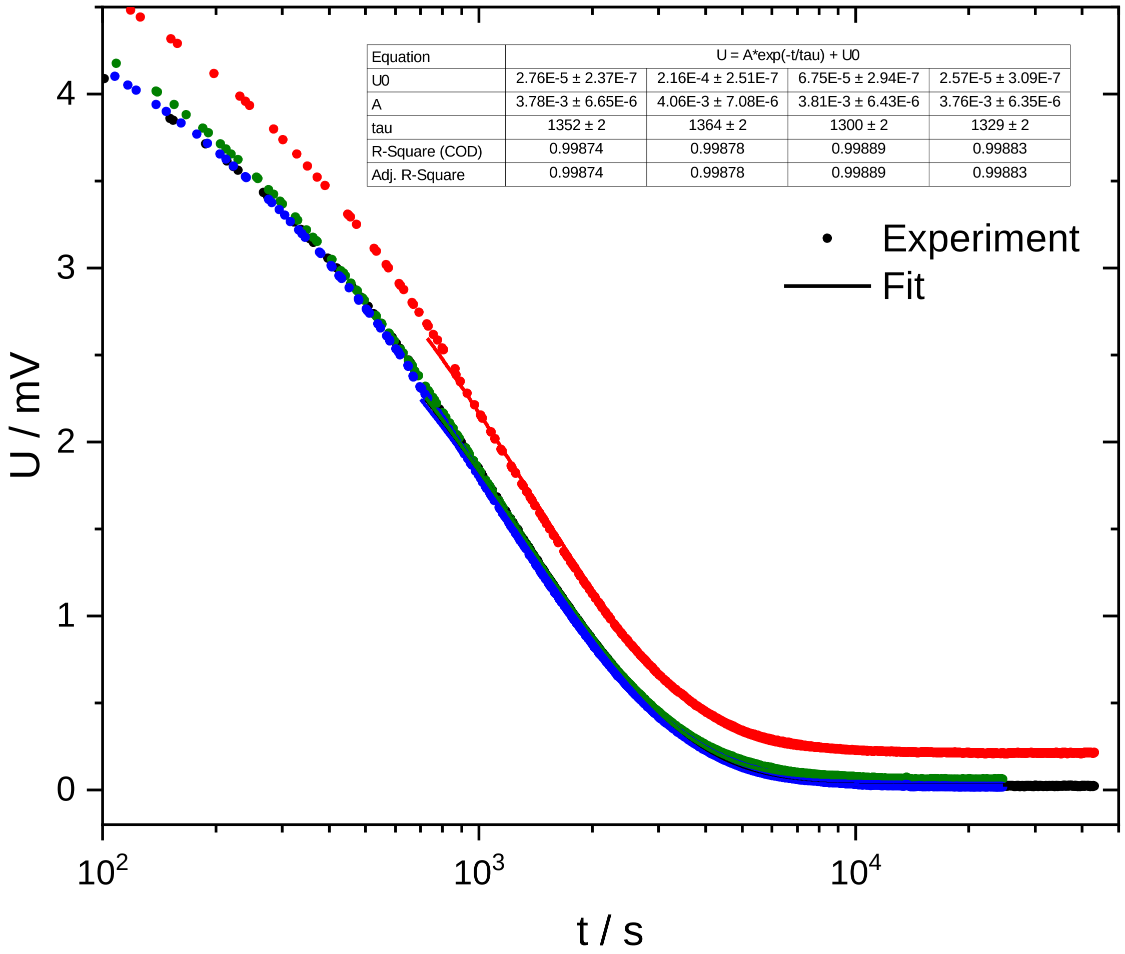

Figure S53: Salt diffusion measurement for four different coin cells (colour coded) with $[Li(SL)_2][TfNCN]$ as sample, exponential fit from t > 700 s.

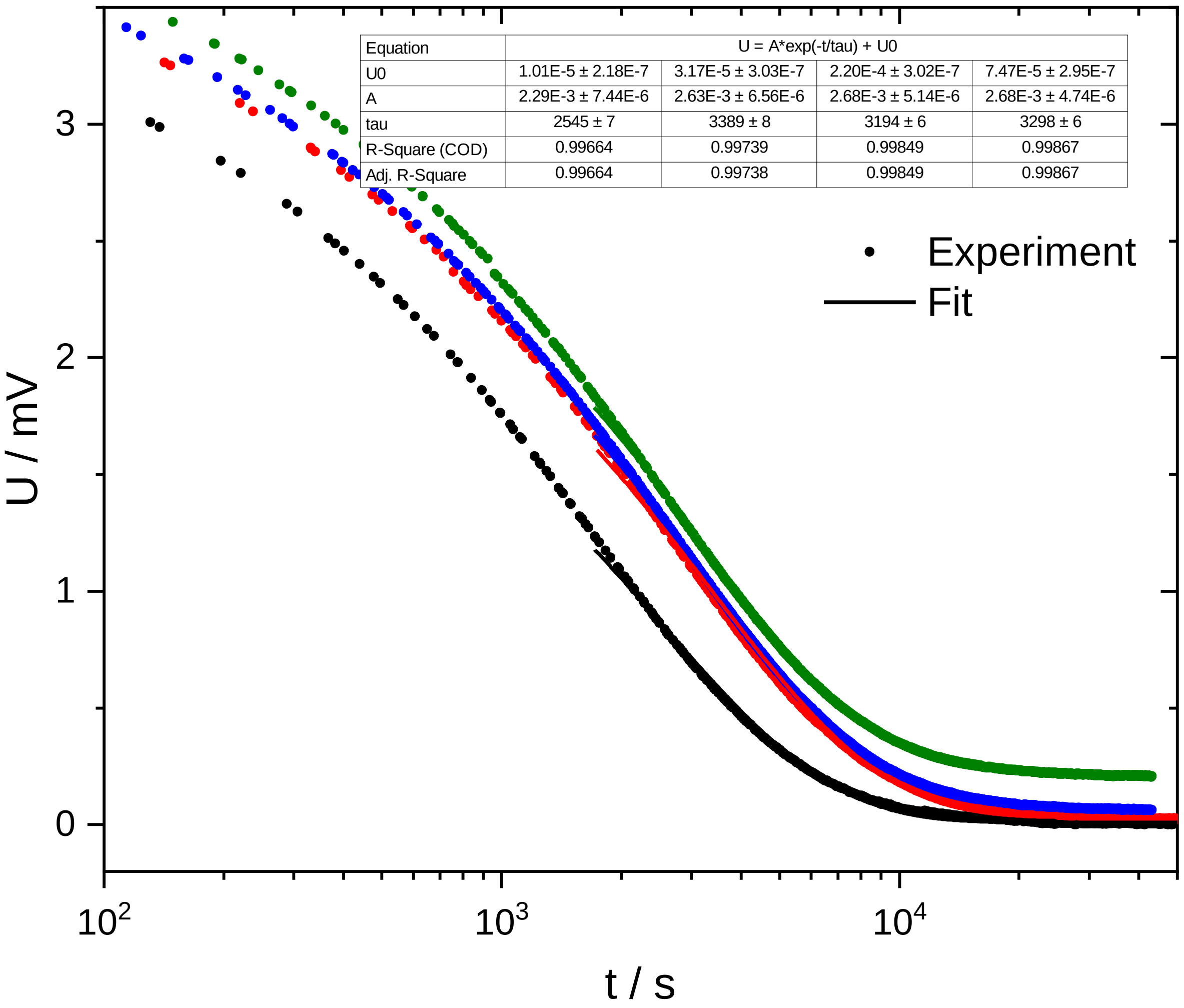

Figure S54: Salt diffusion measurement for four different coin cells (colour coded) with [Li(SL)$_2$][TfNMs] as sample, exponential fit from t > 1700 s.

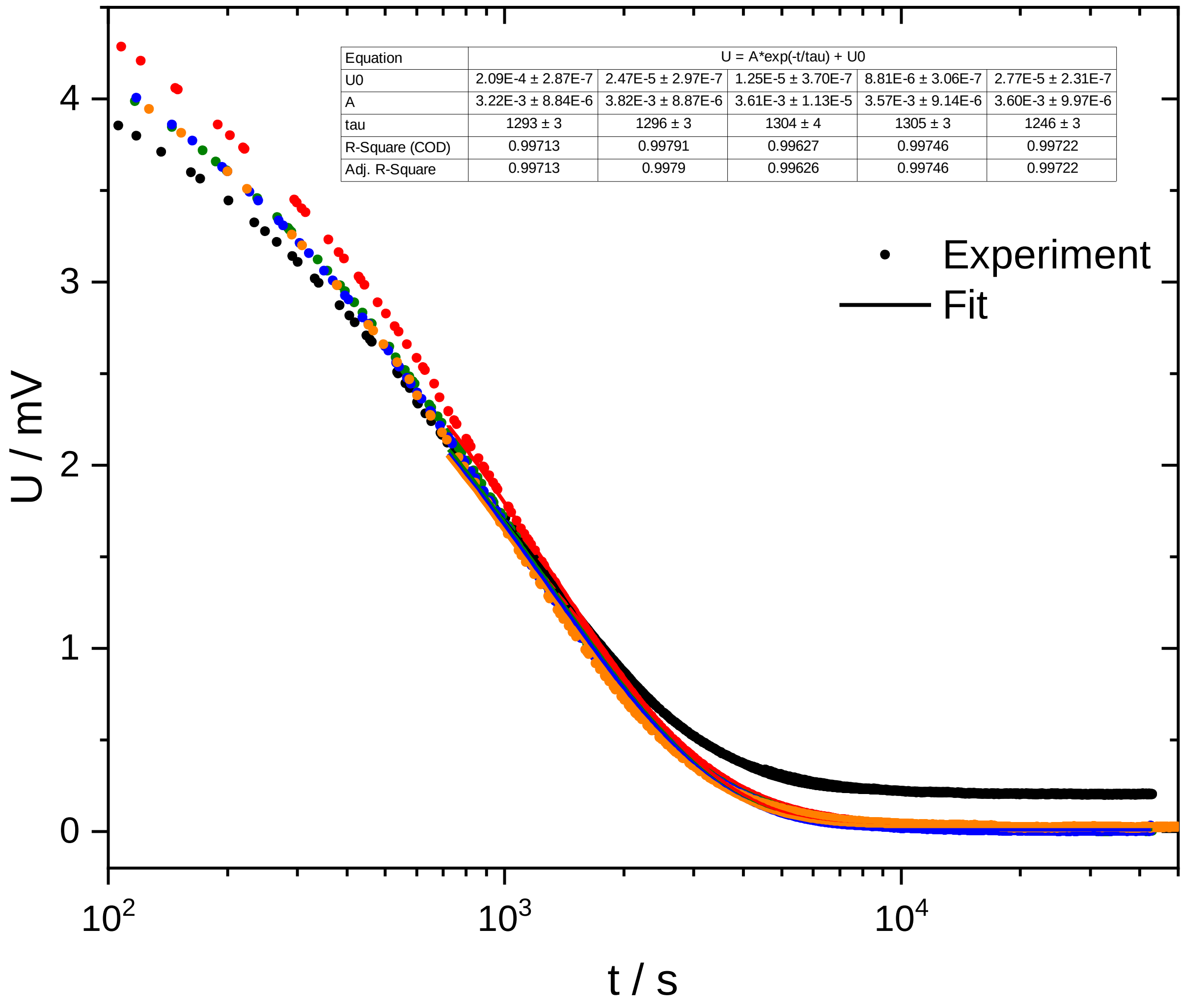

Figure S55: Salt diffusion measurement for five different coin cells (colour coded) with [Li(SL)$_2$][TfNAc] as sample, exponential fit from t > 700 s.

From the fit in Figure S53, the salt diffusion coefficient can be obtained *via* Equation (S11).

$$D_{salt}^{*} = \frac{L^2 b}{\pi^2} = \frac{L^2}{\pi^2 \tau} \qquad \text{(S11)}$$

Here, $L = 0.21(1)$ mm is the separator thickness. D_{salt}^{*} does not include the correction for the presence of the GA55 separator, *cf.* Section 8.2. Thus, following Equation (S9), using $D_{salt} = N_m D_{salt}^{*}$, the values shown in Table S12 were obtained.

Table S12: Experimental salt diffusion coefficients.

	D_{salt}^{*} / 10^{-7} cm^2 s^{-1}	D_{salt} / 10^{-7} cm^2 s^{-1}
[Li(SL)$_2$][TfNCN]	1.05(10)	1.31(14)
[Li(SL)$_2$][TfNMs]	0.46(8)	0.57(10)
[Li(SL)$_2$][TfNAc]	1.09(11)	1.35(14)

10. Electromotive Force

Manipulation of samples and the assembly of the measurement cell were performed inside an argon filled glovebox with ≤0.5 ppm water content (VAC vacuum atmospheres company, model OMNI-LAB). Reference electrodes were prepared using $LiNTf_2$ 1 mol/L in triglyme as reference electrolyte filled in 6 mm glass tubes for electrochemical measurements with IPPG (Ion Permeability Porous Glass) tip (ALS Co. Ltd, Japan). The porous glass part of the electrode was immersed in the reference electrolyte overnight so that the porous glass becomes visibly saturated. Every electrode was prepared by tightly wrapping Li foil (Honjo Metal Co., Ltd., Japan) around Nickel wire (0.8 mm diameter, 99.99 % purity, The Nilaco Corporation, Tokyo Ginza Japan) and placing this Li electrode into the reference electrolyte inside the porous glass sample holder. The outside of the reference electrode was then thoroughly rinsed with the sample electrolyte and the electrode lowered into a test tube filled with the sample electrolyte. Another electrode made from lithium foil wrapped around Nickel wire was placed directly into this sample electrolyte. The open circuit voltage OCV was then recorded every 10 s for 3 h using a ModuLab XM potentiostat (Solartron Analytical) potentiostat with the cell placed in a thermostatic chamber (Model SU-242, ESPEC), stable potential within ca 1 mV was generally observed after ca. 30 min of placing the sample in the thermostatic chamber, see Figure S56 for an example.

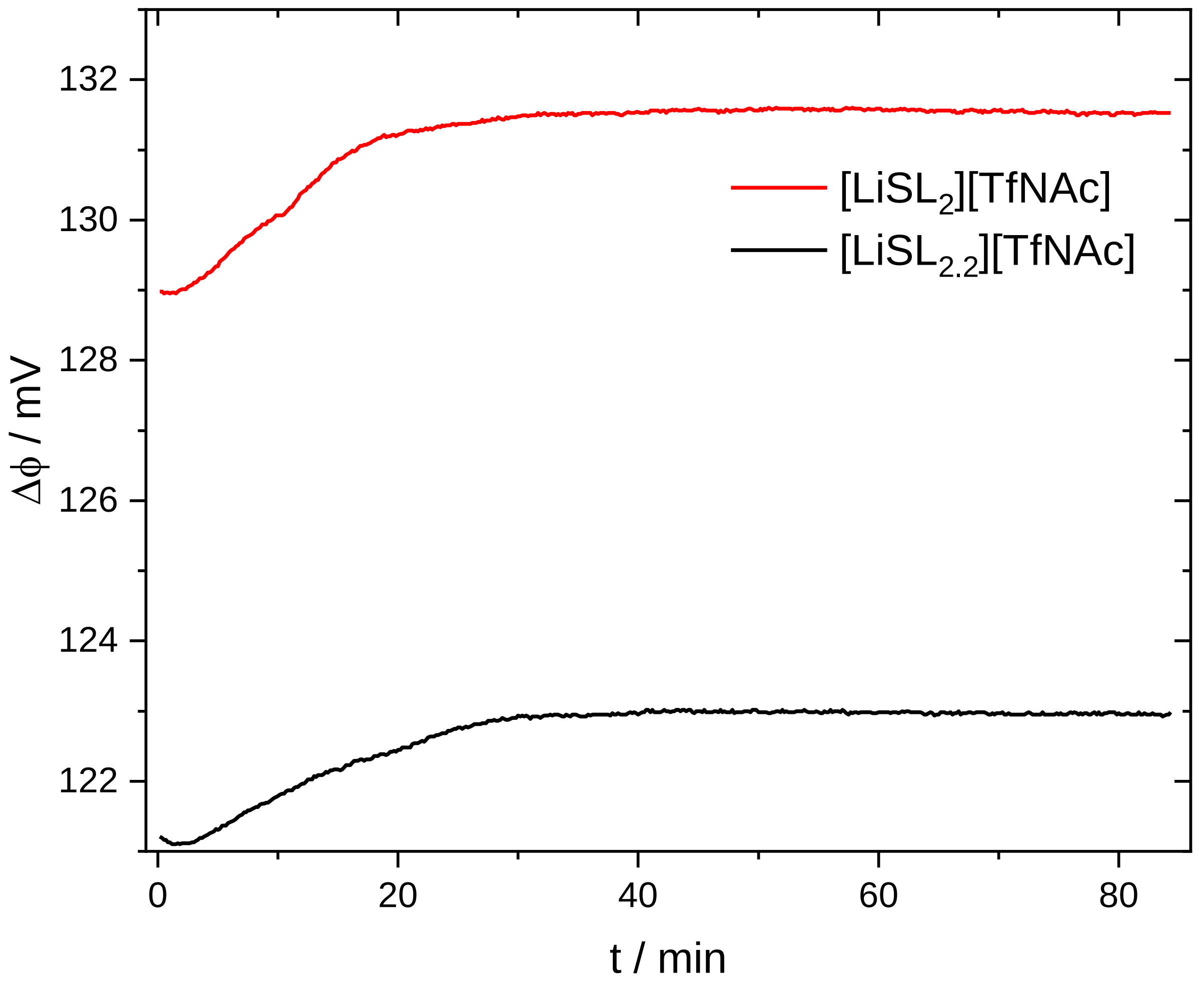

Figure S56: Open circuit voltage of a concentration cell with the sample electrolyte (here $[Li(SL)_x][TfNAc]$ as example) *vs.* 1 M Li[TfNTf] in G3.

From this, $d\Delta\phi/d\ln c$ were obtained, Figure S57 to Figure S59.

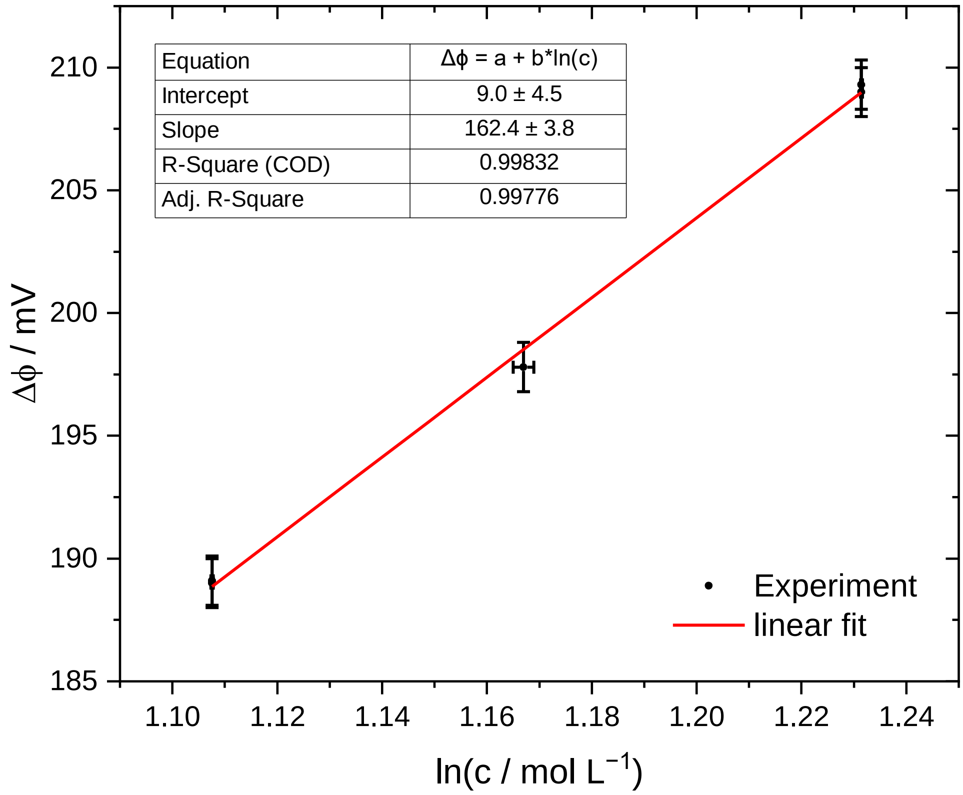

Figure S57: Electrode potential *vs.* Li[TfNTf] 1M in triglyme as a function of concentration. Here, $[Li(SL)_x][TfNCN]$ were measured, with x = 2.0, 2.2, 2.4.

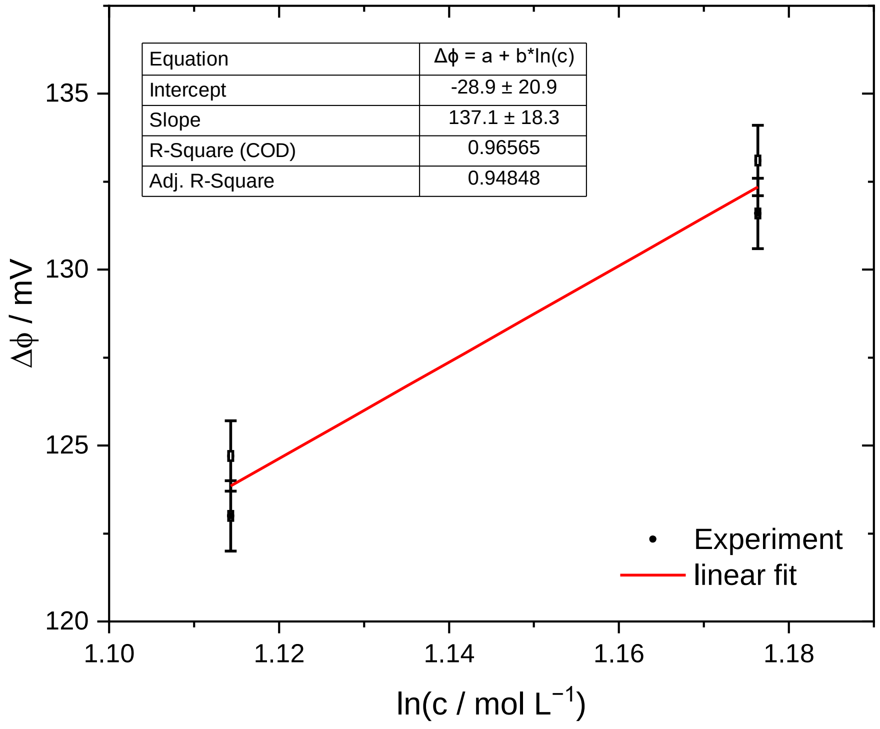

Figure S58: Electrode potential *vs.* Li[TfNTf] 1M in triglyme as a function of concentration. Here, $[Li(SL)_x][TfNAc]$ were measured, with x = 2.0, 2.2.

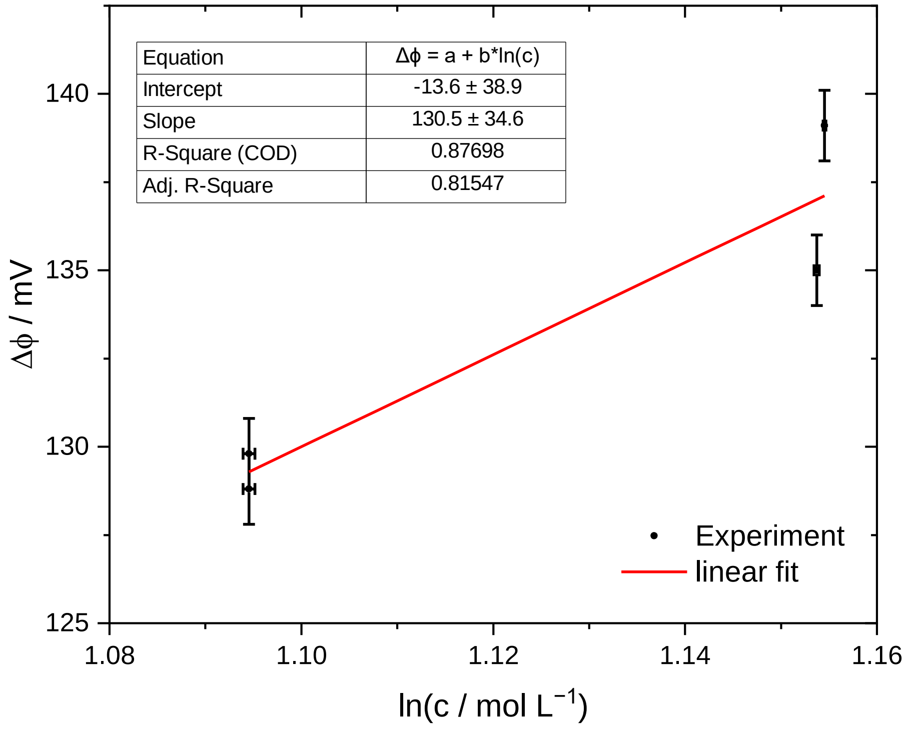

Figure S59: Electrode potential *vs.* Li[TfNTf] 1M in triglyme as a function of concentration. Here, $[Li(SL)_x][TfNMs]$ were measured, with x = 2.0, 2.2.

11. PFGSTE NMR Diffusometry

Diffusion coefficients were measured at 30.0°C *via* NMR diffusometry on an ECX 400 spectrometer (JEOL) equipped with a diffusion probe head with maximum magnetic field gradient of 12 T/m using a pulsed field gradient stimulated echo (PFGSTE) pulse sequence with bipolar sine-shaped pulsed gradients. The samples were filled into Shigemi tubes (BMS-005J, SHIGEMI Co., LTD.) under argon in a glovebox with ≤0.5 ppm water content (VAC vacuum atmospheres company, model OMNI-LAB). The parameters were optimised manually for each measurement (frequency offset, sweep width, time domain, 90° pulse duration, T1 from inversion recovery, diffusion time Δ, gradient duration δ, gradient strength g). The relaxation delay was chosen to be 9 times T_1. The PFGSTE experiment itself was performed using 4 dummy scans and 16 scans per gradient, with 16 increments from weakest to strongest gradient. The diffusion coefficient D was obtained using the Stejskal-Tanner Equation (S12).[60–62] Here, τ is the gradient interspacing (waiting time between the bipolar gradient pulses).

$$\ln\frac{I}{I_0} = -D\gamma^2\delta^2 g^2\left(\Delta - \frac{\delta}{3} - \frac{\tau}{2}\right) \quad \text{(S12)}$$

Using the Nernst-Einstein relation, the self components of the ionic conductivity can be calculated as shown in Equation (S13) and (S14).

$$\sigma_+^{self} = \frac{c_{salt}F^2}{RT} \cdot D_+ \quad \text{(S13)}$$

$$\sigma_-^{self} = \frac{c_{salt}F^2}{RT} \cdot D_- \quad \text{(S14)}$$

Equation (S13) and (S14) are only valid in infinite dilution for electrolytes without ion correlation.[63] Thus, an estimate for the transference number neglecting ion correlations, $t_{Li^+}^{PFG}$, can be obtained as shown in Equation

$$t_{Li^+}^{PFG} = \frac{\sigma_+^{self}}{\sigma_+^{self} + \sigma_-^{self}} = \frac{D_+}{D_+ + D_-} \quad \text{(S15)}$$

An overview of the experimental results and derived quantities is given in Table S13.

Table S13: Overview of the results from the PFGSTE NMR measurement as well as derived quantities. a-d denote repeat measurements.

	Self-diffusion D / 10^{-12} m^2 s^{-1}			$\frac{D_{Li^+}}{D_{Sulfolane}}$	$\frac{D_{Li^+}}{D_{Anion}} = \frac{D_+}{D_-}$	$t_{Li^+}^{PFG}$
	Sulfolane	Lithium	Anion			
[Li(SL)$_2$][TfNCN] a	1.68	1.83	1.03	1.09	1.77	0.64
[Li(SL)$_2$][TfNCN] b	1.64	1.46	1.12	0.88	1.30	0.57
[Li(SL)$_2$][TfNCN] c	1.61	1.76	0.99	1.10	1.77	0.64
[Li(SL)$_2$][TfNCN] d	1.61	1.78	1.00	1.11	1.79	0.64
[Li(SL)$_3$][TfNCN]	8.51	5.79	4.23	0.68	1.37	0.58
[Li(SL)$_2$][TfNMs] a	1.16	1.23	0.73	1.06	1.67	0.63
[Li(SL)$_2$][TfNMs] b	1.17	1.36	0.71	1.16	1.92	0.66
[Li(SL)$_2$][TfNMs] c	1.14	1.33	0.70	1.17	1.92	0.66
[Li(SL)$_2$][TfNMs] d	1.11	1.29	0.68	1.17	1.90	0.65
[Li(SL)$_2$][TfNFs]	4.98	6.84	4.46	1.37	1.53	0.61
[Li(SL)$_2$][TfNTf][64]	2.9	3.5	2.2	1.2	1.6	0.61
[Li(SL)$_2$][PfNFs]	3.91	4.71	3.64	1.20	1.29	0.56
[Li(SL)$_3$][TfCHTf]	6.61	4.75	3.46	0.72	1.37	0.58
[Li(SL)$_2$][TfNAc] a	4.66	1.71	1.66	0.37	1.03	0.51
[Li(SL)$_2$][TfNAc] b	4.11	2.09	1.57	0.51	1.33	0.57
[Li(SL)$_2$][TfNAc] c	4.09	2.10	1.57	0.51	1.33	0.57
[Li(SL)$_2$][TfNAc] d	4.07	1.29	1.57	0.32	0.82	0.45

12. Electrophoretic NMR

Electrophoretic NMR (eNMR) allows the measurement of ionic electrophoretic mobilities by applying an electric field pulse in z direction to a sample during an NMR measurement. Before and after the electric field pulse, the position in z direction of a nucleus is encoded and read out using magnetic field gradient pulses similar to NMR diffusometry. However, in contrast to diffusometry the collective motion of an ion species in the electric field yields a phase shift of the signal.

The eNMR measurements were performed on an Avance Neo 400 spectrometer (Bruker, Germany) equipped with DiffBB probe head (Bruker, Germany) with maximum magnetic field gradient strength of 17 T m^{-1}, using a double stimulated echo sequence. Application of the electric field in an NMR tube is enabled by a sample holder, containing the electrodes, which is immersed into a 5 mm NMR tube.[65]

The mobility can be obtained *via* Equation (S16), more details regarding the experimental setup can be found in the Literature.[65,66]

$$\varphi - \varphi_0 = \gamma g \delta \Delta \frac{U}{d} \cdot \mu_i \tag{S16}$$

Here, similar to (S12), γ is the gyromagnetic ratio of the observed nucleus, Δ is the observation time (50 ms to 350 ms), δ (2-3 ms) is the duration of the magnetic field gradient pulse and g is its strength. U (increased incrementally from 0 V up to 80 V to 200 V depending on the sample) is the applied voltage, d is the electrode distance (2.2 cm), and μ_i is the electrophoretic mobility of species i.

The electrophoretic mobility of a species contributes $\sigma_i^{\mu} = z_i c_i \mu_i F$ to the total conductivity. Thus, the lithium transference can be calculated as shown in Equation (S17).

$$t_{Li^+}^{\mu} = \frac{\mu_{Li^+}}{\mu_{Li^+} - \mu_{Anion}} \tag{S17}$$

Representative phase shifts of the three electrolytes are shown in Figure S60 to Figure S62, here the phase shift is normalized to the experimental parameters to enable a comparison. The mobilities are determined from a linear fit and mobility values are averaged over at least three independent measurements on newly filled sample holders.

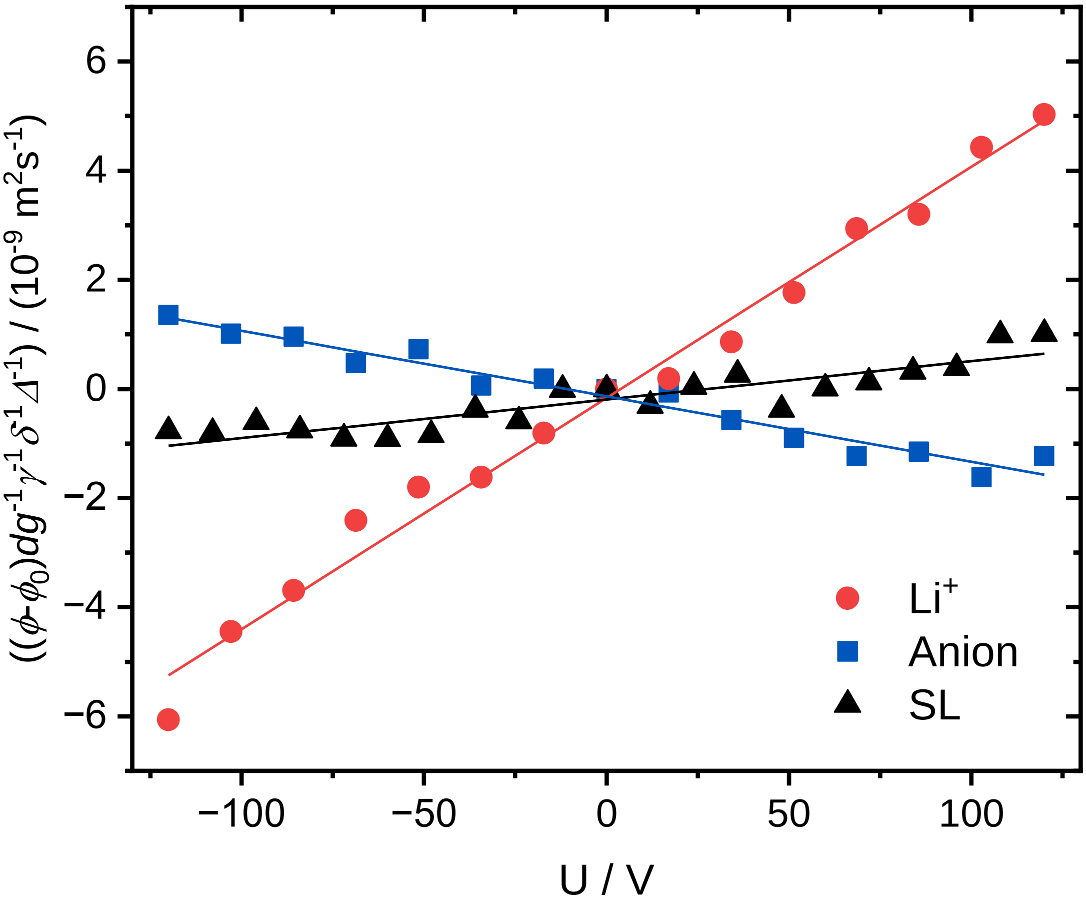

Figure S60: Representative reduced phase shifts from the eNMR measurement for [Li(SL)$_2$][TfNCN].

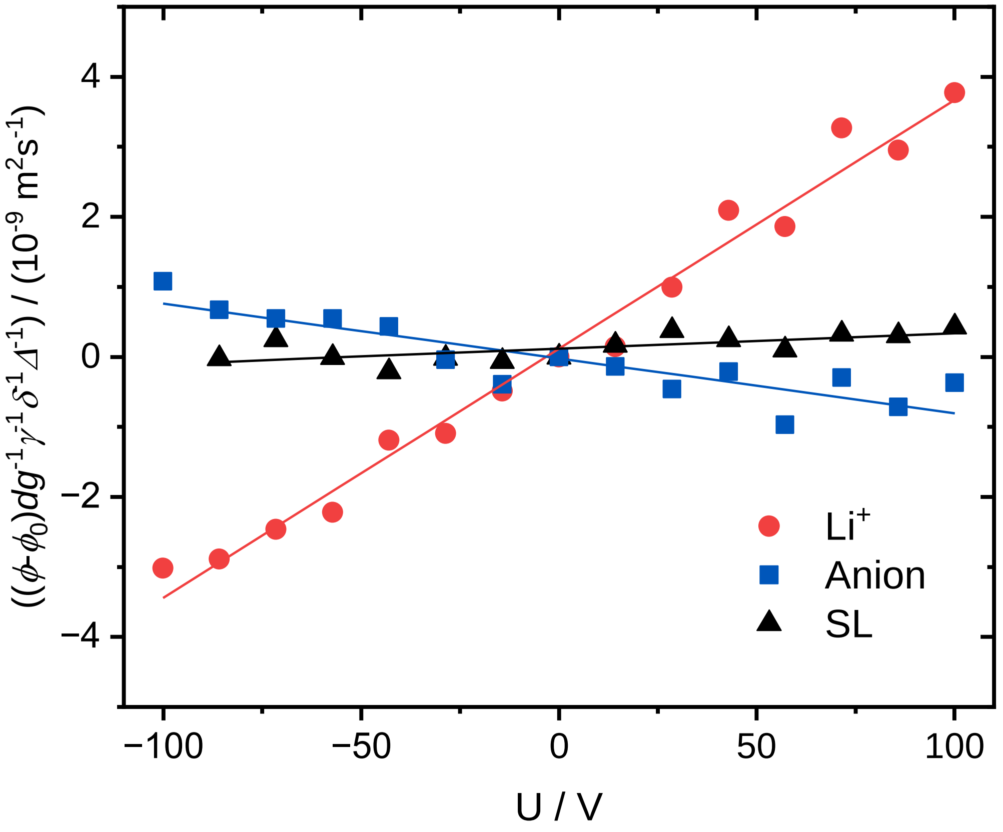

Figure S61: Representative reduced phase shifts from the eNMR measurement for [Li(SL)$_2$][TfNMs].

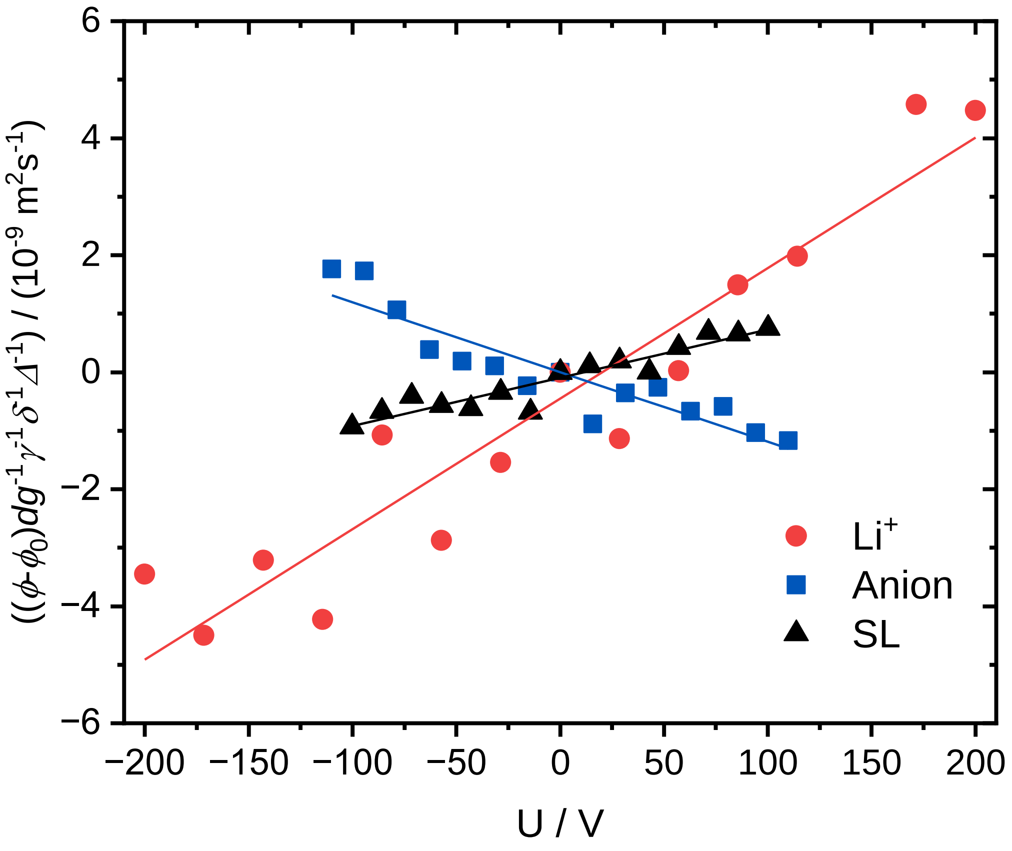

Figure S62: Representative reduced phase shifts from the eNMR measurement for [Li(SL)$_2$][TfNAc].

An overview of the resulting mobilities and the transference numbers derived from this can be found in Table S14 and Figure S63.

Table S14: Overview of the electrophoretic mobilities determined *via* eNMR as well as the determined transference numbers.

System	Mobility μ / 10^{-11} m^2 V^{-1} s^{-1}			$t^{\mu}_{Li^+}$
	Sulfolane	Lithium	Anion	
[Li(SL)$_2$][TfNCN]	0.54 ± 0.20	4.15 ± 0.48	−1.27 ± 0.18	0.77 ± 0.05
[Li(SL)$_2$][TfNMs]	0.17 ± 0.09	3.76 ± 0.48	−0.80 ± 0.16	0.82 ± 0.05
[Li(SL)$_2$][TfNAc]	0.82 ± 0.19	2.24 ± 0.53	−1.24 ± 0.28	0.64 ± 0.11

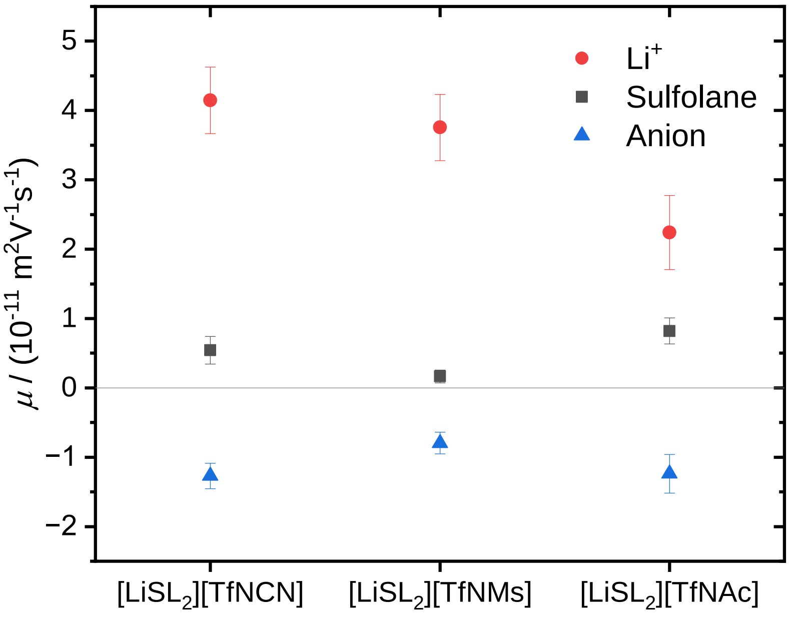

Figure S63: Overview of the electrophoretic mobilities determined *via* eNMR.

Finally, the α and β values were calculated from the eNMR experiments as explained in Section 13.2 and plotted in Figure S64 for comparison with the corresponding contour plot in the main manuscript.[67]

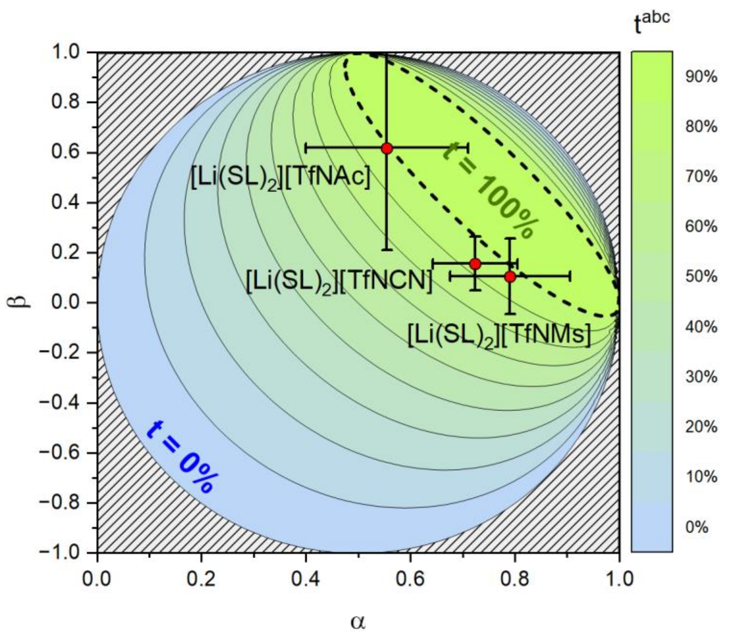

Figure S64: Contour plot showing the transference number resulting from certain combinations of the α and β parameters. The experimental data points shown here were obtained from the eNMR measurements.

13. Onsager coefficients

The Li⁺ transference numbers t_{Ω,Li^+}^{PP} and $t_{Li^+}^{\mu}$ are related to the Onsager coefficients, Equation (S18) and Equation (S19), with the denominator being equal to the ionic conductivity σ.[66,68]

$$t_{\Omega,Li^+}^{PP} = \frac{\sigma_{++} - \frac{(\sigma_{+-})^2}{\sigma_{--}}}{\sigma_{++} + \sigma_{--} - 2\sigma_{+-}} \tag{S18}$$

$$t_{Li^+}^{\mu} = t_{Li^+}^{eNMR} = \frac{\sigma_{++} - \sigma_{+-}}{\sigma_{++} + \sigma_{--} - 2\sigma_{+-}} \tag{S19}$$

Hence, the ratio of t_{Ω,Li^+}^{PP} and $t_{Li^+}^{\mu}$ can also be calculated in this framework, Equation (S20).

$$\frac{t_{\Omega,Li^+}^{PP}}{t_{Li^+}^{\mu}} = \frac{\sigma_{++} - \frac{(\sigma_{+-})^2}{\sigma_{--}}}{\sigma_{++} - \sigma_{+-}} \tag{S20}$$

Furthermore, it is clear from Equation (S18) and Equation (S19) that, if the condition $\sigma_{+-} = \sigma_{--}$ is fulfilled, then both t_{Ω,Li^+}^{PP} and $t_{Li^+}^{\mu}$ reach unity.

13.1. Method A

We follow the literature in order to calculate the Onsager coefficients from σ, t^{PP}_{Ω,Li^+}, D_{salt}, c_{salt}, and $d\Delta\phi/d\ln c$, however using slightly different definitions of A_1 to A_4 as shown in Equation (S21) to (S24).[58,69,70] [1] The reason for the different choice is that the conductivity cancels out in most terms, thus the definition used in the references cited above artificially increases the error predicted by the gaussian error propagation.

$$A_1 = \sigma \tag{S21}$$

$$A_2 = t^{PP}_{\Omega,Li^+} \tag{S22}$$

$$A_3 = D_{salt} \cdot c_{salt} \frac{F^2}{2RT} \tag{S23}$$

$$A_4 = \frac{d\Delta\phi}{d\ln c} \cdot \frac{F}{2RT} \tag{S24}$$

Here, F is Faraday's constant, R is the ideal gas constant, $T = 303.15\,\mathrm{K}$ is the temperature. The Onsager coefficients then take the form in Equation (S25) to (S27). The experimental values used to evaluate Onsager coefficients as well as the resulting derived quantities are summarised in Table S15.

$$\sigma_{++} = \frac{{A_1}^2{A_2}^2{A_4}^2 - 2A_1A_2A_3A_4 + {A_3}^2 + 2A_1A_2^2A_3A_4 - A_2{A_3}^2}{A_1{A_2}^2{A_4}^2} \tag{S25}$$

$$\sigma_{--} = \frac{{A_3}^2(1 - A_2)}{A_1{A_2}^2A_4^2} \tag{S26}$$

$$\sigma_{+-} = \frac{{A_3}^2 - A_1A_2A_3A_4 + A_1{A_2}^2A_3A_4 - A_2{A_3}^2}{A_1{A_2}^2A_4^2} \tag{S27}$$

[1] There are understandably typos in the ${A_2}^2A_3A_4$ and $F/(2RT)$ terms in some publications.

Table S15: Overview of experimental values, resulting Onsager coefficients and other derived quantities, and their uncertainty

	$[Li(SL)_2][TfNCN]$	$[Li(SL)_2][TfNMs]$	$[Li(SL)_2][TfNAc]$
σ / mS cm^{-1}	0.173(4)	0.132(2)	0.101(3)
t^{PP}_{Ω,Li^+}	0.832(8)	0.869(34)	0.891(18)
D_{salt} / 10^{-11} m^2 s^{-1}	1.31(14)	0.57(10)	1.35(14)
$\frac{d\Delta\phi}{d\ln c}$ / mV	163(4)	131(35)	137(18)
c_{salt} / mol L^{-1}	3.426(2)	3.171(2)	3.242(2)
D^+ / 10^{-12} m^2 s^{-1}	1.64(2)	1.30(11)	1.80(19)
D^- / 10^{-12} m^2 s^{-1}	1.03(3)	0.71(4)	1.59(2)
σ_{++} / mS cm^{-1}	0.165(10)	0.115(5)	0.155(34)
σ_{--} / mS cm^{-1}	0.099(22)	0.023(17)	0.129(52)
σ_{+-} / mS cm^{-1}	0.045(16)	0.003(9)	0.092(43)
σ_+^{self} / mS cm^{-1}	0.216(11)	0.153(3)	0.215(23)
σ_-^{self} / mS cm^{-1}	0.131(4)	0.083(1)	0.191(3)
$\sigma_{++}^{distinct}$ / mS cm^{-1} $= \sigma_{++} - \sigma_+^{self}$	−0.051(15)	-0.038(6)	−0.060(41)
$\sigma_{--}^{distinct}$ / mS cm^{-1} $= \sigma_{--} - \sigma_-^{self}$	−0.032(23)	−0.059(17)	−0.061(52)
σ^{cross} / mS cm^{-1} $= \sigma_{++}^{distinct} + \sigma_{--}^{distinct} - 2 \cdot \sigma_{+-}$	−0.174(42)	−0.103(25)	−0.31(11)
σ^{self} / mS cm^{-1} $= \sigma_+^{self} + \sigma_-^{self}$	0.347(14)	0.235(5) mS/cm	0.406(26)
α	0.62(6)	0.83(11)	0.54(13)
β	0.34(14)	0.05(14)	0.64(36)

13.2. Method B

The Onsager coefficients can also be obtained from the eNMR experiment using $t^{\mu}_{Li^+}$ and t^{PP}_{Ω,Li^+}, Equation (S28) to (S30).[66] An overview of the relevant derived quantities can be found in Table S16.

$$\frac{\sigma_{++}}{\sigma} = \frac{2 \cdot t^{\mu}_{Li^+} \cdot t^{PP}_{\Omega,Li^+} - t^{PP}_{\Omega,Li^+} - \left(t^{\mu}_{Li^+}\right)^2}{t^{PP}_{\Omega,Li^+} - 1} \tag{S28}$$

$$\frac{\sigma_{--}}{\sigma} = \frac{\left\{t^{\mu}_{Li^+} \cdot t^{PP}_{\Omega,Li^+} - t^{PP}_{\Omega,Li^+} + t^{\mu}_{Li^+} - \left(t^{\mu}_{Li^+}\right)^2\right\}^2}{\left(1 - t^{PP}_{\Omega,Li^+}\right)\left(t^{PP}_{\Omega,Li^+} - t^{\mu}_{Li^+}\right)^2} \tag{S29}$$

$$\frac{\sigma_{+-}}{\sigma} = \frac{t^{\mu}_{Li^+} \cdot t^{PP}_{\Omega,Li^+} - t^{PP}_{\Omega,Li^+} + t^{\mu}_{Li^+} - \left(t^{\mu}_{Li^+}\right)^2}{t^{PP}_{\Omega,Li^+} - 1} \tag{S30}$$

Table S16: Overview of Onsager coefficients and other quantities derived from the eNMR measurements with their uncertainty.

	[Li(SL)2][TfNCN]	[Li(SL)2][TfNMs]	[Li(SL)2][TfNAc]
σ_{++} / mS cm^{-1}	0.154(16)	0.123(16)	0.158(51)
σ_{--} / mS cm^{-1}	0.059(18)	0.033(16)	0.127(62)
σ_{+-} / mS cm^{-1}	0.017(11)	0.008(12)	0.088(52)
$\sigma_{++}^{distinct}$ / mS cm^{-1}	−0.062(19)	−0.029(17)	−0.057(56)
$\sigma_{--}^{distinct}$ / mS cm^{-1}	−0.072(18)	−0.050(16)	−0.064(62)
σ^{cross} / mS cm^{-1}	−0.168(35)	−0.096(33)	−0.30(13)
α	0.72(8)	0.79(11)	0.55(16)
β	0.16(11)	0.11(15)	0.62(41)

14. Error estimates

The error bars given for the Onsager coefficients σ_{xy} were estimated using Gaussian error propagation, Equation (S31). For the sake of completeness, the derivatives we used are given below.

$$\Delta\sigma_{xy} \approx \sqrt{\sum_{i=1,2,3,4} \left(\frac{d\sigma_{xy}}{dA_i} \cdot \Delta A_i\right)^2} \tag{S31}$$

14.1. Method A

Derivatives of σ_{++}, Equation (S32) to (S35).

$$\frac{d\sigma_{++}}{dA_1} = 1 + \frac{(A_2 - 1)A_3^2}{A_1^2 A_2^2 A_4^2} \tag{S32}$$

$$\frac{d\sigma_{++}}{dA_2} = \frac{A_3\big((A_2 - 2)\,A_3 + 2\,A_1 A_2 A_4\big)}{A_1 A_2^3 A_4^2} \tag{S33}$$

$$\frac{d\sigma_{++}}{dA_3} = \frac{2(\,A_2 - 1)(A_1 A_2 A_4 - A_3)}{A_1 A_2^2 A_4^2} \tag{S34}$$

$$\frac{d\sigma_{++}}{dA_4} = \frac{2(\,A_2 - 1)A_3(A_3 - A_1 A_2 A_4)}{A_1 A_2^2 A_4^3} \tag{S35}$$

Derivatives of σ_{+-}, Equation (S36) to (S39).

$$\frac{d\sigma_{+-}}{dA_1} = \frac{(A_2 - 1)A_3^2}{A_1^2 A_2^2 A_4^2} \tag{S36}$$

$$\frac{d\sigma_{+-}}{dA_2} = \frac{A_3\big((A_2 - 2)A_3 + A_1 A_2 A_4\big)}{A_1 A_2^3 A_4^2} \tag{S37}$$

$$\frac{d\sigma_{+-}}{dA_3} = \frac{(A_2 - 1)(A_1 A_2 A_4 - 2A_3)}{A_1 A_2^2 A_4^2} \tag{S38}$$

$$\frac{d\sigma_{+-}}{dA_4} = \frac{(A_2 - 1)A_3(2A_3 - A_1 A_2 A_4)}{A_1 A_2^2 A_4^3} \tag{S39}$$

Derivatives of σ_{--}, Equation (S40) to (S43).

$$\frac{d\sigma_{--}}{dA_1} = \frac{(A_2 - 1)A_3^2}{A_1^2 A_2^2 A_4^2} \tag{S40}$$

$$\frac{d\sigma_{--}}{dA_2} = \frac{(A_2 - 2)A_3^2}{A_1 A_2^3 A_4^2} \tag{S41}$$

$$\frac{d\sigma_{--}}{dA_3} = -\frac{2(A_2 - 1)A_3}{A_1 A_2^2 A_4^2} \tag{S42}$$

$$\frac{d\sigma_{--}}{dA_4} = -\frac{2(A_2 - 1)A_3^2}{A_1 A_2^2 A_4^3} \tag{S43}$$

The uncertainties ΔA_i were, in turn, also calculated with gaussian error propagation. The sources of error considered were the precision of the balance when weighing the salt and solvent, error of the density, diffusion, and conductivity measurements, the experimental uncertainty of t_{Ω,Li^+}^{PP} as standard error of the mean over six coin cells, uncertainty of the separator thickness as 0.01 mm, uncertainty in b from Equation (S10), uncertainty of the bulk resistance measurement under blocking conditions, uncertainty in the EMF from the linear fits.

14.2. Method B

Error bars given in the main manuscript for the Onsager coefficient determined using eNMR were calculated as shown in Equation (S44) to (S46).

$$\Delta\sigma_{++} \approx \sqrt{(\sigma_{++} \cdot \Delta\sigma^{\mu})^2 + \left(\frac{2\sigma^{\mu}\left(t_{\Omega,Li^+}^{PP} - t_{Li^+}^{\mu}\right)}{t_{\Omega,Li^+}^{PP} - 1} \cdot \Delta t_{Li^+}^{\mu}\right)^2 + \left(\frac{\sigma^{\mu}\left(t_{Li^+}^{\mu} - 1\right)^2}{\left(t_{\Omega,Li^+}^{PP} - 1\right)^2} \cdot \Delta t_{\Omega,Li^+}^{PP}\right)^2} \tag{S44}$$

$$\Delta\sigma_{--} \approx \sqrt{(\sigma_{--} \cdot \Delta\sigma^{\mu})^2 + \left(\frac{2\sigma^{\mu}\left(t_{Li^+}^{\mu} - 1\right)}{t_{\Omega,Li^+}^{PP} - 1} \cdot \Delta t_{Li^+}^{\mu}\right)^2 + \left(\frac{\sigma^{\mu}\left(t_{Li^+}^{\mu} - 1\right)^2}{\left(1 - t_{\Omega,Li^+}^{PP}\right)^2} \cdot \Delta t_{\Omega,Li^+}^{PP}\right)^2} \tag{S45}$$

$$\Delta\sigma_{+-} \approx \sqrt{(\sigma_{+-} \cdot \Delta\sigma^{\mu})^2 + \left(\frac{\sigma^{\mu}\left(t_{Li^+}^{\mu} - 1\right)^2}{\left(t_{\Omega,Li^+}^{PP} - 1\right)^2} \cdot \Delta t_{\Omega,Li^+}^{PP}\right)^2 + \left(\frac{\sigma^{\mu}\left(t_{\Omega,Li^+}^{PP} - 2t_{Li^+}^{\mu} + 1\right)}{t_{\Omega,Li^+}^{PP} - 1} \cdot \Delta t_{Li^+}^{\mu}\right)^2} \tag{S46}$$

Here, the experimental uncertainty of t_{Ω,Li^+}^{PP} as standard error of the mean over six coin cells, the precision of the balance when weighing the salt and solvent, estimated error of the density, and experimental uncertainty of the mobility measurements were considered.

15. Electrochemical window

Cyclic voltammograms and linear sweep voltammograms were recorded in a three-electrode setup (VB7 cell, EC FRONTIER CO., LTD.) with platinum coil counter electrode (CE-200, EC FRONTIER CO., LTD.), working electrode (Copper CU-6355 for reductive stability or Platinum PT-6355 for oxidative stability, 3 mm diameter, EC FRONTIER CO., LTD.), and a Li/Li^+ reference electrode prepared as described in Section 0 (rinsed with the sample electrolyte before assembly). Before use, the copper (or platinum) electrode was polished using deagglomerated alumina suspensions (SANKEI Co., Ltd.) with decreasing particle sizes of 1 µm, 0.3 µm, 0.05 µm (three minutes for each particle size). The platinum coil electrode was heated to glow in an ethanol flame before use.

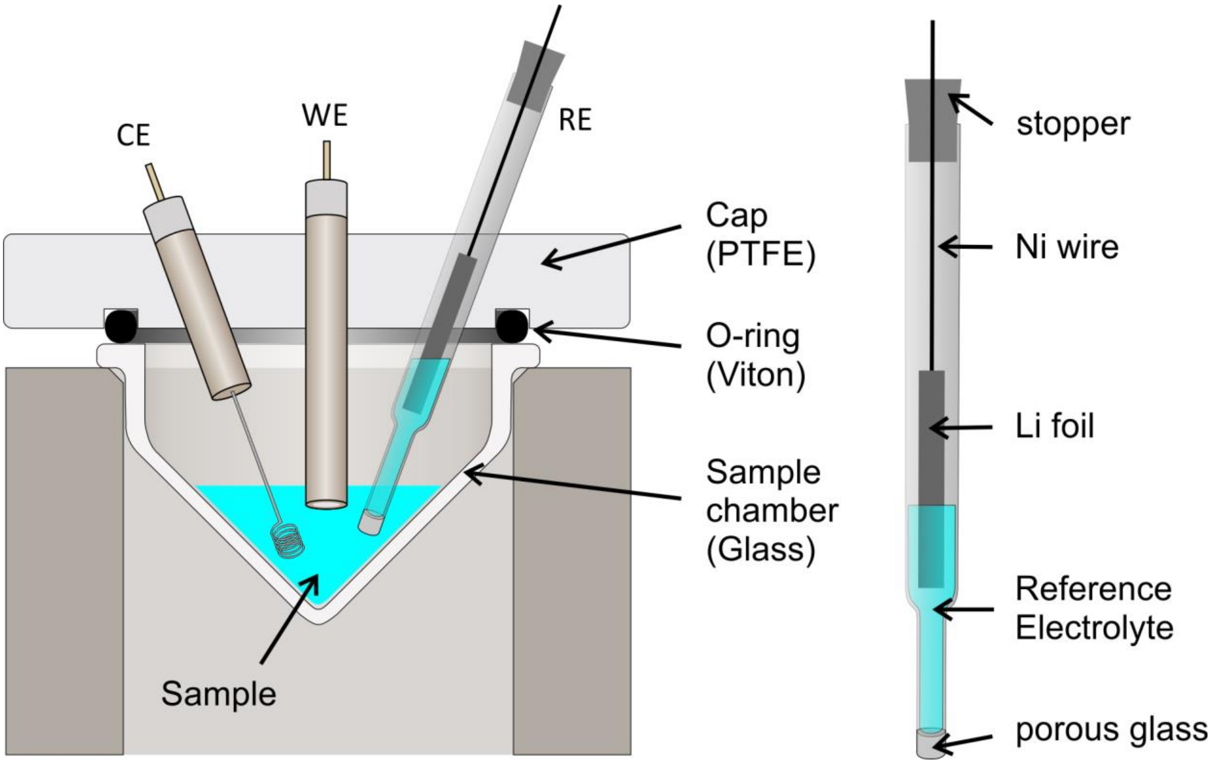

Figure S65: Schematic view of the three electrode setup used for CV and LSV measurements.

The cell and the reference electrode were assembled inside an argon filled glovebox with ≤0.5 ppm water content (VAC vacuum atmospheres company, model OMNI-LAB). The measurements were performed using a SP-300 potentiostat (BioLogic SAS) with the cell placed in a thermostatic chamber (LS-5N BioChamber, Nippon Blower Co. LTD) at 30°C. Before the measurement, cells were allowed to rest while recording the OCV for 2h to ensure stability. For the reductive stability on a copper working electrode measured *via* cyclic voltammetry, the potential was scanned between −0.2 V and +2 V (*vs*. Li/Li^+), starting with a reductive step from OCV, and 30 repeats, Figure S66 to Figure S71. The oxidative stability on a platinum working electrode was measured *via* linear sweep voltammetry, scanning from OCV to +5.5 V (*vs*. Li/Li^+), see Figure S72 to Figure S77. Scan speeds for CV and LSV were 1 mV/s. Results are summarised in Table S17.

Table S17: Electrode potential *vs.* Li/Li^+ at which the current threshold of 10 µA/cm² was reached as well as the electrochemical window.

	E_{WE} at current threshold / V			Electrochemical window $E_{Ox} - E_{Red}$ / V
	Reduction		Oxidation	
	First cycle	Last cycle		
$[Li(SL)_2][TfNCN]$	1.79	0.17	4.99	4.82
$[Li(SL)_3][TfNCN]$	1.76	0.12	4.96	4.84
$[Li(SL)_2][TfNMs]$	1.93	0.08	4.27	4.19
$[Li(SL)_2][TfNAc]$	1.73	0.06	4.91	4.85
$[Li(DMSO)_3][MsNMs]$	1.18	< −0.20	4.33	> 4.53
$[Li(DMSO)_3][MsNMs]$	1.28	< −0.20	4.26	> 4.46

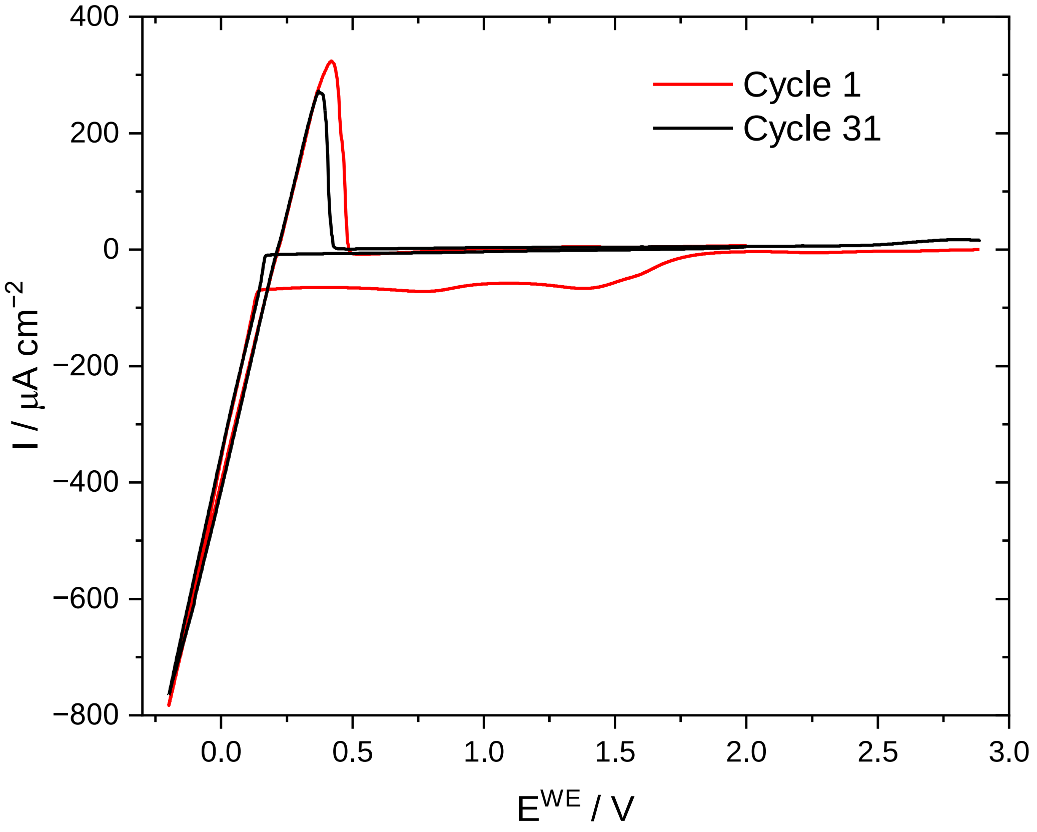

Figure S66: first and last cycle of the CV of [Li(SL)$_2$][TfNCN] with Cu working electrode. The current threshold of 10 µA/cm² was reached at +1.79 V and +0.17 V for the first and last cycle, respectively.

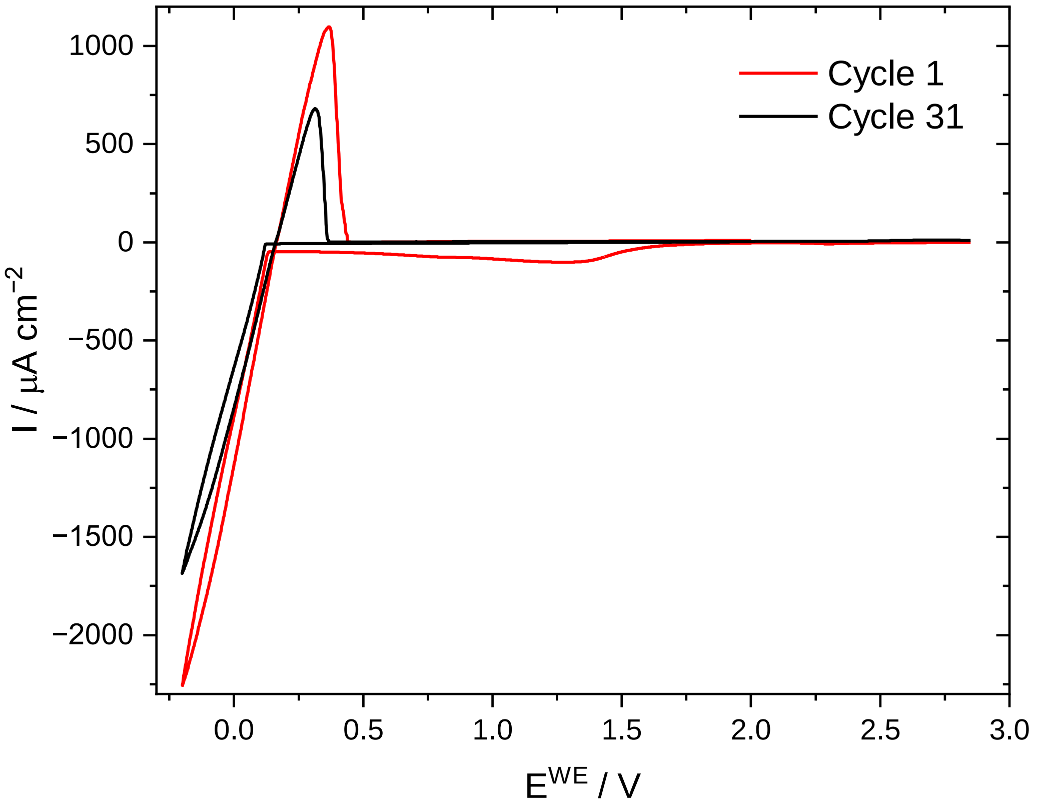

Figure S67: first and last cycle of the CV of [Li(SL)$_3$][TfNCN] with Cu working electrode. The current threshold of 10 µA/cm² was reached at +1.76 V and +0.12 V for the first and last cycle, respectively.

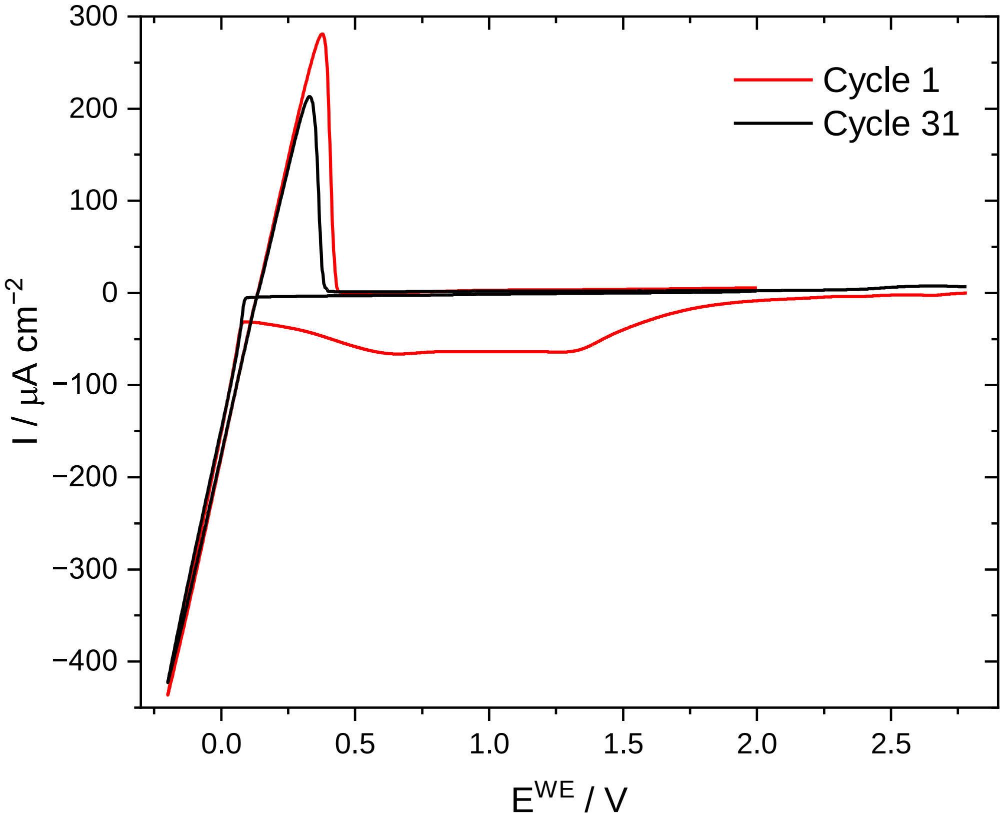

Figure S68: first and last cycle of the CV of [Li(SL)$_2$][TfNMs] with Cu working electrode. The current threshold of 10 µA/cm² was reached at +1.93 V and +0.08 V for the first and last cycle, respectively.

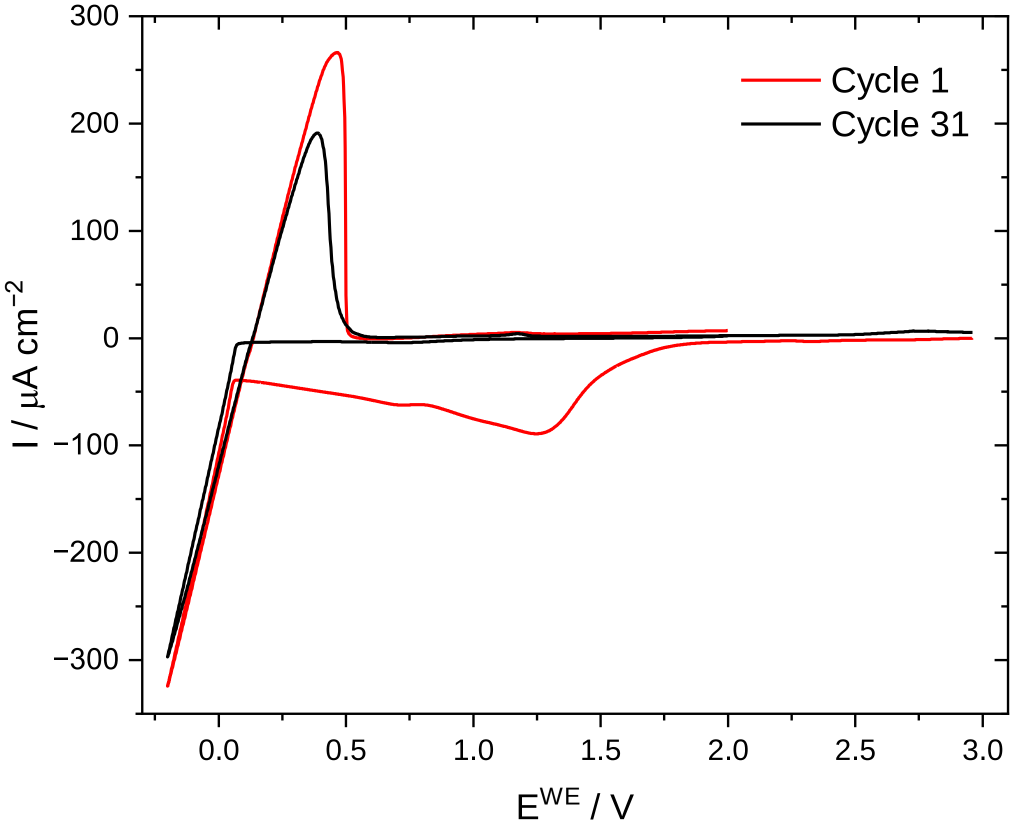

Figure S69: first and last cycle of the CV of [Li(SL)$_2$][TfNAc] with Cu working electrode. The current threshold of 10 µA/cm² was reached at +1.73 V and +0.06 V for the first and last cycle, respectively.

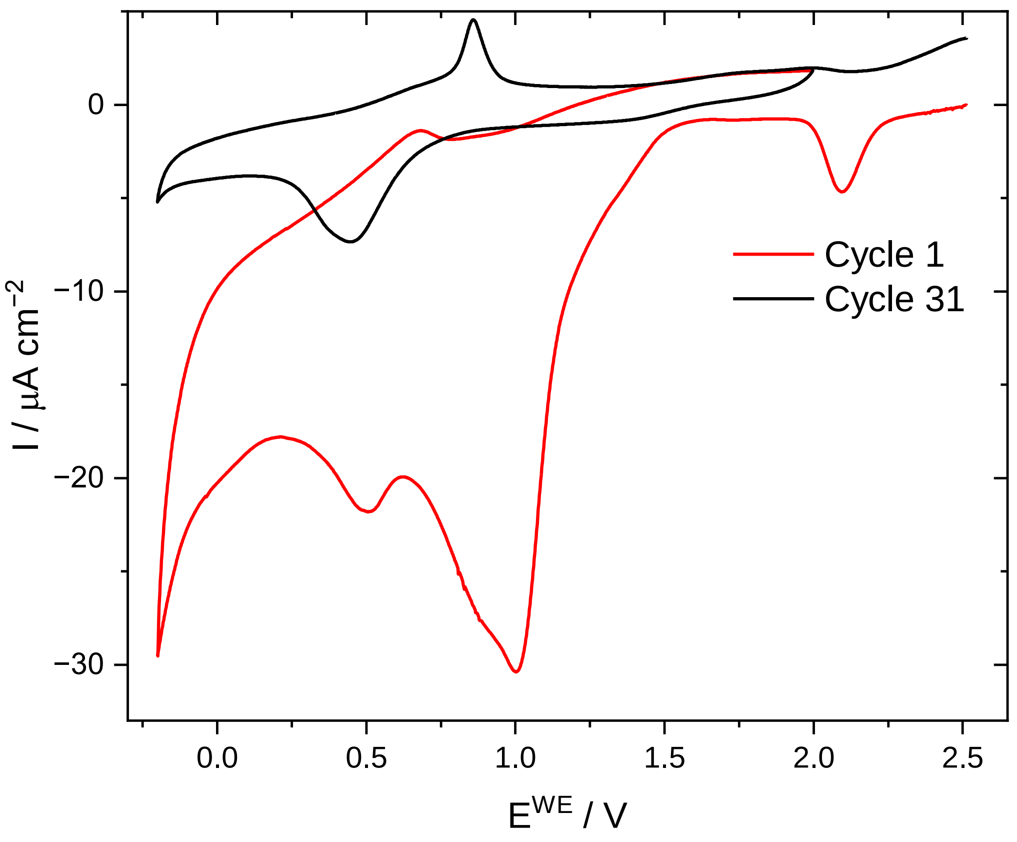

Figure S70: first and last cycle of the CV of [Li(DMSO)$_3$][MsNMs] with Cu working electrode (filtered sample). The current threshold of 10 µA/cm² was reached at +1.18 V during the first cycle.

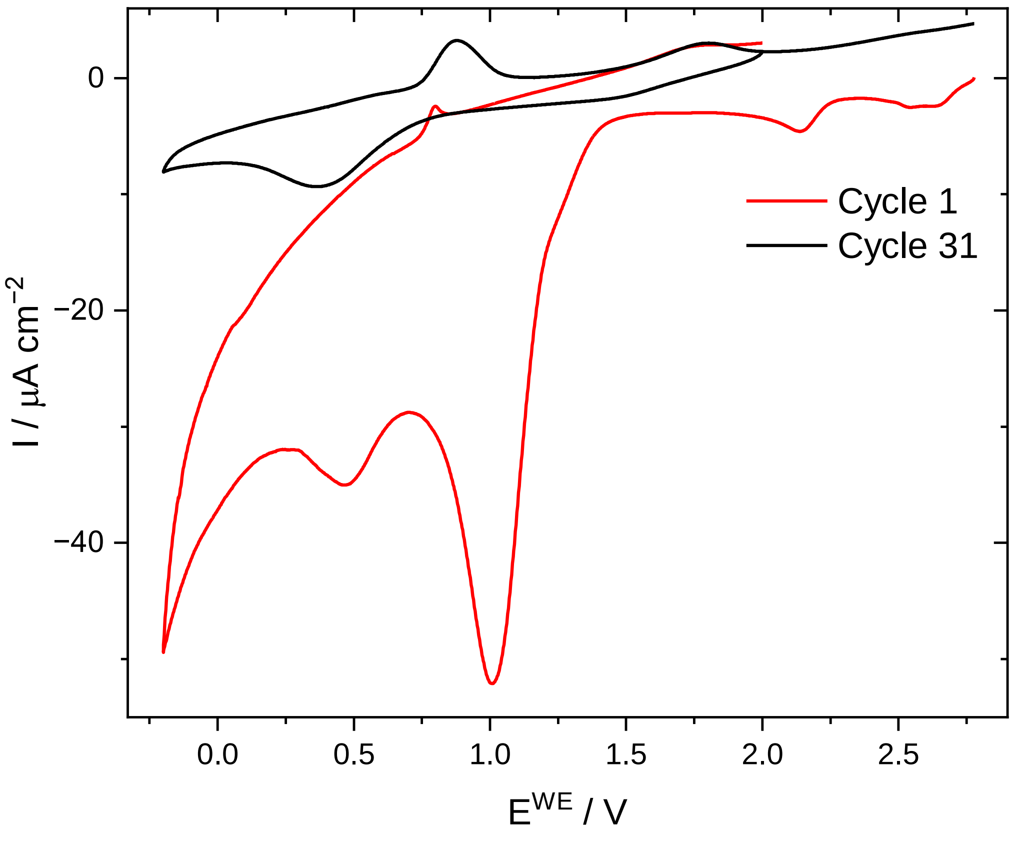

Figure S71: first and last cycle of the CV of [Li(DMSO)$_3$][MsNMs] with Cu working electrode. The current threshold of 10 µA/cm² was reached at +1.28 V during the first cycle.

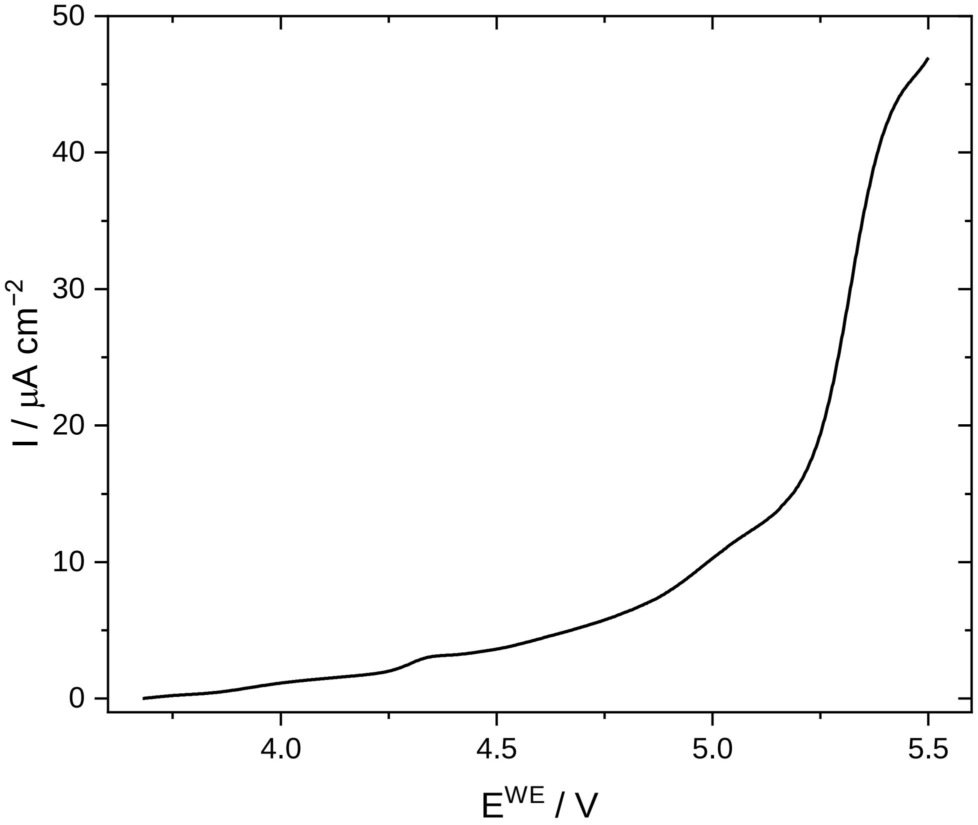

Figure S72: LSV of $[Li(SL)_2][TfNCN]$ with Pt working electrode. The current threshold of 10 µA/cm² was reached at +4.99 V.

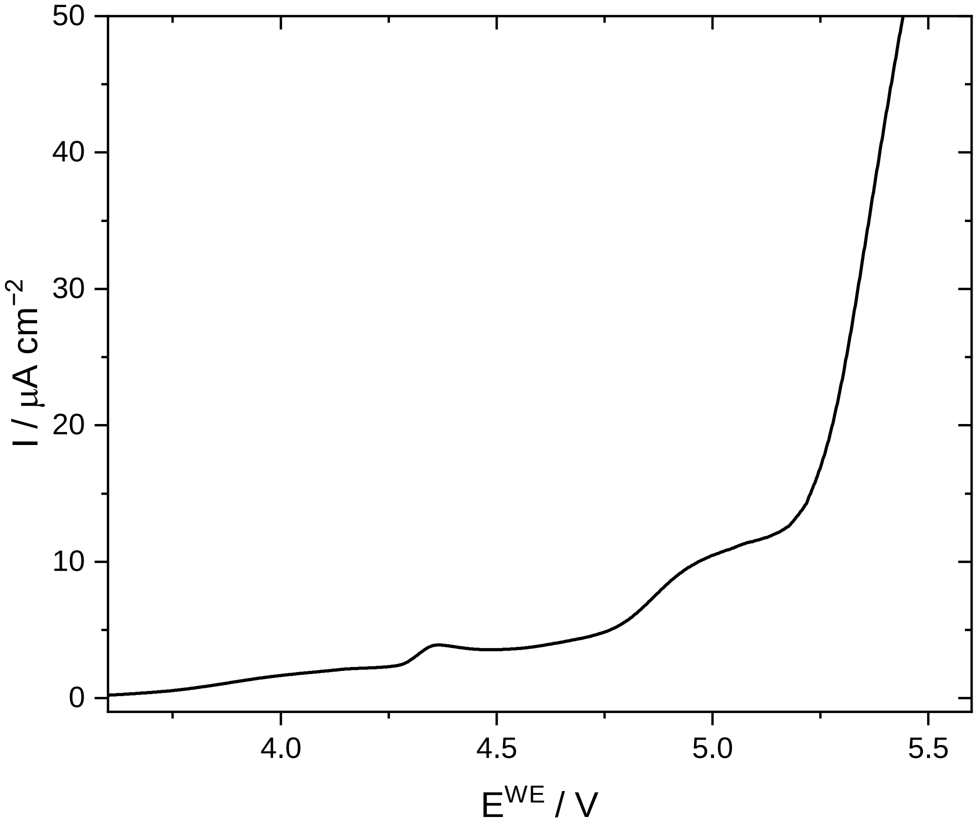

Figure S73: LSV of $[Li(SL)_3][TfNCN]$ with Pt working electrode. The current threshold of 10 µA/cm² was reached at +4.96 V.

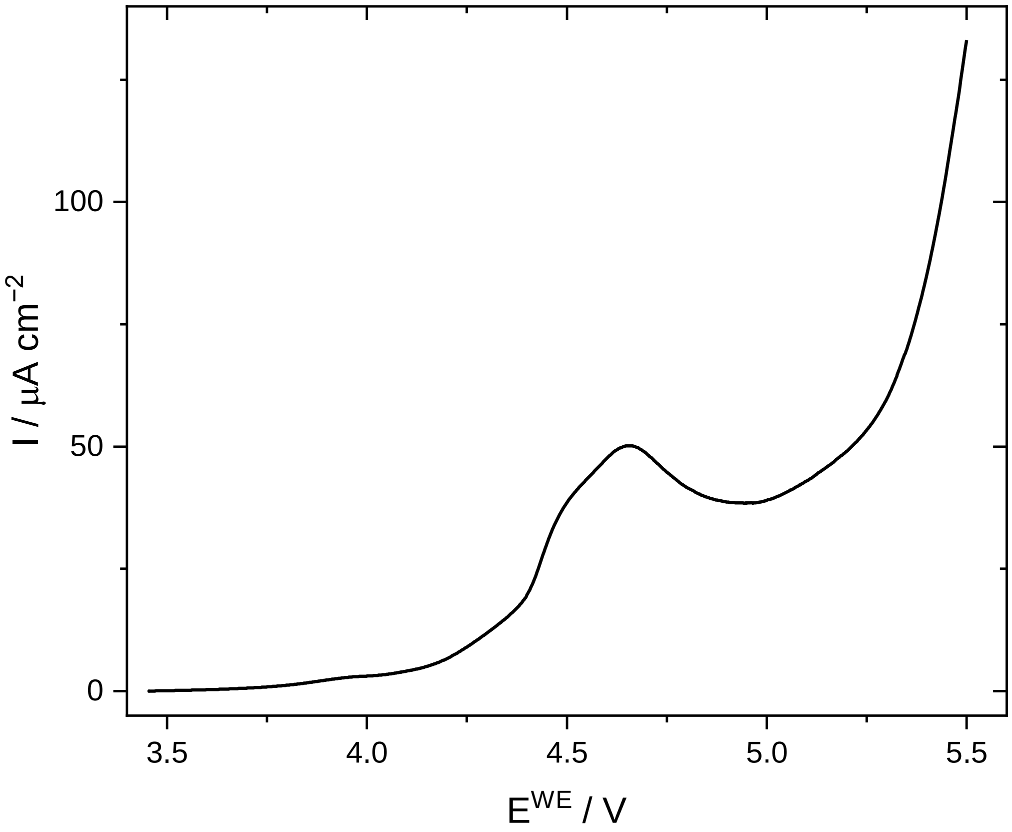

Figure S74: LSV of $[Li(SL)_2][TfNMs]$ with Pt working electrode. The current threshold of 10 µA/cm² was reached at +4.27 V.

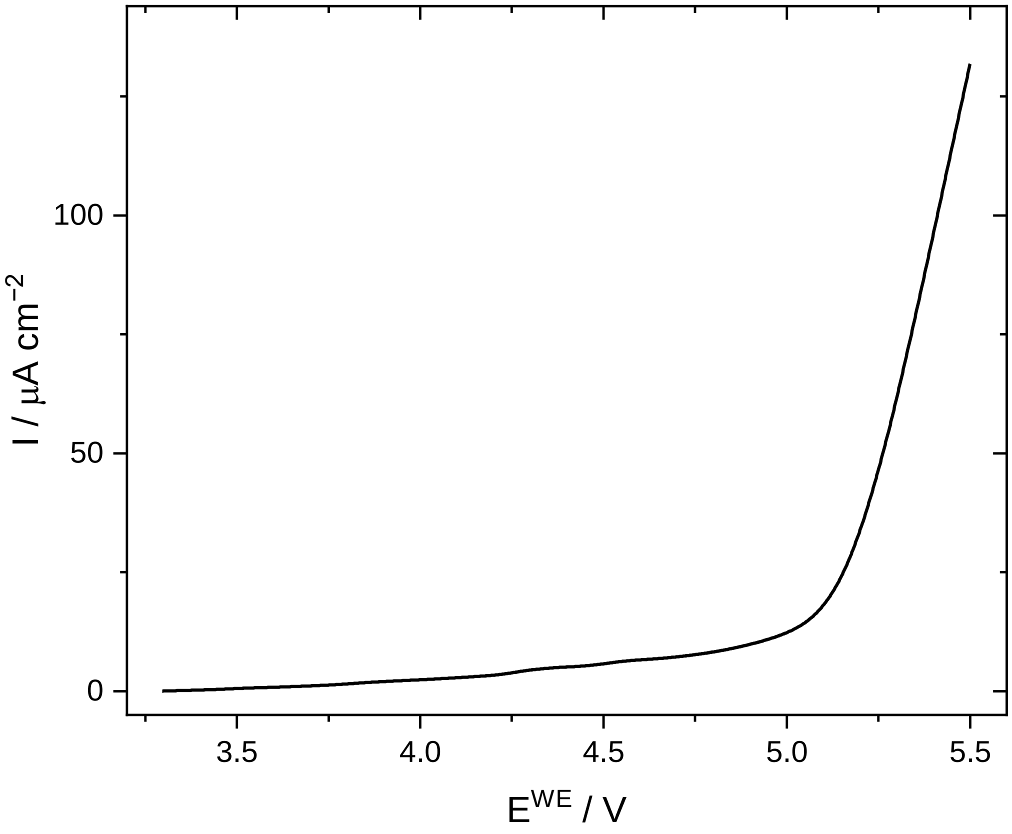

Figure S75: LSV of $[Li(SL)_2][TfNAc]$ with Pt working electrode. The current threshold of 10 µA/cm² was reached at +4.91 V.

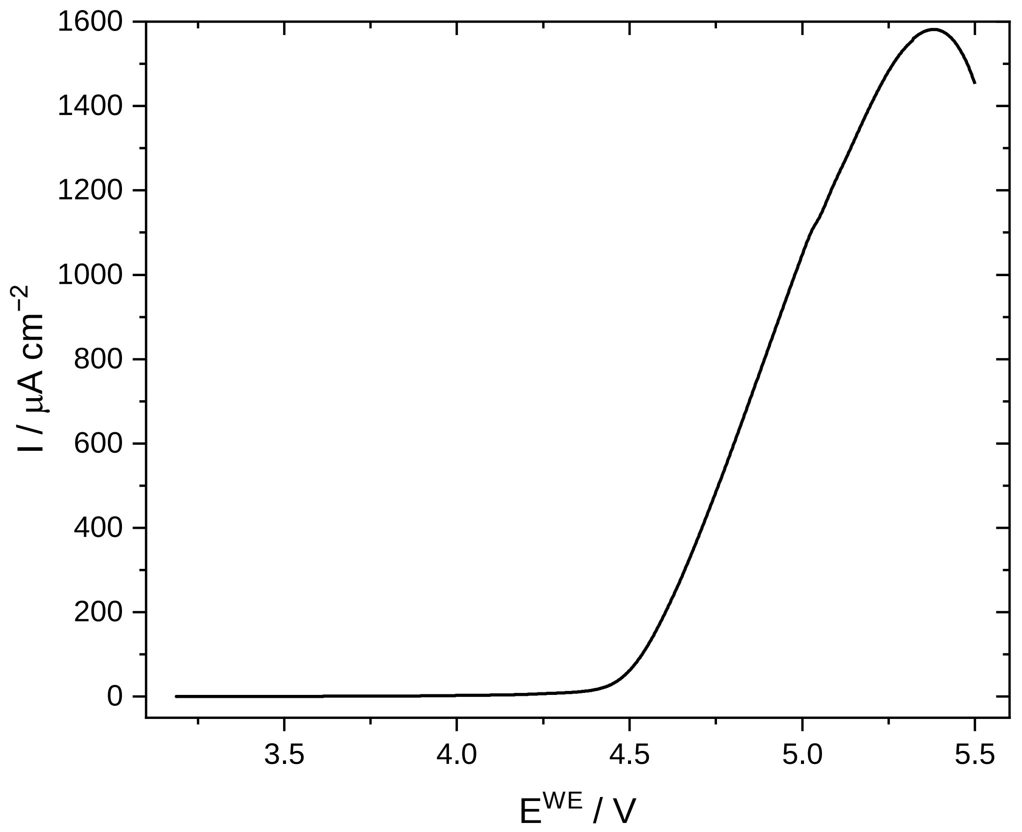

Figure S76: LSV of [Li(DMSO)$_3$][MsNMs] with Pt working electrode (filtered sample). The current threshold of 10 µA/cm² was reached at +4.33 V.

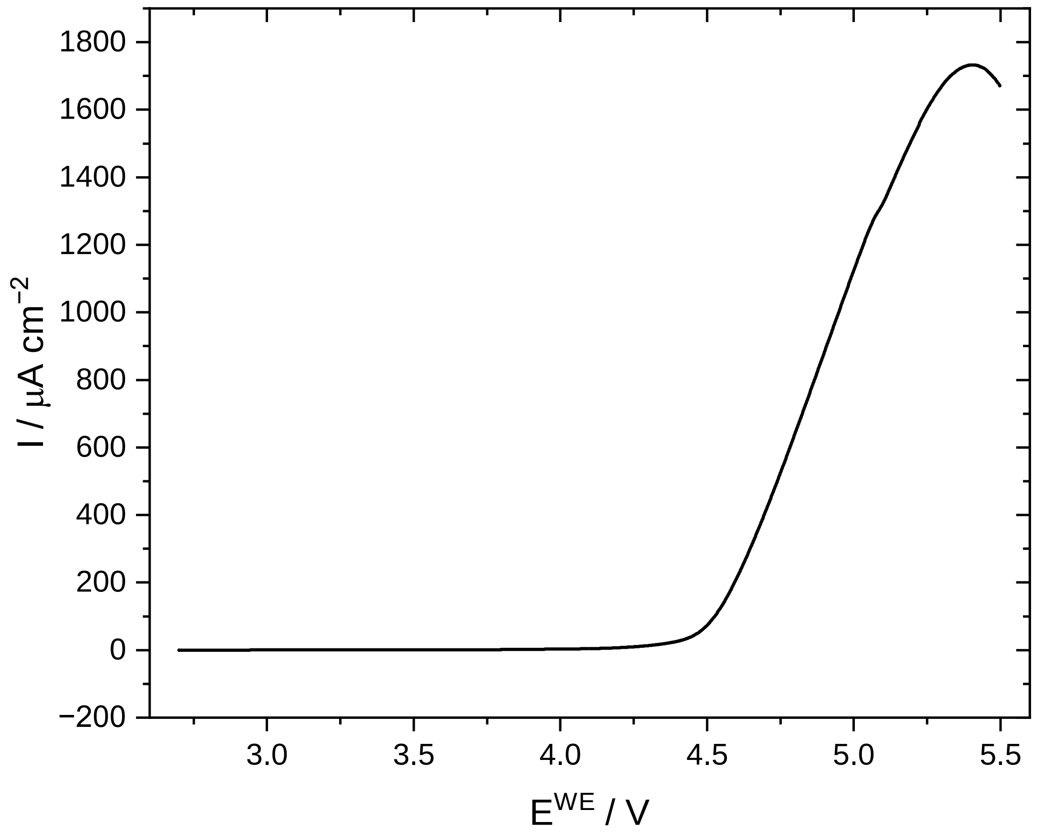

Figure S77: LSV of [Li(DMSO)$_3$][MsNMs] with Pt working electrode. The current threshold of 10 µA/cm² was reached at +4.26 V.

16. *Ab initio* calculations

Ab initio simulations were performed using the Gaussian 09 Software package, revision E.01.[71] The stabilisation energy curves in Section 17.2 were calculated at the MP2(full)/6-311++G(d,p) level of theory to be consistent with previous work. The simulations in Section 16 were performed at the MP2(full)/cc-pVTZ//B3LYP-GD3BJ/6-311+G(d,p) level of theory. Optimisations were performed with the 'verytight' convergence criteria (maximum Force 2×10^{-6}, RMS force 1×10^{-6}, maximum displacement 6×10^{-6}, RMS displacement 4×10^{-6}). SCF convergence criteria were 10^{-11} RMS change and 10^{-8} maximum change in the density matrix for MP2 single point calculations and 10^{-10} RMS change and 10^{-9} maximum change in the density matrix for all other calculations. A pruned integration grid with 99 radial shells and 590 angular points per shell was used. Stationary points were confirmed as true minima *via* frequency analysis / absence of negative eigenvalues in the Hessian. Symmetry was not used in the calculations.

16.1. Dihedral Potential Energy Scan

The potential energy as a function of dihedral angle was calculated by first optimising the structure with the relevant dihedral angle being frozen, followed by the MP2 single point calculations. Dihedral angles were scanned from 0° to 160° in steps of 5°. Surprisingly, the non-fluorinated analogues show lower barriers for the rotation and can thus be considered more conformationally flexible, Figure S78 and Figure S79

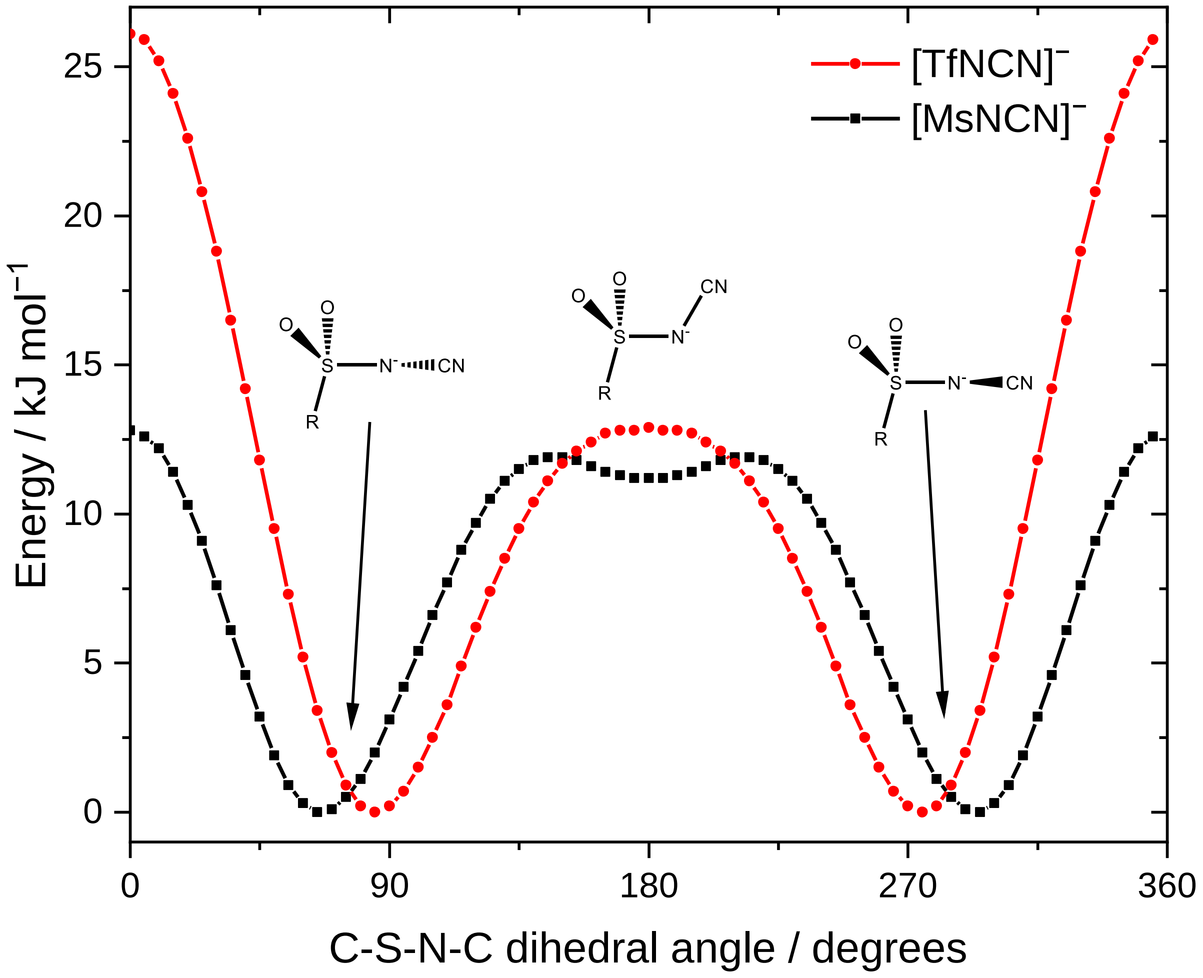

Figure S78: Energy as a function of the C-S-N-C dihedral angle for $[TfNCN]^-$ and $[MsNCN]^-$.

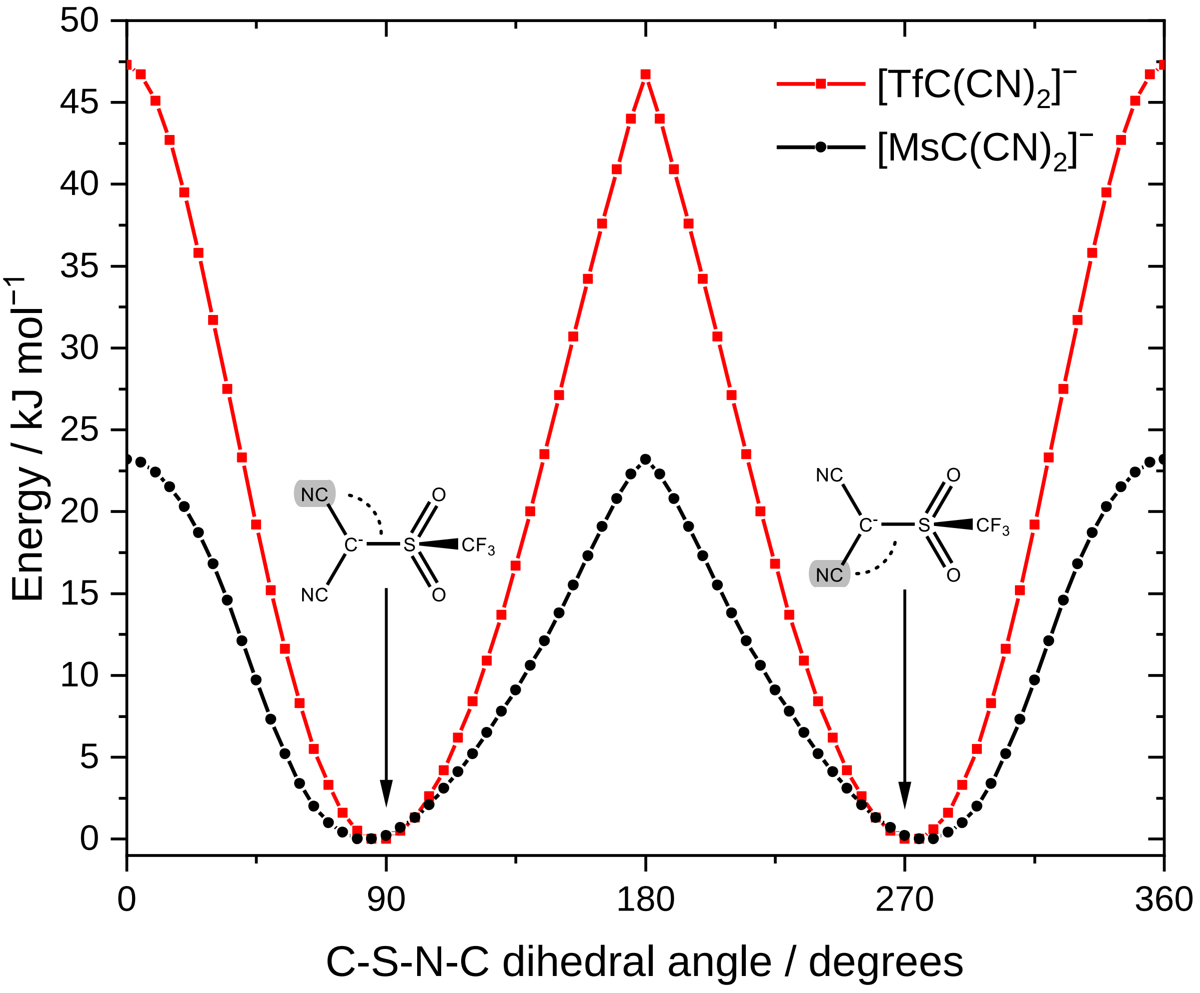

Figure S79: Energy as a function of the C-S-N-C dihedral angle for $[TfC(CN)_2]^-$ and $[MsC(CN)_2]^-$.

16.2. Acidity and Lithium Affinity

Conformers of solvents and anions were selected manually to cover the relevant conformational space.[40] For tetraglyme, a linear conformation and the most stable conformation in complex with Li^+ were considered.[72] Li^+ and H^+ were added near coordinating groups such as CN, imide nitrogen, or oxygen. These initial structures were first optimised in Cartesian coordinates in steepest decent for 40 steps, followed by the final optimisation in internal coordinates using the default Berny algorithm in Gaussian.

Here, the reaction energies for HX → H^+ + X^- and LiX → Li^+ + X^- were calculated using the MP2(full)/cc-pVTZ//B3LYP-GD3BJ/6-311+G(d,p) energy as well as the free energy and enthalpy from the B3LYP-GD3BJ/6-311+G(d,p) calculation. In addition, the stabilisation energy ΔE_{form} was calculated as described in the literature as the sum of deformation energy ΔE_{def} and interaction energy ΔE_{int}, Equation (S47) and Figure S80.[72] The deformation energy is the energetic cost of distorting the anion from its gas phase isolated geometry to the geometry in the complex. The BSSE corrected interaction energy was obtained from a Counterpoise calculation at the MP2(full)/cc-pVTZ//B3LYP-GD3BJ/6-311+G(d,p) level of theory which was performed for the Li^+ complexes.

$$\Delta E_{form} = \Delta E_{def} + \Delta E_{int} \quad \text{(S47)}$$

For some complexes, such as Li[TfN3O1], ΔE_{def} includes the energetic cost for a change in conformation, see Figure S80.

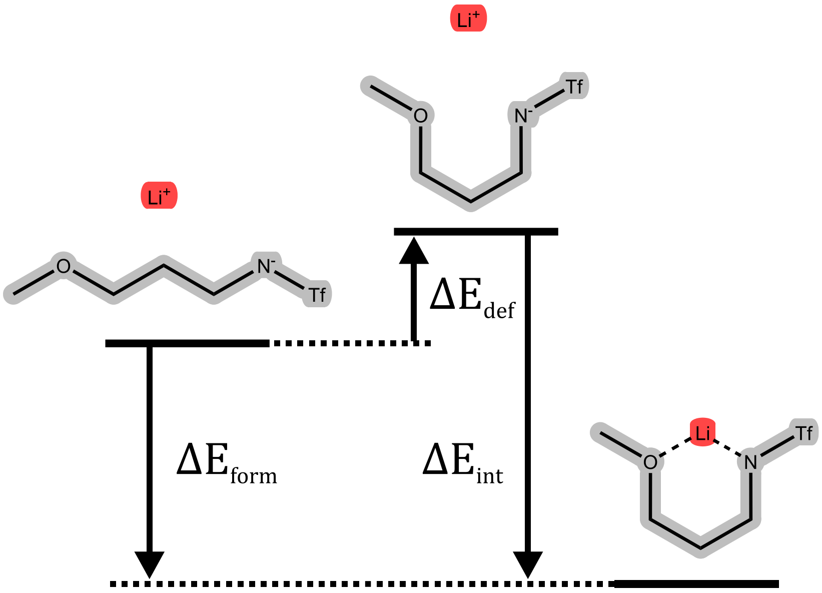

Figure S80: Illustration of the energies and their definition.

The resulting energies are given in Table S18 and Table S19. In addition, the dissociation energies are given as internal MP2 single point energy at the the MP2(full)/cc-pVTZ//B3LYP-GD3BJ/6-311+G(d,p) level of theory without any thermal corrections, and as ΔG and ΔH only at the B3LYP-GD3BJ/6-311+G(d,p) level of theory including the appropriate corrections. In addition, we performed the corresponding calculations using H^+ instead of Li^+, in which case ΔG and ΔH correspond to the gas phase basicity GPB and proton affinity PA, respectively. As a benchmark, using this method, proton affinities (gas phase basicities) of 165 (158) and 389 (383) were obtained for H_3O^+ and H_2O, respectively, in excellent agreement with established values.[73]

Table S18: Energies in kcal/mol. The reaction energies do not include BSSE corrections, but do include thermal contributions.

System name LiX	ΔE_{int}	ΔE_{def}	ΔE_{form}	Reaction energies					
				HX → H^+ + X^-			LiX → Li^+ + X^-		
				MP2	ΔG	ΔH	MP2	ΔG	ΔH
Li[OAc]	−174.9	−3.4	−171.6	361.7	337.8	346.2	180.5	163.5	171.7
Li[TfN3O1]	−174.1	−10.3	−163.8	346.1	325.8	334.2	173.4	158.1	168.2
Li[TFA]	−157.6	−3.2	−154.4	338.2	312.3	320.3	162.5	144.3	151.9
Li[TfNMe]	−157.9	−5.6	−152.3	344.8	323.9	331.9	160.3	144.9	155.8
Li[TfN5]	−156.8	−5.7	−151.1	343.3	323.9	331.4	158.8	145.9	153.8
Li[PO_2F_2]	−158.6	−7.7	−151.0	326.9	301.1	309.7	159.3	138.7	149.5
Li[CMs_3]	−154.1	−4.4	−149.6	313.4	297.5	304.7	157.6	147.4	157.5
Li[MsNCN]	−157.5	−8.7	−148.8	322.7	305.0	313.7	155.7	143.2	153.8
Li[TfNAc]	−150.5	−4.7	−145.8	331.1	311.7	319.0	152.7	139.9	147.7
Li[MsNMs]	−151.9	−7.1	−144.8	327.8	311.7	318.4	152.4	142.2	149.9
Li[TfNTFA]	−149.8	−5.7	−144.2	315.2	293.0	300.7	151.7	137.5	146.5
Li[MsNTFA]	−150.6	−6.7	−143.9	327.0	307.1	314.2	151.6	138.6	146.9
Li[BF_4]	−150.5	−7.7	−142.7	302.2	286.6	290.2	150.0	136.5	144.2
Li[OTf]	−149.3	−7.3	−142.0	312.6	292.6	299.7	150.2	135.1	143.1
Li[$PO_2(CF_3)_2$]	−151.0	−10.0	−141.0	314.6	293.9	301.4	149.3	132.8	141.3
Li[TfCHTf]	−146.7	−6.2	−140.5	316.6	299.2	307.1	148.4	137.7	145.7
Li[5cPFSI]	−144.2	−5.5	−138.7	306.3	287.6	295.0	146.8	133.8	142.3
Li[TfNMs]	−145.6	−7.1	−138.5	316.9	299.9	306.8	146.1	135.0	143.1
Li[$MsC(CN)_2$]	−143.7	−6.1	−137.5	318.7	299.1	306.6	143.8	133.9	142.2
Li[DFTFSI]	−140.5	−7.8	−132.8	310.6	292.7	299.6	140.5	129.3	136.9
Li[TfNCN]	−137.8	−6.1	−131.7	309.0	291.1	297.9	138.5	126.4	134.3
Li[CTf_3]	−135.4	−5.1	−130.3	291.3	275.7	283.0	137.9	127.8	136.2
Li[TfNFs]	−136.4	−7.0	−129.4	306.4	285.7	293.1	136.8	124.4	132.2
Li[FsNFs]	−134.5	−5.4	−129.1	305.8	283.2	290.0	136.0	121.7	129.7
Li[TfNTf]	−137.0	−8.1	−128.9	306.2	287.9	294.9	136.5	125.5	132.8
Li[PfNFs]	−135.1	−6.6	−128.5	305.2	284.8	291.9	135.9	123.1	131.0
Li[PfNPf]	−135.0	−7.4	−127.6	303.9	285.8	292.4	135.4	123.8	130.9
Li[6cPFSI]	−134.3	−7.0	−127.3	303.2	285.1	292.2	134.7	123.1	130.9
Li[SO_2CNNSO_2CN]	−126.8	−7.2	−119.7	293.4	276.8	283.6	126.1	116.3	124.2
Li[$TfC(CN)_2$]	−115.6	−2.0	−113.6	302.3	283.2	290.3	117.3	109.7	116.2

Table S19: Energies in kcal/mol for a small selection of solvents. The reaction energies do not include BSSE corrections, but do include thermal contributions.

				Reaction energies					
				$HX^+ \rightarrow H^+ + X$			$LiX^+ \rightarrow Li^+ + X$		
System name LiX	ΔE_{int}	ΔE_{def}	ΔE_{form}	MP2	ΔG	ΔH	MP2	ΔG	ΔH
$Li[G4]^+$	−119.8	−15.2	−104.6	238.2	223.3	232.2	115.6	105.1	114.7
$Li[GN]^+$	−63.9	−4.2	−59.7	196.6	184.3	192.2	63.7	54.8	63.8
$Li[DMSO]^+$	−56.5	−1.7	−54.8	221.7	209.2	216.2	59.9	54.6	61.7
$Li[SL]^+$	−58.1	−7.7	−50.4	203.5	193.2	200.1	55.4	48.6	56.3
$Li[PC]^+$	−52.6	−3.6	−49.0	205.3	190.7	198.1	52.4	46.6	53.4
$Li[THF]^+$	−43.7	−1.7	−42.0	204.8	191.6	199.6	45.5	39.5	47.2

The calculations were performed using ion pairs in a vacuum, thus some bias in the results can be expected. For example, the ion pair with the lowest interaction energy for Li[TfNCN] does not involve coordination to the nitrogen atom of the nitrile group, Figure S81. However, the gas phase optimised tetramer shows that cooperative coordination due to the low steric demand of the nitrile group is possible and leads to more negative interaction energies, in line with the clustering observed in our MD simulations. The interaction energy for the solvent (Sulfolane) is lower, *cf.* Table S19 and Figure S81e), which is expected due to the reduced electrostatic interactions, see also the SAPT2+ calculations in Section 16.5.

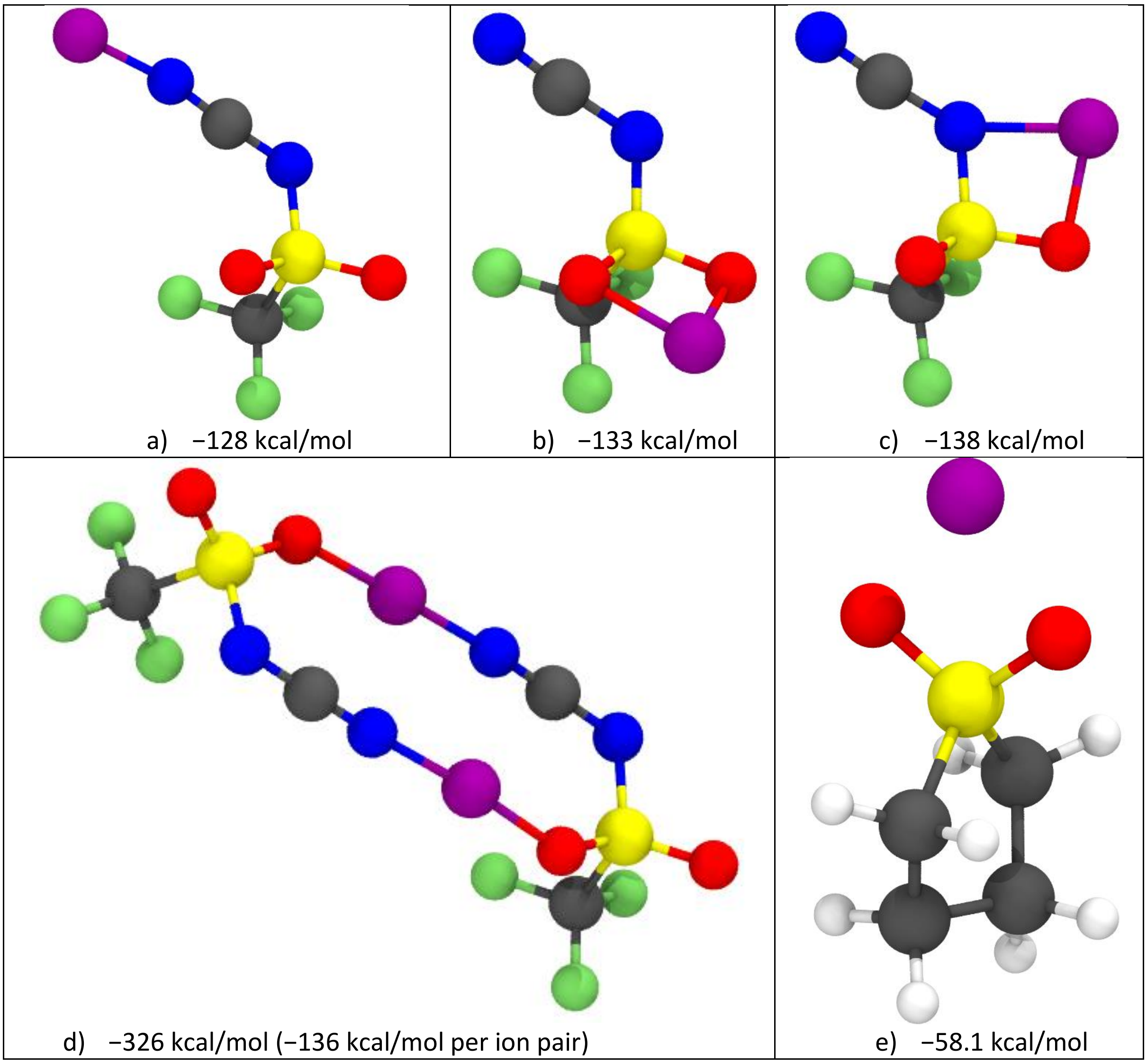

Figure S81: Structures and interaction energies ΔE_{int} of key clusters in the Li[TfNCN] system at the MP2(full)/cc-pVTZ//B3LYP-GD3BJ/6-311+G(d,p) level of theory (Counterpoise corrected).

The difference between Li^+ coordination *via* the nitrile group in Figure S81a) and *via* the SO2 group in Figure S81b) can also be analysed in the natural bond orbital (NBO) framework using the second order stabilisation energy $\Delta E^{(2)}$. The orbitals with significant contribution to $\Delta E^{(2)}$ are shown in Figure S82 and Figure S83. The sum of the second order stabilisation energies between anion and lithium is 69 kJ/mol for structure a) and 127 kJ/mol for structure b). The orbital interaction energies are thus small compared with the electrostatic interaction.

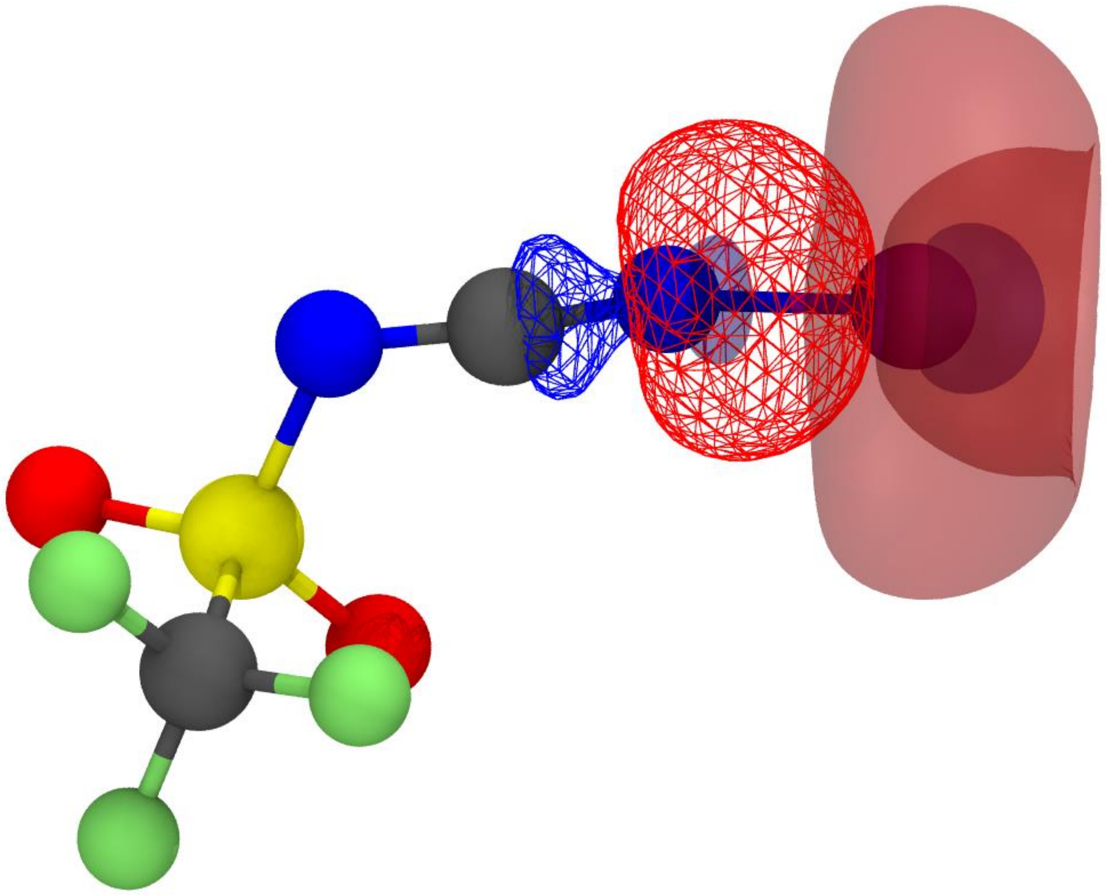

Figure S82: NBO orbitals corresponding to the nitrogen lone pair (wireframe, orbital number 44, sp hybrid) and the unoccupied lone pair orbital of the lithium atom (transparent surface, orbital number 45, $sp_{0.4}$ hybrid). For the interaction of these two orbitals, $\Delta E^{(2)}$ = 35 kJ/mol.

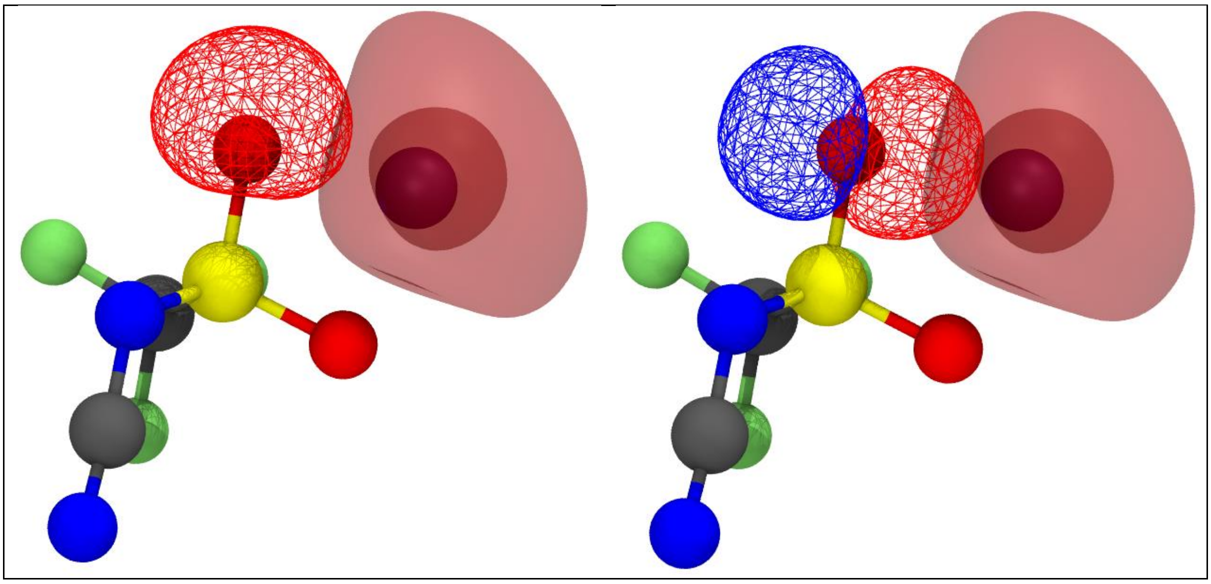

Figure S83: NBO orbitals corresponding to the lone pairs on oxygen atoms (wireframe) and the unoccupied orbital of the lithium atom (transparent surface, orbital number 45, $sp_{0.1}$ hybrid). The oxygen orbitals are only shown for one of the atoms. left = orbital number 32, $sp_{0.29}$ hybrid and right = orbital number 33, p orbital. The interaction energies with lithium are $\Delta E^{(2)}$ = 14 kJ/mol and $\Delta E^{(2)}$ = 12 kJ/mol for left and right, respectively (similar for the oxygen atom whose contributions are not shown, 14 kJ/mol and 11 kJ/mol).

16.3. Influence of Solvation

The computational results, especially the stabilisation energy, can be significantly affected by solvation, hence we performed additional simulations to estimate the impact of solvation.[74,75] Starting from the minimum energy gas phase geometries, we optimised geometries and calculated energies at the MP2(full)/cc-pVTZ//B3LYP-GD3BJ/6-311+G(d,p) level of theory but in the presence of a solvent using the Solvation Model Density (SMD) approach.[76] We chose DMSO, for which the required parameters are known, as a solvent with high donor number and relatively high dielectric constant (comparable to sulfolane).[77,78] Thus, $\Delta E_{X^-}^{solv}$ and ΔE_{LiX}^{solv} are the MP2 internal energies of the solvated species minus that of the species in the gas phase for the anions and neutral complexes with lithium, respectively, Equation (S48). Only the internal energies without entropic corrections are presented here for the sake of simplicity. Within this approximation, $\Delta E_{Li^+}^{solv} = -87.9\ \mathrm{kcal\ mol^{-1}}$, which is significantly different from the actual solvation energy of a lithium cation in DMSO from the gas phase due to the importance of an explicit solvation sphere.[79] This is expected but not problematic as it only induces a constant shift in ΔE_{form}^{solv} but does not change the qualitative trends. The results are shown in Table S20 and Figure S84.

$$\Delta E_{form}^{solv} = \Delta E_{form} + \Delta E_{LiX}^{solv} - \left(\Delta E_{X^-}^{solv} + \Delta E_{Li^+}^{solv}\right) \qquad \text{(S48)}$$

Table S20: Solvation energies and resulting stabilisation energies in the presence of the SMD model. All values in kcal mol^{-1}.

System name LiX	$\Delta E_{X^-}^{solv}$	ΔE_{LiX}^{solv}	ΔE_{form}^{solv}	System name LiX	$\Delta E_{X^-}^{solv}$	ΔE_{LiX}^{solv}	ΔE_{form}^{solv}
Li[OAc]	−58.3	−12.6	−37.9	Li[TfCHTf]	−41.6	−12.5	−23.5
Li[TfN3O1]	−50.2	−11.6	−37.3	Li[5cPFSI]	−44.5	−14.3	−20.6
Li[TFA]	−52.4	−14.1	−28.2	Li[TfNMs]	−47.2	−19.5	−22.9
Li[TfNMe]	−49.9	−13.0	−27.5	Li[MsC(CN)$_2$]	−47.9	−18.1	−19.8
Li[TfN5]	−49.8	−14.4	−27.8	Li[DFTFSI]	−46.1	−20.1	−18.8
Li[PO$_2$F$_2$]	−53.9	−13.6	−22.8	Li[TfNCN]	−48.0	−20.8	−16.5
Li[CMs$_3$]	−49.7	−20.0	−32.0	Li[CTf$_3$]	−38.5	−15.7	−19.6
Li[MsNCN]	−55.4	−17.6	−23.1	Li[TfNFs]	−44.3	−19.0	−16.1
Li[TfNAc]	−47.2	−16.1	−26.7	Li[FsNFs]	−46.6	−20.7	−15.2
Li[MsNMs]	−51.0	−20.1	−26.0	Li[TfNTf]	−42.2	−17.9	−16.6
Li[TfNTFA]	−43.0	−11.4	−24.6	Li[PfNFs]	−42.7	−18.2	−16.1
Li[MsNTFA]	−48.0	−17.6	−25.6	Li[PfNPf]	−39.2	−16.1	−16.5
Li[BF$_4$]	−58.8	−20.1	−16.1	Li[6cPFSI]	−42.2	−18.5	−15.6
Li[OTf]	−50.3	−16.4	−20.1	Li[SO$_2$CNNSO$_2$CN]	−38.7	−19.4	−12.5
Li[PO$_2$(CF$_3$)$_2$]	−45.6	−12.2	−19.6	Li[TfC(CN)$_2$]	−40.8	−31.4	−16.2

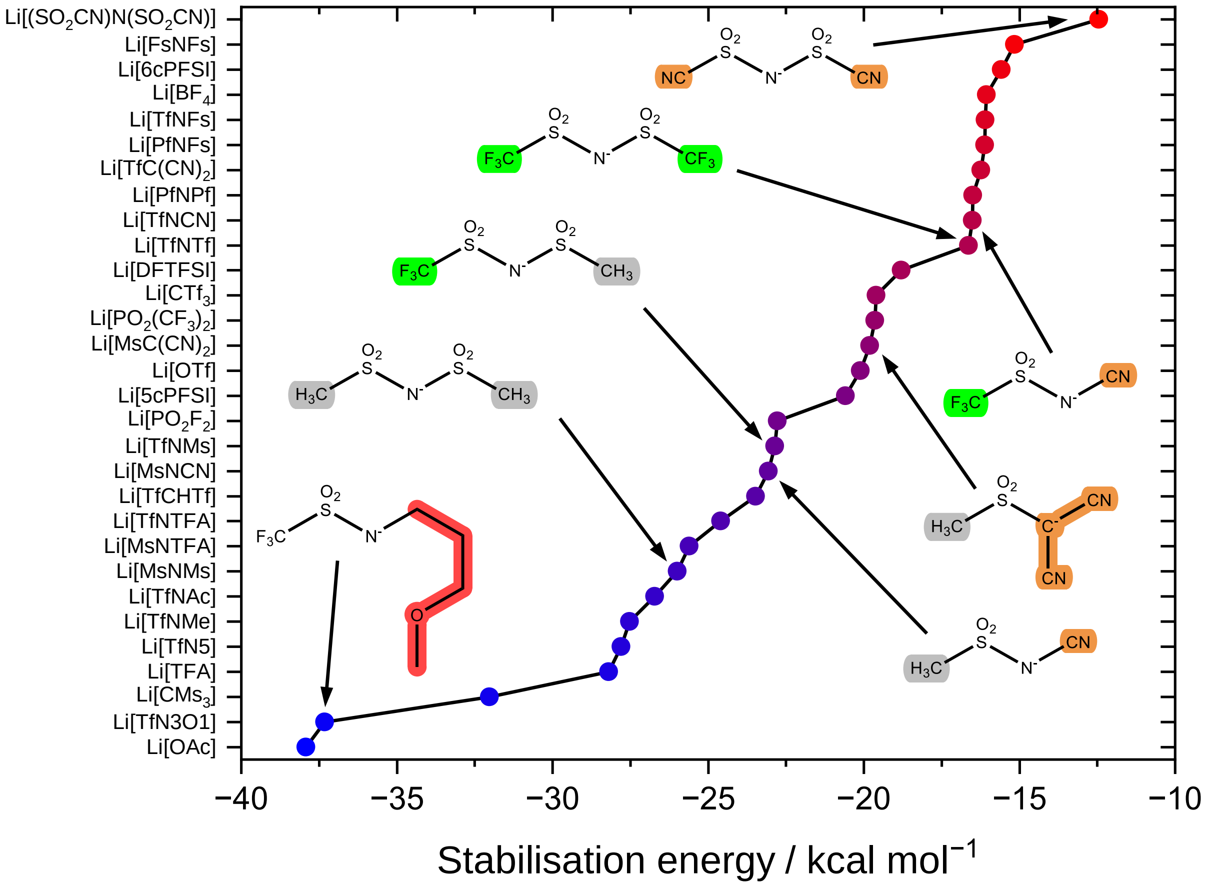

Figure S84: Stabilisation energy including solvation.

The simulation results presented in Figure S81 do not contain any solvation effect. Thus, we have also calculated the interaction energies in the presence of sulfolane as explicit solvent. To this end, we have selected cluster #3 (Lithium coordinated by one [TfNCN]$^-$ anion *via* the CN group and three sulfolane molecules), and cluster #4 (Lithium coordinated by one [TfNCN]$^-$ anion *via* the sulfonyl group and three sulfolane molecules) from the speciation analysis in Section 17.5. These two clusters were then fully optimised at the B3LYP-GD3BJ/6-311+G(d,p) level of theory, and the interaction energies ΔE_{int} calculated from Counterpoise calculations at the same level of theory. We found that the difference in energy between these two clusters was negligible relative to the accuracy of the method ($\Delta E_{int} = -102.4\ \mathrm{kcal\ mol^{-1}}$ for #3 and $-101.8\ \mathrm{kcal\ mol^{-1}}$ for #4), hence further substantiating our hypothesis that the nitrile and sulfonyl groups have a comparable coordination tendency in a realistic solvation environment due to the low steric demand of the nitrile group.

16.4. Surface charge density analysis

The statistical distribution of the electrostatic potential on the Van-der-Waals surface was calculated from the wavefunction at the MP2(full)/cc-pVTZ//B3LYP-GD3BJ/6-311+G(d,p) level of theory using the Multiwfn software package.[80–83] The grid spacing was chosen to be 0.15 Bohr, and the electrostatic potential on the surface binned into 90 bins from −160 to −30 kcal mol^{-1}. The key results are presented in Figure S85, other data are given in the accompanying files.

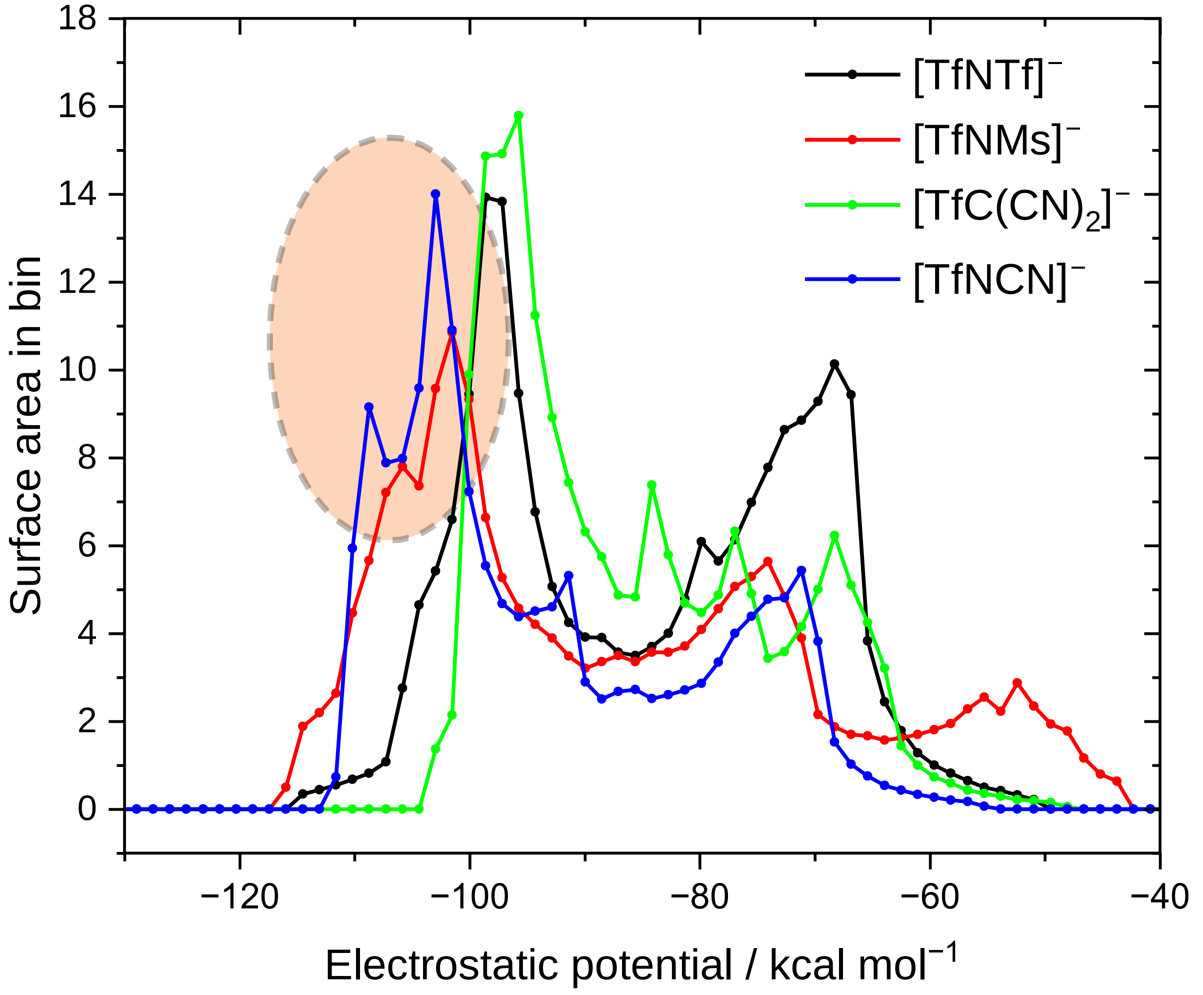

Figure S85: Statistical distribution of the surface electrostatic potential (*i.e.* absolute area of the VdW surface with a given electrostatic potential) with a bin width of 1.444 kcal mol^{-1}.

16.5. Symmetry adapted perturbation theory

SAPT2+ calculations were performed using the Psi4 software package, version 1.8.2, as described previously.[84,85] The SCF was converged with a density fitting algorithm (DF) to an energy threshold of 10^{-8}, using a aug-cc-pVDZ basis set with frozen core approximation. Four geometries were considered: the three relevant minimum energy configurations of Li[TfNCN] (with the lithium interacting with a) the nitrile-N, b) one oxygen and the imide-nitrogen atom, and c) the two oxygen atoms) and the minimum energy configuration of Li[TfNTf] (corresponding to c), with lithium being coordinated by one oxygen atom and the imide nitrogen atom). The starting geometry was the one calculated at the B3LYP-GD3BJ/6-311+G(d,p) level of theory, the total SAPT2+ interaction energies show excellent agreement (within 1 kcal mol^{-1}) with the MP2(full)/cc-pVTZ//B3LYP-GD3BJ/6-311+G(d,p) level of theory Counterpoise corrected interaction energies. The results are given in Table S21 to Table S24. For comparison, we also performed a SAPT2+ calculation for the Li^+-Sulfolane complex at the B3LYP-GD3BJ/6-311+G(d,p) equilibrium geometry, which resulted in the following energy decomposition: Electrostatic −46.2 kcal mol^{-1}, Exchange 16.2 kcal mol^{-1}, Induction −28.8 kcal mol^{-1}, Dispersion −0.5 kcal mol^{-1}.

Table S21: SAPT2+ energy decomposition for Li[TfNCN] given in kcal mol^{-1}, coordination geometry a), the distance is given relative to the starting geometry, with the lithium atom shifted rigidly along the N-Li vector.

Distance / Å	Electrostatics	Dispersion	Exchange	Induction	total
−0.7	−174.8	−7.6	360.0	−105.2	72.4
−0.6	−169.2	−5.0	248.4	−79.6	−5.4
−0.5	−161.9	−3.4	171.3	−61.8	−55.7
−0.4	−154.2	−2.2	118.3	−49.4	−87.5
−0.3	−146.7	−1.5	81.8	−40.7	−107.0
−0.2	−139.6	−1.0	56.8	−34.5	−118.4
−0.1	−133.0	−0.7	39.5	−29.9	−124.2
0.0	−126.9	−0.5	27.5	−26.5	−126.4
0.1	−121.3	−0.4	19.2	−23.6	−126.1
0.2	−116.1	−0.3	13.4	−21.2	−124.2
0.3	−111.3	−0.2	9.4	−19.1	−121.2
0.4	−106.8	−0.2	6.6	−17.2	−117.6
0.6	−98.9	−0.1	3.2	−14.0	−109.8
0.8	−92.2	−0.1	1.5	−11.3	−102.0
1.0	−86.4	0.0	0.7	−9.1	−94.8
1.5	−74.9	0.0	0.1	−5.4	−80.2
2.0	−66.4	0.0	0.0	−3.3	−69.6
3.0	−54.3	0.0	0.0	−1.4	−55.8
4.0	−46.2	0.0	0.0	−0.8	−47.0
5.0	−40.3	0.0	0.0	−0.4	−40.8
6.0	−35.8	0.0	0.0	−0.3	−36.1
8.0	−29.3	0.0	0.0	−0.1	−29.4
10.0	−24.8	0.0	0.0	−0.1	−24.9
12.0	−21.6	0.0	0.0	0.0	−21.6

Table S22: SAPT2+ energy decomposition for Li[TfNCN] given in kcal mol^{-1}, coordination geometry b), the distance is given relative to the starting geometry, with the lithium atom shifted rigidly along the S−Li vector.

Distance / Å	Electrostatics	Dispersion	Exchange	Induction	total
−0.7	−129.1	−4.1	151.2	−72.0	−54.0
−0.6	−132.0	−3.3	118.7	−64.1	−80.7
−0.5	−133.3	−2.6	92.5	−57.4	−100.8
−0.4	−133.2	−2.0	71.5	−51.7	−115.4
−0.3	−132.1	−1.6	54.8	−46.7	−125.6
−0.2	−130.2	−1.3	41.8	−42.3	−132.0
−0.1	−127.8	−1.0	31.7	−38.5	−135.6
0.0	−125.0	−0.8	24.0	−35.0	−136.9
0.1	−122.0	−0.6	18.0	−31.9	−136.5
0.2	−118.8	−0.5	13.5	−29.0	−134.8
0.3	−115.5	−0.4	10.0	−26.4	−132.2
0.4	−112.2	−0.3	7.5	−24.0	−129.1
0.6	−105.8	−0.2	4.1	−19.8	−121.7
0.8	−99.9	−0.1	2.2	−16.2	−114.0
1.0	−94.4	−0.1	1.2	−13.3	−106.6
1.5	−82.9	0.0	0.3	−8.0	−90.6
2.0	−73.7	0.0	0.1	−4.9	−78.6
3.0	−60.5	0.0	0.0	−2.0	−62.5
4.0	−51.3	0.0	0.0	−1.0	−52.3
5.0	−44.6	0.0	0.0	−0.6	−45.1
6.0	−39.4	0.0	0.0	−0.3	−39.7
8.0	−31.9	0.0	0.0	−0.1	−32.1
10.0	−26.8	0.0	0.0	−0.1	−26.9
12.0	−23.1	0.0	0.0	0.0	−23.2

Table S23: SAPT2+ energy decomposition for Li[TfNCN] given in kcal mol^{-1}, coordination geometry c), the distance is given relative to the starting geometry, with the lithium atom shifted rigidly along the S–Li vector.

Distance / Å	Electrostatics	Dispersion	Exchange	Induction	total
−0.7	−124.6	−4.0	154.0	−67.9	−42.4
−0.6	−128.2	−3.1	119.0	−60.1	−72.5
−0.5	−129.7	−2.4	91.2	−53.6	−94.5
−0.4	−129.7	−1.9	69.5	−48.0	−110.2
−0.3	−128.7	−1.5	52.6	−43.2	−120.8
−0.2	−126.9	−1.2	39.6	−39.1	−127.5
−0.1	−124.5	−0.9	29.7	−35.4	−131.1
0.0	−121.7	−0.7	22.1	−32.1	−132.4
0.1	−118.7	−0.5	16.4	−29.1	−132.0
0.2	−115.6	−0.4	12.1	−26.4	−130.4
0.3	−112.4	−0.3	8.9	−24.0	−127.8
0.4	−109.2	−0.3	6.5	−21.7	−124.7
0.6	−103.1	−0.2	3.5	−17.8	−117.5
0.8	−97.3	−0.1	1.8	−14.5	−110.1
1.0	−92.1	−0.1	1.0	−11.8	−102.9
1.5	−80.8	0.0	0.2	−7.0	−87.6
2.0	−71.8	0.0	0.0	−4.3	−76.1
3.0	−58.8	0.0	0.0	−1.8	−60.6
4.0	−49.7	0.0	0.0	−0.9	−50.7
5.0	−43.1	0.0	0.0	−0.5	−43.7
6.0	−38.1	0.0	0.0	−0.3	−38.4
8.0	−30.9	0.0	0.0	−0.1	−31.0
10.0	−26.0	0.0	0.0	−0.1	−26.1
12.0	−22.5	0.0	0.0	0.0	−22.5

Table S24: SAPT2+ energy decomposition for Li[TfNTf] given in kcal mol[−1], the distance is given relative to the starting geometry, with the lithium atom shifted rigidly along the S−Li vector.

Distance / Å	Electrostatics	Dispersion	Exchange	Induction	total
−0.7	−126.2	−4.2	149.0	−73.5	−55.0
−0.6	−129.1	−3.4	117.0	−65.6	−81.0
−0.5	−130.3	−2.7	91.0	−58.8	−100.7
−0.4	−130.2	−2.1	70.3	−53.0	−115.1
−0.3	−129.2	−1.7	53.8	−47.9	−125.0
−0.2	−127.4	−1.3	41.0	−43.5	−131.3
−0.1	−125.2	−1.1	31.0	−39.6	−134.8
0.0	−122.6	−0.8	23.3	−36.0	−136.1
0.1	−119.7	−0.7	17.5	−32.8	−135.8
0.2	−116.8	−0.5	13.0	−29.9	−134.2
0.3	−113.8	−0.4	9.7	−27.3	−131.8
0.4	−110.7	−0.3	7.2	−24.8	−128.7
0.6	−104.9	−0.2	3.9	−20.5	−121.7
0.8	−99.4	−0.1	2.1	−16.9	−114.3
1.0	−94.3	−0.1	1.1	−13.9	−107.2
1.5	−83.5	0.0	0.2	−8.6	−91.9
2.0	−74.9	0.0	0.0	−5.4	−80.3
3.0	−62.0	0.0	0.0	−2.4	−64.4
4.0	−52.7	0.0	0.0	−1.3	−54.0
5.0	−45.8	0.0	0.0	−0.7	−46.5
6.0	−40.4	0.0	0.0	−0.5	−40.9
8.0	−32.7	0.0	0.0	−0.2	−32.9
10.0	−27.4	0.0	0.0	−0.1	−27.5
12.0	−23.5	0.0	0.0	−0.1	−23.6

The total energy as a function of distance of the lithium atom from its minimum energy position is relatively similar for the three systems, Figure S86. In terms of energy contributions, the nitrile group and the N-SO_2 group show a different balance of electrostatic and induction, Figure S87.

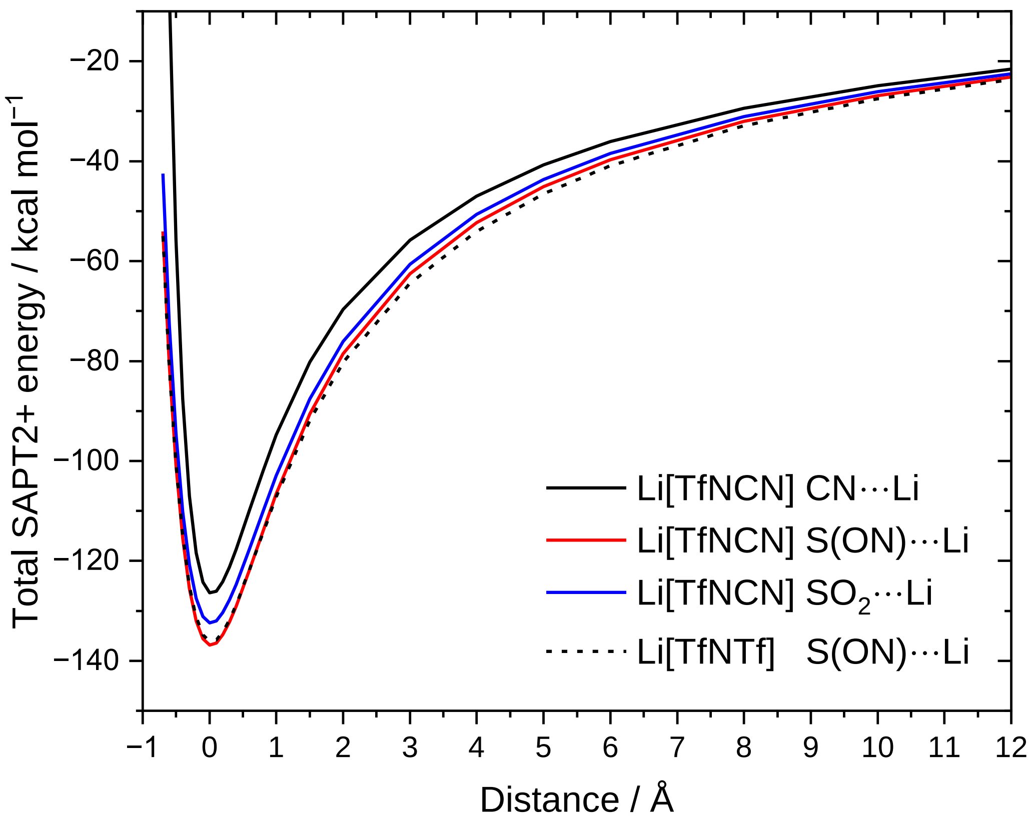

Figure S86: Total energy from the SAPT2+ calculation as a function of distance.

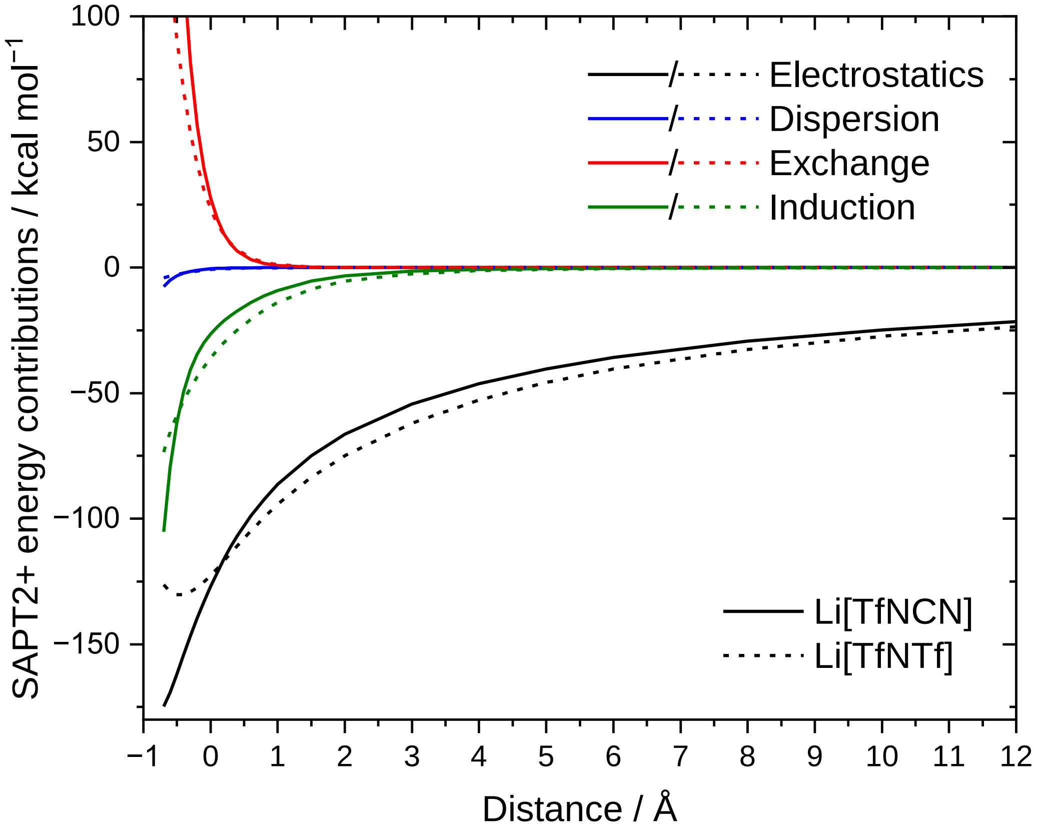

Figure S87: Contributions to SAPT2+ energy.

17. MD Simulation Details

MD simulations were carried out in the NPT ensemble using the MPDynPFF software package (in-house).[86] The system temperature and pressure were maintained at 403 K and 0.1 MPa using a Nosé-Hoover chain thermostat[87–89] and an Andersen barostat,[90] respectively, and time constants for the thermostat and the barostat were set at 0.5 ps and 2 ps, respectively. All C–H bonds were held rigid using the SHAKE/RATTLE algorithm,[91] and the reversible RESPA was employed for the multiple time step integration of the equations representing the motion of atoms.[92,93] The Lennard-Jones interaction was truncated at 12 Å, whilst long-range electrostatic and induced polarisation interactions were estimated using the Ewald method.[94] Pair nonbonded interactions between atoms separated by one or two bonds were not considered (scaled with 0.0), while 1-4 interactions (atoms separated by three bonds) were scaled with 0.5.[95,96] The time step size for updating electrostatic interactions in the Ewald reciprocal space was 8 fs, whilst that for the other interactions was 2 fs. Periodic boundary conditions were employed for all three dimensions.

To reduce any biases arising from the initial arrangement of molecules, following procedures were carried out for preparation of the initial structures. The system was initially allowed to evolve at 453 K and 10 MPa from the low-density condition of the initial box size (*i.e.*, $2.7 \cdot 10^4$ nm^3) for 50 ps, and was subsequently equilibrated at 453 K and 0.1 MPa for 1 ns. Additional 1 ns equilibration MD runs were then carried out at 403 K without changing the pressure, and a 100 ns production run was performed collecting the trajectory data at 0.2 ps intervals for analysis. The TRAVIS software package (version Jul 29 2022), was used for trajectory and cluster analysis.[97–99] The number of molecules in a cubic cell (ca. ≈ 3 nm side length) was chosen as follows for consistency purposes: Li[TfNCN]/SL = 70/140, Li[TfNTf]/SL = 70/140, and Li[TfNCN]/Li[TfNTf]/SL = 35/35/140.

For the purpose of these simulations, the OPLS–AA-based polarisable force field was employed, as described in our previous report[56,67] and in Table S25 to Table S28 and Figure S88. For structural analysis, visualisation of the structures was carried out using VMD software.[100]

17.1. Force field parameters

The force field parameters were modified to reproduce molecular structures and the stabilisation energy of Li^+–solvent and Li^+–anion complexes optimised at the MP2(full)/6-311++G(d,p) level of theory based on the OPLS-based parameters obtained from literature.[56,96,101–103] Atomic charges of the molecules were determined based on the atomic charges obtained by electrostatic potential fitting[104,105] based on the MP2/6-311++G(d,p) wave functions.

The total potential was defined by the following equation. Detailed information is available in our previous report.[67]

$$\begin{aligned}(\boldsymbol{r}^N) = &\sum_{i=1}^{N_{\text{bond}}} \frac{1}{2}k_b(r-r_0)^2 + \sum_{i=1}^{N_{\text{angle}}} \frac{1}{2}k_\theta(\theta-\theta_0)^2 + \sum_{i=1}^{N_{\text{torsion}}} \frac{1}{2}k_t(1+\cos(n\varphi+\delta)) + \\ &\sum_{i=1}^{N}\sum_{j>i}^{N} 4\varepsilon\left\{\left(\frac{\sigma}{r_{ij}}\right)^{12} - \left(\frac{\sigma}{r_{ij}}\right)^{6}\right\} + \sum_{i=1}^{N}\sum_{j>i}^{N} \frac{q_i q_j}{4\pi\varepsilon_0 r_{ij}} + U_{\text{ind}}(\boldsymbol{r}^N)\end{aligned} \tag{S49}$$

Here, the first three terms describe the intramolecular interactions and the last three terms describe the nonbonded interactions, respectively. Here, k_b, k_θ and k_t are force constants, r_0 and θ_0 are the equilibrium bond length and angle at the energy minimum, n is the number of waves, δ is the phase, ε is the potential depth at the minimum, σ is the distance at which the potential is zero, ε_0 is the

vacuum permittivity and q_i is the partial charge on *i*-th atom, respectively. The final term is the induction term, which is based on the isotropic atomic induced dipole model and described by the following equation.

$$U_{\text{ind}}(\boldsymbol{r}^N) = -\sum_{i=1}^{N} \mu_i \cdot E_i^0 - \frac{1}{2}\sum_{i=1}^{N}\sum_{j=1}^{N} \mu_i \cdot T_{ij} \cdot \mu_j + \sum_{i=1}^{N} \frac{\mu_i \cdot \mu_i}{2\alpha_i} \tag{S50}$$

μ_i ($= \alpha_i E_{\text{Total}}$) is the induced dipole moment, α_i is the isotropic atomic dipole polarizability of *i*-th atom. E_{Total} is the total electric field. E_i^0 is the electric field created by partial atomic charges, and T_{ij} is the second order dipole tensor.[67,106]

Table S25: Bond stretching parameters.

Bond	k_b / kcal mol^{-1} Å^{-2}	r_0 / Å
CS–CS	268.000	1.529
CS–HC	340.000	1.090
SFO–OFO	1274.000	1.459
SFO–CS	471.000	1.823
CF–FC	884.000	1.340
SO–CF	471.000	1.835
SO–OS	1274.000	1.450
NI–SO	744.000	1.600
NI–CCY	1005.200	1.310
CCY–NCY	1300.000	1.157

Table S26: Angle bending parameters.

Angle	k_θ / kcal mol^{-1} rad^{-2}	θ_0 / deg.
CS–CS–CS	58.35	112.7
CS–CS–HC	37.50	110.7
HC–CS–HC	33.00	107.8
OFO–SFO–OFO	100.00	115.0
OFO–SFO–CS	100.00	102.6
SFO–CS–CS	166.00	110.4
SFO–CS–HC	37.43	110.7
CS–SFO–CS	195.00	94.0
FC–CF–FC	187.00	108.6
SO–CF–FC	166.00	110.4
OS–SO–OS	232.00	120.2
OS–SO–NI	189.00	111.4
CF–SO–OS	208.00	102.6
CF–SO–NI	195.00	100.2
SO–NI–SO	80.00	121.0
NI–CCY–NCY	101.60	175.2
SO–NI–CCY	45.75	118.6

Table S27: Torsional parameters.

Dihedral	k_t / kcal mol^{-1}			δ / degrees
	$n = 1$	$n = 2$	$n = 3$	
OFO–SFO–CS–CS	0.0	0.0	0.0	0.0
OFO–SFO–CS–HC	0.0	0.0	0.0	0.0
SFO–CS–CS–CS	0.363	0.217	−0.098	0.0
SFO–CS–CS–HC	0.0	0.0	0.0	0.0
CS–SFO–CS–CS	1.46	−1.073	0.279	0.0
CS–SFO–CS–HC	0.0	0.0	0.0	0.0
CS–CS–CS–CS	1.743	0.157	0.279	0.0
CS–CS–CS–HC	0.0	0.0	0.366	0.0
HC–CS–CS–HC	0.0	0.0	0.318	0.0
FC–CF–SO–NI	0.0	0.0	0.0	0.0
FC–CF–SO–OS	0.0	0.0	0.171	0.0
SO–NI–SO–CF	7.833	0.500	−0.764	0.0
SO–NI–SO–OS	0.0	0.0	−0.004	0.0
CF–SO–NI–CCY	6.268	5.274	−0.292	0.0
SO–NI–CCY–NCY	0.0	0.0	0.0	0.0
OS–SO–NI–CCY	0.0	0.0	0.366	0.0

Table S28: Nonbonding parameters.

Atom	σ / Å	ε / kcal mol^{-1}	α / a.u.
CS	3.50	0.066	9.000
HC	2.50	0.030	2.000
OFO	3.20	0.050	7.200
SFO	3.55	0.350	16.000
LI	2.58	0.003	0.000
CF	3.50	0.066	9.000
FC	2.95	0.054	2.500
OS	3.08	0.130	5.000
SO	3.55	0.250	16.000
NI	3.45	0.170	8.000
CCY	3.30	0.066	9.000
NCY	3.22	0.170	8.000

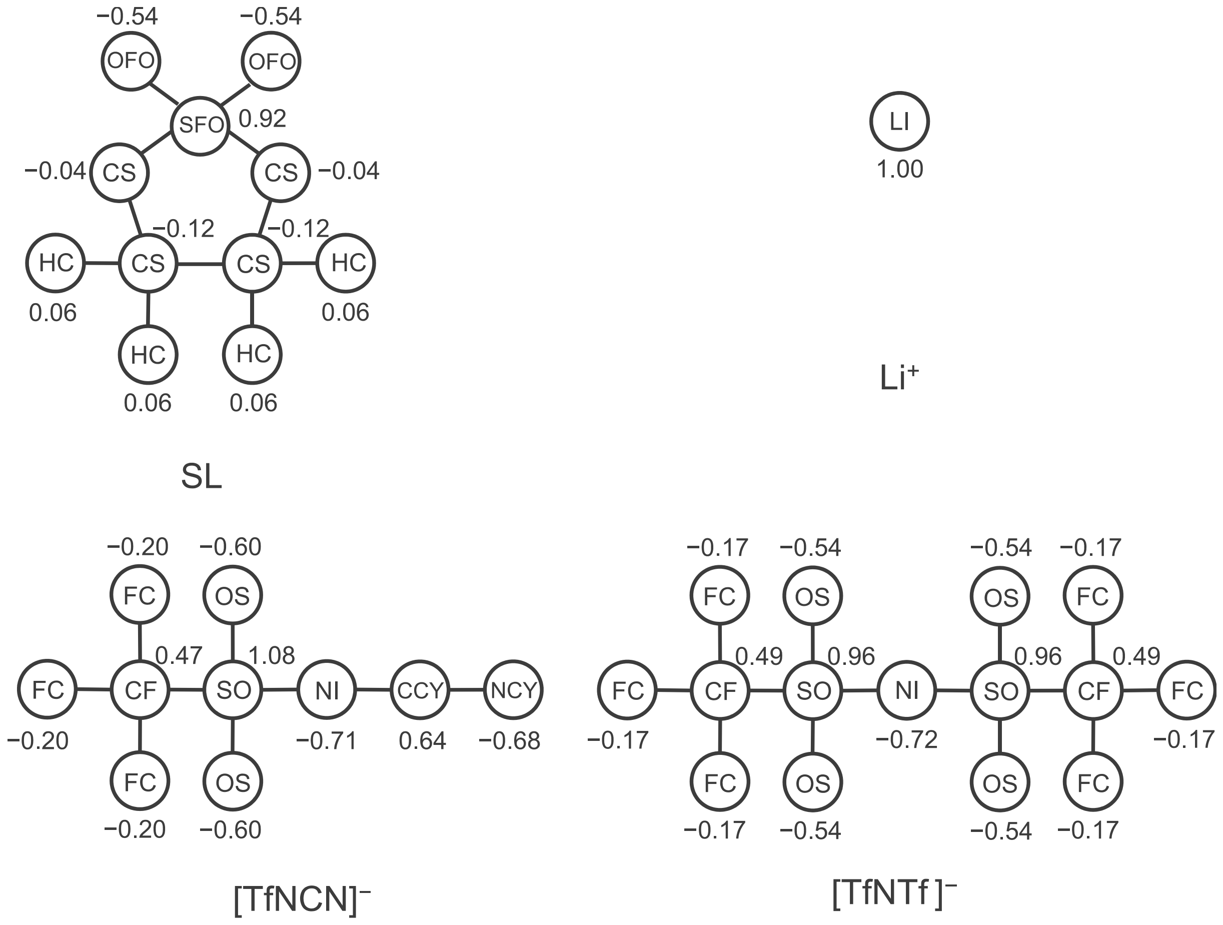

Figure S88: Atom types and atomic charges, q, of SL, Li^+ and anions used for the MD simulation.

17.2. Parameterisation of the $[TfNCN]^-$ anion

Angle bending

The S–N–C angle bending parameter was determined by fitting the MP2(full)/cc-pVTZ//B3LYP-GD3BJ/6-311+G(d,p) level of theory energy with the harmonic potential, *cf.* Equation (S49), Figure S89.

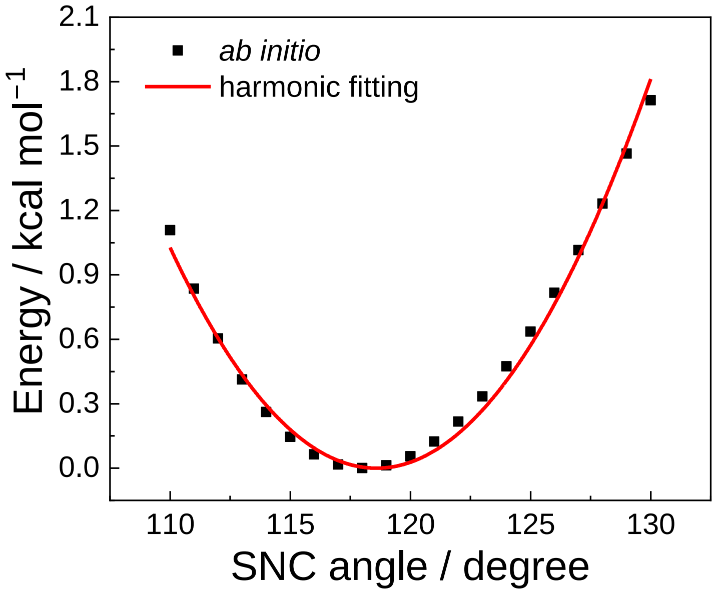

Figure S89: Harmonic fit of the of S–N–C angle bending energy.

Dihedral torsions

Since C–S–N–C dihedral torsion dominates the flexibility of $[TfNCN]^-$, the corresponding force field parameter was optimised by fitting the difference between the *ab initio* target potential energy calculated at the MP2(full)/cc-pVTZ//B3LYP-GD3BJ/6-311+G(d,p) level of theory and the energy of the force field without the nonbonded contribution to be fitted (*i.e.* here the C–S–N–C dihedral contribution) with the OPLS–AA-based function (described as the third term of Equation (S49)) to reproduce the energy to within ≈1 kcal mol^{-1}, Figure S90 and Figure S91.[107–109]

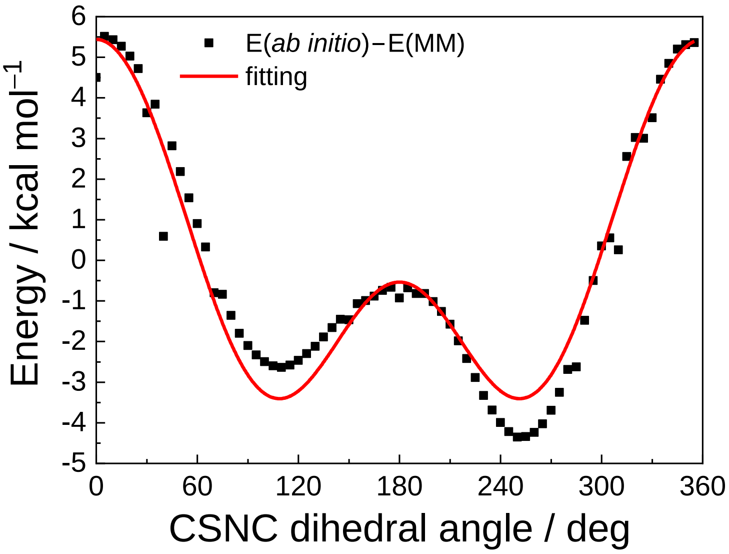

Figure S90: The target function to be fitted – *i.e.* the difference between the *ab initio* and force field energies – and the OPLS fit.

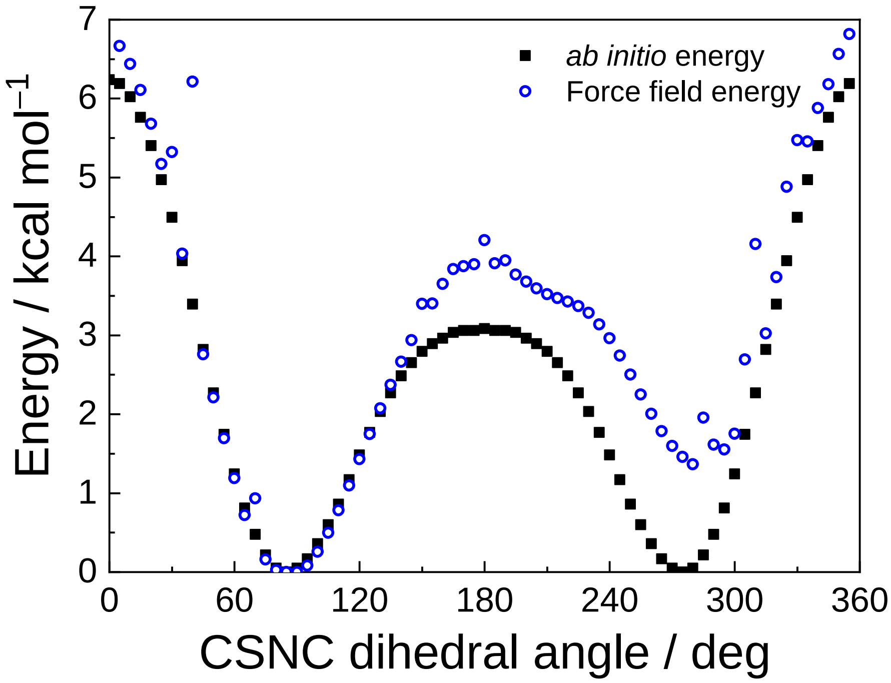

Figure S91: Comparison of *ab initio* and force field energy as a function of the torsion angle.

To evaluate the performance of the final force field, we recorded the dihedral angles of every anion throughout the production run (3.5×10^7 occurrences) with a bin width of 5 degrees. Assuming a Boltzmann distribution at 298.15 K, the energy profile shown in Figure S92 is obtained. The agreement with the *ab initio* calculation is excellent, thus confirming the successful parameterisation. Furthermore, no significant distortion due to the anion being embedded in the bulk is observed, which is consistent with our previous work.

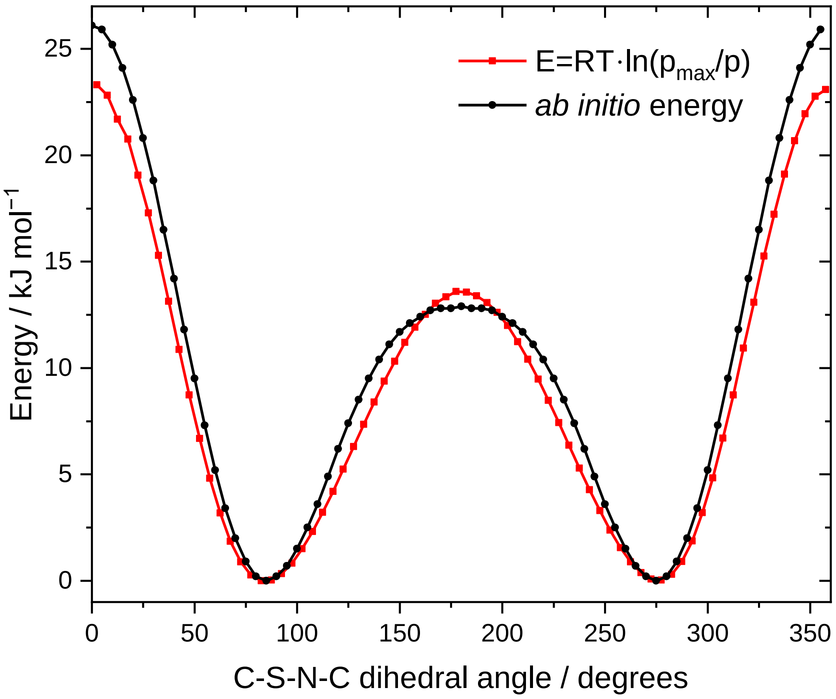

Figure S92: *Ab initio* energy profile from the static calculation in the gas phase in direct comparison with the potential of mean force obtained from the distribution of dihedral angles during the MD simulation.

Stabilisation energy of Li^{+}–$[TfNCN]^{-}$

Finally, all force field parameters were modified to reproduce the MP2(full)/6-311++G(d,p) level optimised molecular structures and stabilisation energies of the Li^{+}–$[TfNCN]^{-}$ pair as a function of distance between Li^{+} and possible coordination sites of $[TfNCN]^{-}$ within ≈1 kcal mol^{-1}.

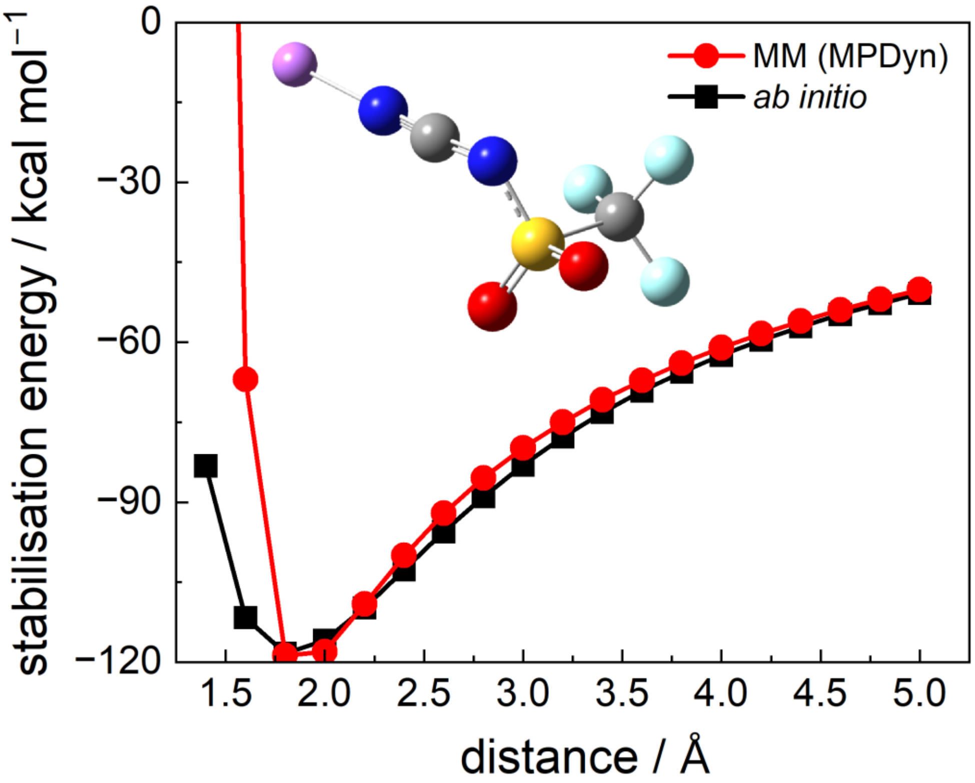

Figure S93: Stabilisation energy in the Li^{+}–$[TfNCN]^{-}$ ion pair as a function of distance between Li^{+} (purple sphere in the CPK model in the insert) and nitrogen atom of the nitrile group.

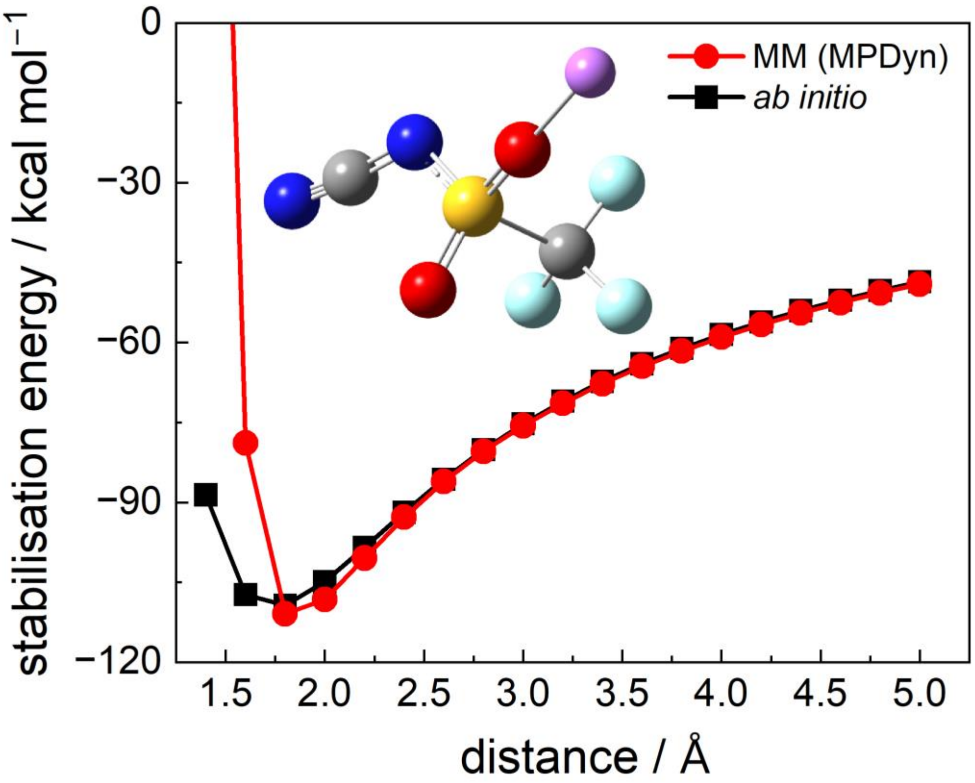

Figure S94: Stabilisation energy in the Li⁺–[TfNCN]⁻ ion pair as a function of distance between Li⁺ (purple sphere in the CPK model in the insert) and the oxygen atom of the SO_2 group. Here, the lithium ion coordinates the oxygen atom in opposite direction to the NCN motif.

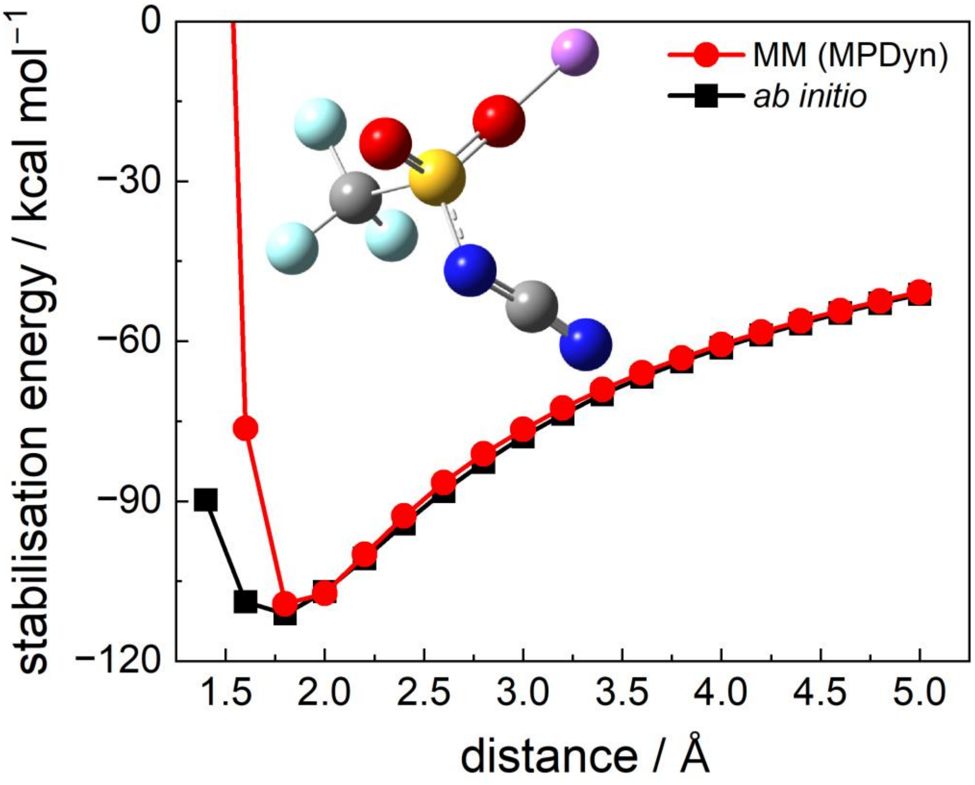

Figure S95: Stabilisation energy in the Li⁺–[TfNCN]⁻ ion pair as a function of distance between Li⁺ (purple sphere in the CPK model in the insert) and the oxygen atom of the SO_2 group. Here, the lithium ion coordinates the oxygen atom on the same side as the NCN motif.

17.3. Dynamic properties

We used the prealpha software package to calculate dynamic properties (source code, examples, binaries, and manual can be found under https://github.com/FPhilippi/prealpha). The main purpose was to check that the timescales of relaxations are short compared to the length of the simulation to ensure sufficient equilibration.

The α_2 parameter was calculated as shown in Equation (S51).[14,67,110–113]

$$\alpha_2(t) = \frac{3\langle(\Delta r)^4\rangle}{5\langle(\Delta r)^2\rangle^2} - 1 \tag{S51}$$

Here, Δr is the distance which the centre of mass of a tagged molecule travelled during the time t. Cornered brackets denote an ensemble average over the 100 ns trajectory.

Furthermore, we calculated the intermittent dihedral autocorrelation function, Equation (S52).[14,114,115]

$$C_{dihedral}(t) = \frac{\langle(h(t) - \langle h\rangle)(h(0) - \langle h\rangle)\rangle}{\langle(h(0) - \langle h\rangle)^2\rangle} \tag{S52}$$

Here, h is a binary function tracking the conformational state of the anions. For the $[TfNCN]^-$ anion, the function is 1 when the C-S-N-C dihedral angle is between 0 and 180 degrees. For the $[TfNTf]^-$ anion, the function is 1 when both C-S-N-S backbone dihedral angles are either between 0 and 150 degrees, or when both dihedral angles are between 210 and 360 degrees. This corresponds to the *trans* conformer.[14] In all other cases, h evaluates to zero.

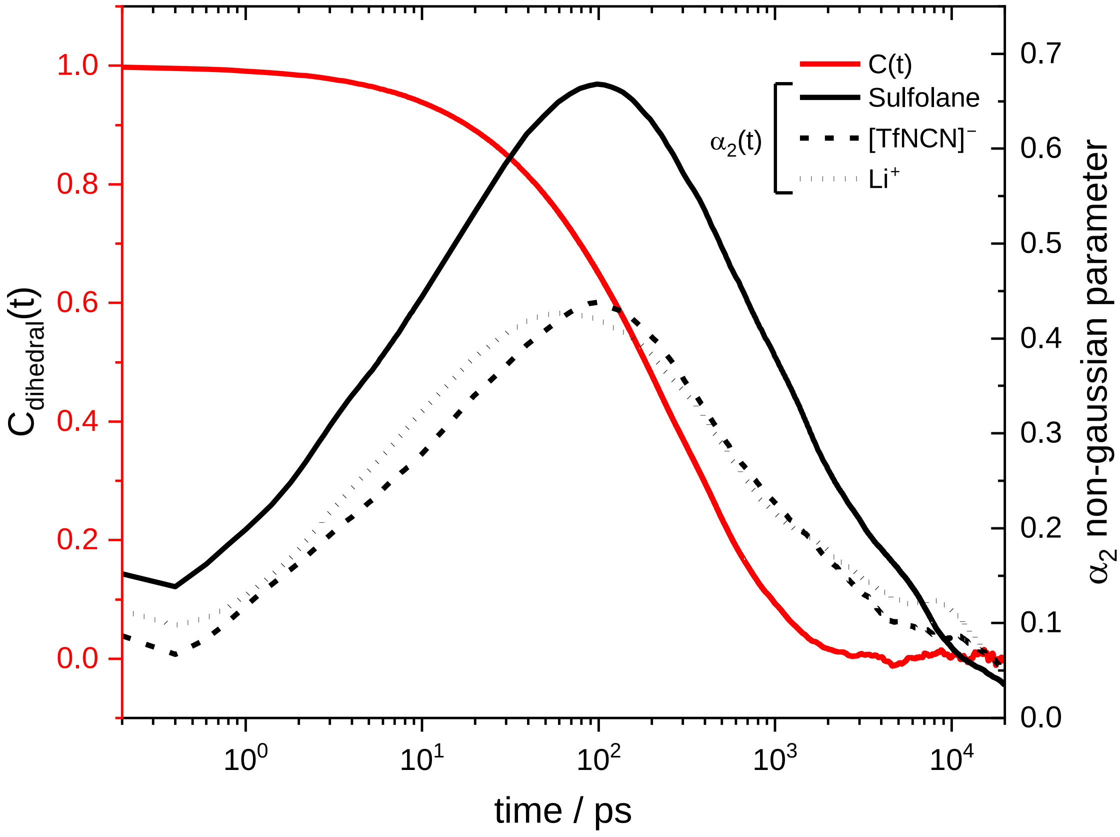

Figure S96: Non-gaussian parameter α_2 and intermittent dihedral autocorrelation function for $[Li(SL)_2][TfNCN]$, revealing timescales of relaxation.

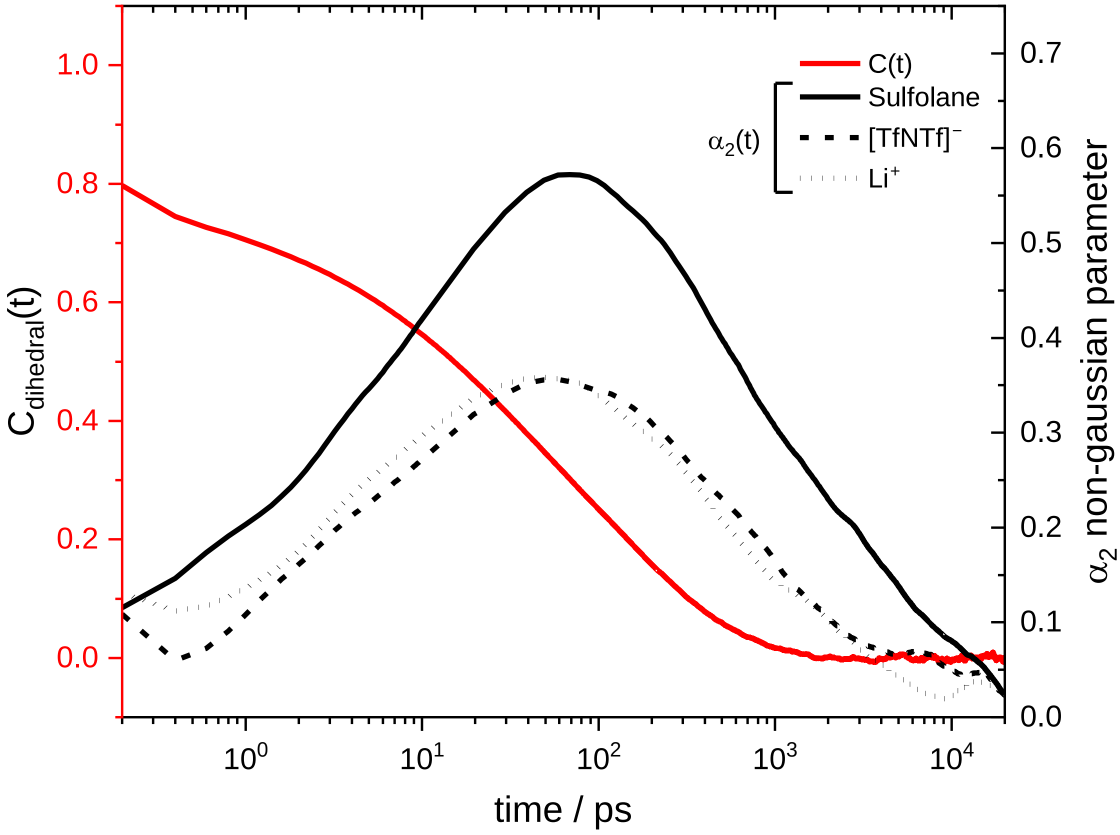

Figure S97: Non-gaussian parameter α_2 and intermittent dihedral autocorrelation function for $[Li(SL)_2][TfNTf]$, revealing timescales of relaxation.

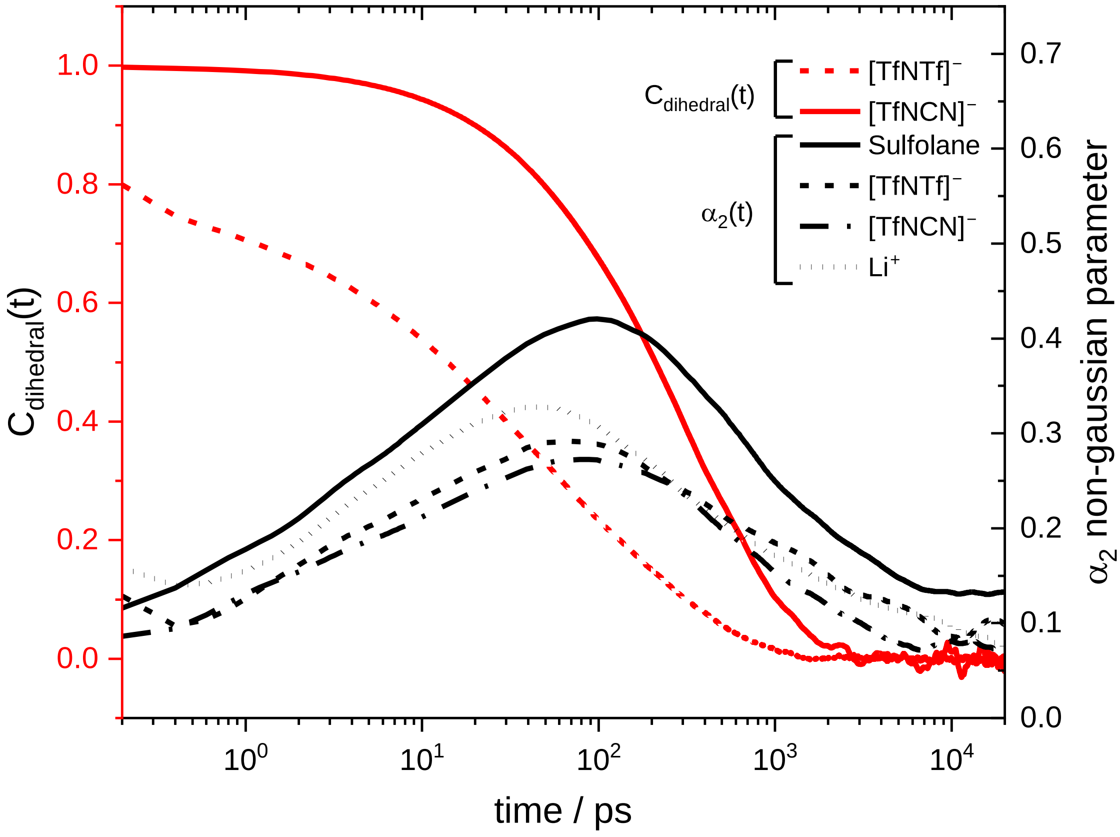

Figure S98: Non-gaussian parameter α_2 and intermittent dihedral autocorrelation function for [Li_2SL_4][TfNCN][TfNTf], revealing timescales of relaxation.

17.4. Static properties

Figure S99 shows a comparison of the lithium-lithium radial distribution functions from the MD simulations of [$Li(SL)_2$][TfNTf] and [$Li(SL)_2$][TfNCN]. It is evident that the latter shows a more diffuse coordination environment in the sense that more distances are significantly populated, while the pronounced peak at 4-5 Å is suppressed compared to [$Li(SL)_2$][TfNTf].

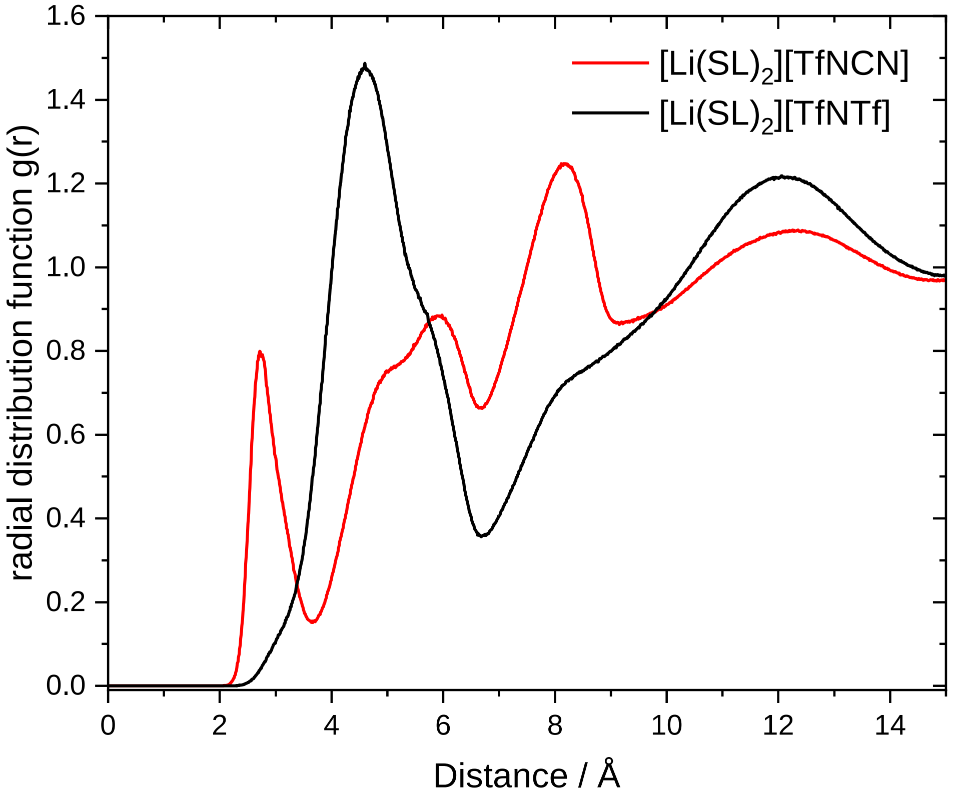

Figure S99: Lithium-lithium radial distribution functions from the MD simulations.

The small peak at 2.7 Å corresponds to clustering of lithium atoms, which also leaves a signature in the cluster count distribution function shown in Figure S100.[98]

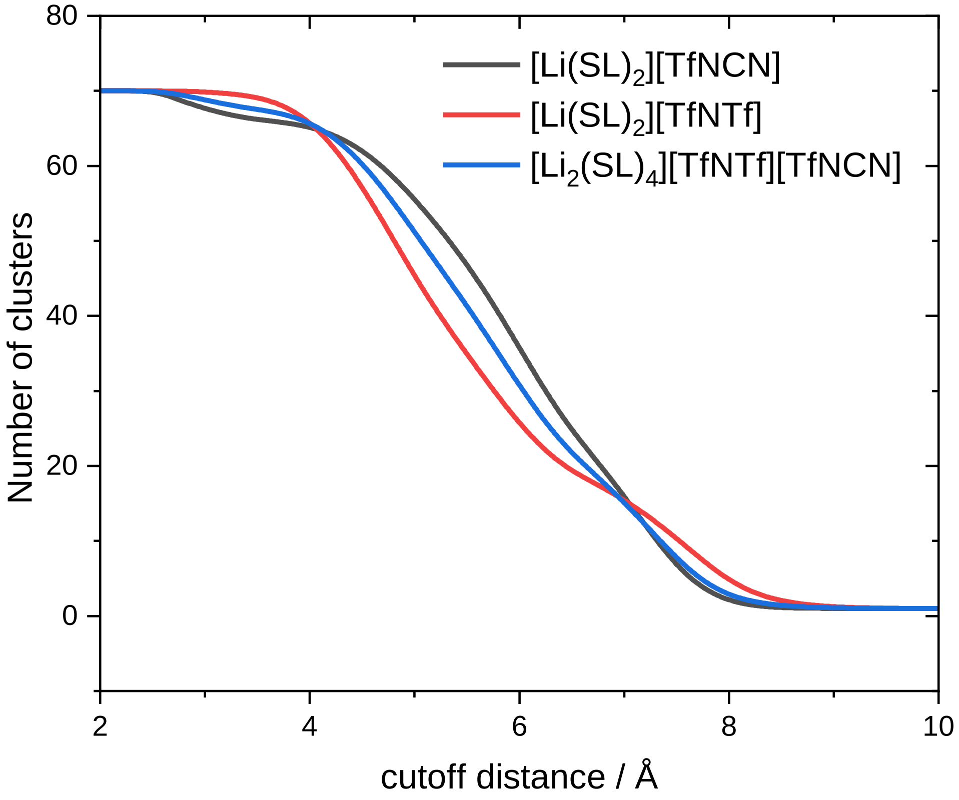

Figure S100: Cluster count distribution functions from the cluster analysis of lithium atoms in the MD simulations.

Based on the atom-wise radial distribution functions, we identified coordinating atoms (O and N) around lithium atoms and obtained their coordination number in the first coordination shell as the value of the corresponding number integral up to the minimum of the radial distribution function after the first peak. The relative contributions are shown in Figure S101 together with the coordination number, which is close to the expected value of $n_1 = 4$ in all cases.

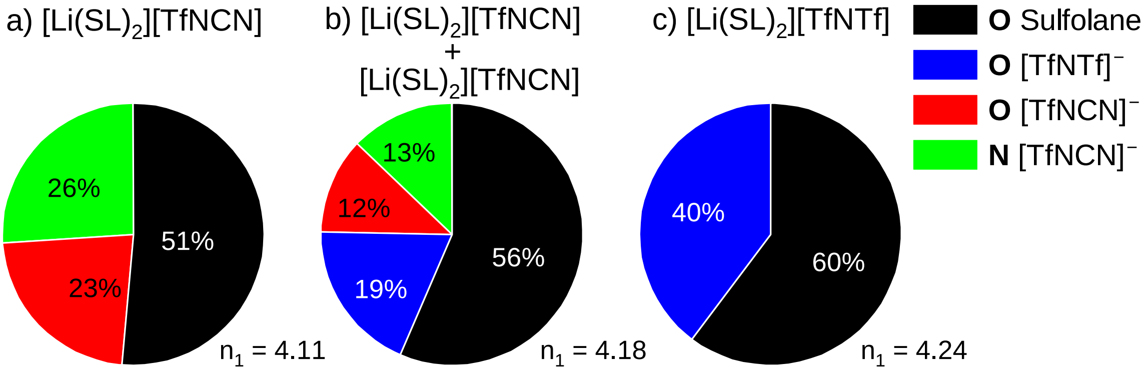

Figure S101: relative contributions to the first coordination shell (coordination number ≈ 4) around lithium atoms from the MD simulations, resolved by atom types.

It is noteworthy that the nitrile nitrogen atoms disproportionately contribute to the coordination sphere, despite their lower population. This can also be seen in the neighbourhood probability matrix from the Voronoi analysis, Figure S102.[97,99,116,117]

Within the framework of the Voronoi analysis, the probability that any of the neighbours of lithium is a nitrile nitrogen atom is 99.2%. Thus, assuming four neighbours, the probability of finding a nitrile nitrogen atom in a specific neighbour location is $P = 1 - \sqrt[4]{1 - 0.992} = 70\%$. The actual average neighbour count from the Voronoi analysis (nitrile N and all O atoms) was slightly higher with $n_1 = 4.35$ neighbours, leading to $P = 67\%$. Compared to this, the probability of finding lithium as a neighbour of a nitrile nitrogen atom from the Voronoi analysis was found to be 78%. The difference between these two numbers can be interpreted as nitrile induced lithium clustering, in the sense that there are bridging nitrile groups which have more than one lithium atom as neighbours.

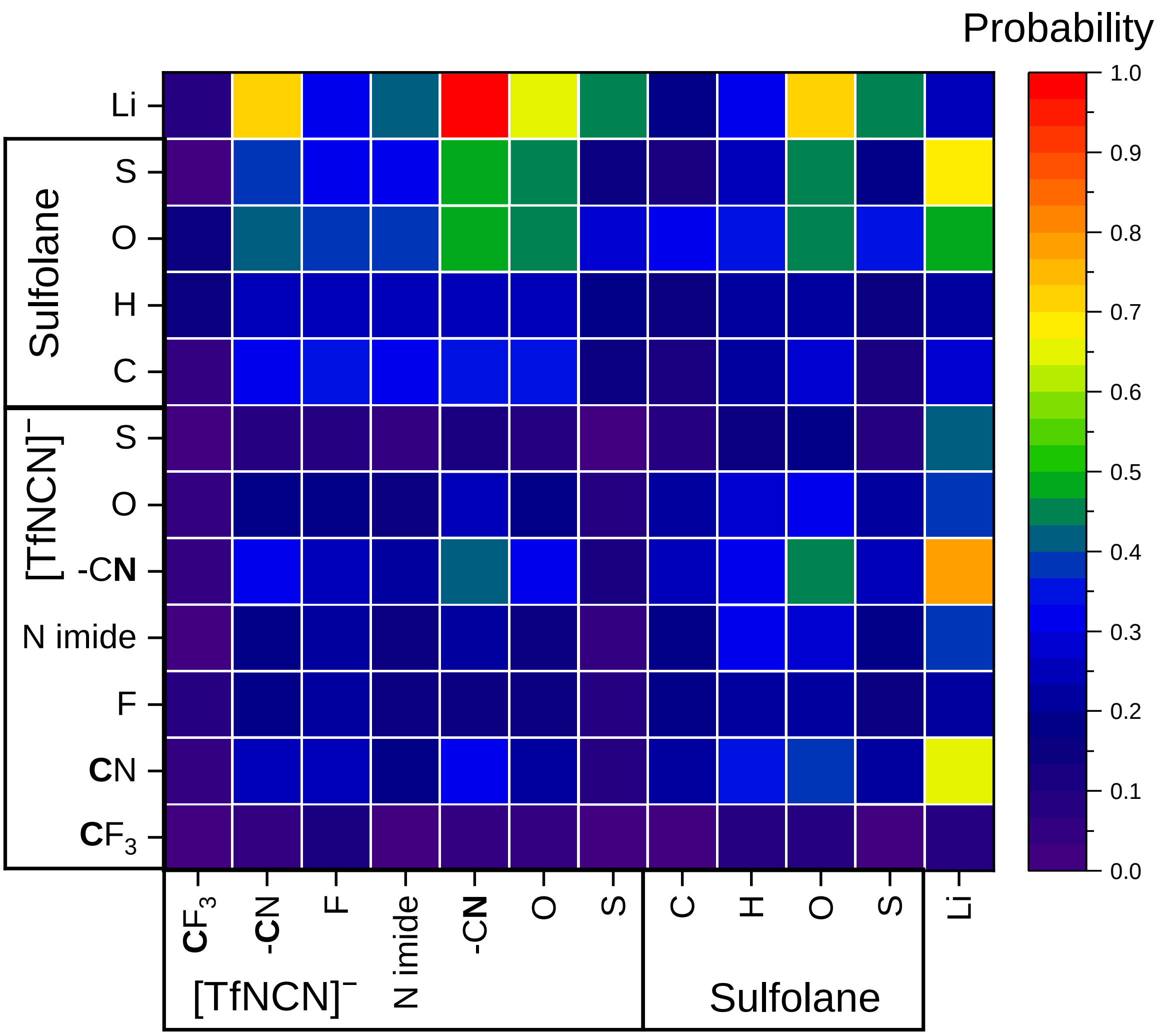

Figure S102: Heatmap showing the neighbourhood probabilities from a Voronoi analysis. Here, the probability of finding any number of nitrile-nitrogen atoms in the vicinity of a lithium atom is 99.2%. Thus, assuming a random distribution and four near neighbours around any lithium atom, the probability of finding a nitrile nitrogen atom in any given neighbour position is 70%.

Finally, assuming four neighbours and no cooperative coordination, the probabilities to find a certain number of anions coordinated to a specific lithium cation are summarised in Table S29.

Table S29: Probability of finding n anions coordinated to a single lithium cation.

	n = 0	n = 1	n = 2	n = 3	n = 4	n > 1
RDF (number integral)	7%	26%	37%	24%	6%	67%
Voronoi analysis	5%	21%	37%	29%	8%	74%

The results agree well across the two different analysis methods. The majority of lithium cations is coordinated by more than one anion.

17.5. Speciation Analysis

We performed the speciation analysis implemented in the prealpha software package to identify realistic input structures for the *ab initio* calculation of the interaction energies in Section 16.3. The speciation analysis works as follows:

1) Acceptor and donor atoms are selected. In this case, the acceptor atom was lithium, and the donor atoms were the Nitrile-N of the anion as well as the sulfonyl oxygen atoms of anion and sulfolane. For the sake of simplicity, other lithium atoms were not considered as donors.
2) Atoms are grouped together by elements. In other words, the two sulfonyl oxygen atoms in cation and anion are treated as identical, but the nitrile-N and sulfonyl-O are treated as different.
3) Cutoffs were defined as the respective minima in the atom-atom radial distribution functions.
4) For every Li acceptor atom, all donor atoms within the defined cutoff are identified. Every donor atom close enough to the acceptor atom is labelled as a connection.
5) From this list of connections, each acceptor is sorted into a list of species. Two different acceptors are only considered to be the same species if the number of neighbouring donor atoms, the number of neighbouring molecules, and the number of total connections are the same. In addition, all the connections to the acceptor are checked for the molecule type of the donor (*i.e.* anion *vs.* sulfolane), the atom group of the donor (O vs N), and the atom group of the acceptor (only Li in this case). If any of the connections of one acceptor does not have a counterpart in the other acceptor, then they are considered a different species.
6) While sorting the acceptors into the list of species, if no match is found, then a new species is created and appended to the list. If a match is found, then the count of that species is incremented by one.
7) At the end of the analysis, the species are sorted by most common (#1), second most common (#2), *etc.*, and the corresponding statistics are printed (see accompanying files).
8) For each species, the intermittent binary autocorrelation function $C(t)$ is calculated, Equation (S53). The binary identifier h for this species and a given acceptor molecule at a given time is 1 if the molecule belongs to this species, and 0 otherwise. The lifetime of the species can then be calculated from the decay of $C(t)$.

$$C(t) = \frac{\langle (h(t_0 + t) - \langle h \rangle)(h(t_0) - \langle h \rangle) \rangle}{\langle (h(t_0) - \langle h \rangle)^2 \rangle} \qquad \text{(S53)}$$

The resulting intermittent dihedral autocorrelation functions are summarised in Figure S103. The four most common species are shown in Figure S104. Common species are not necessarily also long lived, the longest lived species #12 occurs in less than 1% of the cases (Which is a lithium cation coordinated by four anions, two *via* the nitrile group and two via the sulfonyl group). We chose #3 and #4 as models for the *ab initio* calculation in Section 16.3. The reason for this choice is that these two species are common (≈10%), have a relatively long lifetime, and differ only in the coordination of the anion (nitrile *vs.* sulfonyl group).

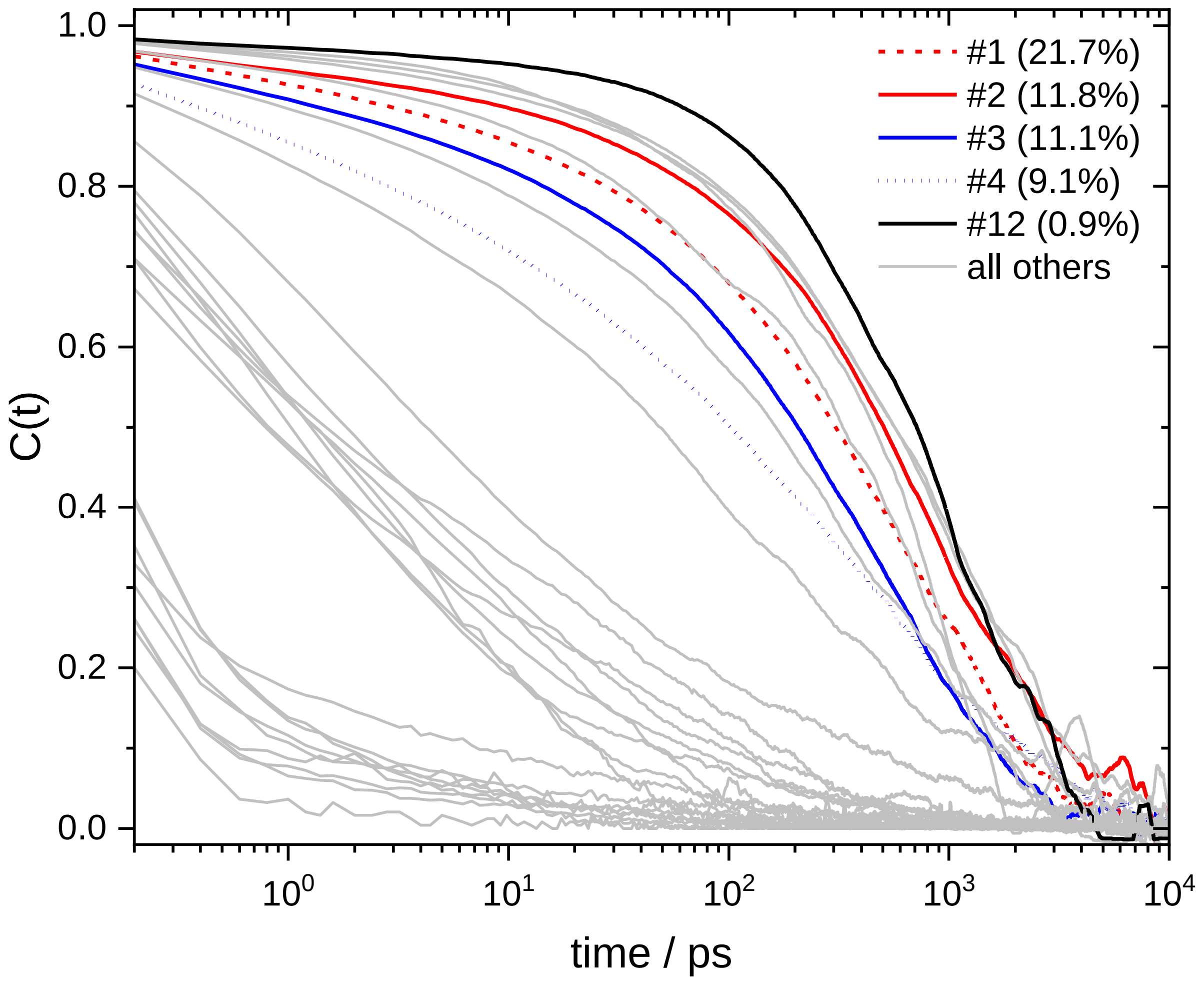

Figure S103: Intermittent binary autocorrelation function from the speciation analysis. The percentages in brackets give the proportion of this species.

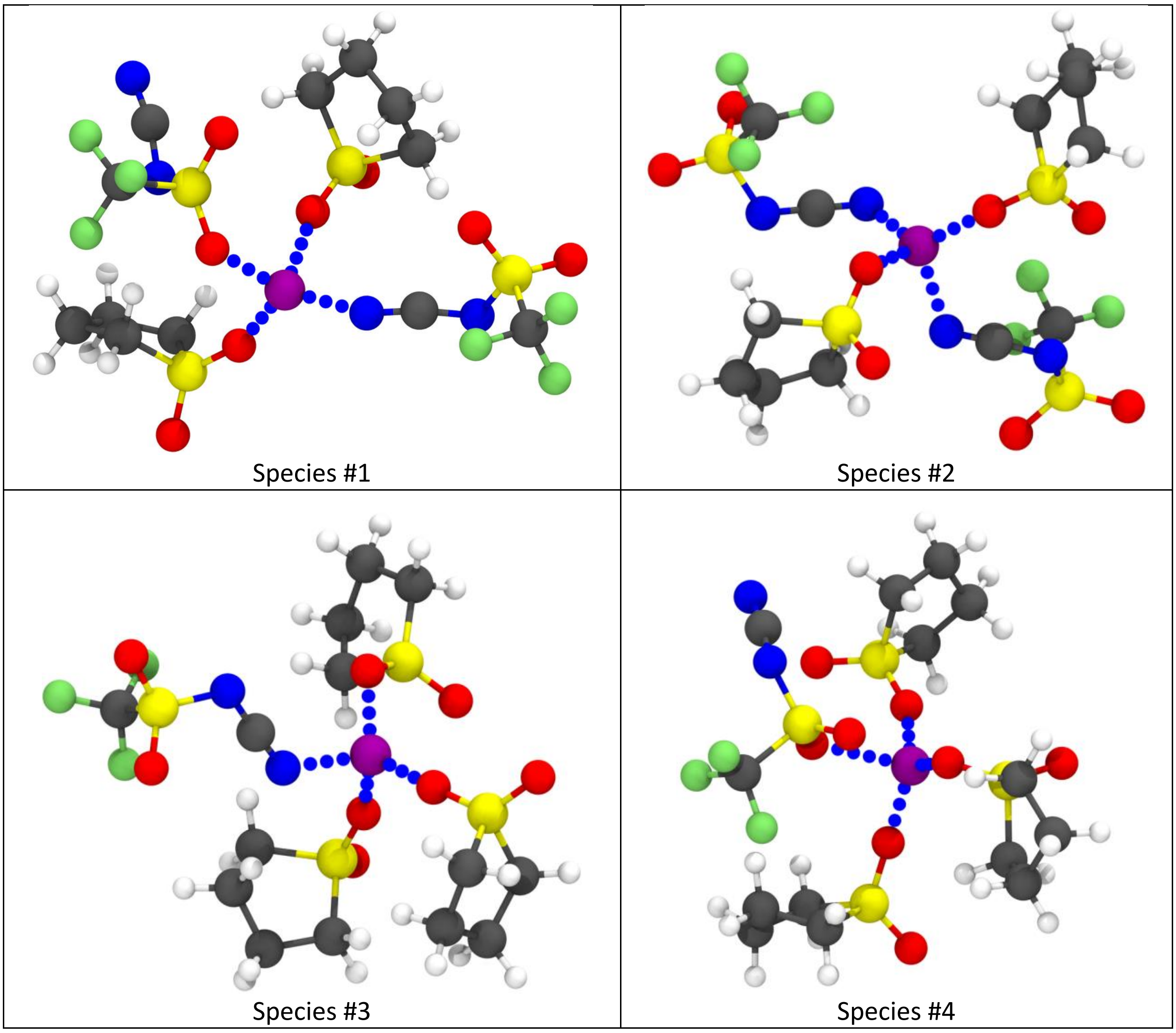

Figure S104: Key species identified in the speciation analysis (4 most common species, numbered by occurrence). For each species, the last occurrence in the trajectory is shown.

18. Crystallography

Crystals were obtained as described above. Datasets were collected using a Rigaku XtaLAB AFC12 Kappa dual offset diffractometer (Cu K_α radiation, 1.54184 Å). Data processing was carried out using CrysAlisPro,[118] solutions were solved and refined using Olex-2.[119] Hydrogen atoms were placed in geometrically assigned positions with X–H distances of 0.95 Å (CH), 0.97 Å (CH_2) or 0.98 Å (CH_3) and refined using a riding model, with $U_{iso}(H) = 1.2\ U_{eq}(C)$ (CH, CH_2), or $1.5\ U_{eq}(C)$ (CH_3). CCDC deposition numbers 2336048 (**LiTfNAc**), 2336049 (**LiTfN3O1-sulfolane**), 2336050 (**LiTfN3O1**), 2336051 (**Li(SL)TfN5**), 2336052 (**HCMs(CN)$_2$**) and 2336053 (**LiCMs(CN)$_2$**) contain crystallographic data in CIF format, which is also summarized below (Table S30). **LiTfNAc** was a polymeric structure which contained a very large number of formula units in the asymmetric unit (Z' = 15). No higher symmetry was detected and the data metrics were acceptable in the monoclinic space group *C*2. It was also refined as a two-component twin with matrix [-1 0 0 0 -1 0 0 0 -1] and BASF 0.017(19). Two CF_3 groups exhibited rotational disorder which was successfully modelled. **LiTfN3O1-sulfolane** had the sulfolane group disordered over two positions which was successfully modelled. **Li(SL)TfN5** exhibited positional disorder in the n-pentyl group. EADP and SADI restraints were needed to retain a sensible geometry in the alkyl chain. **LiCMs(CN)$_2$** had an acetone molecule which was positionally disordered across three distinct orientations. The occupations were fixed at 35%, 35%, 30% and ISOR was needed on one C atom.

Table S30: Summary of CIF data for structures reported in this paper.

	**LiTfNAc**	**LiTfN3O1 - sulfolane**	**LiTfN3O1**
Empirical formula	$C_3H_3F_3LiNO_3S$	$2(C_{10}H_{18}F_6Li_2N_2O_6S_2)\cdot C_4H_8SO_2$	$C_5H_9F_3LiNO_3S$
Formula weight (g / mol)	197.06	1028.69	227.13
Crystal system	monoclinic	monoclinic	triclinic
Space group	*C*2	*I*2/*a*	*P*–1
a (Å)	63.7924(10)	10.3980(2)	5.5440(2)
b (Å)	9.6005(1)	19.9938(3)	9.2231(2)
c (Å)	17.2224(2)	22.1730(4)	10.2431(2)
α (°)	90	90	80.255(2)
β (°)	90.202(1)	95.895(2)	83.499(2)
γ (°)	90	90	86.548(2)
Volume ($Å^3$)	10547.6(2)	4585.29(14)	512.43(2)
Z	60	4	2
Density (calc.) (g/cm^3)	1.861	1.490	1.472
Absorption coefficient	4.452	3.314	3.126
F(000)	5880	2112	232
2θ range (°)	10.27–143.54	8.85–143.72	8.81–143.53
Reflections collected	35303	11939	9087
Independent reflections	17816	4385	1966
Data / restraints / parameters	17816/15/1634	4385/0/296	1966/0/128
Goodness-of-fit on F^2	1.029	1.044	1.125
Final *R* indices [I>2σ(I)]	$R_1 = 0.0568$	$R_1 = 0.0543$	$R_1 = 0.0632$
	$wR_2 = 0.1364$	$wR_2 = 0.1543$	$wR_2 = 0.1781$
R indices (all data)	$R_1 = 0.0624$	$R_1 = 0.0593$	$R_1 = 0.0659$
	$wR_2 = 0.1406$	$wR_2 = 0.1601$	$wR_2 = 0.1828$
Largest diff. peak/hole ($eÅ^{-3}$)	0.79/–0.74	0.78/–0.47	0.43/–0.60

Table S30 (continued): Summary of CIF data for structures reported in this paper.

	**Li(SL)TfN5**	**HCMs(CN)$_2$**	**LiCMs(CN)$_2$**
Empirical formula	$C_{10}H_{19}F_3LiNO_4S_2$	$C_4H_4N_2O_2S$	$C_7H_9LiN_2O_3S$
Formula weight (g / mol)	345.32	144.15	208.16
Crystal system	monoclinic	triclinic	triclinic
Space group	*C*2/*c*	*P*–1	*P*–1
a (Å)	24.3672(12)	5.4317(2)	6.7137(3)
b (Å)	5.7733(2)	7.1254(3)	7.9569(3)
c (Å)	24.0521(8)	8.5194(3)	11.1702(4)
α (°)	90	95.165(3)	98.120(3)
β (°)	105.238(4)	96.669(3)	97.915(4)
γ (°)	90	104.049(3)	113.035(4)
Volume (Å^3)	3264.7(2)	315.30(2)	531.30(4)
Z	8	2	2
Density (calc.) (g/cm^3)	1.405	1.518	1.301
Absorption coefficient	3.367	3.987	2.586
F(000)	1440	148	216
2θ range (°)	7.52–144.84	10.53–143.10	12.37–143.58
Reflections collected	6485	5481	4630
Independent reflections	3103	1207	1993
Data / restraints / parameters	3103/1/186	1207/0/84	1993/6/188
Goodness-of-fit on F^2	1.115	1.068	1.103
Final *R* indices [I>2σ(I)]	R_1 = 0.0639	R_1 = 0.0308	R_1 = 0.0530
	wR_2 = 0.1895	wR_2 = 0.0795	wR_2 = 0.1348
R indices (all data)	R_1 = 0.0767	R_1 = 0.0314	R_1 = 0.0546
	wR_2 = 0.1994	wR_2 = 0.0799	wR_2 = 0.1364
Largest diff. peak/hole (eÅ^{-3})	0.39/–0.73	0.41/–0.36	0.42/–0.70

19. References

[1] B. F. Soares, D. R. Nosov, J. M. Pires, A. A. Tyutyunov, E. I. Lozinskaya, D. Y. Antonov, A. S. Shaplov, I. M. Marrucho, *Molecules* **2022**, *27*, 413.

[2] T. ZOU, L. LU, X.-L. LIU, Z. ZHANG, L.-B. WANG, X.-L. FU, G.-H. GAO, Y. KOU, M.-Y. HE, *Chinese J. Chem.* **2008**, *26*, 1469–1480.

[3] P. Nürnberg, E. I. Lozinskaya, A. S. Shaplov, M. Schönhoff, *J. Phys. Chem. B* **2020**, *124*, 861–870.

[4] D. Penley, X. Wang, Y.-Y. Lee, M. N. Garaga, R. Ghahremani, S. Greenbaum, E. J. Maginn, B. Gurkan, *J. Chem. Eng. Data* **2022**, *67*, 1810–1823.

[5] T. C. Lourenço, L. G. Dias, J. L. F. Da Silva, *ACS Appl. Energy Mater.* **2021**, *4*, 4444–4458.

[6] T. Mendez-Morales, Z. Li, M. Salanne, *Batter. Supercaps* **2021**, *4*, 646–652.

[7] T. Rüther, A. I. Bhatt, A. S. Best, K. R. Harris, A. F. Hollenkamp, *Batter. Supercaps* **2020**, *3*, 793–827.

[8] L. Schkeryantz, P. Nguyen, W. D. McCulloch, C. E. Moore, K. C. Lau, Y. Wu, *J. Phys. Chem. C* **2022**, *126*, 11407–11413.

[9] L. A. Schkeryantz, J. Zheng, W. D. McCulloch, L. Qin, S. Zhang, C. E. Moore, Y. Wu, *Chem. Mater.* **2020**, *32*, 10423–10434.

[10] R. E. A. Dillon, C. L. Stern, D. F. Shriver, *Chem. Mater.* **2000**, *12*, 1122–1126.

[11] P. Bonhôte, A.-P. Dias, M. Armand, N. Papageorgiou, K. Kalyanasundaram, M. Grätzel, *Inorg. Chem.* **1996**, *35*, 1168–1178.

[12] J. M. Pringle, J. Golding, K. Baranyai, C. M. Forsyth, G. B. Deacon, J. L. Scott, D. R. MacFarlane, *New J. Chem.* **2003**, *27*, 1504–1510.

[13] B. Helferich, H. Grünert, *Berichte der Dtsch. Chem. Gesellschaft (A B Ser.* **1940**, *73*, 1131–1133.

[14] F. Philippi, D. Rauber, O. Palumbo, K. Goloviznina, J. McDaniel, D. Pugh, S. Suarez, C. C. Fraenza, A. Padua, C. W. M. Kay, T. Welton, *Chem. Sci.* **2022**, *13*, 9176–9190.

[15] D. A. Osborne, M. Breedon, T. Rüther, M. J. S. Spencer, *J. Mater. Chem. A* **2022**, *10*, 13254–13265.

[16] Y. K. J. Bejaoui, F. Philippi, H.-G. Stammler, K. Radacki, L. Zapf, N. Schopper, K. Goloviznina, K. A. M. Maibom, R. Graf, J. A. P. Sprenger, R. Bertermann, H. Braunschweig, T. Welton, N. V. Ignat'ev, M. Finze, *Chem. Sci.* **2023**, *14*, 2200–2214.

[17] M. Zhang, T. Sonoda, M. Mishima, T. Honda, I. Leito, I. A. Koppel, W. Bonrath, T. Netscher, *J. Phys. Org. Chem.* **2014**, *27*, 676–679.

[18] T. Tamura, T. Hachida, K. Yoshida, N. Tachikawa, K. Dokko, M. Watanabe, *J. Power Sources* **2010**, *195*, 6095–6100.

[19] J. Scheers, E. Jónsson, P. Jacobsson, P. Johansson, *Electrochemistry* **2012**, *80*, 18–25.

[20] T. Chen, B. S. Majumdar, *Main Gr. Chem.* **2016**, *15*, 375–382.

[21] H. Zhang, U. Oteo, H. Zhu, X. Judez, M. Martinez-Ibañez, I. Aldalur, E. Sanchez-Diez, C. Li, J. Carrasco, M. Forsyth, M. Armand, *Angew. Chemie Int. Ed.* **2019**, *58*, 7829–7834.

[22] R. Arnaud, D. Benrabah, J.-Y. Sanchez, *J. Phys. Chem.* **1996**, *100*, 10882–10891.

[23] D. BENRABAH, J. SANCHEZ, D. DEROO, M. ARMAND, *Solid State Ionics* **1994**, *70–71*, 157–162.

[24] P. Johansson, *J. Phys. Chem. A* **2001**, *105*, 9258–9264.

[25] D. BENRABAH, J. SANCHEZ, M. ARMAND, *Solid State Ionics* **1993**, *60*, 87–92.

[26] F. J. Waller, A. G. M. Barrett, D. C. Braddock, D. Ramprasad, R. M. McKinnell, A. J. P. White, D. J. Williams, R. Ducray, *J. Org. Chem.* **1999**, *64*, 2910–2913.

[27] P. Burk, I. A. Koppel, I. Koppel, L. M. Yagupolskii, R. W. Taft, *J. Comput. Chem.* **1996**, *17*, 30–41.

[28] B. Mandal, T. Sooksimuang, B. Griffin, A. Padhi, R. Filler, *Solid State Ionics* **2004**, *175*, 267–272.

[29] J. V. Silverton, D. T. Gibson, S. C. Abrahams, *Acta Crystallogr.* **1965**, *19*, 651–658.

[30] U. Oteo, M. Martinez-Ibañez, I. Aldalur, E. Sanchez-Diez, J. Carrasco, M. Armand, H. Zhang, *ChemElectroChem* **2019**, *6*, 1019–1022.

[31] D. Gyabeng, L. Qiao, H. Zhang, U. Oteo, M. Armand, M. Forsyth, F. Chen, L. A. O'Dell, *J. Phys. Chem. C* **2021**, *125*, 14818–14826.

[32] B. A. Fortuin, L. Meabe, S. R. Peña, Y. Zhang, L. Qiao, J. Etxabe, L. Garcia, H. Manzano, M. Armand, M. Martínez-Ibañez, J. Carrasco, *J. Phys. Chem. C* **2023**, *127*, 1955–1964.

[33] A. Santiago, J. Castillo, I. Garbayo, A. Saenz de Buruaga, J. A. Coca Clemente, L. Qiao, R. Cid Barreno, M. Martinez-Ibañez, M. Armand, H. Zhang, C. Li, *ACS Appl. Energy Mater.* **2021**, *4*, 4459–4464.

[34] D. Reber, N. Takenaka, R. S. Kühnel, A. Yamada, C. Battaglia, *J. Phys. Chem. Lett.* **2020**, 4720–4725.

[35] H. Sano, K. Kubota, Z. Siroma, S. Kuwabata, H. Matsumoto, *J. Electrochem. Soc.* **2020**, *167*, 070502.

[36] K. Kubota, H. Matsumoto, *J. Electrochem. Soc.* **2014**, *161*, A902–A907.

[37] J. Liu, T. Kaneko, J. Ock, S. Kondou, K. Ueno, K. Dokko, K. Sodeyama, M. Watanabe, *J. Phys. Chem. C* **2023**, *127*, 5689–5701.

[38] Y. Ugata, G. Hasegawa, N. Kuwata, K. Ueno, M. Watanabe, K. Dokko, *J. Phys. Chem. C* **2022**, *126*, 19084–19090.

[39] J. S. Ho, O. A. Borodin, M. S. Ding, L. Ma, M. A. Schroeder, G. R. Pastel, K. Xu, *ENERGY Environ. Mater.* **2023**, *6*, 1–9.

[40] F. Philippi, D. Pugh, D. Rauber, T. Welton, P. A. Hunt, *Chem. Sci.* **2020**, *11*, 6405–6422.

[41] H. Matsumoto, H. Kageyama, Y. Miyazaki, *Chem. Commun.* **2002**, 1726–1727.

[42] M. B. Herath, T. Hickman, S. E. Creager, D. D. DesMarteau, *J. Fluor. Chem.* **2011**, *132*, 52–56.

[43] Y. Li, B. Cheng, F. Jiao, K. Wu, *ACS Appl. Mater. Interfaces* **2020**, *12*, 16298–16307.

[44] A. M. Borys, *Organometallics* **2023**, *42*, 182–196.

[45] W. L. F. Armarego, in *Purif. Lab. Chem.*, Elsevier, **2017**, pp. 95–634.

[46] W. L. F. Armarego, in *Purif. Lab. Chem.*, Elsevier, **2017**, pp. 1–70.

[47] R. Dijkstra, H. J. Backer, *Recl. des Trav. Chim. des Pays-Bas* **1954**, *73*, 569–574.

[48] C. T. Supuran, A. Scozzafava, F. Briganti, *J. Enzyme Inhib.* **1999**, *14*, 289–306.

[49] S. Lascaud, M. Perrier, A. Vallee, S. Besner, J. Prud'homme, M. Armand, *Macromolecules* **1994**, *27*, 7469–7477.

[50] M. T. Martin, F. Roschangar, J. F. Eaddy, *Tetrahedron Lett.* **2003**, *44*, 5461–5463.

[51] J. Perchais, J.-P. Fleury, *Tetrahedron* **1974**, *30*, 999–1009.

[52] V. Jacob, S. Mann, G. Huttner, O. Walter, L. Zsolnai, E. Kaifer, P. Rutsch, P. Kircher, E. Bill, *Eur. J. Inorg. Chem.* **2001**, *2001*, 2625–2640.

[53] M. Della Monica, L. Jannelli, U. Lamanna, *J. Phys. Chem.* **1968**, *72*, 1068–1071.

[54] O. Palumbo, A. Paolone, F. Philippi, D. Rauber, T. Welton, *Int. J. Mol. Sci.* **2023**, *24*, 11046.

[55] O. Palumbo, F. Trequattrini, F. M. Vitucci, A. Paolone, *J. Phys. Chem. B* **2015**, *119*, 12905–12911.

[56] S. Tsuzuki, S. Ikeda, W. Shinoda, K. Shigenobu, K. Ueno, K. Dokko, M. Watanabe, *J. Phys. Chem. B* **2023**, *127*, 6333–6341.

[57] P. Ravn Sørensen, T. Jacobsen, *Electrochim. Acta* **1982**, *27*, 1671–1675.

[58] K. Shigenobu, K. Dokko, M. Watanabe, K. Ueno, *Phys. Chem. Chem. Phys.* **2020**, *22*, 15214–15221.

[59] D. B. Shah, H. Q. Nguyen, L. S. Grundy, K. R. Olson, S. J. Mecham, J. M. DeSimone, N. P. Balsara, *Phys. Chem. Chem. Phys.* **2019**, *21*, 7857–7866.

[60] G. H. Sørland, *Dynamic Pulsed-Field-Gradient NMR*, Springer Berlin Heidelberg, Berlin, Heidelberg, **2014**.

[61] C. S. Johnson, *Prog. Nucl. Magn. Reson. Spectrosc.* **1999**, *34*, 203–256.

[62] E. O. Stejskal, J. E. Tanner, *J. Chem. Phys.* **1965**, *42*, 288–292.

[63] H. K. Kashyap, H. V. R. Annapureddy, F. O. Raineri, C. J. Margulis, *J. Phys. Chem. B* **2011**, *115*, 13212–13221.

[64] A. Nakanishi, K. Ueno, D. Watanabe, Y. Ugata, Y. Matsumae, J. Liu, M. L. Thomas, K. Dokko, M. Watanabe, *J. Phys. Chem. C* **2019**, *123*, 14229–14238.

[65] M. Gouverneur, J. Kopp, L. van Wüllen, M. Schönhoff, *Phys. Chem. Chem. Phys.* **2015**, *17*, 30680–30686.

[66] S. Pfeifer, F. Ackermann, F. Sälzer, M. Schönhoff, B. Roling, *Phys. Chem. Chem. Phys.* **2021**, *23*, 628–640.

[67] S. Ikeda, S. Tsuzuki, T. Sudoh, K. Shigenobu, K. Ueno, K. Dokko, M. Watanabe, W. Shinoda, *J. Phys. Chem. C* **2023**, *127*, 13837–13845.

[68] N. M. Vargas-Barbosa, B. Roling, *ChemElectroChem* **2020**, *7*, 367–385.

[69] D. Dong, F. Sälzer, B. Roling, D. Bedrov, *Phys. Chem. Chem. Phys.* **2018**, *20*, 29174–29183.

[70] T. Sudoh, S. Ikeda, K. Shigenobu, S. Tsuzuki, K. Dokko, M. Watanabe, W. Shinoda, K. Ueno, *J. Phys. Chem. C* **2023**, *127*, 12295–12303.

[71] Gaussian 09, Revision E.01, M. J. Frisch, G. W. Trucks, H. B. Schlegel, G. E. Scuseria, M. A. Robb, J. R. Cheeseman, G. Scalmani, V. Barone, B. Mennucci, G. A. Petersson, H. Nakatsuji, M. Caricato, X. Li, H. P. Hratchian, A. F. Izmaylov, J. Bloino, G. Zheng, J. L. Sonnenberg, M. Hada, M. Ehara, K. Toyota, R. Fukuda, J. Hasegawa, M. Ishida, T. Nakajima, Y. Honda, O. Kitao, H.

Nakai, T. Vreven, J. A. Montgomery, Jr., J. E. Peralta, F. Ogliaro, M. Bearpark, J. J. Heyd, E. Brothers, K. N. Kudin, V. N. Staroverov, T. Keith, R. Kobayashi, J. Normand, K. Raghavachari, A. Rendell, J. C. Burant, S. S. Iyengar, J. Tomasi, M. Cossi, N. Rega, J. M. Millam, M. Klene, J. E. Knox, J. B. Cross, V. Bakken, C. Adamo, J. Jaramillo, R. Gomperts, R. E. Stratmann, O. Yazyev, A. J. Austin, R. Cammi, C. Pomelli, J. W. Ochterski, R. L. Martin, K. Morokuma, V. G. Zakrzewski, G. A. Voth, P. Salvador, J. J. Dannenberg, S. Dapprich, A. D. Daniels, O. Farkas, J. B. Foresman, J. V. Ortiz, J. Cioslowski, and D. J. Fox, Gaussian, Inc., Wallingford CT, **2013**.

[72] S. Tsuzuki, W. Shinoda, S. Seki, Y. Umebayashi, K. Yoshida, K. Dokko, M. Watanabe, *ChemPhysChem* **2013**, *14*, 1993–2001.

[73] A. Moser, K. Range, D. M. York, *J. Phys. Chem. B* **2010**, *114*, 13911–13921.

[74] N. Yao, X. Chen, X. Shen, R. Zhang, Z. Fu, X. Ma, X. Zhang, B. Li, Q. Zhang, *Angew. Chemie Int. Ed.* **2021**, *60*, 21473–21478.

[75] X. Chen, X. Zhang, H. Li, Q. Zhang, *Batter. Supercaps* **2019**, *2*, 128–131.

[76] A. V. Marenich, C. J. Cramer, D. G. Truhlar, *J. Phys. Chem. B* **2009**, *113*, 6378–6396.

[77] P. Zhou, W. Hou, Y. Xia, Y. Ou, H.-Y. Zhou, W. Zhang, Y. Lu, X. Song, F. Liu, Q. Cao, H. Liu, S. Yan, K. Liu, *ACS Nano* **2023**, *17*, 17169–17179.

[78] S. Mukherji, N. V. S. Avula, S. Balasubramanian, *ACS Omega* **2020**, *5*, 28285–28295.

[79] N. F. Carvalho, J. R. Pliego, *Phys. Chem. Chem. Phys.* **2015**, *17*, 26745–26755.

[80] T. Lu, F. Chen, *J. Comput. Chem.* **2012**, *33*, 580–592.

[81] T. Lu, F. Chen, *J. Mol. Graph. Model.* **2012**, *38*, 314–323.

[82] J. Zhang, *J. Chem. Theory Comput.* **2018**, *14*, 572–587.

[83] S. Manzetti, T. Lu, *J. Phys. Org. Chem.* **2013**, *26*, 473–483.

[84] D. G. A. Smith, L. A. Burns, A. C. Simmonett, R. M. Parrish, M. C. Schieber, R. Galvelis, P. Kraus, H. Kruse, R. Di Remigio, A. Alenaizan, A. M. James, S. Lehtola, J. P. Misiewicz, M. Scheurer, R. A. Shaw, J. B. Schriber, Y. Xie, Z. L. Glick, D. A. Sirianni, J. S. O'Brien, J. M. Waldrop, A. Kumar, E. G. Hohenstein, B. P. Pritchard, B. R. Brooks, H. F. Schaefer, A. Y. Sokolov, K. Patkowski, A. E. DePrince, U. Bozkaya, R. A. King, F. A. Evangelista, J. M. Turney, T. D. Crawford, C. D. Sherrill, *J. Chem. Phys.* **2020**, *152*, DOI 10.1063/5.0006002.

[85] K. Goloviznina, E. Bakis, F. Philippi, N. Scaglione, T. Rekis, L. Laimina, M. Costa Gomes, A. Padua, *J. Phys. Chem. Lett.* **2024**, *15*, 248–253.

[86] W. Shinoda, **2022**, Okayama.

[87] S. Nosé, *Mol. Phys.* **1984**, *52*, 255–268.

[88] W. G. Hoover, *Phys. Rev. A* **1985**, *31*, 1695–1697.

[89] G. J. Martyna, M. L. Klein, M. Tuckerman, *J. Chem. Phys.* **1992**, *97*, 2635–2643.

[90] H. C. Andersen, *J. Chem. Phys.* **1980**, *72*, 2384–2393.

[91] J.-P. Ryckaert, G. Ciccotti, H. J. C. Berendsen, *J. Comput. Phys.* **1977**, *23*, 327–341.

[92] M. Tuckerman, B. J. Berne, G. J. Martyna, *J. Chem. Phys.* **1992**, *97*, 1990–2001.

[93] G. J. Martyna, M. E. Tuckerman, D. J. Tobias, M. L. Klein, *Mol. Phys.* **1996**, *87*, 1117–1157.

[94] M. P. Allen, D. J. Tildesley, *Computer Simulation of Liquids*, Oxford University Press, **2017**.

[95] G. Kaminski, W. L. Jorgensen, *J. Phys. Chem.* **1996**, *100*, 18010–18013.

[96] W. L. Jorgensen, D. S. Maxwell, J. Tirado-Rives, *J. Am. Chem. Soc.* **1996**, *118*, 11225–11236.

[97] M. Brehm, B. Kirchner, *J. Chem. Inf. Model.* **2011**, *51*, 2007–2023.

[98] T. Frömbgen, J. Blasius, V. Alizadeh, A. Chaumont, M. Brehm, B. Kirchner, *J. Chem. Inf. Model.* **2022**, *62*, 5634–5644.

[99] M. Brehm, M. Thomas, S. Gehrke, B. Kirchner, *J. Chem. Phys.* **2020**, *152*, 164105.

[100] W. Humphrey, A. Dalke, K. Schulten, *J. Mol. Graph.* **1996**, *14*, 33–38.

[101] B. Doherty, X. Zhong, S. Gathiaka, B. Li, O. Acevedo, *J. Chem. Theory Comput.* **2017**, *13*, 6131–6145.

[102] M. L. P. Price, D. Ostrovsky, W. L. Jorgensen, *J. Comput. Chem.* **2001**, *22*, 1340–1352.

[103] S. V. Sambasivarao, O. Acevedo, *J. Chem. Theory Comput.* **2009**, *5*, 1038–1050.

[104] U. C. Singh, P. A. Kollman, *J. Comput. Chem.* **1984**, *5*, 129–145.

[105] B. H. Besler, K. M. Merz, P. A. Kollman, *J. Comput. Chem.* **1990**, *11*, 431–439.

[106] O. Borodin, G. D. Smith, *J. Phys. Chem. B* **2006**, *110*, 6279–6292.

[107] J. N. Canongia Lopes, A. A. H. Pádua, *Theor. Chem. Acc.* **2012**, *131*, 1129.

[108] J. N. Canongia Lopes, J. Deschamps, A. A. H. Pádua, *J. Phys. Chem. B* **2004**, *108*, 2038–2047.

[109] A. A. H. Pádua, *J. Phys. Chem. A* **2002**, *106*, 10116–10123.

[110] B. Vorselaars, A. V. Lyulin, K. Karatasos, M. A. J. Michels, *Phys. Rev. E - Stat. Nonlinear, Soft Matter Phys.* **2007**, *75*, 011504.

[111] A. Rahman, *Phys. Rev.* **1964**, *136*, A405–A411.

[112] B. P. Bhowmik, I. Tah, S. Karmakar, *Phys. Rev. E* **2018**, *98*, 022122.

[113] R. Richert, *J. Phys. Condens. Matter* **2002**, *14*, 201.

[114] A. Luzar, *J. Chem. Phys.* **2000**, *113*, 10663–10675.

[115] R. J. Gowers, P. Carbone, *J. Chem. Phys.* **2015**, *142*, 224907.

[116] M. Brehm, M. Thomas, *Molecules* **2021**, *26*, 1875.

[117] M. Brehm, H. Weber, M. Thomas, O. Hollóczki, B. Kirchner, *ChemPhysChem* **2015**, *16*, 3271–3277.

[118] CrysAlis Pro: Agilent, **2014**, Agilent Technologies Ltd, Yarnton, Oxfordshire, UK.

[119] O. V. Dolomanov, L. J. Bourhis, R. J. Gildea, J. A. K. Howard, H. Puschmann, *J. Appl. Crystallogr.* **2009**, *42*, 339–341.
